# Supplementary material for: Enteropathogen antibody dynamics and force of infection among children in low-resource settings
Source: eLife. 2019 Aug 19;8:e45594. doi: 10.7554/eLife.45594 (PMC6746552; doi:10.7554/eLife.45594)
Supplement: Supplementary file 1. [file elife-45594-supp1.zip › SIFile1.html]

Enteropathogen antibody dynamics and force of infection among children in low-resource settings


Code 

- Show All Code
- Hide All Code

# Enteropathogen antibody dynamics and force of infection among children in low-resource settings

### Supplementary Information File 1. Effect of interventions, bead lots, and season on enteropathogen antibody response.

# Notebook summary

This notebook compares antibody measurements among children assigned to different treatment arms in the Asembo, Kenya household water filter trial and in the Kongwa, Tanzania annual azithromycin distribution trial. For additional details on the interventions and design, please see the main text and previously published articles related to the trials:

Morris, J. F., Murphy, J., Fagerli, K., Schneeberger, C., Jaron, P., Moke, F., Juma, J., Ochieng, J. B., Omore, R., Roellig, D., Xiao, L., Priest, J. W., Narayanan, J., Montgomery, J. M., Hill, V., Mintz, E., Ayers, T. L. & O’Reilly, C. E. A Randomized Controlled Trial to Assess the Impact of Ceramic Water Filters on Prevention of Diarrhea and Cryptosporidiosis in Infants and Young Children-Western Kenya, 2013. *Am. J. Trop. Med. Hyg.* 98, 1260–1268 (2018). https://www.ncbi.nlm.nih.gov/pubmed/29611500

Wilson, N., Goodhew, B., Mkocha, H., Joseph, K., Bandea, C., Black, C., Igietseme, J., Munoz, B., West, S. K., Lammie, P., Kasubi, M. & Martin, D. L. Evaluation of a Single Dose of Azithromycin for Trachoma in Low-Prevalence Communities. *Ophthalmic Epidemiol.* 26, 1–6 (2019). https://www.ncbi.nlm.nih.gov/pubmed/30543311

The results show that the interventions did not effect enteropathogen antibody response. Based on these results, we pooled data across treatment arms in all analyses.

The second section of this notebook stratifies the analyses from Tanzania in years 1 versus years 2-4. The Tanania study used different bead lots in year 1 versus years 2-4 of the study. Different bead lots could theoretically have different antigen coupling efficiency, which could introduce differences in the MFI-bg measurements, since they are on an arbitrary scale that depends on how much antibody from the sample binds to the beads. Results of the stratified analyses show that the patterns observed were qualitatively and quantitatively similar for year 1 (n=902) versus years 2-4 (n=4,087). Owing to the much larger number of samples from later years, the results of this analysis also show that the pooled results match almost exactly the estimates from years 2-4.

The third section of this notebook examines the influence of rainy versus dry season in the Leogane, Haiti cohort (the only study with measurements that spanned dry and rainy seasons). The results suggest slightly higher IgG levels and seroprevalence for many pathogens during the wet season compared with the dry season. The increases are of small magnitude, however. For example, increases in seroprevalence associated with the wet season ranged from 1 to 4 percentage points.

# Script preamble

```
#-----------------------------
# preamble
#-----------------------------
library(here)
```

```
## here() starts at /Users/benarnold/enterics-seroepi
```

```
here::here()
```

```
## [1] "/Users/benarnold/enterics-seroepi"
```

```
# load packages
library(tidyverse)
```

```
## ── Attaching packages ────────────────────────────── tidyverse 1.2.1 ──
```

```
## ✔ ggplot2 3.1.1       ✔ purrr   0.3.2  
## ✔ tibble  2.1.1       ✔ dplyr   0.8.0.1
## ✔ tidyr   0.8.3       ✔ stringr 1.4.0  
## ✔ readr   1.1.1       ✔ forcats 0.3.0
```

```
## ── Conflicts ───────────────────────────────── tidyverse_conflicts() ──
## ✖ dplyr::filter() masks stats::filter()
## ✖ dplyr::lag()    masks stats::lag()
```

```
library(tmleAb) # development package: devtools::install_github("ben-arnold/tmleAb")
```

```
## Welcome to the tmleAb package
## Targeted maximum likelihood estimation for antibody measurements.
## (Version 0.3.4, release date 2017-12-19)
## 
## Periodically check for the latest development version using 
## devtools::install_github('ben-arnold/tmleAb')  
## 
## This software is based on work funded by the
## National Institute of Allergy and Infectious Diseases
## grant K01-AI119180
```

```
library(mgcv)
```

```
## Loading required package: nlme
```

```
## 
## Attaching package: 'nlme'
```

```
## The following object is masked from 'package:dplyr':
## 
##     collapse
```

```
## This is mgcv 1.8-27. For overview type 'help("mgcv-package")'.
```

```
library(knitr)
library(kableExtra)
```

```
## 
## Attaching package: 'kableExtra'
```

```
## The following object is masked from 'package:dplyr':
## 
##     group_rows
```

```
  options(knitr.table.format = "html") 

# set up for parallel computing
library(foreach)
```

```
## 
## Attaching package: 'foreach'
```

```
## The following objects are masked from 'package:purrr':
## 
##     accumulate, when
```

```
library(doParallel)
```

```
## Loading required package: iterators
```

```
## Loading required package: parallel
```

```
registerDoParallel(cores = detectCores() - 1)


# bright color blind palette:  https://personal.sron.nl/~pault/ 
cblack <- "#000004FF"
cblue <- "#3366AA"
cteal <- "#11AA99"
cgreen <- "#66AA55"
cchartr <- "#CCCC55"
cmagent <- "#992288"
cred <- "#EE3333"
corange <- "#EEA722"
cyellow <- "#FFEE33"
cgrey <- "#777777"

# safe color blind palette
# http://jfly.iam.u-tokyo.ac.jp/color/
# http://www.cookbook-r.com/Graphs/Colors_(ggplot2)/
cbPalette <- c("#999999", "#E69F00", "#56B4E9", "#009E73", "#F0E442", "#0072B2", "#D55E00", "#CC79A7")
```

# Asembo, Kenya Intervention Effects

## Load the data and format

```
#-----------------------------
# load the formatted data
# with seropositivity indicators
# created with:
# asembo-enteric-ab-data-format.Rmd -->
# asembo-enteric-ab-distributions.Rmd
#-----------------------------
dl <- readRDS(here::here("data","asembo_analysis2.rds"))

# list the enteric antigens and make formatted labels for them
mbavars <- c("vsp3","vsp5","cp17","cp23","leca","salb","sald","p18","p39","etec","cholera")

mbalabs <- c("Giardia VSP-3","Giardia VSP-5","Cryptosporidium Cp17","Cryptosporidium Cp23","E. histolytica LecA","Salmonella LPS B","Salmonella LPS D","Campylobacter p18","Campylobacter p39","ETEC LT B subunit","Cholera toxin B subunit")
```

```
#-----------------------------
# create composite 
# seropositivity indicators
# that use information from
# multiple antigens
#-----------------------------

dgi <- dl %>% 
  filter(antigen %in% c("vsp3","vsp5")) %>%
  select(childid,time,tr,sex,age,agediff,pathogen,antigen,seropos) %>%
  spread(antigen,seropos) %>%
  mutate(seropos=ifelse(vsp3==1|vsp5==1,1,0),
         antigen="vsp3vsp5",
         antigenf="Giardia VSP-3 or VSP-5") %>%
  select(-vsp3,-vsp5)

dcr <- dl %>% 
  filter(antigen %in% c("cp17","cp23")) %>%
  select(childid,time,tr,sex,age,agediff,pathogen,antigen,seropos) %>%
  spread(antigen,seropos) %>%
  mutate(seropos=ifelse(cp17==1|cp23==1,1,0),
         antigen="cp17cp23",
         antigenf="Cryptosporidium Cp17 or Cp23") %>%
  select(-cp17,-cp23)

ds <- dl %>% 
  filter(antigen %in% c("salb","sald")) %>%
  select(childid,time,tr,sex,age,agediff,pathogen,antigen,seropos) %>%
  spread(antigen,seropos) %>%
  mutate(seropos=ifelse(salb==1|sald==1,1,0),
         antigen="salbsald",
         antigenf="Salmonella LPS groups B or D") %>%
  select(-salb,-sald)

dcp <- dl %>% 
  filter(antigen %in% c("p18","p39")) %>%
  select(childid,time,tr,sex,age,agediff,pathogen,antigen,seropos) %>%
  spread(antigen,seropos) %>%
  mutate(seropos=ifelse(p18==1|p39==1,1,0),
         antigen="p18p39",
         antigenf="Campylobacter p18 or p39") %>%
  select(-p18,-p39)


dlp <- bind_rows(dl,dgi,dcr,ds,dcp) %>%
  mutate(antigenf=factor(antigenf,
                         levels=c("Giardia VSP-3 or VSP-5",
                                  "Cryptosporidium Cp17 or Cp23",
                                  "E. histolytica LecA",
                                  "Salmonella LPS groups B or D",
                                  "ETEC LT B subunit",
                                  "Cholera toxin B subunit",
                                  "Campylobacter p18 or p39")) ) %>%
  filter(!is.na(antigenf)) %>%
  select(pathogen,antigen,antigenf,
         childid,time,tr,sex,age,agediff,
         mfi,logmfi,logmfidiff,
         roccut,mixcut,unexp,unexpcut,serocut,serocut_desc,
         posroc,posmix,posunex,seropos) %>%
  arrange(pathogen,antigenf,childid,time)

#-----------------------------
# create a dummy variable equal
# to 1 for all obs as a trick to
# get marginal predictions by
# age while still including REs
# for child
#-----------------------------
dlp <- dlp %>%
  mutate(dummy=1)
```

## Estimate curves in each treatment arm

```
#----------------------------------
# simulataneous CIs for GAMs
# estimated by resampling the 
# Baysian posterior estimates of
# the variance-covariance matrix
# assuming that it is multivariate normal
# the function below also estimates 
# the unconditional variance-covariance
# matrix, Vb=vcov(x,unconditional=TRUE), 
# which allows for undertainty in the actual
# estimated mean as well 
# (Marra & Wood 2012 Scandinavian Journal of Statistics, 
#  Vol. 39: 53–74, 2012, doi: 10.1111/j.1467-9469.2011.00760.x )
# simultaneous CIs provide much better coverage than pointwise CIs
# see: http://www.fromthebottomoftheheap.net/2016/12/15/simultaneous-interval-revisited/
#----------------------------------

gamCI <- function(m,newdata,nreps=10000) {
  require(mgcv)
  require(dplyr)
  Vb <- vcov(m,unconditional = TRUE)
  pred <- predict(m, newdata, se.fit = TRUE)
  fit <- pred$fit
  se.fit <- pred$se.fit
  BUdiff <- MASS::mvrnorm(n=nreps, mu = rep(0, nrow(Vb)), Sigma = Vb)
  Cg <- predict(m, newdata, type = "lpmatrix")
  simDev <- Cg %*% t(BUdiff)
  absDev <- abs(sweep(simDev, 1, se.fit, FUN = "/"))
  masd <- apply(absDev, 2L, max)
  crit <- quantile(masd, prob = 0.95, type = 8)
  pred <- data.frame(newdata,fit=pred$fit,se.fit=pred$se.fit)
  pred <- mutate(pred,
                 uprP = fit + (2 * se.fit),
                 lwrP = fit - (2 * se.fit),
                 uprS = fit + (crit * se.fit),
                 lwrS = fit - (crit * se.fit)
  )
  return(pred)
}


#-----------------------------
# fit cubic spline over age, separately for each 
# treatment arm and antigen
# estimate approximate simultaneous confidence intervals
# for the fits
# set the dummy to 0 to 
# zero out all of the random effects
# see posts on Stack Exchange for explanation:
# https://stats.stackexchange.com/questions/131106/predicting-with-random-effects-in-mgcv-gam/131116#131116
# https://stats.stackexchange.com/questions/189384/predicting-mean-smooth-in-gam-with-smooth-by-random-factor-interaction
#-----------------------------

library(mgcv)
pgamfits <- foreach(ab=levels(dlp$antigenf),.combine=rbind) %dopar% {
    pd <- filter(dlp, antigenf==ab)
    gfit <- mgcv::gam(seropos~s(age, bs="cr",by=tr)+s(childid,bs="re",by=dummy)+tr, data=pd,family="binomial")
    newd0 <- pd %>% mutate(dummy=0,tr="Cont")
    newd1 <- pd %>% mutate(dummy=0,tr="Int")
    gsci0 <- gamCI(m=gfit,newdata=newd0,nreps=1000)
    gsci1 <- gamCI(m=gfit,newdata=newd1,nreps=1000)
    gsci <- bind_rows(gsci0,gsci1) %>% mutate(antigenf=ab)
    return(gsci)
}

# use antilogit to get back to prevalence from the logit link
pgamfits <- pgamfits %>%
  mutate(fit  = 1/(1+exp(-fit)),
         uprP = 1/(1+exp(-uprP)),
         lwrP = 1/(1+exp(-lwrP)),
         uprS = 1/(1+exp(-uprS)),
         lwrS = 1/(1+exp(-lwrS))) %>%
  mutate(antigenf=factor(antigenf,levels=levels(dlp$antigenf)),
         tr=factor(tr,levels=c("Cont","Int"),labels=c("Control","Intervention"))
  )
```

## Estimate seroprevalence by treatment arm

Estimate overall mean seroprevalence by treatment arm and compare arms. Standard errors account for repeated measurements within child using the influence curve.

```
#-----------------------------
# estimate means by treatment
# arm and difference between
# arms using targeted maximum
# likelihood estimation
# get 95% CIs through the
# influence curve clustered
# on child ID
# adjust for age with GLM
#-----------------------------
tmlediffs <- foreach(ab=levels(dlp$antigenf),.combine=rbind) %dopar% {
    pd <- filter(dlp,antigenf==ab) %>% mutate(tr01=ifelse(tr=="Int",1,0))
    diffmu <- tmleAb(Y=pd$seropos,X=pd$tr01,W=pd$age,
                     id=pd$childid,SL.library=c("SL.glm"),
                     family="binomial")
    di <- data.frame(antigenf=ab,diff=diffmu$psi,lb=diffmu$lb,ub=diffmu$ub,p=diffmu$p)
    return(di)
  }

#-----------------------------
# report estimates in tables
#-----------------------------
knitr::kable(tmlediffs,digits=2,caption="Difference in seroprevalence (Intervention minus Control)")  %>%
  kable_styling(bootstrap_options = "striped",full_width = TRUE)
```

Difference in seroprevalence (Intervention minus Control)

| antigenf | diff | lb | ub | p |
| --- | --- | --- | --- | --- |
| Giardia VSP-3 or VSP-5 | 0.00 | -0.08 | 0.07 | 0.92 |
| Cryptosporidium Cp17 or Cp23 | -0.06 | -0.15 | 0.03 | 0.17 |
| E. histolytica LecA | -0.01 | -0.06 | 0.04 | 0.72 |
| Salmonella LPS groups B or D | 0.04 | -0.03 | 0.10 | 0.26 |
| ETEC LT B subunit | 0.01 | -0.05 | 0.07 | 0.68 |
| Cholera toxin B subunit | 0.00 | -0.08 | 0.07 | 0.89 |
| Campylobacter p18 or p39 | -0.01 | -0.07 | 0.04 | 0.62 |

No evidence for lower enteric antibody seroprevalence due to intervention.

## Figure of seroprevlance curves

```
# bright color-blind palette defined in an earlier code chunk
pcols <- c(cblack,cteal)

ppcurve <- ggplot(data=pgamfits,aes(x=age,color=tr,fill=tr)) +
  # approximate simultaneous CI from spline fit
  geom_ribbon(aes(ymin=lwrS,ymax=uprS),alpha=0.2,color=NA) +
  # spline fit
  geom_line(aes(y=fit)) +
  # plot aesthetics
  facet_wrap(~antigenf,nrow=7,ncol=2) +
  scale_x_continuous(limits=c(4,17),breaks=seq(4,16,by=2))+
  scale_y_continuous(breaks=seq(0,1,by=0.2),labels=sprintf("%1.0f",seq(0,1,by=0.2)*100))+
  coord_cartesian(ylim=c(0,1))+
  labs(x="Age in months",y="Seroprevalence (%)") +
  scale_fill_manual(values=pcols) +
  scale_color_manual(values=pcols) +
  theme_minimal() +
  theme(legend.position = "right")

ppcurve
```

```
## Warning: Removed 2 rows containing missing values (geom_path).
```

# Kongwa, Tanzania Intervention Effects

In Kongwa, Tanzania, antibody measurements were among children ages 1-9 years old. Without children <1, it was difficult to identify seropositivity cutoff values based on mixture models for any of the enteric pathogens (see main text). Therefore, there were only 3 pathogens (Giardia, Crypto, E. histolytica) where we could classify children as seropositive or seronegative becuase out-of-sample specimens were used to estimate cutoffs using an ROC curve. In light of this limitation, below we compare the intervention and control groups using mean log MFI values, which is a reasonable alternative in this situation (Arnold et al. 2017.

*Salmonella* and *Campylobacter* antibodies were not measured after the first year enrollment survey in 2012. No clusters were treated at enrollment, therefore we cannot assess the effect of azithromycin treatment on these pathogens. Therefore, limit comparisons to years 2013-2015 to avoid any potential time trends, including those due to a change in bead lots from 2012 until the later years.

```
#-----------------------------
# load the formatted data
# for enteric pathogens
# with seropositivity indicators
#-----------------------------
dl <- readRDS(here::here("data","kongwa_analysis2.rds"))

dl <- dl %>%
  filter(!antigen %in% c("salb","sald","p18","p39","pgp3")) %>%
  filter(!year %in% c(2012)) %>%
  mutate(antigenf=droplevels(antigenf),
         pathogen=droplevels(pathogen),
         tr=factor(ifelse(aztr==0,"Control","Intervention"),levels=c("Control","Intervention"))
  )
```

## Estimate curves in each treatment arm

```
#-----------------------------
# fit cubic spline over age
# estimate approximate simultaneous confidence intervals
# for the fits
# include random effects for
# cluster to account for repeated
# observations
#-----------------------------
gamfits <- foreach(ab=levels(dl$antigenf),.combine=rbind) %dopar% {
    pd <- filter(dl, antigenf==ab) %>%
      mutate(dummy=1)
    gfit <- mgcv::gam(logmfi~s(age, bs="cr",k=9,by=tr)+s(clusterid,bs="re",by=dummy)+tr ,data=pd,family="gaussian")
    newd0 <- pd %>% mutate(dummy=0,tr="Control")
    newd1 <- pd %>% mutate(dummy=0,tr="Intervention")
    gsci0 <- gamCI(m=gfit,newdata=newd0,nreps=1000)
    gsci1 <- gamCI(m=gfit,newdata=newd1,nreps=1000)
    gsci <- bind_rows(gsci0,gsci1) %>% mutate(antigenf=ab)
    return(gsci)
  }

gamfits <- gamfits %>%
  mutate(antigenf=factor(antigenf,levels=levels(dl$antigenf)),
         tr=factor(tr,levels=levels(dl$tr))
  )
```

## Estimate means by treatment arm

Estimate overall mean log MFI by treatment arm and compare arms. Standard errors account for repeated measurements within clusters using the influence curve.

```
#-----------------------------
# estimate means by treatment
# arm and difference between
# arms using targeted maximum
# likelihood estimation
# get 95% CIs through the
# influence curve clustered
# on child ID
# adjust for age with glm
#-----------------------------
ktmlemeans <- foreach(tri=levels(dl$tr),.combine=rbind) %:% 
  foreach(ab=levels(dl$antigenf),.combine=rbind) %dopar% {
    pd <- filter(dl,tr==tri & antigenf==ab)
    mu <- tmleAb(Y=pd$logmfi,id=pd$clusterid,W=select(pd,age),
                 SL.library=c("SL.glm"))
    di <- data.frame(tr=tri,antigenf=ab,mean=mu$psi,se=mu$se,lb=mu$lb,ub=mu$ub)
    return(di)
  }

ktmlediffs <- foreach(ab=levels(dl$antigenf),.combine=rbind) %dopar% {
    pd <- filter(dl,antigenf==ab) %>% mutate(tr01=ifelse(tr=="Control",0,1))
    diffmu <- tmleAb(Y=pd$logmfi,X=pd$tr01,W=select(pd,age),
                     id=pd$clusterid,SL.library=c("SL.glm"))
    di <- data.frame(antigenf=ab,diff=diffmu$psi,se=diffmu$se,lb=diffmu$lb,ub=diffmu$ub,p=diffmu$p)
    return(di)
  }

#-----------------------------
# report estimates in tables
#-----------------------------

# seroprevalence among intervention and control
knitr::kable(arrange(ktmlemeans,antigenf,tr),digits=2,caption="Mean MFI in the Intervention and Control groups")  %>%
  kable_styling(bootstrap_options = "striped",full_width = TRUE)
```

Mean MFI in the Intervention and Control groups

| tr | antigenf | mean | se | lb | ub |
| --- | --- | --- | --- | --- | --- |
| Control | Giardia VSP-3 | 2.55 | 0.04 | 2.48 | 2.62 |
| Intervention | Giardia VSP-3 | 2.62 | 0.02 | 2.58 | 2.67 |
| Control | Giardia VSP-5 | 2.64 | 0.04 | 2.56 | 2.71 |
| Intervention | Giardia VSP-5 | 2.71 | 0.02 | 2.66 | 2.75 |
| Control | Cryptosporidium Cp17 | 3.20 | 0.04 | 3.12 | 3.27 |
| Intervention | Cryptosporidium Cp17 | 3.22 | 0.02 | 3.17 | 3.27 |
| Control | Cryptosporidium Cp23 | 3.36 | 0.03 | 3.29 | 3.42 |
| Intervention | Cryptosporidium Cp23 | 3.40 | 0.02 | 3.35 | 3.44 |
| Control | E. histolytica LecA | 2.08 | 0.06 | 1.95 | 2.20 |
| Intervention | E. histolytica LecA | 2.32 | 0.04 | 2.24 | 2.40 |
| Control | ETEC LT B subunit | 4.30 | 0.02 | 4.27 | 4.33 |
| Intervention | ETEC LT B subunit | 4.33 | 0.01 | 4.31 | 4.35 |
| Control | Cholera toxin B subunit | 3.99 | 0.03 | 3.94 | 4.04 |
| Intervention | Cholera toxin B subunit | 4.01 | 0.02 | 3.98 | 4.05 |

```
# difference between groups
knitr::kable(ktmlediffs,digits=2,caption="Difference in MFI (Intervention minus Control)")  %>%
  kable_styling(bootstrap_options = "striped",full_width = TRUE)
```

Difference in MFI (Intervention minus Control)

| antigenf | diff | se | lb | ub | p |
| --- | --- | --- | --- | --- | --- |
| Giardia VSP-3 | 0.07 | 0.04 | -0.02 | 0.15 | 0.14 |
| Giardia VSP-5 | 0.06 | 0.05 | -0.03 | 0.15 | 0.19 |
| Cryptosporidium Cp17 | 0.02 | 0.04 | -0.07 | 0.11 | 0.63 |
| Cryptosporidium Cp23 | 0.04 | 0.04 | -0.04 | 0.12 | 0.32 |
| E. histolytica LecA | 0.25 | 0.08 | 0.09 | 0.41 | 0.00 |
| ETEC LT B subunit | 0.02 | 0.02 | -0.01 | 0.06 | 0.23 |
| Cholera toxin B subunit | 0.02 | 0.03 | -0.04 | 0.09 | 0.51 |

These results suggest that IgG values for *E. histolytica* LecA were slightly *higher* in intervention clusters than control clusters (difference = 0.25, 95% CI = 0.09, 0.42). This is consistent with age dependent mean curves (below) that show the intervention clusters that received annual azithromycin distribution consistently had slightly higher mean LecA levels.

Below, estimate the difference between arms in LecA seroprevalence, which suggests a higher seroprevalence in the intervention clusters that received annual azythromycin distribution (21% relative increase).

```
leca_serop <- foreach(tri=levels(dl$tr),.combine=rbind) %dopar% {
    pd <- filter(dl,tr==tri & antigenf=="E. histolytica LecA")
    mu <- tmleAb(Y=pd$posroc,id=pd$clusterid,W=select(pd,age),
                 SL.library=c("SL.glm"),family="binomial")
    di <- data.frame(tr=tri,antigenf="E. histolytica LecA", mean=mu$psi,se=mu$se,lb=mu$lb,ub=mu$ub)
    return(di)
}


pd <- dl %>%
  filter(antigenf=="E. histolytica LecA") %>%
  mutate(tr01=ifelse(tr=="Control",0,1))
leca_diff <- tmleAb(Y=pd$posroc,X=pd$tr01,W=select(pd,age),
                     id=pd$clusterid,SL.library=c("SL.glm"),
                    family="binomial")
```

```
##  Additive Effect
##    Parameter Estimate:  0.10546
##    Estimated Variance:  0.0017945
##               p-value:  0.012793
##     95% Conf Interval: (0.02243, 0.18849) 
## 
##  Additive Effect among the Treated
##    Parameter Estimate:  0.1055
##    Estimated Variance:  0.0017947
##               p-value:  0.012766
##     95% Conf Interval: (0.022463, 0.18853) 
## 
##  Additive Effect among the Controls
##    Parameter Estimate:  0.10568
##    Estimated Variance:  0.0017941
##               p-value:  0.012599
##     95% Conf Interval: (0.022657, 0.18869) 
## 
##  Relative Risk
##    Parameter Estimate:  1.2091
##               p-value:  0.020537
##     95% Conf Interval: (1.0296, 1.4199) 
## 
##               log(RR):  0.18988
##     variance(log(RR)):  0.0067198 
## 
##  Odds Ratio
##    Parameter Estimate:  1.5359
##               p-value:  0.011999
##     95% Conf Interval: (1.0989, 2.1466) 
## 
##               log(OR):  0.42911
##     variance(log(OR)):  0.029176
```

## Figure of mean curves

```
# bright color-blind palette defined in an earlier code chunk
pcols <- c(cblack,cteal)

mucurve <- ggplot(data=gamfits,aes(x=age,color=tr,fill=tr)) +
  # approximate simultaneous CI from spline fit
  geom_ribbon(aes(ymin=lwrS,ymax=uprS),alpha=0.2,color=NA) +
  # spline fit
  geom_line(aes(y=fit)) +
  # plot aesthetics
  facet_wrap(~antigenf,nrow=7,ncol=2) +
  scale_x_continuous(limits=c(1,9),breaks=1:9)+
  scale_y_continuous(breaks=seq(0,4,by=1))+
  coord_cartesian(ylim=c(0,4.5))+
  labs(x="Age in years",y="log10 Luminex Response (MFI-bg)") +
  scale_fill_manual(values=pcols) +
  scale_color_manual(values=pcols) +
  theme_minimal() +
  theme(legend.position = "right")

mucurve
```

# Kongwa, Tanzania Bead Lot Effects

## Load and format data

```
#-----------------------------
# load the formatted data
# created with 
# asembo-enteric-ab-data-format.Rmd
#-----------------------------
dl <- readRDS(here::here("data","kongwa_analysis.rds"))

# list the enteric antigens and formatted labels for them
mbavars <- c("vsp3","vsp5","cp17","cp23","leca","salb","sald","etec","cholera","p18","p39","pgp3")

mbalabs <- c("Giardia VSP-3","Giardia VSP-5","Cryptosporidium Cp17","Cryptosporidium Cp23","E. histolytica LecA","Salmonella LPS group B","Salmonella LPS group D","ETEC LT B subunit","Cholera toxin B subunit","Campylobacter p18","Campylobacter p39","C. trachomatis pgp3")

dl <- dl %>%
  mutate(antigen=as.character(antigen))

#-----------------------------
# subset to antigens measured
# in all years
#-----------------------------
dl <- dl %>%
  filter(antigen %in% c("vsp3","vsp5","cp17","cp23","leca","etec"))

#-----------------------------
# create a stratification
# variable for bead set
#-----------------------------
dl <- dl %>%
  mutate(beadset = factor(ifelse(year==2012,"Year 1","Years 2-4")),
         antigenf=factor(antigenf))
```

```
#----------------------------
# Add ROC cutoffs 
# Estimated by Diana Martin
# for years 1 and 2-4 for antigens
# included in multiple years
#----------------------------
dl$roccut <- NA
dl$roccut[dl$antigen %in% "vsp3"] <- log10(116) 
dl$roccut[dl$antigen %in% "vsp5"] <- log10(109) 
dl$roccut[dl$antigen %in% "cp23"] <- log10(1185)
dl$roccut[dl$antigen %in% "cp17"] <- log10(321)
dl$roccut[dl$antigen %in% "leca"] <- log10(153)
dl$roccut[dl$antigen %in% "pgp3"] <- log10(818)


# different cutoffs for bead set used in years 2-4
dl$roccut[dl$antigen %in% "vsp3"  & dl$svy >1] <- log10(170)
dl$roccut[dl$antigen %in% "vsp5"  & dl$svy >1] <- log10(141)
dl$roccut[dl$antigen %in% "cp23"  & dl$svy >1] <- log10(378) 
dl$roccut[dl$antigen %in% "cp17"  & dl$svy >1] <- log10(180) 
dl$roccut[dl$antigen %in% "leca"  & dl$svy >1] <- log10(94)
dl$roccut[dl$antigen %in% "pgp3"  & dl$svy >1] <- log10(882)
```

## Age stratified antibody distributions

This is a beadset-stratified version of Figure 1 - supplement 1 in the article.

```
# create age categories
dlplot <- dl %>%
  mutate(agecat = cut(age,breaks=c(0,1,2,3,4,10),
                      labels=c("1 year","2 years","3 years","4 years","5-9 years")),
         antigenf=factor(antigenf,levels=mbalabs)
         ) %>%
  filter(antigen!="pgp3") %>% mutate(antigenf=droplevels(antigenf)) %>%
  group_by(antigen) %>%
  mutate(plotroc=min(roccut))


# custom color blind color palette is in the preamble chunck
pcols <- c(cteal,corange)


# custom log labels
log10labs <- c( 
  expression(10^0),
  expression(10^1),
  expression(10^2),
  expression(10^3),
  expression(10^4)
)

# faceted over age and antigen
p2 <- ggplot(data=dlplot,aes(x=logmfi,group=beadset,fill=beadset,color=beadset)) +
  facet_grid(agecat~antigenf,scales='free')+
  geom_density(aes(y=..density..),color=NA,alpha=0.5)+
  geom_vline(aes(xintercept = roccut,color=beadset)) +
  coord_cartesian(ylim=c(0, 1.25))+
  scale_x_continuous(expand=c(0,0),limits=c(0,5),breaks=0:4,labels=log10labs) +
  scale_fill_manual(values=pcols)+
  scale_color_manual(values=pcols)+
  labs(title="",x="Luminex Response (MFI-bg)",y="Density") +
  theme_minimal()+
  theme(
    strip.text.x = element_text(size=9),
    strip.text.y=element_text(size=12,angle=0),
    legend.position="top"
  )

p2
```

```
## Warning: Removed 4989 rows containing missing values (geom_vline).
```

The patterns are very similar across beadsets. Vertical lines are ROC-cutoffs for each beadset.

Below is a figure that contrasts the distribution of the Year 1 beadset (blue) with the overall distribution (black line); and then Years 2-4 beadset (orange) with the overall distribution (black line). The plots show that the combined analysis, which pools data over all years, essentially reflects the patterns observed in years 2-4 – consistent with that bead set contributing >4 times more observations to the analysis (n=4,087 vs n=902).

```
# faceted over age and antigen
p3 <- ggplot(data=dlplot,aes(x=logmfi)) +
  facet_grid(agecat~antigenf,scales='free')+
  geom_density(data=filter(dlplot,beadset=="Year 1"), aes(y=..density..),fill=cteal,color=NA,alpha=0.5)+
  geom_density(aes(y=..density..),fill=NA,color="black")+
  # scale_y_continuous(expand=c(0,0),breaks=seq(0,40,by=10)) +
  coord_cartesian(ylim=c(0, 1.25))+
  scale_x_continuous(expand=c(0,0),limits=c(0,5),breaks=0:4,labels=log10labs) +

  labs(title="",x="Luminex Response (MFI-bg)",y="Density") +
  theme_minimal()+
  theme(
    strip.text.x = element_text(size=9),
    strip.text.y=element_text(size=12,angle=0),
    legend.position="top"
  )

p3
```

```
p4 <- ggplot(data=dlplot,aes(x=logmfi)) +
  facet_grid(agecat~antigenf,scales='free')+
  geom_density(data=filter(dlplot,beadset=="Years 2-4"), aes(y=..density..),fill=corange,color=NA,alpha=0.5)+
  geom_density(aes(y=..density..),fill=NA,color="black")+
  coord_cartesian(ylim=c(0, 1.25))+
  scale_x_continuous(expand=c(0,0),limits=c(0,5),breaks=0:4,labels=log10labs) +

  labs(title="",x="Luminex Response (MFI-bg)",y="Density") +
  theme_minimal()+
  theme(
    strip.text.x = element_text(size=9),
    strip.text.y=element_text(size=12,angle=0),
    legend.position="top"
  )

p4
```

## Joint distribution of antibody response

Summarize the joint distribution of antibody response, stratified by beadsets used in Y1 and Y2-4 for antigens included in both beadsets.

```
#--------------------------------
# create antigen groupings for
# comparisons
# drop obs not contributing
#--------------------------------
dl2 <- dl %>%
  mutate(comp= ifelse(antigen %in% c("vsp3","vsp5"),"Giardia",NA),
         comp= ifelse(antigen %in% c("cp17","cp23"),"Cryptosporidium",comp),
         comp= factor(comp,levels=c("Giardia","Cryptosporidium"))
         ) %>% 
  mutate(xlab=ifelse(antigen %in% c("vsp3","vsp5"),"VSP-3",NA),
         ylab=ifelse(antigen %in% c("vsp3","vsp5"),"VSP-5",NA),
         xlab=ifelse(antigen %in% c("cp17","cp23"),"Cp17",xlab),
         ylab=ifelse(antigen %in% c("cp17","cp23"),"Cp23",ylab)
         ) %>%
  filter(!is.na(comp))

#--------------------------------
# for antigen each pair, 
# label it as "x" or "y" to 
# spread it to wide format
#--------------------------------
dw <- dl2 %>%
  mutate(xy=ifelse(antigen %in% c("vsp3","cp17"),"x","y")) %>%
  select(beadset,comp,xlab,ylab,id,xy,logmfi) %>%
  spread(xy,logmfi) %>%
  mutate(beadset=factor(beadset,levels=c("Year 1","Years 2-4")))
```

```
#--------------------------------
# estimate spearman's correlation
# within each country, comparison
#--------------------------------
dcorr <- dw %>%
  group_by(beadset,comp) %>%
  mutate(corxy=cor(x,y,method="spearman",use="pairwise.complete.obs") ) %>%
  summarize(corxy=max(corxy,na.rm=T))
```

```
#--------------------------------
# estimate smooths, trimmed to
# drop the bottom and top 1% of
# data in each comparison
# to avoid edge effects
#--------------------------------
dsmooths <- foreach(beadseti=levels(dw$beadset),.combine=rbind) %:%
                      foreach(compi=levels(dw$comp),.combine=rbind) %do% {
                        pd <- filter(dw,beadset==beadseti & comp==compi)
                        if(nrow(pd)>0) {
                          xqs <- quantile(pd$x,probs=c(0.01,0.99),na.rm=TRUE)
                          newd <- data.frame(x=seq(xqs[1],xqs[2],by=0.01))
                          lfit <- loess(y~x,data=pd)
                          return(data.frame(beadset=beadseti,comp=compi,x=newd,y=predict(lfit,newdata=newd)))
                        }
                        
  
}
```

```
pcols <- c(cteal,corange)

# grab labels
dlabs <- dw %>% select(beadset,comp,xlab,ylab) %>% group_by(beadset,comp) %>% slice(1)

complot <- ggplot(data=dw,aes(x=x,y=y,color=beadset)) +
  facet_grid(comp~beadset) +
  geom_point(pch=19,alpha=0.1) +
  geom_line(data=dsmooths,aes(x=x,y=y),col="black",size=1.2)+
  geom_text(data=dcorr,
            aes(x=0.5,y=4.3,label=paste("rho ==",sprintf("%1.2f",corxy)) ),
            parse=TRUE, col="black")   +
  geom_text(data=dlabs,aes(x=2.3,y=0.1,label=xlab),color="gray40",angle=0)+
  geom_text(data=dlabs,aes(x=0.1,y=2.3,label=ylab),color="gray40",angle=90)+
  scale_x_continuous(limits=c(0,4.6),breaks=0:4,labels = log10labs)+
  scale_y_continuous(limits=c(0,4.6),breaks=0:4,labels = log10labs)+
  scale_color_manual(values=pcols) +
  coord_equal() +
  labs(x="Luminex Response (MFI-bg)",y="Luminex Response (MFI-bg)") +
  theme_minimal(base_size=12) +
  theme(
    strip.text.x=element_text(size=12),
    strip.text.y=element_text(size=12,angle=0),
    legend.position="none"
  )


complot
```

Stratified version of Figure 2 in the main text. The relationships and correlation estimates are nearly identical across beadsets.

## Age-dependent mean curves

Estimate age-dependent mean log10 MFI-bg in the Tanzania study, separately for the two different bead sets.

```
# ---------------------------------------
# fit cubic spline over age, separately for each antigen
# and beadset
# estimate approximate simultaneous confidence intervals
# for the fits
# ---------------------------------------
library(mgcv)
gamfits <- foreach(ab=levels(dl$antigenf),.combine=rbind) %:%
  foreach(beadseti=levels(dl$beadset),.combine=rbind) %dopar% {
    pd <- dplyr::filter(dl, antigenf==ab & beadset==beadseti)
    gfit <- gam(logmfi~s(age, bs="cr",k=9),data=pd)
    gsci <- gamCI(m=gfit,newdata=pd,nreps=1000)
    gsci$antigen <- ab
    return(gsci)
  }

# ---------------------------------------
# get overall, pooled estimate
# ---------------------------------------
gamfits2 <- foreach(ab=levels(dl$antigenf),.combine=rbind) %dopar% {
    pd <- dplyr::filter(dl, antigenf==ab)
    gfit <- gam(logmfi~s(age, bs="cr",k=9),data=pd)
    gsci <- gamCI(m=gfit,newdata=pd,nreps=1000)
    gsci$antigen <- ab
    gsci$beadset <- factor("Combined",levels=c("Year 1","Years 2-4","Combined"))
    return(gsci)
}

gamfits <- gamfits %>%
  mutate(beadset=factor(beadset,levels=c("Year 1","Years 2-4","Combined"))) %>%
  bind_rows(gamfits2)
```

```
log10labs <- c( 
  expression(10^0),
  expression(10^1),
  expression(10^2),
  expression(10^3),
  expression(10^4)
)


dplot <- data.frame(gamfits) %>%
  dplyr::filter(pathogen!="V. cholerae") %>%
  mutate(pathogen=factor(pathogen,levels=c("Giardia",
                                           "Cryptosporidium",
                                           "E. histolytica",
                                           "ETEC")),
         antigenf=factor(antigenf,levels=c("Giardia VSP-3","Giardia VSP-5",
                                           "Cryptosporidium Cp17","Cryptosporidium Cp23",
                                           "E. histolytica LecA",
                                           "ETEC LT B subunit")))

pcols <- c(cteal,corange,cblack)

pcurve <- ggplot(data=dplot,aes(x=age+0.5,group=beadset,color=beadset,fill=beadset)) +
  # approximate simultaneous CI from spline fit
  geom_ribbon(aes(ymin=lwrS,ymax=uprS),alpha=0.2,color=NA) +
  # spline fit
  geom_line(aes(y=fit)) +
  # plot aesthetics
  facet_wrap(~antigenf,nrow=6,ncol=2) +
  scale_x_continuous(limits=c(0,10),breaks=seq(0,10,by=2))+
  scale_y_continuous(breaks=0:4,labels=log10labs)+
  coord_cartesian(ylim=c(0,4.5))+
  labs(x="Age in years",y="Luminex Response (MFI-bg)") +
  scale_fill_manual(values=pcols) +
  scale_color_manual(values=pcols) +
  theme_minimal(base_size=16) +
  theme(legend.position = "top")

pcurve
```

The results show that in general the MFI-bg levels were slightly lower in year 1 than in years 2-4, but that the qualitative age-dependent patterns are similar. Since year is perfectly confounded by bead lot, there is no way to distinguish whether differences are due to between-year differences in exposure or due to bead lot effects. The plots also show that the combined analysis, which pools data over all years, essentially reflects the patterns observed in years 2-4 – consistent with that bead set contributing >4 times more observations to the analysis (n=4,087 vs n=902).

# Leogane, Haiti seasonal effects

Of the three studies included in this analysis, Haiti was the only cohort that spanned wet and rainy seasons. Following seasonsal definitions for Haiti described by Moss et al. 2014, we examined the effect of season on IgG levels and seroprevalence. Moss et al. (2014) previously examined *Giardia*, *Cryptosporidium*, and *E. histolytica* IgG responses by season, and found evidence for higher mean levels of VSP and LecA antibody responses among measurements collected during the wet season. Below, we expanded the analysis to include all pathogens included in this study, and examined both IgG levels and seroprevalence.

In Haiti, there are two rainy seasons: from April - June and August - October. We classified measurements collected in these months as wet season measurements, and all others as dry season measurements. We then compared distributions of IgG, mean IgG levels, and seroprevalence between the two seasons. We also compared age-dependent mean IgG levels stratified by season to examine whether the curves suggested evidence for a shift, as has been demonstrated for malaria in a very high transmission setting (Arnold et al. 2017). We found some evidence for slightly higher IgG levels and seroprevalence, but differences were small and there was no evidence for dramatic shifts in the age-dependent mean curves.

Moss, D. M., Priest, J. W., Hamlin, K., Derado, G., Herbein, J., Petri, W. A., Jr & Lammie, P. J. Longitudinal evaluation of enteric protozoa in Haitian children by stool exam and multiplex serologic assay. Am. J. Trop. Med. Hyg. 90, 653–660 (2014).

Arnold, B. F., van der Laan, M. J., Hubbard, A. E., Steel, C., Kubofcik, J., Hamlin, K. L., Moss, D. M., Nutman, T. B., Priest, J. W. & Lammie, P. J. Measuring changes in transmission of neglected tropical diseases, malaria, and enteric pathogens from quantitative antibody levels. PLoS Negl. Trop. Dis. 11, e0005616 (2017).

## Load the data and format

```
#-----------------------------
# load the formatted data
# with seropositivity indicators
# created with:
# haiti-enteric-ab-data-format.Rmd -->
# Fig1-haiti-enteric-ab-distributions.Rmd
#-----------------------------
dl <- readRDS(here::here("data","haiti_analysis2.rds"))

# list the enteric antigens and make formatted labels for them
mbavars <- c("vsp3","vsp5","cp17","cp23","leca","salb","sald","p18","p39","etec","cholera")

mbalabs <- c("Giardia VSP-3","Giardia VSP-5","Cryptosporidium Cp17","Cryptosporidium Cp23","E. histolytica LecA","Salmonella LPS B","Salmonella LPS D","Campylobacter p18","Campylobacter p39","ETEC LT B subunit","Cholera toxin β subunit")

#-----------------------------
# Following the analysis in
# Moss et al. 2014, there are
# generally two annual rainy
# seasons in Haiti:
# April - June, August - October
# Moss, D. M., et al. Am. J. Trop. Med. Hyg. 90, 653–660 (2014).
#-----------------------------
dl <- dl %>%
  mutate(season=ifelse(month %in% c(4,5,6,8,9,10),"wet season","dry season"),
         season=factor(season,levels=c("dry season","wet season"))
)
```

## Age and MFI distributions by season

Examine distribution of measurement ages by season to confirm there is not significant imbalance

```
#-----------------------------
# distribution of measurement
# ages by season
#-----------------------------
dplot_age <- dl %>% filter(pathogen=="ETEC")

page <- ggplot(data=dplot_age,aes(x=age,stat(density),color=season,fill=season)) +
  geom_density(alpha=0.4)+
  scale_fill_manual(values=cbPalette[c(2,6)])+
  scale_color_manual(values=cbPalette[c(2,6)])
page
```

```
page2 <- ggplot(data=dplot_age,aes(x=age,stat(density),color=season,fill=season)) +
  geom_histogram(alpha=0.4,bins=30)+
  scale_fill_manual(values=cbPalette[c(2,6)])+
  scale_color_manual(values=cbPalette[c(2,6)])
page2
```

Examine MFI by antibody and season

```
dlplot <- dl %>% 
  mutate(season=factor(ifelse(season=="dry season","dry","wet"),levels=c("dry","wet")))

log10labs <- c( 
  expression(10^0),
  expression(10^1),
  expression(10^2),
  expression(10^3),
  expression(10^4)
)

pcols <- cbPalette[c(2:4,6:8)]

pbox_season <- ggplot(data=dlplot,aes(x=season,y=logmfi,color=pathogen,fill=pathogen))+
  facet_grid(~antigenf)+
  geom_jitter(height=0,width=0.2,size=0.4,alpha=0.5) +
  geom_boxplot(outlier.shape=NA,color="gray30",fill=NA)+
  scale_y_continuous(breaks=0:4,labels=log10labs)+
  scale_color_manual(values=pcols)+
  scale_fill_manual(values=pcols)+
  coord_cartesian(ylim=c(0,4.5))+
  labs(y="Luminex response (MFI-bg)")+
  theme(
    legend.position="none",
    strip.text=element_text(size=6)
  )

pbox_season
```

The boxplots suggest a slight shift in IgG to higher levels for many pathogens, including Giardia, Cryptosporidium, Salmonella, and norovirus. Below, we examine differences in mean MFI and seroprevalence, adjusting for age and accounting for repeated measures among children.

## MFI by season, adjusted for age

```
#-----------------------------
# estimate means by season 
# and difference between
# seasons using targeted maximum
# likelihood estimation
# get 95% CIs through the
# influence curve clustered
# on child ID
# adjust for age with a GAM
#-----------------------------
mfi_tmlemeans <- foreach(seasi=levels(dl$season),.combine=rbind) %:% 
  foreach(ab=levels(dl$antigenf),.combine=rbind) %dopar% {
    pd <- filter(dl,season==seasi & antigenf==ab)
    mu <- tmleAb(Y=pd$logmfi,id=pd$id,W=pd$age,
                 SL.library=c("SL.gam")
                 )
    di <- data.frame(season=seasi,antigenf=ab,mean=mu$psi,se=mu$se,lb=mu$lb,ub=mu$ub)
    return(di)
  }

mfi_tmlediffs <- foreach(ab=levels(dl$antigenf),.combine=rbind) %dopar% {
    pd <- filter(dl,antigenf==ab) %>% mutate(wet=ifelse(season=="wet season",1,0))
    diffmu <- tmleAb(Y=pd$logmfi,X=pd$wet,W=pd$age,
                     id=pd$id,SL.library=c("SL.gam")
                     )
    di <- data.frame(antigenf=ab,diff=diffmu$psi,lb=diffmu$lb,ub=diffmu$ub,p=diffmu$p)
    return(di)
  }

# bring back pathogen info for ploting aesthetics
haiti_paths <- dl %>%
  ungroup() %>%
  group_by(antigenf) %>%
  select(antigenf,pathogen) %>%
  slice(1)

mfi_tmlemeans <- mfi_tmlemeans %>% left_join(haiti_paths,by="antigenf") %>%
  mutate(season=ifelse(season=="dry season","dry","wet"))
mfi_tmlediffs <- mfi_tmlediffs %>% left_join(haiti_paths,by="antigenf")
```

```
pcols <- cbPalette[c(2:4,6:8)]

log10labs <- c( 
  expression(10^1),
  expression(10^2),
  expression(10^3),
  expression(10^4)
)

p_mfi_season <- ggplot(data=mfi_tmlemeans,aes(x=season,y=mean,color=pathogen,fill=pathogen))+
  facet_grid(.~antigenf)+
  geom_errorbar(aes(ymin=lb,ymax=ub),width=0.1)+
  geom_point()+
  labs(y="adjusted mean Luminex response (MFI-bg)")+
  scale_color_manual(values=pcols)+
  scale_fill_manual(values=pcols)+
  scale_y_continuous(breaks=1:4,labels=log10labs)+
  coord_cartesian(ylim=c(1,4.5))+
  theme(
    legend.position="none",
    strip.text=element_text(size=6)
  )

p_mfi_season
```

```
pcols <- cbPalette[c(2:4,6:8)]

p_mfi_diff_season <- ggplot(data=mfi_tmlediffs,aes(x=antigenf,y=diff,color=pathogen,fill=pathogen))+
  geom_errorbar(aes(ymin=lb,ymax=ub),width=0.1)+
  geom_point()+
  geom_hline(yintercept=0,linetype="dashed")+
  labs(y="increase in response during wet season (log10 MFI-bg)",x="Antibody")+
  scale_color_manual(values=pcols)+
  scale_fill_manual(values=pcols)+
  # scale_y_continuous(breaks=1:4,labels=log10labs)+
  # coord_cartesian(ylim=c(1,4.5))+
  theme(
    legend.position="none",
    axis.text.x=element_text(angle=45,hjust=1)
  )

p_mfi_diff_season
```

This analysis shows that mean MFI is higher across antibodies during the wet season compared to the dry season, even after adjusting for age. However, the magnitude of the differences is small and on the order of 0.1 to 0.2 (log10). Below, we compared seroprevalence by season.

## Seroprevalence by season

```
#----------------------------------
# create seropositivity indicators 
# combining information over antigens
#----------------------------------
dlp <- dl %>%
  mutate(pathogen2 = case_when(
    antigenf == "Norovirus GI.4" ~ "Norovirus GI.4",
    antigenf == "Norovirus GII.4.NO" ~ "Norovirus GII.4.NO",
    TRUE ~ as.character(pathogen)),
    pathogen2=factor(pathogen2,levels=c("Giardia","Cryptosporidium","E. histolytica","Salmonella","ETEC","Norovirus GI.4","Norovirus GII.4.NO"))
  ) %>%
  mutate(seropos=ifelse(!is.na(posroc),posroc,posmix),
         serocut=ifelse(!is.na(posroc),log10(roccut),mixcut),
         serocut_desc=ifelse(!is.na(posroc),"ROC","Mixture Model")) %>%
  mutate(seropos=ifelse(!is.na(seropos),seropos,posunex),
         serocut_desc=ifelse(!is.na(serocut),serocut_desc,"Unexp dist"),
         serocut=ifelse(!is.na(serocut),serocut,unexpcut)) %>%
  ungroup() %>% 
  group_by(pathogen,pathogen2,id,sampleid,agecat,age,season) %>%
  summarize(seropos=max(seropos))

dlp <- dlp %>%
  ungroup() %>%
  mutate(id=as.character(id))

#-----------------------------
# estimate seroprevalence by season 
# and difference between
# seasons using targeted maximum
# likelihood estimation
# also estimate the risk ratio
# get 95% CIs through the
# influence curve clustered
# on child ID
# adjust for age with a GAM
#-----------------------------
serop_tmlemeans <- foreach(seasi=levels(dlp$season),.combine=rbind) %:% 
  foreach(ab=levels(dlp$pathogen2),.combine=rbind) %dopar% {
    pd <- filter(dlp,season==seasi & pathogen2==ab)
    mu <- tmleAb(Y=pd$seropos,id=pd$id,W=pd$age,
                 SL.library=c("SL.gam"),
                 family="binomial")
    di <- data.frame(season=seasi,pathogen2=ab,mean=mu$psi,se=mu$se,lb=mu$lb,ub=mu$ub)
    return(di)
  }

serop_tmlediffs <- foreach(ab=levels(dlp$pathogen2),.combine=rbind) %dopar% {
    pd <- filter(dlp,pathogen2==ab) %>% mutate(wet=ifelse(season=="wet season",1,0))
    diffmu <- tmleAb(Y=pd$seropos,X=pd$wet,W=pd$age,
                     id=pd$id,SL.library=c("SL.gam"),
                     family="binomial")
    di <- data.frame(pathogen2=ab,diff=diffmu$psi,lb=diffmu$lb,ub=diffmu$ub,p=diffmu$p,
                     rr=diffmu$tmle_fit$estimates$RR$psi,
                     rr_lb=diffmu$tmle_fit$estimates$RR$CI[1],
                     rr_ub=diffmu$tmle_fit$estimates$RR$CI[2])
    return(di)
  }

# bring back pathogen info for ploting aesthetics
haiti_paths2 <- dlp %>%
  ungroup() %>%
  group_by(pathogen2) %>%
  select(pathogen2,pathogen) %>%
  slice(1)

serop_tmlemeans <- serop_tmlemeans %>% left_join(haiti_paths2,by="pathogen2") %>%
  mutate(season=ifelse(season=="dry season","dry","wet"))
serop_tmlediffs <- serop_tmlediffs %>% left_join(haiti_paths2,by="pathogen2")
```

```
pcols <- cbPalette[c(2:4,6:8)]

p_serop_season <- ggplot(data=serop_tmlemeans,aes(x=season,y=mean,color=pathogen,fill=pathogen))+
  facet_grid(.~pathogen2)+
  geom_errorbar(aes(ymin=lb,ymax=ub),width=0.1)+
  geom_point()+
  labs(y="seroprevalence (%)")+
  scale_color_manual(values=pcols)+
  scale_fill_manual(values=pcols)+
  scale_y_continuous(breaks=seq(0,1,by=0.1),labels=sprintf("%1.0f",seq(0,1,by=0.1)*100))+
  coord_cartesian(ylim=c(0,1))+
  theme(
    legend.position="none"
  )

p_serop_season
```

```
# custom color blind color palette is in the preamble chunck
pcols <- cbPalette[c(2:4,6:8)]
serop_tmlediffs <- serop_tmlediffs %>%
  mutate(difflab = sprintf("%1.1f",diff*100))
p_serop_diff_season <- ggplot(data=serop_tmlediffs,aes(x=pathogen2,y=diff,color=pathogen,fill=pathogen))+
  geom_errorbar(aes(ymin=lb,ymax=ub),width=0.1)+
  geom_point()+
  geom_text(aes(y=diff,label=difflab),nudge_x = 0.2)+
  geom_hline(yintercept=0,linetype="dashed")+
  labs(y="increase in seroprevalence during wet season (%)",x="Pathogen")+
  scale_color_manual(values=pcols)+
  scale_fill_manual(values=pcols)+
  scale_y_continuous(breaks=seq(-0.04,0.12,by=0.02),labels=sprintf("%1.0f",seq(-0.04,0.12,by=0.02)*100))+
  coord_cartesian(ylim=c(-0.04,0.12))+
  theme(
    legend.position="none",
    axis.text.x=element_text(angle=45,hjust=1)
  )

p_serop_diff_season
```

```
# custom color blind color palette is in the preamble chunck
pcols <- cbPalette[c(2:4,6:8)]
serop_tmlediffs <- serop_tmlediffs %>%
  mutate(rrlab = sprintf("%1.2f",rr))
p_serop_rr_season <- ggplot(data=serop_tmlediffs,aes(x=pathogen2,y=rr,color=pathogen,fill=pathogen))+
  geom_errorbar(aes(ymin=rr_lb,ymax=rr_ub),width=0.1)+
  geom_point()+
  geom_text(aes(y=rr,label=rrlab),nudge_x = 0.2)+
  geom_hline(yintercept=1,linetype="dashed")+
  labs(y="wet season prevalence ratio",x="Pathogen")+
  scale_color_manual(values=pcols)+
  scale_fill_manual(values=pcols)+
  scale_y_log10(breaks=c(0.9,1,1.1,1.2))+
  coord_cartesian(ylim=c(0.9,1.2))+
  theme(
    legend.position="none",
    axis.text.x=element_text(angle=45,hjust=1)
  )

p_serop_rr_season
```

Consistent with comparisons of IgG levels, this analysis demonstrated that seroprevalence was consistently elevated during the wet season across pathogens, but the increase was small, ranging from 0.8 to 4.8 percentage points, or relative increase of between 1 and 7 percent.

## Estimate age-seroprevalence curves by season

Above, there was slight evidence for higher IgG levels and seroprevalence across pathogens during the wet season as compared to the dry season. Below, estimate age-dependent mean MFI levels using cubic splines to examine whether season changes these overall patterns by age. The results suggest differences between seasons are very slight, consistent with the results above.

```
#----------------------------------
# simulataneous CIs for GAMs
# estimated by resampling the 
# Baysian posterior estimates of
# the variance-covariance matrix
# assuming that it is multivariate normal
# the function below also estimates 
# the unconditional variance-covariance
# matrix, Vb=vcov(x,unconditional=TRUE), 
# which allows for undertainty in the actual
# estimated mean as well 
# (Marra & Wood 2012 Scandinavian Journal of Statistics, 
#  Vol. 39: 53–74, 2012, doi: 10.1111/j.1467-9469.2011.00760.x )
# simultaneous CIs provide much better coverage than pointwise CIs
# see: http://www.fromthebottomoftheheap.net/2016/12/15/simultaneous-interval-revisited/
#----------------------------------

gamCI <- function(m,newdata,nreps=10000) {
  require(mgcv)
  require(dplyr)
  Vb <- vcov(m,unconditional = TRUE)
  pred <- predict(m, newdata, se.fit = TRUE)
  fit <- pred$fit
  se.fit <- pred$se.fit
  BUdiff <- MASS::mvrnorm(n=nreps, mu = rep(0, nrow(Vb)), Sigma = Vb)
  Cg <- predict(m, newdata, type = "lpmatrix")
  simDev <- Cg %*% t(BUdiff)
  absDev <- abs(sweep(simDev, 1, se.fit, FUN = "/"))
  masd <- apply(absDev, 2L, max)
  crit <- quantile(masd, prob = 0.95, type = 8)
  pred <- data.frame(newdata,fit=pred$fit,se.fit=pred$se.fit)
  pred <- mutate(pred,
                 uprP = fit + (2 * se.fit),
                 lwrP = fit - (2 * se.fit),
                 uprS = fit + (crit * se.fit),
                 lwrS = fit - (crit * se.fit)
  )
  return(pred)
}
```

Estimate age dependent mean MFI curves by season

```
#-----------------------------
# fit cubic spline over age, separately for each 
# season and antigen
# estimate approximate simultaneous confidence intervals
# for the fits
# set the dummy to 0 to 
# zero out child-level random effects
# see posts on Stack Exchange for explanation:
# https://stats.stackexchange.com/questions/131106/predicting-with-random-effects-in-mgcv-gam/131116#131116
# https://stats.stackexchange.com/questions/189384/predicting-mean-smooth-in-gam-with-smooth-by-random-factor-interaction
#-----------------------------

#-----------------------------
# create a dummy variable equal
# to 1 for all obs as a trick to
# get marginal predictions by
# age while still including REs
# for child
#-----------------------------
dl <- dl %>%
  ungroup() %>%
  mutate(dummy=1) %>%
  mutate(id=as.character(id),
         fid=factor(id))

library(mgcv)
gamfits <- foreach(ab=levels(dl$antigenf),.combine=rbind) %dopar% {
    pd <- filter(dl, antigenf==ab)
    gfit <- mgcv::gam(logmfi~s(age, bs="cr",by=season)+s(fid,bs="re",by=dummy)+season, data=pd)
    newd0 <- pd %>% mutate(dummy=0,season="dry season")
    newd1 <- pd %>% mutate(dummy=0,season="wet season")
    gsci0 <- gamCI(m=gfit,newdata=newd0,nreps=1000)
    gsci1 <- gamCI(m=gfit,newdata=newd1,nreps=1000)
    gsci <- bind_rows(gsci0,gsci1) %>% mutate(antigenf=ab)
    return(gsci)
}

# reinstate factors
gamfits <- gamfits %>%
  mutate(antigenf=factor(antigenf,levels=levels(dl$antigenf)),
         season=factor(season,levels=c("dry season","wet season"))
  )
```

Figure of age-dependent mean MFI curves

```
# bright color-blind palette defined in an earlier code chunk
pcols <- cbPalette[c(2,6)]

log10labs <- c( 
  expression(10^0),
  expression(10^1),
  expression(10^2),
  expression(10^3),
  expression(10^4)
)

pcurve <- ggplot(data=gamfits,aes(x=age,color=season,fill=season)) +
  # approximate simultaneous CI from spline fit
  geom_ribbon(aes(ymin=lwrS,ymax=uprS),alpha=0.2,color=NA) +
  # spline fit
  geom_line(aes(y=fit)) +
  # plot aesthetics
  facet_wrap(~antigenf,nrow=5,ncol=2) +
  scale_x_continuous(breaks=seq(0,10,by=1))+
  scale_y_continuous(breaks=0:4,labels=log10labs)+
  coord_cartesian(ylim=c(0,4.5),xlim=c(0,10))+
  labs(x="age in years",y="Luminex response (MFI-bg)") +
  scale_fill_manual(values=pcols) +
  scale_color_manual(values=pcols) +
  theme_minimal() +
  theme(legend.position = "top")

pcurve
```

# Session Info

```
sessionInfo()
```

```
## R version 3.5.3 (2019-03-11)
## Platform: x86_64-apple-darwin15.6.0 (64-bit)
## Running under: macOS High Sierra 10.13.6
## 
## Matrix products: default
## BLAS: /Library/Frameworks/R.framework/Versions/3.5/Resources/lib/libRblas.0.dylib
## LAPACK: /Library/Frameworks/R.framework/Versions/3.5/Resources/lib/libRlapack.dylib
## 
## locale:
## [1] en_US.UTF-8/en_US.UTF-8/en_US.UTF-8/C/en_US.UTF-8/en_US.UTF-8
## 
## attached base packages:
## [1] parallel  stats     graphics  grDevices utils     datasets  methods  
## [8] base     
## 
## other attached packages:
##  [1] tmle_1.3.0-1             SuperLearner_2.0-25-9000
##  [3] nnls_1.4                 doParallel_1.0.11       
##  [5] iterators_1.0.9          foreach_1.4.4           
##  [7] kableExtra_0.8.0.0001    knitr_1.22              
##  [9] mgcv_1.8-27              nlme_3.1-137            
## [11] tmleAb_0.3.4             forcats_0.3.0           
## [13] stringr_1.4.0            dplyr_0.8.0.1           
## [15] purrr_0.3.2              readr_1.1.1             
## [17] tidyr_0.8.3              tibble_2.1.1            
## [19] ggplot2_3.1.1            tidyverse_1.2.1         
## [21] here_0.1                
## 
## loaded via a namespace (and not attached):
##  [1] Rcpp_1.0.1        lubridate_1.7.4   lattice_0.20-38  
##  [4] assertthat_0.2.1  rprojroot_1.3-2   digest_0.6.18    
##  [7] psych_1.8.4       R6_2.4.0          cellranger_1.1.0 
## [10] plyr_1.8.4        backports_1.1.4   evaluate_0.13    
## [13] httr_1.4.0        highr_0.8         pillar_1.4.0     
## [16] rlang_0.3.4       lazyeval_0.2.2    readxl_1.1.0     
## [19] rstudioapi_0.9.0  Matrix_1.2-15     rmarkdown_1.12   
## [22] labeling_0.3      splines_3.5.3     foreign_0.8-71   
## [25] munsell_0.5.0     broom_0.4.4       compiler_3.5.3   
## [28] modelr_0.1.2      xfun_0.6          pkgconfig_2.0.2  
## [31] mnormt_1.5-5      htmltools_0.3.6   tidyselect_0.2.5 
## [34] codetools_0.2-16  viridisLite_0.3.0 crayon_1.3.4     
## [37] withr_2.1.2       grid_3.5.3        jsonlite_1.6     
## [40] gtable_0.3.0      magrittr_1.5      scales_1.0.0     
## [43] cli_1.1.0         stringi_1.4.3     reshape2_1.4.3   
## [46] xml2_1.2.0        tools_3.5.3       glue_1.3.1       
## [49] hms_0.4.2         yaml_2.2.0        colorspace_1.3-2 
## [52] rvest_0.3.2       haven_2.1.0
```
