# Supplementary material for: Enteropathogen antibody dynamics and force of infection among children in low-resource settings
Source: eLife. 2019 Aug 19;8:e45594. doi: 10.7554/eLife.45594 (PMC6746552; doi:10.7554/eLife.45594)
Supplement: Supplementary file 2. [file elife-45594-supp2.zip › SIFile2.html]

Enteropathogen antibody dynamics and force of infection among children in low-resource settings


Code 

- Show All Code
- Hide All Code

# Enteropathogen antibody dynamics and force of infection among children in low-resource settings

### Supplementary Information File 2. Classification agreement between different seropositivity cutoff approaches.

# Summary

This notebook compares agreement between seropositivity cutoff values estimated by three different methods:

**ROC-based cutoffs using out-of-sample known positive and negative specimens**: These cutoff values are available only for a select group of antigens where confirmed positive and negative specimens existed.

**Finite Gaussian mixture models**: Estimated by fitting a 2-component, finite Gaussian mixture model to the antibody distributions among children 1 year old or younger. Then, we estimated the mean plus 3 standard deviations of the lower component to define a seropositivity cutoff. In many cases the mixture model-based cutoffs were very unrealistic because the model failed to identify a reasonable seronegative distribution. We only examine agreement among antigens for which we could estimate reasonable cutoff values.

**Distribution of antibody levels among children who were presumed unexposed**: In longitudinal cohorts (Haiti, Kenya), we identified children who had antibody levels increase by at least 2 on the log10 scale. We then estimated the mean plus 3 standard deviations of the measurements before the change to define a seropositivity cutoff.

Below, we make all possible comparisons between methods, but not all three cutoff values are available for every antibody in every cohort. ROC-based cutoffs were available for *Giardia sp.*, *Cryptosporidium spp.*, and *E. histolytica* (Haiti, Tanzania only). Mixture model-based cutoffs were only available if mixture models fit a reasonable distribution for the lower component. Cutoff values based on the distribution of antibody measurements before children seroconverted could only be estimated in the two longitudinal cohorts (Haiti, Kenya).

Distribution figures at the beginning of each cohort’s estimates provide a visual summary and comparison of seropositivity cutoffs.

The step of defining seropositivity cutoffs presents challenges for enteropathogens, but our findings provide guidance for future studies. Our results demonstrate that within-sample approaches to estimate seropositivity cutoffs are feasible for enteropathogens, but important considerations must be addressed. Two-component mixture models fit the data and provided reasonable cutoff estimates only when restricted to an age range that included two clearly delineated subpopulations of seronegative and seropositive responses. For most pathogens studied, this required measurements among children <1 year old, an age range during which IgG responses still followed a bimodal distribution. The Tanzania study enrolled children 1 to 9 years old, and it was impossible to characterize the distribution of unexposed IgG levels with a mixture model for any enteropathogens except Giardia and E. histolytica (Figure 1 - supplement 1). Even in the Haiti and Kenya cohorts, which measured children <1 year, the only reliable approach to estimate seropositivity cutoffs for the highest transmission pathogens like ETEC and Campylobacter was to estimate a distribution among presumed unexposed by identifying measurements among children who subsequently experienced a large increase in IgG (Figure 1, Figure 1 - supplement 3), a strategy only possible in a longitudinal design. High levels of agreement (>95%) across multiple cutoff approaches, consistent with findings from trachoma serology (Migchelsen et al. 2017 PLOS NTDs doi:10.1371/journal.pntd.0005230), supports a pragmatic approach that could include multiple strategies within the same study, depending on data availability and on the empirical distribution of IgG response. Measuring a sufficient number of young children before primary exposure, preferably with longitudinal measurements, will help ensure that within-sample seropositivity cutoff estimation is possible.

# Script preamble

```
#-----------------------------
# preamble
#-----------------------------
library(here)
here::here()
```

```
## [1] "/Users/benarnold/enterics-seroepi"
```

```
# load packages
library(tidyverse)
library(psych)
library(knitr)
library(kableExtra)
  options(knitr.table.format = "html") 


# set up for parallel computing
# configure for a laptop (use only 3 cores)
library(foreach)
library(doParallel)
registerDoParallel(cores=3)


# bright color blind palette:  https://personal.sron.nl/~pault/ 
cblack <- "#000004FF"
cblue <- "#3366AA"
cteal <- "#11AA99"
cgreen <- "#66AA55"
cchartr <- "#CCCC55"
cmagent <- "#992288"
cred <- "#EE3333"
corange <- "#EEA722"
cyellow <- "#FFEE33"
cgrey <- "#777777"
```

# Leogane, Haiti

## Load the data

```
#-----------------------------
# load the formatted data
# created with 
# haiti-enteric-ab-data-format.Rmd ->
# Fig1-haiti-ab-distributions.Rmd
#-----------------------------
dl <- readRDS(here::here("data","haiti_analysis2.rds"))
```

## Figure of distributions and cutoffs

Limited to children \(\leq 1\) year old to ensure a sufficient number of uenxposed children.

```
pcols <- c(cred,corange,cgreen,cteal,cblue,cmagent)

log10labs <- c( 
  expression(10^0),
  expression(10^1),
  expression(10^2),
  expression(10^3),
  expression(10^4)
)

pmix <- ggplot(data=filter(dl,age<=1),aes(x=logmfi,group=pathogen,color=pathogen,fill=pathogen)) +
  facet_wrap(~antigenf,nrow=6,ncol=2,scales="free_y") +
  # plot empirical distribution
  geom_histogram(aes(y=..density..),alpha=0.7)+
  geom_density(aes(y=..density..),color=cgrey,fill=NA,alpha=0.7)+
  # add vertical lines for the cutoff values
  geom_vline(aes(xintercept=roccut)) +
  geom_vline(aes(xintercept=mixcut),linetype=2) +
  geom_vline(aes(xintercept=unexpcut),linetype=3)+
  # labels and formatting
  scale_x_continuous(limits = c(0,4.5),breaks = 0:4,labels=log10labs) +
  coord_cartesian(ylim=c(0,2)) +
  labs(x="Luminex Response (MFI-bg)") +
  scale_fill_manual(values=pcols) +
  scale_color_manual(values=pcols) +
  theme_minimal(base_size = 16) +
  theme(legend.position = "none")

pmix
```

```
## `stat_bin()` using `bins = 30`. Pick better value with `binwidth`.
```

## ROC vs Mixture Model cutoffs

For antibodies with both cutoffs available (*Giardia*, *Cryptosporidium*, *E. histolytica*), summarize classification agreement and Cohen’s Kappa.

```
# compare classification by ROC and mixture models 
# for Giardia, Cryptosporidium, and E. histolytica 
# the 3 pathogens with both
dcomp <- dl %>%
  ungroup() %>%
  filter(age<=1) %>%
  filter(antigen %in% c("vsp3","vsp5","cp17","cp23","leca")) %>% 
  mutate(antigen=factor(antigen))


dclass <- foreach(ab=levels(dcomp$antigen),.combine=rbind) %do% {
  dcompi <- dcomp %>% filter(antigen==ab)
  tabi <- as.vector(table(dcompi$posroc,dcompi$posmix))
  names(tabi) <- c("roc0mix0","roc1mix0","roc0mix1","roc1mix1")
  kappai <- psych::cohen.kappa(table(dcompi$posroc,dcompi$posmix))$kappa
  tabi <- data.frame(t(tabi))
  tabi <- tabi %>%
    mutate(Nobs=roc0mix0+roc1mix0+roc0mix1+roc1mix1,
           agreement=(roc0mix0+roc1mix1)/Nobs,
           kappa=kappai,
           antigen=ab) %>%
    select(antigen,Nobs,everything())
  tabi
}

knitr::kable(dclass,digits=3,caption="Summary of seropositivity classifications by ROC curve and Gaussian mixture models for Giardia (VSP-3 and VSP-5), Cryptosporidium (Cp17, Cp23), and E. histolytica (LecA)") %>%
  kable_styling(bootstrap_options = "striped",full_width = TRUE)
```

Summary of seropositivity classifications by ROC curve and Gaussian mixture models for Giardia (VSP-3 and VSP-5), Cryptosporidium (Cp17, Cp23), and E. histolytica (LecA)

| antigen | Nobs | roc0mix0 | roc1mix0 | roc0mix1 | roc1mix1 | agreement | kappa |
| --- | --- | --- | --- | --- | --- | --- | --- |
| cp17 | 59 | 34 | 0 | 4 | 21 | 0.932 | 0.858 |
| cp23 | 59 | 41 | 0 | 0 | 18 | 1.000 | 1.000 |
| leca | 59 | 52 | 0 | 1 | 6 | 0.983 | 0.914 |
| vsp3 | 59 | 45 | 0 | 3 | 11 | 0.949 | 0.848 |
| vsp5 | 59 | 42 | 0 | 5 | 12 | 0.915 | 0.774 |

## ROC vs unexposed cutoffs

For antibodies with both cutoffs available (*Giardia*, *Cryptosporidium*, *E. histolytica*), summarize classification agreement and Cohen’s Kappa.

```
dcomp <- dl %>%
  ungroup() %>%
  filter(age<=1) %>%
  filter(antigen %in% c("vsp3","vsp5","cp17","cp23","leca")) %>% 
  mutate(antigen=factor(antigen))


dclass2 <- foreach(ab=levels(dcomp$antigen),.combine=rbind) %do% {
  dcompi <- dcomp %>% filter(antigen==ab)
  tabi <- as.vector(table(dcompi$posroc,dcompi$posunex))
  names(tabi) <- c("roc0unexp0","roc1unexp0","roc0unexp1","roc1unexp1")
  kappai <- psych::cohen.kappa(table(dcompi$posroc,dcompi$posunex))$kappa
  tabi <- data.frame(t(tabi))
  tabi <- tabi %>%
    mutate(Nobs=roc0unexp0+roc1unexp0+roc0unexp1+roc1unexp1,
           agreement=(roc0unexp0+roc1unexp1)/Nobs,
           kappa=kappai,
           antigen=ab) %>%
    select(antigen,Nobs,everything())
  tabi
}

knitr::kable(dclass2,digits=3,caption="Summary of seropositivity classifications by ROC curve and distribution among unexposed for Giardia (VSP-3 and VSP-5), Cryptosporidium (Cp17, Cp23), and E. histolytica (LecA)") %>%
  kable_styling(bootstrap_options = "striped",full_width = TRUE)
```

Summary of seropositivity classifications by ROC curve and distribution among unexposed for Giardia (VSP-3 and VSP-5), Cryptosporidium (Cp17, Cp23), and E. histolytica (LecA)

| antigen | Nobs | roc0unexp0 | roc1unexp0 | roc0unexp1 | roc1unexp1 | agreement | kappa |
| --- | --- | --- | --- | --- | --- | --- | --- |
| cp17 | 59 | 38 | 4 | 0 | 17 | 0.932 | 0.846 |
| cp23 | 59 | 39 | 0 | 2 | 18 | 0.966 | 0.922 |
| leca | 59 | 50 | 0 | 3 | 6 | 0.949 | 0.772 |
| vsp3 | 59 | 47 | 0 | 1 | 11 | 0.983 | 0.946 |
| vsp5 | 59 | 47 | 0 | 0 | 12 | 1.000 | 1.000 |

## Mixture model vs unexposed cutoffs

```
#-----------------------------
# compare classification by mixture
# model and distribution among the unexposed
# for Giardia, Crypto, E. hist,
# Salmonella, Noro GI and GII
# the pathogens with both
#-----------------------------
dcomp <- dl %>%
  ungroup() %>%
  filter(age<=1) %>%
  filter(antigen %in% c("vsp3","vsp5","cp17","cp23","leca","salb","sald","norogi","norogii")) %>% 
  mutate(antigen=factor(antigen))


dclass3 <- foreach(ab=levels(dcomp$antigen),.combine=rbind) %do% {
  dcompi <- dcomp %>% filter(antigen==ab)
  tabi <- as.vector(table(dcompi$posmix,dcompi$posunex))
  names(tabi) <- c("mix0unexp0","mix1unexp0","mix0unexp1","mix1unexp1")
  kappai <- psych::cohen.kappa(table(dcompi$posmix,dcompi$posunex))$kappa
  tabi <- data.frame(t(tabi))
  tabi <- tabi %>%
    mutate(Nobs=mix0unexp0+mix1unexp0+mix0unexp1+mix1unexp1,
           agreement=(mix0unexp0+mix1unexp1)/Nobs,
           kappa=kappai,
           antigen=ab) %>%
    select(antigen,Nobs,everything())
  tabi
}

knitr::kable(dclass3,digits=3,caption="Summary of seropositivity classifications by Gaussian mixture model and distribution among unexposed for Giardia (VSP-3 and VSP-5), Cryptosporidium (Cp17, Cp23), E. histolytica (LecA), Salmonella (LPS D and LPS B), and Norovirus (Groups I and II)") %>%
  kable_styling(bootstrap_options = "striped",full_width = TRUE)
```

Summary of seropositivity classifications by Gaussian mixture model and distribution among unexposed for Giardia (VSP-3 and VSP-5), Cryptosporidium (Cp17, Cp23), E. histolytica (LecA), Salmonella (LPS D and LPS B), and Norovirus (Groups I and II)

| antigen | Nobs | mix0unexp0 | mix1unexp0 | mix0unexp1 | mix1unexp1 | agreement | kappa |
| --- | --- | --- | --- | --- | --- | --- | --- |
| cp17 | 59 | 34 | 8 | 0 | 17 | 0.864 | 0.710 |
| cp23 | 59 | 39 | 0 | 2 | 18 | 0.966 | 0.922 |
| leca | 59 | 50 | 0 | 2 | 7 | 0.966 | 0.856 |
| norogi | 59 | 36 | 0 | 3 | 20 | 0.949 | 0.891 |
| norogii | 59 | 39 | 3 | 0 | 17 | 0.949 | 0.882 |
| salb | 59 | 39 | 0 | 3 | 17 | 0.949 | 0.882 |
| sald | 59 | 47 | 9 | 0 | 3 | 0.847 | 0.347 |
| vsp3 | 59 | 45 | 2 | 0 | 12 | 0.966 | 0.902 |
| vsp5 | 59 | 42 | 5 | 0 | 12 | 0.915 | 0.774 |

# Asembo, Kenya

## Load the data

```
#-----------------------------
# load the formatted data
# with seropositivity indicators
# created with:
# asembo-enteric-ab-data-format.Rmd -->
# Fig1sup3-asembo-ab-distributions.Rmd
#-----------------------------
dl <- readRDS(here::here("data","asembo_analysis2.rds"))
```

## Figure of distributions and cutoffs

```
pcols <- c(cred,corange,cgreen,cteal,cblue,cmagent,cgrey)

log10labs <- c( 
  expression(10^0),
  expression(10^1),
  expression(10^2),
  expression(10^3),
  expression(10^4)
)

pmix <- ggplot(data=dl,aes(x=logmfi,group=pathogen,color=pathogen,fill=pathogen)) +
  facet_wrap(~antigenf,nrow=6,ncol=2,scales="free_y") +
  # plot empirical distribution
  geom_histogram(aes(y=..density..),alpha=0.7)+
  geom_density(aes(y=..density..),color=cgrey,fill=NA,alpha=0.7)+
  # add vertical lines for the cutoff values
  geom_vline(aes(xintercept=roccut)) +
  geom_vline(aes(xintercept=mixcut),linetype=2) +
  geom_vline(aes(xintercept=unexpcut),linetype=3)+
  # labels and formatting
  scale_x_continuous(limits = c(0,4.5),breaks = 0:4,labels=log10labs) +
  coord_cartesian(ylim=c(0,2)) +
  labs(x="Luminex Response (MFI-bg)") +
  scale_fill_manual(values=pcols) +
  scale_color_manual(values=pcols) +
  theme_minimal(base_size = 16) +
  theme(legend.position = "none")

pmix
```

```
## `stat_bin()` using `bins = 30`. Pick better value with `binwidth`.
```

## ROC vs Mixture Model cutoffs

For antibodies where both cutoffs are available (*Giardia* and *Cryptosporidium*), summarize classification agreement and Cohen’s Kappa.

```
#-----------------------------
# compare classification by ROC and mixture models 
# for Giardia and Cryptosporidium, the 2 pathogens
# with both
#-----------------------------
dcomp <- dl %>%
  filter(antigen %in% c("vsp3","vsp5","cp17","cp23")) %>% 
  mutate(antigen=factor(antigen))


dclass <- foreach(ab=levels(dcomp$antigen),.combine=rbind) %do% {
  dcompi <- dcomp %>% filter(antigen==ab)
  tabi <- as.vector(table(dcompi$posroc,dcompi$posmix))
  names(tabi) <- c("roc0mix0","roc1mix0","roc0mix1","roc1mix1")
  kappai <- psych::cohen.kappa(table(dcompi$posroc,dcompi$posmix))$kappa
  tabi <- data.frame(t(tabi))
  tabi <- tabi %>%
    mutate(Nobs=roc0mix0+roc1mix0+roc0mix1+roc1mix1,
           agreement=(roc0mix0+roc1mix1)/Nobs,
           kappa=kappai,
           antigen=ab) %>%
    select(antigen,Nobs,everything())
  tabi
}

knitr::kable(dclass,digits=3,caption="Summary of seropositivity classifications by ROC curve and Gaussian mixture models for Giardia (VSP-3 and VSP-5) and Cryptosporidium (Cp17, Cp23)") %>%
  kable_styling(bootstrap_options = "striped",full_width=TRUE)
```

Summary of seropositivity classifications by ROC curve and Gaussian mixture models for Giardia (VSP-3 and VSP-5) and Cryptosporidium (Cp17, Cp23)

| antigen | Nobs | roc0mix0 | roc1mix0 | roc0mix1 | roc1mix1 | agreement | kappa |
| --- | --- | --- | --- | --- | --- | --- | --- |
| cp17 | 445 | 301 | 0 | 7 | 137 | 0.984 | 0.964 |
| cp23 | 445 | 340 | 12 | 0 | 93 | 0.973 | 0.922 |
| vsp3 | 445 | 326 | 0 | 27 | 92 | 0.939 | 0.833 |
| vsp5 | 445 | 335 | 0 | 14 | 96 | 0.969 | 0.912 |

Classification was very consistent between the two approaches.

## ROC vs unexposed cutoffs

```
#-----------------------------
# compare classification by ROC and 
# distribution among the unexposed
# for Giardia and Cryptosporidium, 
# the 2 pathogens with both
#-----------------------------
dcomp <- dl %>%
  filter(antigen %in% c("vsp3","vsp5","cp17","cp23")) %>% 
  mutate(antigen=factor(antigen))


dclass2 <- foreach(ab=levels(dcomp$antigen),.combine=rbind) %do% {
  dcompi <- dcomp %>% filter(antigen==ab)
  tabi <- as.vector(table(dcompi$posroc,dcompi$posunex))
  names(tabi) <- c("roc0unexp0","roc1unexp0","roc0unexp1","roc1unexp1")
  kappai <- psych::cohen.kappa(table(dcompi$posroc,dcompi$posunex))$kappa
  tabi <- data.frame(t(tabi))
  tabi <- tabi %>%
    mutate(Nobs=roc0unexp0+roc1unexp0+roc0unexp1+roc1unexp1,
           agreement=(roc0unexp0+roc1unexp1)/Nobs,
           kappa=kappai,
           antigen=ab) %>%
    select(antigen,Nobs,everything())
  tabi
}

knitr::kable(dclass2,digits=3,caption="Summary of seropositivity classifications by ROC curve and distribution among unexposed for Giardia (VSP-3 and VSP-5) and Cryptosporidium (Cp17, Cp23)") %>%
  kable_styling(bootstrap_options = "striped",full_width=TRUE)
```

Summary of seropositivity classifications by ROC curve and distribution among unexposed for Giardia (VSP-3 and VSP-5) and Cryptosporidium (Cp17, Cp23)

| antigen | Nobs | roc0unexp0 | roc1unexp0 | roc0unexp1 | roc1unexp1 | agreement | kappa |
| --- | --- | --- | --- | --- | --- | --- | --- |
| cp17 | 445 | 288 | 0 | 20 | 137 | 0.955 | 0.899 |
| cp23 | 445 | 265 | 0 | 75 | 105 | 0.831 | 0.625 |
| vsp3 | 445 | 326 | 0 | 27 | 92 | 0.939 | 0.833 |
| vsp5 | 445 | 333 | 0 | 16 | 96 | 0.964 | 0.900 |

Classification was very consistent between the two approaches, but slightly lower for *Cryptosporidium* Cp23. The Cp23 cutoff value estimated using the presumed unexposed was much lower compared with ROC-based cutoff.

## Mixture model vs unexposed cutoffs

For antibodies where both cutoffs are available (*Giardia*, *Cryptosporidium*, *Campylobacter*), summarize classification agreement and Cohen’s Kappa.

```
#-----------------------------
# compare classification by mixture
# model and distribution among the unexposed
# for Giardia and Cryptosporidium, 
# and Campylobacter p39
# the 3 pathogens with both
#-----------------------------
dcomp <- dl %>%
  filter(antigen %in% c("vsp3","vsp5","cp17","cp23","p39")) %>% 
  mutate(antigen=factor(antigen))


dclass3 <- foreach(ab=levels(dcomp$antigen),.combine=rbind) %do% {
  dcompi <- dcomp %>% filter(antigen==ab)
  tabi <- as.vector(table(dcompi$posmix,dcompi$posunex))
  names(tabi) <- c("mix0unexp0","mix1unexp0","mix0unexp1","mix1unexp1")
  kappai <- psych::cohen.kappa(table(dcompi$posmix,dcompi$posunex))$kappa
  tabi <- data.frame(t(tabi))
  tabi <- tabi %>%
    mutate(Nobs=mix0unexp0+mix1unexp0+mix0unexp1+mix1unexp1,
           agreement=(mix0unexp0+mix1unexp1)/Nobs,
           kappa=kappai,
           antigen=ab) %>%
    select(antigen,Nobs,everything())
  tabi
}

knitr::kable(dclass3,digits=3,caption="Summary of seropositivity classifications by Gaussian mixture model and distribution among unexposed for Giardia (VSP-3 and VSP-5), Cryptosporidium (Cp17, Cp23), and Campylobacter (p39)") %>%
  kable_styling(bootstrap_options = "striped",full_width=TRUE)
```

Summary of seropositivity classifications by Gaussian mixture model and distribution among unexposed for Giardia (VSP-3 and VSP-5), Cryptosporidium (Cp17, Cp23), and Campylobacter (p39)

| antigen | Nobs | mix0unexp0 | mix1unexp0 | mix0unexp1 | mix1unexp1 | agreement | kappa |
| --- | --- | --- | --- | --- | --- | --- | --- |
| cp17 | 445 | 288 | 0 | 13 | 144 | 0.971 | 0.935 |
| cp23 | 445 | 265 | 0 | 87 | 93 | 0.804 | 0.560 |
| p39 | 445 | 64 | 0 | 1 | 380 | 0.998 | 0.991 |
| vsp3 | 445 | 326 | 0 | 0 | 119 | 1.000 | 1.000 |
| vsp5 | 445 | 333 | 0 | 2 | 110 | 0.996 | 0.988 |

Classification was very consistent between the two approaches, but slightly lower for *Cryptosporidium* Cp23. The Cp23 cutoff value estimated using the presumed unexposed was much lower compared with mixture model-based cutoff.

# Kongwa, Tanzania

In the Tanzania study, children were enrolled between ages 1-9 years. Enteric pathogen transmission was sufficiently high that most children were exposed by age 1, so there were relatively few children who were unexposed. This meant that mixture models only estimated reasonable seropositivity cutoffs for *Giardia* and *E. histolytica*. Comparisons between ROC-based cutoffs and mixture model-based cutoffs focus on these two pathogens.

## Load data

```
#-----------------------------
# load the formatted data
# created with 
# kongwa-enteric-ab-data-format.Rmd ->
# Fig1sup1-kongwa-ab-distributions.Rmd
#-----------------------------
dl <- readRDS(here::here("data","kongwa_analysis2.rds"))
```

## Figure of distributions and cutoffs

Limited to children \(\leq 1\) year old to ensure a sufficient number of uenxposed children.

```
dplot <- dl %>%
  filter(age<=1) %>%
  group_by(antigen) %>%
  mutate(plotroc=min(roccut))

pcols <- c(cred,corange,cgreen,cteal,cblue,cmagent,cgrey,cchartr)


log10labs <- c( 
  expression(10^0),
  expression(10^1),
  expression(10^2),
  expression(10^3),
  expression(10^4)
)

pmix <- ggplot(data=dplot,aes(x=logmfi,group=pathogen,color=pathogen,fill=pathogen)) +
  facet_wrap(~antigenf,nrow=6,ncol=2,scales="free_y") +
  # plot empirical distribution
  geom_histogram(aes(y=..density..),alpha=0.7)+
  geom_density(aes(y=..density..),color=cgrey,fill=NA,alpha=0.7)+
  # add vertical lines for the cutoff values
  geom_vline(aes(xintercept=plotroc)) +
  geom_vline(aes(xintercept=mixcut),linetype=2) +
  # labels and formatting
  scale_x_continuous(limits = c(0,4.5),breaks = 0:4,labels=log10labs) +
  coord_cartesian(ylim=c(0,2)) +
  labs(x="Luminex Response (MFI-bg)") +
  scale_fill_manual(values=pcols) +
  scale_color_manual(values=pcols) +
  theme_minimal(base_size = 16) +
  theme(legend.position = "none")

pmix
```

```
## `stat_bin()` using `bins = 30`. Pick better value with `binwidth`.
```

## ROC vs Mixture Model cutoffs

In cases where both cutoffs are available (*Giardia*, and *E. histolytica*), summarize agreement between classifications.

```
# for Giardia and E. histolytica, the 2 pathogens
# with both
dcomp <- dl %>%
  filter(age==1) %>%
  filter(antigen %in% c("vsp3","vsp5","leca")) %>% 
  mutate(antigen=factor(antigen))


dclass <- foreach(ab=levels(dcomp$antigen),.combine=rbind) %do% {
  dcompi <- dcomp %>% filter(antigen==ab)
  tabi <- as.vector(table(dcompi$posroc,dcompi$posmix))
  names(tabi) <- c("roc0mix0","roc1mix0","roc0mix1","roc1mix1")
  kappai <- psych::cohen.kappa(table(dcompi$posroc,dcompi$posmix))$kappa
  tabi <- data.frame(t(tabi))
  tabi <- tabi %>%
    mutate(Nobs=roc0mix0+roc1mix0+roc0mix1+roc1mix1,
           agreement=(roc0mix0+roc1mix1)/Nobs,
           kappa=kappai,
           antigen=ab) %>%
    select(antigen,Nobs,everything())
  tabi
}

knitr::kable(dclass,digits=2,caption="Summary of seropositivity classifications by ROC curve and Gaussian mixture models for Giardia (VSP-3) and E. histolytica (LecA)") %>%
  kable_styling(bootstrap_options = "striped",full_width=TRUE)
```

Summary of seropositivity classifications by ROC curve and Gaussian mixture models for Giardia (VSP-3) and E. histolytica (LecA)

| antigen | Nobs | roc0mix0 | roc1mix0 | roc0mix1 | roc1mix1 | agreement | kappa |
| --- | --- | --- | --- | --- | --- | --- | --- |
| leca | 408 | 309 | 38 | 0 | 61 | 0.91 | 0.71 |
| vsp3 | 408 | 97 | 0 | 3 | 308 | 0.99 | 0.98 |
| vsp5 | 408 | 97 | 2 | 0 | 309 | 1.00 | 0.99 |

Classification was very consistent between the two approaches for *Giardia*, but slightly lower for *E. histolytica*.

# Session Info

```
sessionInfo()
```

```
## R version 3.5.3 (2019-03-11)
## Platform: x86_64-apple-darwin15.6.0 (64-bit)
## Running under: macOS High Sierra 10.13.6
## 
## Matrix products: default
## BLAS: /System/Library/Frameworks/Accelerate.framework/Versions/A/Frameworks/vecLib.framework/Versions/A/libBLAS.dylib
## LAPACK: /Library/Frameworks/R.framework/Versions/3.5/Resources/lib/libRlapack.dylib
## 
## locale:
## [1] en_US.UTF-8/en_US.UTF-8/en_US.UTF-8/C/en_US.UTF-8/en_US.UTF-8
## 
## attached base packages:
## [1] parallel  stats     graphics  grDevices utils     datasets  methods  
## [8] base     
## 
## other attached packages:
##  [1] doParallel_1.0.11     iterators_1.0.9       foreach_1.4.4        
##  [4] kableExtra_0.8.0.0001 knitr_1.22            psych_1.8.4          
##  [7] forcats_0.3.0         stringr_1.4.0         dplyr_0.8.0.1        
## [10] purrr_0.3.2           readr_1.1.1           tidyr_0.8.3          
## [13] tibble_2.1.1          ggplot2_3.1.1         tidyverse_1.2.1      
## [16] here_0.1             
## 
## loaded via a namespace (and not attached):
##  [1] tidyselect_0.2.5  xfun_0.6          reshape2_1.4.3   
##  [4] haven_2.1.0       lattice_0.20-38   colorspace_1.3-2 
##  [7] viridisLite_0.3.0 htmltools_0.3.6   yaml_2.2.0       
## [10] rlang_0.3.4       pillar_1.4.0      withr_2.1.2      
## [13] foreign_0.8-71    glue_1.3.1        modelr_0.1.2     
## [16] readxl_1.1.0      plyr_1.8.4        munsell_0.5.0    
## [19] gtable_0.3.0      cellranger_1.1.0  rvest_0.3.2      
## [22] codetools_0.2-16  evaluate_0.13     labeling_0.3     
## [25] highr_0.8         broom_0.4.4       Rcpp_1.0.1       
## [28] backports_1.1.4   scales_1.0.0      jsonlite_1.6     
## [31] mnormt_1.5-5      hms_0.4.2         packrat_0.4.9-3  
## [34] digest_0.6.18     stringi_1.4.3     grid_3.5.3       
## [37] rprojroot_1.3-2   cli_1.1.0         tools_3.5.3      
## [40] magrittr_1.5      lazyeval_0.2.2    crayon_1.3.4     
## [43] pkgconfig_2.0.2   rsconnect_0.8.8   xml2_1.2.0       
## [46] lubridate_1.7.4   assertthat_0.2.1  rmarkdown_1.12   
## [49] httr_1.4.0        rstudioapi_0.9.0  R6_2.4.0         
## [52] nlme_3.1-137      compiler_3.5.3
```
