# Supplementary material for: Enteropathogen antibody dynamics and force of infection among children in low-resource settings
Source: eLife. 2019 Aug 19;8:e45594. doi: 10.7554/eLife.45594 (PMC6746552; doi:10.7554/eLife.45594)
Supplement: Supplementary file 3. [file elife-45594-supp3.zip › SIFile3.html]

Enteropathogen antibody dynamics and force of infection among children in low-resource settings


Code 

- Show All Code
- Hide All Code

# Enteropathogen antibody dynamics and force of infection among children in low-resource settings

### Supplementary Information File 3. Joint distributions of antibody response.

# Notebook summary

This notebook summarizes the joint distribution of antibody responses in each cohort. The first section creates main text Figure 2, which summarizes comparisons between different antigens for the same pathogen plus a comparison between ETEC and cholera, where the toxin B subunit is known to elicit cross-reactivity. Each scatter plot also includes the spearman’s rank correlation coefficient (\(\rho\)). After creating Figure 2, the notebook creates supplemental figures that include pairs plots for the joint distribution of every combination of enteric antibody responses in each cohort.

# Script preamble

```
#-----------------------------
# preamble
#-----------------------------
# set to local workspace
library(here)
```

```
## here() starts at /Users/benarnold/enterics-seroepi
```

```
here::here()
```

```
## [1] "/Users/benarnold/enterics-seroepi"
```

```
# load packages
library(tidyverse)
```

```
## ── Attaching packages ────────────────────────────── tidyverse 1.2.1 ──
```

```
## ✔ ggplot2 3.1.1       ✔ purrr   0.3.2  
## ✔ tibble  2.1.1       ✔ dplyr   0.8.0.1
## ✔ tidyr   0.8.3       ✔ stringr 1.4.0  
## ✔ readr   1.1.1       ✔ forcats 0.3.0
```

```
## ── Conflicts ───────────────────────────────── tidyverse_conflicts() ──
## ✖ dplyr::filter() masks stats::filter()
## ✖ dplyr::lag()    masks stats::lag()
```

```
library(scales)
```

```
## 
## Attaching package: 'scales'
```

```
## The following object is masked from 'package:purrr':
## 
##     discard
```

```
## The following object is masked from 'package:readr':
## 
##     col_factor
```

```
library(viridis)
```

```
## Loading required package: viridisLite
```

```
## 
## Attaching package: 'viridis'
```

```
## The following object is masked from 'package:scales':
## 
##     viridis_pal
```

```
library(ellipse)
```

```
## 
## Attaching package: 'ellipse'
```

```
## The following object is masked from 'package:graphics':
## 
##     pairs
```

```
library(RColorBrewer)

# set up for parallel computing
library(foreach)
```

```
## 
## Attaching package: 'foreach'
```

```
## The following objects are masked from 'package:purrr':
## 
##     accumulate, when
```

```
library(doParallel)
```

```
## Loading required package: iterators
```

```
## Loading required package: parallel
```

```
registerDoParallel(cores = detectCores() - 1)

# bright color blind palette:  https://personal.sron.nl/~pault/ 
cblack <- "#000004FF"
cblue <- "#3366AA"
cteal <- "#11AA99"
cgreen <- "#66AA55"
cchartr <- "#CCCC55"
cmagent <- "#992288"
cred <- "#EE3333"
corange <- "#EEA722"
cyellow <- "#FFEE33"
cgrey <- "#777777"

# custom log labels
log10labs <- c( 
  expression(10^0),
  expression(10^1),
  expression(10^2),
  expression(10^3),
  expression(10^4)
)
```

# Load and format data

```
#--------------------------------
# load the various datasets
#--------------------------------
dh <- readRDS(here::here("data","haiti_analysis.rds"))
dk <- readRDS(here::here("data","asembo_analysis.rds"))
dt <- readRDS(here::here("data","kongwa_analysis.rds"))

#--------------------------------
# subset to common variables
# and append
#--------------------------------
dh <- dh %>% 
  mutate(country="Haiti") %>%
  select(country,id,sid=sampleid,antigen,antigenf,logmfi)
dk <- dk %>% 
  mutate(country="Kenya",sid = ifelse(time=="A","1","2"),id=as.integer(childid)) %>%
  select(country,id,sid,antigen,antigenf,logmfi)
  
dt <- dt %>% 
  mutate(country="Tanzania",sid="1") %>%
  select(country,id,sid,antigen,antigenf,logmfi)

dall <- bind_rows(dh,dk,dt)
```

```
## Warning in bind_rows_(x, .id): binding character and factor vector,
## coercing into character vector
```

```
## Warning in bind_rows_(x, .id): Unequal factor levels: coercing to character
```

```
## Warning in bind_rows_(x, .id): binding character and factor vector,
## coercing into character vector

## Warning in bind_rows_(x, .id): binding character and factor vector,
## coercing into character vector

## Warning in bind_rows_(x, .id): binding character and factor vector,
## coercing into character vector

## Warning in bind_rows_(x, .id): binding character and factor vector,
## coercing into character vector
```

```
#--------------------------------
# create antigen groupings for
# comparisons
# drop obs not contributing
#--------------------------------
d <- dall %>%
  mutate(comp= ifelse(antigen %in% c("vsp3","vsp5"),"Giardia",NA),
         comp= ifelse(antigen %in% c("cp17","cp23"),"Cryptosporidium",comp),
         comp= ifelse(antigen %in% c("p18","p39"),"Campylobacter",comp),
         comp= ifelse(antigen %in% c("sald","salb"),"Salmonella",comp),
         comp= ifelse(antigen %in% c("cholera","etec"),"ETEC\nV. cholerae",comp),
         comp= ifelse(antigen %in% c("norogi","norogii"),"Norovirus",comp),
         comp= ifelse(country=="Haiti" & antigen =="etec",NA,comp),
         comp= factor(comp,levels=c("Giardia","Cryptosporidium","Campylobacter","Salmonella","ETEC\nV. cholerae","Norovirus"))
         ) %>% 
  mutate(xlab=ifelse(antigen %in% c("vsp3","vsp5"),"VSP-3",NA),
         ylab=ifelse(antigen %in% c("vsp3","vsp5"),"VSP-5",NA),
         xlab=ifelse(antigen %in% c("cp17","cp23"),"Cp17",xlab),
         ylab=ifelse(antigen %in% c("cp17","cp23"),"Cp23",ylab),
         xlab=ifelse(antigen %in% c("p18","p39"),"p18",xlab),
         ylab=ifelse(antigen %in% c("p18","p39"),"p39",ylab),
         xlab=ifelse(antigen %in% c("salb","sald"),"LPS Group B",xlab),
         ylab=ifelse(antigen %in% c("salb","sald"),"LPS Group D",ylab),
         xlab=ifelse(antigen %in% c("cholera","etec"),"Cholera toxin B subunit",xlab),
         ylab=ifelse(antigen %in% c("cholera","etec"),"ETEC LT B subunit",ylab),
         xlab=ifelse(antigen %in% c("norogi","norogii"),"GII.4.NO",xlab),
         ylab=ifelse(antigen %in% c("norogi","norogii"),"GI.4",ylab)
         ) %>%
  filter(!is.na(comp))

#--------------------------------
# for antigen each pair, 
# label it as "x" or "y" to 
# spread it to wide format
#--------------------------------
dw <- d %>%
  mutate(xy=ifelse(antigen %in% c("vsp3","cp17","sald","p18","cholera","norogii"),"x","y")) %>%
  select(country,comp,xlab,ylab,id,sid,xy,logmfi) %>%
  spread(xy,logmfi) %>%
  mutate(country=factor(country,levels=c("Haiti","Kenya","Tanzania")))
```

# Estimate correlations and smooths

```
#--------------------------------
# estimate spearman's correlation
# within each country, comparison
#--------------------------------
dcorr <- dw %>%
  group_by(country,comp) %>%
  mutate(corxy=cor(x,y,method="spearman",use="pairwise.complete.obs") ) %>%
  summarize(corxy=max(corxy,na.rm=T))
```

```
#--------------------------------
# estimate smooths, trimmed to
# drop the bottom and top 1% of
# data in each comparison
# to avoid edge effects
#--------------------------------
dsmooths <- foreach(countryi=levels(dw$country),.combine=rbind) %:%
                      foreach(compi=levels(dw$comp),.combine=rbind) %do% {
                        pd <- filter(dw,country==countryi & comp==compi)
                        if(nrow(pd)>0) {
                          xqs <- quantile(pd$x,probs=c(0.01,0.99),na.rm=TRUE)
                          newd <- data.frame(x=seq(xqs[1],xqs[2],by=0.01))
                          lfit <- loess(y~x,data=pd)
                          return(data.frame(country=countryi,comp=compi,x=newd,y=predict(lfit,newdata=newd)))
                        }
                        
  
}
```

# Figure 2

Summary composite figure, **Figure 2 in the main text**

```
vircols <- viridis(n=4,alpha=1,begin=0.2,end=0.97)
pcol <- vircols[2]

# grab labels
dlabs <- dw %>% select(country,comp,xlab,ylab) %>% group_by(country,comp) %>% slice(1)

complot <- ggplot(data=dw,aes(x=x,y=y)) +
  facet_grid(comp~country) +
  geom_point(pch=19,color=pcol,alpha=0.1) +
  geom_line(data=dsmooths,aes(x=x,y=y),col="black",size=1.2)+
  geom_text(data=dcorr,
            aes(x=0.5,y=4.3,label=paste("rho ==",sprintf("%1.2f",corxy)) ),
            parse=TRUE, col="black")   +
  geom_text(data=dlabs,aes(x=2.3,y=0.1,label=xlab),color="gray40",angle=0)+
  geom_text(data=dlabs,aes(x=0.1,y=2.3,label=ylab),color="gray40",angle=90)+
  scale_x_continuous(limits=c(0,4.6),breaks=0:4,labels = log10labs)+
  scale_y_continuous(limits=c(0,4.6),breaks=0:4,labels = log10labs)+
  coord_equal() +
  labs(x="Luminex Response (MFI-bg)",y="Luminex Response (MFI-bg)") +
  theme_minimal(base_size=12) +
  theme(
    strip.text.x=element_text(size=12),
    strip.text.y=element_text(size=12,angle=0),
    legend.position="none"
  )


complot
```

```
## Warning: Removed 9076 rows containing missing values (geom_point).
```

```
# save PDF and TIFF versions
ggsave(here::here("figs","Fig2-ab-scatter-composite.pdf"),plot=complot,device=cairo_pdf,width=13,height=15)
```

```
## Warning: Removed 9076 rows containing missing values (geom_point).
```

```
ggsave(here::here("figs","Fig2-ab-scatter-composite.TIFF"),plot=complot,device="tiff",width=13,height=15)
```

```
## Warning: Removed 9076 rows containing missing values (geom_point).
```

# Individual country pairs plots

The above figure was created as a synthesis across individual country pairs plots. Below, the script creates each pairs plot that shows the joint relationship between every combination of antigens in each cohort.

```
#----------------------------------
# correlation ellipse
#----------------------------------
myellipse<-function(x,y,...){
  maxx <- max(x,na.rm=TRUE)
  minx <- min(x,na.rm=TRUE)
  maxy <- max(y,na.rm=TRUE)
  miny <- min(y,na.rm=TRUE)
  midx <- (maxx+minx)/2
  midy <- (maxy+miny)/2
  corxy <- cor(x,y,method="spearman",use="pairwise.complete.obs")
  colgroup<-cut(corxy,breaks=seq(-0.1,1,length=20),labels=F)
  viridiscols <- viridis(20)
  cols<-viridiscols[colgroup]
  xyc <-sprintf("%1.2f",corxy)
  xyc[grep("NA",xyc)]<-""
  exy <- ellipse(corxy,centre=c(midx,midy),scale=c((maxx-minx)/6,(maxy-miny)/6))
  polygon(exy,col=alpha(cols,alpha=0.5))
  lines(exy)
  if(!is.na(corxy)) {
    if(corxy<0.8) {
      text(midx,midy,xyc,cex=0.8)
    } else{
      text(maxx,midy-((maxy-miny)/3),xyc,cex=0.8,adj=1)
    }
  }
  
}


#----------------------------------
# scatter plot with loess fit
# (trimmed to reduce edge effects)
#----------------------------------
scatterloess<-function(x,y,cex=0.4,...){
  ld <- data.frame(x,y)
  ld <- ld[complete.cases(ld),]
  if(nrow(ld)>0) {
    points(ld$x,ld$y,pch=19,cex=cex,col=alpha('black',alpha=0.2))
    viridiscols <- viridis(11)
    lfit <- loess(y~x,data=ld)
    xqs <- quantile(x,probs=c(0.01,0.99),na.rm=TRUE)
    px <- seq(xqs[1],xqs[2],by=0.01)
    py <- predict(lfit,newdata=data.frame(x=px))
    lines(px,py,col=viridiscols[1],lwd=1.5)
  }
  
}
```

## Haiti

```
# list the enteric antigens in Haiti and formatted labels for them
mbavars <- c("vsp3","vsp5","cp17","cp23","leca","salb","sald","etec","norogi","norogii")
mbalabs <- c("Giardia\nVSP-3","Giardia\nVSP-5","Cryptosporidium\nCp17","Cryptosporidium\nCp23","E. histolytica\nLecA","Salmonella\nLPS B","Salmonella\nLPS D","ETEC\nLT B subunit","Norovirus\nGI", "Norovirus\nGII")

hmat <- dall %>%
  filter(country=="Haiti") %>%
  select(id,sid,antigen,logmfi) %>%
  spread(antigen,logmfi) 

pairs(hmat[mbavars],labels=mbalabs,cex=0.1,las=1, 
      upper.panel=scatterloess,
      lower.panel=myellipse
)
```

## Kenya

```
# list the enteric antigens in Asembo Kenya and formatted labels for them
mbavars <- c("vsp3","vsp5","cp17","cp23","leca","salb","sald","etec","cholera","p18","p39")

mbalabs <- c("Giardia\nVSP-3","Giardia\nVSP-5","Cryptosporidium\nCp17","Cryptosporidium\nCp23","E. histolytica\nLecA","Salmonella\nLPS B","Salmonella\nLPS D","ETEC\nLT B subunit","Cholera\ntoxin B subunit","Campylobacter\np18","Campylobacter\np39")

kmat <- dall %>%
  filter(country=="Kenya") %>%
  select(id,sid,antigen,logmfi) %>%
  spread(antigen,logmfi)

pairs(kmat[mbavars],labels=mbalabs,cex=0.1,las=1,
      upper.panel=scatterloess,
      lower.panel=myellipse
)
```

## Tanzania

There are a few blank panels in this figure. The reason is that some antigens were included only in year 1, and the cholera beta toxin was only included in years 2-4. Table 1 and the Methods of the article include additional details.

```
# list the enteric antigens in Kongwa, Tanzania and formatted labels for them
mbavars <- c("vsp3","vsp5","cp17","cp23","leca","salb","sald","etec","cholera","p18","p39")

mbalabs <- c("Giardia\nVSP-3","Giardia\nVSP-5","Cryptosporidium\nCp17","Cryptosporidium\nCp23",
             "E. histolytica\nLecA","Salmonella\nLPS B","Salmonella\nLPS D","ETEC\nLT B subunit","Cholera\ntoxin B subunit","Campylobacter\np18","Campylobacter\np39")

tmat <- dall %>%
  filter(country=="Tanzania") %>%
  select(id,sid,antigen,logmfi) %>%
  spread(antigen,logmfi)
pairs(tmat[mbavars],labels=mbalabs,cex=0.1,las=1,
      upper.panel=scatterloess,
      lower.panel=myellipse
)
```

# Session Info

```
sessionInfo()
```

```
## R version 3.5.3 (2019-03-11)
## Platform: x86_64-apple-darwin15.6.0 (64-bit)
## Running under: macOS High Sierra 10.13.6
## 
## Matrix products: default
## BLAS: /Library/Frameworks/R.framework/Versions/3.5/Resources/lib/libRblas.0.dylib
## LAPACK: /Library/Frameworks/R.framework/Versions/3.5/Resources/lib/libRlapack.dylib
## 
## locale:
## [1] en_US.UTF-8/en_US.UTF-8/en_US.UTF-8/C/en_US.UTF-8/en_US.UTF-8
## 
## attached base packages:
## [1] parallel  stats     graphics  grDevices utils     datasets  methods  
## [8] base     
## 
## other attached packages:
##  [1] doParallel_1.0.11  iterators_1.0.9    foreach_1.4.4     
##  [4] RColorBrewer_1.1-2 ellipse_0.4.1      viridis_0.5.1     
##  [7] viridisLite_0.3.0  scales_1.0.0       forcats_0.3.0     
## [10] stringr_1.4.0      dplyr_0.8.0.1      purrr_0.3.2       
## [13] readr_1.1.1        tidyr_0.8.3        tibble_2.1.1      
## [16] ggplot2_3.1.1      tidyverse_1.2.1    here_0.1          
## 
## loaded via a namespace (and not attached):
##  [1] tidyselect_0.2.5 xfun_0.6         reshape2_1.4.3   haven_2.1.0     
##  [5] lattice_0.20-38  colorspace_1.3-2 htmltools_0.3.6  yaml_2.2.0      
##  [9] rlang_0.3.4      pillar_1.4.0     foreign_0.8-71   glue_1.3.1      
## [13] withr_2.1.2      modelr_0.1.2     readxl_1.1.0     plyr_1.8.4      
## [17] munsell_0.5.0    gtable_0.3.0     cellranger_1.1.0 rvest_0.3.2     
## [21] codetools_0.2-16 psych_1.8.4      evaluate_0.13    knitr_1.22      
## [25] broom_0.4.4      Rcpp_1.0.1       backports_1.1.4  jsonlite_1.6    
## [29] gridExtra_2.3    mnormt_1.5-5     hms_0.4.2        digest_0.6.18   
## [33] stringi_1.4.3    grid_3.5.3       rprojroot_1.3-2  cli_1.1.0       
## [37] tools_3.5.3      magrittr_1.5     lazyeval_0.2.2   crayon_1.3.4    
## [41] pkgconfig_2.0.2  xml2_1.2.0       lubridate_1.7.4  assertthat_0.2.1
## [45] rmarkdown_1.12   httr_1.4.0       rstudioapi_0.9.0 R6_2.4.0        
## [49] nlme_3.1-137     compiler_3.5.3
```
