# Supplementary material for: Enteropathogen antibody dynamics and force of infection among children in low-resource settings
Source: eLife. 2019 Aug 19;8:e45594. doi: 10.7554/eLife.45594 (PMC6746552; doi:10.7554/eLife.45594)
Supplement: Supplementary file 4. [file elife-45594-supp4.zip › SIFile4.html]

Enteropathogen antibody dynamics and force of infection among children in low-resource settings


Code 

- Show All Code
- Hide All Code

# Enteropathogen antibody dynamics and force of infection among children in low-resource settings

### Supplementary Information File 4. Antibody response among Kenyan children with- and without infections detected in stool

# Notebook Summary

The Asembo, Kenya cohort study monitored children weekly from enrollment to the final follow-up to measure caregiver reported diarrhea symptoms. Study staff collected stool from children with reported diarrhea in the past 7 days at enrollment or during weekly follow-up. The stool was tested with a commercial immunoassay for infection with *Cryptosporidium parvum* and *Giardia intestinalis* (Quik Chek, TechLab, Inc, Blacksburg, VA). Additionally, 18S and GP60-based PCR assays were used to test for infection with *Cryptosporidium*. For additional details about stool collection and testing in this cohort, please refer to the original trial’s publication:

Morris JF, Murphy J, Fagerli K, Schneeberger C, Jaron P, Moke F, Juma J, et al. A Randomized Controlled Trial to Assess the Impact of Ceramic Water Filters on Prevention of Diarrhea and Cryptosporidiosis in Infants and Young Children-Western Kenya, 2013. *Am. J. Trop. Med. Hyg.* 2018; 98(5): 1260–68. https://doi.org/10.4269/ajtmh.17-0731.

Here, we used the stool-based infection results to compare IgG responses between children who had a confirmed infection during the study versus those who did not.

The analyses show that there were a substantial number of children in the study who had serological evidence of infection with *Cryptosporidium* or *Giardia* despite not having an infection detected in diarrheal stool. For example, the seroprevalence to *Giardia* VSP-3 or VSP-5 increased from 1% (0%, 5%) at enrollment to 22% (14%, 32%) at follow-up among children without confirmed infection. This suggests that many children were either not shedding oocysts at the time of diarrheal stool collection, or the infections were asymptomatic.

The analyses also show that nearly all children with infections identified in stool seroconverted between enrollment and the final follow-up visit; three children with confirmed *Cryptosporidium* infection did not show evidence of elevated IgG to Cp17 or Cp23 antigens, and two children with confirmed *Giardia* infection did not show evidence of elevated IgG to VSP-3 or VSP-5 antigens. An analysis of IgG levels by time since infection suggested that antibody waning could not explain the absence of seroconversion in these children. IgG levels remained high over periods of 0-28 weeks from infection, particularly for *Cryptosporidium*; *Giardia* IgG levels waned slightly after 10 weeks from infection among the 25 children with confirmed infections during the follow-up period.

# Script preamble

```
#-----------------------------
# preamble
#-----------------------------
library(here)
```

```
## here() starts at /Users/benarnold/enterics-seroepi
```

```
here::here()
```

```
## [1] "/Users/benarnold/enterics-seroepi"
```

```
# load packages
library(tidyverse)
```

```
## ── Attaching packages ────────────────────────────── tidyverse 1.2.1 ──
```

```
## ✔ ggplot2 3.1.1       ✔ purrr   0.3.2  
## ✔ tibble  2.1.1       ✔ dplyr   0.8.0.1
## ✔ tidyr   0.8.3       ✔ stringr 1.4.0  
## ✔ readr   1.1.1       ✔ forcats 0.3.0
```

```
## ── Conflicts ───────────────────────────────── tidyverse_conflicts() ──
## ✖ dplyr::filter() masks stats::filter()
## ✖ dplyr::lag()    masks stats::lag()
```

```
library(lubridate)
```

```
## 
## Attaching package: 'lubridate'
```

```
## The following object is masked from 'package:here':
## 
##     here
```

```
## The following object is masked from 'package:base':
## 
##     date
```

```
library(kableExtra)
```

```
## 
## Attaching package: 'kableExtra'
```

```
## The following object is masked from 'package:dplyr':
## 
##     group_rows
```

```
# set up for parallel computing
# configure for a laptop (use only 3 cores)
library(foreach)
```

```
## 
## Attaching package: 'foreach'
```

```
## The following objects are masked from 'package:purrr':
## 
##     accumulate, when
```

```
library(doParallel)
```

```
## Loading required package: iterators
```

```
## Loading required package: parallel
```

```
registerDoParallel(cores = detectCores() - 1)

# safe color blind palette
# http://jfly.iam.u-tokyo.ac.jp/color/
# http://www.cookbook-r.com/Graphs/Colors_(ggplot2)/
cbPalette <- c("#999999", "#E69F00", "#56B4E9", "#009E73", "#F0E442", "#0072B2", "#D55E00", "#CC79A7")
```

# Load the data

Among 133 children with stool tested during weekly diarrheal surveillance (n=216 stools tested), 132 also had serology measurements at enrollment or follow-up. There were 15 children with serology measurements at enrollment but not at follow-up, and 1 child with a serology measurement at follow-up but not enrollment; 116 children had longitudinal IgG measurements at both time points. Thus, there were 131 children with stool testing who had IgG measured at enrollment, and 117 children with stool testing who had IgG measured at follow-up. The file `asembo_stool.rds` includes the stool testing results. The file `asembo_analysis2.rds` includes the processed serology testing results.

```
#-----------------------------
# load the stool data
# created with 
# asembo-enteric-ab-data-format.Rmd
#-----------------------------
ds <- readRDS(here::here("data","asembo_stool.rds"))

# set measurement time to "B" to merge to the serology data
# by matching the stool measurements to follow-up IgG
# measurements. Doing this makes it easier to estimate IgG levels with
# time since infection
ds <- ds %>%
  mutate(time=factor("B",levels=c("A","B"))) %>%
  mutate(childid=as.character(childid))

#-----------------------------
# load the formatted serology data
# created with 
# asembo-enteric-ab-data-format.Rmd -->
# Fig1sup3-asembo-ab-distributions.Rmd
#-----------------------------
dl <- readRDS(here::here("data","asembo_analysis2.rds")) %>%
  ungroup() 

#-----------------------------
# merge stool testing data
# onto serology data
# Note: individual children 
# were measured between 1 and 5 
# times in the stool dataset
#
# filter to children who had
# stool measurements
#-----------------------------
dl2 <- dl %>% 
  left_join(ds,by=c("childid","time")) %>%
  filter(childid %in% unique(ds$childid)) %>%
  filter(pathogen %in% c("Cryptosporidium","Giardia"))
```

```
## Warning: Column `childid` joining factor and character vector, coercing
## into character vector
```

# Cryptosporidium

## IgG levels by infection status

**Figure caption:** *Cryptosporidium* IgG levels at enrollment and 6-months later at follow-up among 132 Kenyan children ages 4-18 months old who had one or more diarrhea episodes during weekly surveillance. Diarrheal stools were tested for *Cryptosporidium* infection by PCR or immunoassay (Quik CheckTM, TechLab, Inc. Blacksburg, VA) as described in Morris et al. (2018). Heavy lines indicate medians and boxes contain the interquartile range. Among 132 children with stool tested during the follow-up period, 17 children tested positive for *Cryptosporidium*. Horizontal dashed lines mark seropositivity cutoffs for each antigen based on ROC curves of known positive and negative specimens (dashed) or based on Gaussian mixture model (dot dash) as described in the Methods.

```
# ---------------------------
# identify if ever had a 
# detected Crypto infection
# ---------------------------
dcr1 <- dl2 %>%
  group_by(childid,antigenf) %>%
  filter(pathogen=="Cryptosporidium") %>%
  mutate(crypto = max(ifelse(stool_crypto==1,1,0),na.rm=T),
         cryptof = ifelse(crypto==1,"stool-confirmed infection","no stool-confirmed infection"),
         cryptof = factor(cryptof,levels=c("no stool-confirmed infection","stool-confirmed infection")),
         visit=ifelse(time=="A","Enrollment","Follow-up"),
         visit=factor(visit)
  ) %>%
  group_by(antigenf,childid,time) %>%
  slice(1) %>%
  ungroup()

# ---------------------------
# plot distributions
# ---------------------------

log10labs <- c( 
  expression(10^0),
  expression(10^1),
  expression(10^2),
  expression(10^3),
  expression(10^4)
)

pcols <- cbPalette[c(6,7)]
pcrypto_box <- ggplot(data=dcr1,aes(x=visit,y=logmfi,color=cryptof)) +
  facet_grid(antigenf~cryptof)+
  geom_hline(aes(yintercept=roccut),linetype="dashed")+
  geom_hline(aes(yintercept=mixcut),linetype="dotdash")+ 
  # plot children without stool-confirmed infections
  geom_jitter(width=0.25,size=2,alpha=0.5)+
  geom_boxplot(color="black",fill=NA,outlier.shape = NA)+
  scale_y_continuous(breaks=0:4,labels=log10labs)+
  scale_color_manual(values=pcols,guide=guide_legend(include=FALSE))+
  scale_fill_manual(values=pcols)+
  coord_cartesian(ylim=c(0,4.5)) +
  labs(x="Measurement",y="IgG Luminex response (MFI-bg)")+
  theme_gray(base_size=14)+
  theme(
    panel.grid.minor.x=element_blank(),
    legend.position = "none"
  )
pcrypto_box
```

The figure shows that the median IgG levels were higher at follow-up among children with a confirmed *Cryptosporidium* infection. However, there were many children who had no confirmed infection that showed serological evidence of infection during follow-up, presumably because they were not shedding pathogen at the time of diarrheal stool testing or had asymptomatic infections. Many children showed serological evidence of previous exposure to *Cryptosporidium* at enrollment.

## Mean IgG levels by infection status

**Figure caption:** Mean IgG levels at enrollment and at follow-up 6-months later among 132 Kenyan children ages 4-18 months old who had one or more diarrhea episodes during weekly surveillance. Diarrheal stools were tested for *Cryptosporidium* infection by PCR or immunoassay (Quik CheckTM, TechLab, Inc. Blacksburg, VA) as described in Morris et al. (2018). Among 132 children tested during the follow-up period, 17 children tested positive for *Cryptosporidium*. Error bars indicate 95% confidence intervals.

```
# ---------------------------
# collapse by measurement 
# time and infection status
# mean IgG and 95% CI
# by antibody
# ---------------------------
dcr2 <- dcr1 %>%
  mutate(n=1) %>%
  group_by(antigenf,time,cryptof) %>%
  summarize(nobs = sum(n),
            igg_mu = mean(logmfi),
            igg_sd = sd(logmfi),
  )
dcr2 <- dcr2 %>%
  mutate(igg_se = igg_sd/sqrt(nobs),
         igg_lb = igg_mu - 2*igg_se,
         igg_ub = igg_mu + 2*igg_se) %>%
  mutate(visit = ifelse(time=="A","Enrollment","Follow-up"),
         visit = factor(visit)) %>%
  ungroup() %>%
  select(antigenf,visit,cryptof,nobs,contains("igg"))
```

```
log10labs <- c( 
  expression(10^1),
  expression(10^2),
  expression(10^3),
  expression(10^4)
)

pcols <- cbPalette[c(6,2)]
pcrypto_igg <- ggplot(data=dcr2,aes(x=visit, y=igg_mu,color=cryptof,fill=cryptof,shape=cryptof)) +
  facet_grid(.~antigenf) +
  geom_errorbar(aes(ymin=igg_lb,ymax=igg_ub),
                width=0.1,
                position=position_nudge(x=rep(c(-0.1,0.1),4))
                )+
  geom_point(size=3,bg="white",
             position=position_nudge(x=rep(c(-0.1,0.1),4))
             )+
  scale_y_continuous(breaks=1:4,labels=log10labs)+
  scale_shape_manual(values=c(21,19))+
  scale_color_manual(values=pcols)+
  coord_cartesian(ylim=c(1,4))+
  labs(x="Measurement",y="Geometric mean IgG Luminex response (MFI-bg)")+
  theme_gray(base_size=14)+
  theme(
    legend.position="right",
    legend.title = element_blank()
  )

pcrypto_igg
```

```
dcr2 %>% 
  arrange(antigenf,cryptof,visit) %>%
  select(antigenf,cryptof,visit,nobs,igg_mu,igg_lb,igg_ub) %>%
  knitr::kable(digits=2,
             caption="Raw estimates: geometric mean IgG levels by infection status and visit",
             row.names = FALSE) %>%
  kable_styling(bootstrap_options = c("striped"),full_width = TRUE)
```

Raw estimates: geometric mean IgG levels by infection status and visit

| antigenf | cryptof | visit | nobs | igg\_mu | igg\_lb | igg\_ub |
| --- | --- | --- | --- | --- | --- | --- |
| Cryptosporidium Cp17 | no stool-confirmed infection | Enrollment | 114 | 1.70 | 1.53 | 1.88 |
| Cryptosporidium Cp17 | no stool-confirmed infection | Follow-up | 100 | 2.48 | 2.23 | 2.73 |
| Cryptosporidium Cp17 | stool-confirmed infection | Enrollment | 17 | 2.11 | 1.47 | 2.76 |
| Cryptosporidium Cp17 | stool-confirmed infection | Follow-up | 17 | 3.53 | 3.02 | 4.03 |
| Cryptosporidium Cp23 | no stool-confirmed infection | Enrollment | 114 | 1.71 | 1.52 | 1.90 |
| Cryptosporidium Cp23 | no stool-confirmed infection | Follow-up | 100 | 2.47 | 2.23 | 2.70 |
| Cryptosporidium Cp23 | stool-confirmed infection | Enrollment | 17 | 2.22 | 1.64 | 2.80 |
| Cryptosporidium Cp23 | stool-confirmed infection | Follow-up | 17 | 3.50 | 2.98 | 4.01 |

IgG levels to Cp17 and Cp23 increased from enrollment to follow-up among all children, but the increase was substantially larger among the 17 children who had a confirmed *Cryptosporidium* infection in the diarrheal stool surveillance.

## Seroprevalence by infection status

**Figure caption:** Seroprevalence at enrollment and at follow-up 6-months later among 132 Kenyan children ages 4-18 months old who had one or more diarrhea episodes during weekly surveillance. Diarrheal stools were tested for *Cryptosporidium* infection by PCR or immunoassay (Quik CheckTM, TechLab, Inc. Blacksburg, VA) as described in Morris et al. (2018). Among 132 children with stool tested during the follow-up period, 17 children tested positive for *Cryptosporidium*. Error bars indicate 95% exact binomial confidence intervals. Children were classified as seropositive if their IgG levels were above ROC-derived seropositivity cutoffs for either Cp17 or Cp23, as described in the Methods.

```
# ---------------------------
# collapse by measurement 
# time and infection status
# combine info over antibodies
# seroprev and exact 
# binomial 95% CI
# ---------------------------

dcr3 <- dcr1 %>%
  group_by(childid,time) %>%
  mutate(seropos = max(seropos)) %>%
  slice(1) %>%
  mutate(n=1) %>%
  group_by(time,cryptof) %>%
  summarize(nobs = sum(n),
            npos = sum(seropos)
  )
  
spci <- foreach(i=1:nrow(dcr3),.combine=rbind) %do% {
  btest <- binom.test(x=dcr3$npos[i],
                      n=dcr3$nobs[i],
                      alternative="two.sided")
  
  data.frame(time=dcr3$time[i],
             cryptof=dcr3$cryptof[i],
             seroprev=btest$estimate,
             seroprev_lb=btest$conf.int[1],
             seroprev_ub=btest$conf.int[2])
}
dcr3 <- dcr3 %>% 
  left_join(spci,by=c("time","cryptof")) %>%
  mutate(visit = ifelse(time=="A","Enrollment","Follow-up"),
         visit = factor(visit),
         antigenf = "Cryptosporidium Cp17 or Cp23") %>%
  ungroup() %>%
  select(antigenf,visit,cryptof,nobs,npos,starts_with("seroprev"))
```

```
pcols <- cbPalette[c(6,2)]
pcrypto_sp <- ggplot(data=dcr3,aes(x=visit, y=seroprev,color=cryptof,fill=cryptof,shape=cryptof)) +
  facet_grid(.~antigenf)+
  geom_errorbar(aes(ymin=seroprev_lb,ymax=seroprev_ub),
                width=0.1,
                position=position_nudge(x=rep(c(-0.1,0.1),2))
                )+
  geom_point(size=3,bg="white",
             position=position_nudge(x=rep(c(-0.1,0.1),2))
             )+
  scale_y_continuous(breaks=seq(0,1,by=0.2),labels = sprintf("%1.0f",seq(0,1,by=0.2)*100))+
  scale_shape_manual(values=c(21,19))+
  scale_color_manual(values=pcols)+
  coord_cartesian(ylim=c(0,1))+
  # guides()+
  labs(x="Measurement",y="Seroprevalence (%)")+
  theme_gray(base_size=14)+
  theme(
    legend.position="right",
    legend.title = element_blank()
  )

pcrypto_sp
```

```
dcr3 %>% 
  arrange(antigenf,cryptof,visit) %>%
  select(antigenf,cryptof,visit,nobs,npos,seroprev,seroprev_lb,seroprev_ub) %>%
  knitr::kable(digits=2,
             caption="Raw estimates: seroprevalence by infection status and visit",
             row.names = FALSE) %>%
  kable_styling(bootstrap_options = c("striped"),full_width = TRUE)
```

Raw estimates: seroprevalence by infection status and visit

| antigenf | cryptof | visit | nobs | npos | seroprev | seroprev\_lb | seroprev\_ub |
| --- | --- | --- | --- | --- | --- | --- | --- |
| Cryptosporidium Cp17 or Cp23 | no stool-confirmed infection | Enrollment | 114 | 18 | 0.16 | 0.10 | 0.24 |
| Cryptosporidium Cp17 or Cp23 | no stool-confirmed infection | Follow-up | 100 | 47 | 0.47 | 0.37 | 0.57 |
| Cryptosporidium Cp17 or Cp23 | stool-confirmed infection | Enrollment | 17 | 6 | 0.35 | 0.14 | 0.62 |
| Cryptosporidium Cp17 or Cp23 | stool-confirmed infection | Follow-up | 17 | 14 | 0.82 | 0.57 | 0.96 |

All children had higher seroprevalence to *Cryptosporidium* Cp17 or Cp23 at follow-up. Although confidence intervals were wide due to small numbers of children with a confirmed infection in a diarrheal stool, seroprevalence increased to 82% (14/17) among that subgroup, far higher than the 47% (47/100) among children who did not have a confirmed infection.

## IgG trajectories among those with confirmed infection

There were 205 children in the cohort with longitudinal serology measurements. Of these, 89 children did not have a diarrheal stool tested. Among the 116 children with longitudinal serological measurements who were also tested for infection in diarrheal stools during weekly surveillance, 99 were negative and 17 (15%) were positive for *Cryptosporidium*. Summarize the longitudinal trajectories for the 17 children who were confirmed positive for infection during follow-up.

**Figure caption:** IgG Luminex response for antibodies to *Cryptosporidium parvum* among 17 children with antibodies measured who also had confirmed infections during follow-up based on diarrheal stool testing. Diarrheal stools were tested for *Cryptosporidium* infection by PCR or immunoassay (Quik CheckTM, TechLab, Inc. Blacksburg, VA) as described in Morris et al. (2018). Horizontal dashed lines mark seropositivity cutoffs for each antigen based on ROC curves of known positive and negative specimens (dashed) or based on Gaussian mixture model (dot dash) as described in the Methods. Children who did not have at least one measurement above seropositivity cutoffs are labeled with color.

```
dcr4 <- dl2 %>%
  group_by(childid,antigenf) %>%
  filter(pathogen=="Cryptosporidium" & (time=="A" | stool_crypto==1) ) %>%
  mutate(nobs=n()) %>%
  filter(nobs>1) %>%
  # identify seroconverters
  mutate(seroposA = max(ifelse(time=="A" & seropos==1, 1, 0)),
         seroposB = max(ifelse(time=="B" & seropos==1, 1, 0)),
         seroconv = factor(case_when(
           seroposA==0 & seroposB==1 ~ "seroconversion",
           seroposA==0 & seroposB==0 ~ "no seroconversion",
           seroposA==1  ~ "previously exposed",
         ),levels=c("previously exposed","seroconversion","no seroconversion"))
  ) %>%
  mutate(visit=ifelse(time=="A","Enrollment","Follow-up"),
         visit=factor(visit))

# make figure of results
log10labs <- c( 
  expression(10^0),
  expression(10^1),
  expression(10^2),
  expression(10^3),
  expression(10^4)
)

pcols <- cbPalette[c(2,3,4,7,8)]
pcrypto_long <- ggplot(data=filter(dcr4,seroconv!="no seroconversion"),aes(x=visit,y=logmfi,group=childid)) +
  facet_grid(.~antigenf)+
  geom_hline(aes(yintercept=roccut),linetype="dashed")+
  geom_hline(aes(yintercept=mixcut),linetype="dotdash")+ 
  # plot children who seroconverted or were previously exposed
  geom_point(size=2,alpha=0.4)+
  geom_line(alpha=0.4)+
  # add children who didn't seroconvert. Color them by ID
  geom_line(data=filter(dcr4,seroconv=="no seroconversion"),
             aes(group=childid,color=childid), alpha=0.8)+
  geom_point(data=filter(dcr4,seroconv=="no seroconversion"),
             aes(group=childid,fill=childid),pch=21,size=2,color="black",alpha=0.9)+
  scale_y_continuous(breaks=0:4,labels=log10labs)+
  scale_color_manual(values=pcols,guide=guide_legend(include=FALSE))+
  scale_fill_manual(values=pcols)+
  coord_cartesian(ylim=c(0,4.5)) +
  labs(x="Measurement",y="IgG Luminex response (MFI-bg)")+
  theme_gray(base_size=14)+
  theme(
    panel.grid.minor.x=element_blank(),
    legend.position = "right"
  )
pcrypto_long
```

```
# save PDF and TIFF versions
ggsave(here::here("figs","asembo-mfi-inf-crypto.pdf"),plot=pcrypto_long,device=cairo_pdf,width=5,height=4)
ggsave(here::here("figs","asembo-mfi-inf-crypto.TIFF"),plot=pcrypto_long,device="tiff",width=5,height=4)
```

Longitudinal IgG trajectories show that there were three children (IDs: 10, 102, 42) who had confirmed *Cryptosporidium* infection in a diarrhea stool between enrollment and follow-up, but did not seroconvert to either the Cp17 or Cp23 antigens. A recent assessment in Bangladesh (Kabir et al. 2018 CID; https://www.ncbi.nlm.nih.gov/pubmed/29718129) estimated the immunoassay specificity as 97% for *Cryptosporidium* infection, so false positives are theoretically possible though unlikely. Two children (ID: 58, 65) showed evidence of seroconversion by only one antigen. Five children with confirmed infections between enrollment and follow-up were seropositive at enrollment to both antigens, suggesting a prior infection.

## IgG levels by time since confirmed infection

Among children with confirmed infections, examine IgG levels with time since infection to see if there is evidence for substantial decay over short periods.

**Figure caption:** IgG levels by weeks between infection detected in stool and antibody measurement at follow-up among 17 children with confirmed infections. Diarrheal stools were tested for *Cryptosporidium* infection by PCR or immunoassay (Quik CheckTM, TechLab, Inc. Blacksburg, VA) as described in Morris et al. (2018). Horizontal dashed lines mark seropositivity cutoffs for each antigen based on ROC curves of known positive and negative specimens (dashed) or based on Gaussian mixture model (dot dash) as described in the Methods. Measurements below seropositivity cutoffs are labeled with color.

```
# note: there is one child w/ a confirmed crypto infection
# who was not measured for serology at follow-up. childid==223
# That leaves 17 children with confirmed infections.
dcr5 <- dl2 %>%
  filter(pathogen=="Cryptosporidium" & stool_crypto==1)

# restrict to children with follow-up antibody measurements
dcr5 <- dcr5 %>%
  filter(time=="B") %>%
  mutate(infwk = week-stool_week)

# confirm stool measures preceded antibody measures
table(dcr5$infwk)
```

```
## 
##  5  8  9 13 14 16 18 19 20 21 23 25 
##  2  2  2  2  4  6  2  2  2  6  2  2
```

```
# make figure of results
log10labs <- c( 
  expression(10^0),
  expression(10^1),
  expression(10^2),
  expression(10^3),
  expression(10^4)
)

pcols <- cbPalette[c(2,3,4,6,7,8)]
pcrypto_time <- ggplot(data=filter(dcr5,logmfi>=serocut),aes(x=infwk,y=logmfi)) +
  facet_grid(.~antigenf)+
  geom_hline(aes(yintercept=roccut),linetype="dashed")+
  geom_hline(aes(yintercept=mixcut),linetype="dotdash")+ 
  geom_point(size=3,pch=21,color="black",fill="black",alpha=0.5)+
  # add points below seropositivity cutoff, and color by childid
  geom_point(data=filter(dcr5,logmfi<serocut),
             aes(x=infwk,y=logmfi,fill=childid),
             size=3,pch=21,color="black")+
  scale_x_continuous(breaks=seq(4,26,by=2))+
  scale_y_continuous(breaks=0:4,labels=log10labs)+
  scale_fill_manual(values=pcols)+
  coord_cartesian(xlim=c(4,26),ylim=c(0,4.5)) +
  labs(x="weeks between infection in diarrheal stool and antibody measurement",y="IgG Luminex response (MFI-bg)")+
  theme(
    panel.grid.minor.x=element_blank()
  )
pcrypto_time
```

```
# save PDF and TIFF versions
ggsave(here::here("figs","asembo-mfi-v-inf-wks-crypto.pdf"),plot=pcrypto_time,device=cairo_pdf,width=7,height=4)
ggsave(here::here("figs","asembo-mfi-v-inf-wks-crypto.TIFF"),plot=pcrypto_time,device="tiff",width=7,height=4)
```

Most IgG responses remained high irrespective of time since infection over a range of 5 to 25 weeks. As with the longitudinal trajectories, this figure shows the three children (IDs: 10, 102, 42) who had confirmed infections but did not have IgG levels above seropositivity cutoffs for either Cp17 or Cp23 at their follow-up measurement 9-23 weeks later. Time since infection does not seem to explain why these three children had low IgG levels.

# Giardia

## IgG levels by infection status

**Figure caption:** *Giardia intestinalis* IgG levels at enrollment and 6-months later at follow-up among 132 Kenyan children ages 4-18 months old who had one or more diarrhea episodes during weekly surveillance. Diarrheal stools were tested for *Giardia* infection by immunoassay (Quik CheckTM, TechLab, Inc. Blacksburg, VA) as described in Morris et al. (2018). Heavy lines indicate medians and boxes contain the interquartile range. Among 132 children tested during the follow-up period, 25 children tested positive for *Giardia*. Horizontal dashed lines mark seropositivity cutoffs for each antigen based on ROC curves of known positive and negative specimens (dashed) or based on Gaussian mixture model (dot dash) as described in the Methods.

```
# ---------------------------
# identify if ever had a 
# detected Crypto infection
# ---------------------------
dgi1 <- dl2 %>%
  group_by(childid,antigenf) %>%
  filter(pathogen=="Giardia") %>%
  mutate(giardia = max(ifelse(stool_giardia==1,1,0),na.rm=T),
         giardiaf = ifelse(giardia==1,"stool-confirmed infection","no stool-confirmed infection"),
         giardiaf = factor(giardiaf,levels=c("no stool-confirmed infection","stool-confirmed infection")),
         visit=ifelse(time=="A","Enrollment","Follow-up"),
         visit=factor(visit)
  ) %>%
  group_by(antigenf,childid,time) %>%
  slice(1) %>%
  ungroup()

# ---------------------------
# plot distributions
# ---------------------------

log10labs <- c( 
  expression(10^0),
  expression(10^1),
  expression(10^2),
  expression(10^3),
  expression(10^4)
)

pcols <- cbPalette[c(6,7)]
pgiardia_box <- ggplot(data=dgi1,aes(x=visit,y=logmfi,color=giardiaf)) +
  facet_grid(antigenf~giardiaf)+
  geom_hline(aes(yintercept=roccut),linetype="dashed")+
  geom_hline(aes(yintercept=mixcut),linetype="dotdash")+ 
  geom_jitter(width=0.25,size=2,alpha=0.5)+
  geom_boxplot(color="black",fill=NA,outlier.shape = NA)+
  scale_y_continuous(breaks=0:4,labels=log10labs)+
  scale_color_manual(values=pcols,guide=guide_legend(include=FALSE))+
  scale_fill_manual(values=pcols)+
  coord_cartesian(ylim=c(0,4.5)) +
  labs(x="Measurement",y="IgG Luminex response (MFI-bg)")+
  theme_gray(base_size=14)+
  theme(
    panel.grid.minor.x=element_blank(),
    legend.position = "none"
  )
pgiardia_box
```

The figure shows that the median IgG levels were higher at follow-up among children with a confirmed *Giardia* infection. However, there were many children who had no confirmed infection that showed serological evidence of infection during follow-up, presumably because they were not shedding pathogen at the time of diarrheal stool testing or had asymptomatic infections. Many children with a confirmed infection during follow-up showed serological evidence of previous exposure to *Giardia* at enrollment, as indicated by IgG responses above seropositivity cutoffs.

## Mean IgG levels by infection status

**Figure caption:** Mean *Giardia intestinalis* IgG levels at enrollment and at follow-up 6-months later among 132 Kenyan children ages 4-18 months old who had one or more diarrhea episodes during weekly surveillance. Diarrheal stools were tested for *Giardia* infection by immunoassay (Quik CheckTM, TechLab, Inc. Blacksburg, VA) as described in Morris et al. (2018). Among 132 children tested during the follow-up period, 25 children tested positive for *Giardia*. Error bars indicate 95% confidence intervals.

```
# ---------------------------
# collapse by measurement 
# time and infection status
# mean IgG and 95% CI
# by antibody
# ---------------------------
dgi2 <- dgi1 %>%
  mutate(n=1) %>%
  group_by(antigenf,time,giardiaf) %>%
  summarize(nobs = sum(n),
            igg_mu = mean(logmfi),
            igg_sd = sd(logmfi),
  )
dgi2 <- dgi2 %>%
  mutate(igg_se = igg_sd/sqrt(nobs),
         igg_lb = igg_mu - 2*igg_se,
         igg_ub = igg_mu + 2*igg_se) %>%
  mutate(visit = ifelse(time=="A","Enrollment","Follow-up"),
         visit = factor(visit)) %>%
  ungroup() %>%
  select(antigenf,visit,giardiaf,nobs,contains("igg"))
```

```
log10labs <- c( 
  expression(10^0),
  expression(10^1),
  expression(10^2),
  expression(10^3),
  expression(10^4)
)

pcols <- cbPalette[c(6,2)]
pgiardia_igg <- ggplot(data=dgi2,aes(x=visit, y=igg_mu,color=giardiaf,fill=giardiaf,shape=giardiaf)) +
  facet_grid(.~antigenf) +
  geom_errorbar(aes(ymin=igg_lb,ymax=igg_ub),
                width=0.1,
                position=position_nudge(x=rep(c(-0.1,0.1),4))
                )+
  geom_point(size=3,bg="white",
             position=position_nudge(x=rep(c(-0.1,0.1),4))
             )+
  scale_y_continuous(breaks=0:4,labels=log10labs)+
  scale_shape_manual(values=c(21,19))+
  scale_color_manual(values=pcols)+
  coord_cartesian(ylim=c(0,4))+
  labs(x="Measurement",y="Geometric mean IgG Luminex resonse (MFI-bg)")+
  theme_gray(base_size=14)+
  theme(
    legend.position="right",
    legend.title = element_blank()
  )

pgiardia_igg
```

```
dgi2 %>% 
  arrange(antigenf,giardiaf,visit) %>%
  select(antigenf,giardiaf,visit,nobs,igg_mu,igg_lb,igg_ub) %>%
  knitr::kable(digits=2,
             caption="Raw estimates: geometric mean IgG levels by visit and infection status",
             row.names = FALSE) %>%
  kable_styling(bootstrap_options = c("striped"),full_width = TRUE)
```

Raw estimates: geometric mean IgG levels by visit and infection status

| antigenf | giardiaf | visit | nobs | igg\_mu | igg\_lb | igg\_ub |
| --- | --- | --- | --- | --- | --- | --- |
| Giardia VSP-3 | no stool-confirmed infection | Enrollment | 106 | 0.78 | 0.68 | 0.88 |
| Giardia VSP-3 | no stool-confirmed infection | Follow-up | 92 | 1.51 | 1.23 | 1.79 |
| Giardia VSP-3 | stool-confirmed infection | Enrollment | 25 | 1.69 | 1.12 | 2.25 |
| Giardia VSP-3 | stool-confirmed infection | Follow-up | 25 | 3.64 | 3.29 | 3.98 |
| Giardia VSP-5 | no stool-confirmed infection | Enrollment | 106 | 0.80 | 0.70 | 0.90 |
| Giardia VSP-5 | no stool-confirmed infection | Follow-up | 92 | 1.53 | 1.25 | 1.80 |
| Giardia VSP-5 | stool-confirmed infection | Enrollment | 25 | 1.68 | 1.13 | 2.24 |
| Giardia VSP-5 | stool-confirmed infection | Follow-up | 25 | 3.64 | 3.30 | 3.98 |

Mean IgG levels to VSP-3 and VSP-5 increased from enrollment to follow-up among all children, but the increase was substantially larger among the 25 children who had a confirmed *Giardia* infection in the diarrheal stool surveillance.

## Seroprevalence by infection status

**Figure caption:** *Giardia intestinalis* seroprevalence at enrollment and at follow-up 6-months later among 132 Kenyan children ages 4-18 months old who had one or more diarrhea episodes during weekly surveillance. Diarrheal stools were tested for *Giardia* infection by immunoassay (Quik CheckTM, TechLab, Inc. Blacksburg, VA) as described in Morris et al. (2018). Among 132 children with stool tested during the follow-up period, 25 children tested positive for *Giardia*. Error bars indicate 95% exact binomial confidence intervals. Children were classified as seropositive if their IgG levels were above ROC-derived seropositivity cutoffs for either VSP-3 or VSP-5, as described in the Methods.

```
# ---------------------------
# collapse by measurement 
# time and infection status
# combine info over antibodies
# seroprev and exact 
# binomial 95% CI
# ---------------------------

dgi3 <- dgi1 %>%
  group_by(childid,time) %>%
  mutate(seropos = max(seropos)) %>%
  slice(1) %>%
  mutate(n=1) %>%
  group_by(time,giardiaf) %>%
  summarize(nobs = sum(n),
            npos = sum(seropos)
  )
  
spci_g <- foreach(i=1:nrow(dgi3),.combine=rbind) %do% {
  btest <- binom.test(x=dgi3$npos[i],
                      n=dgi3$nobs[i],
                      alternative="two.sided")
  
  data.frame(time=dgi3$time[i],
             giardiaf=dgi3$giardiaf[i],
             seroprev=btest$estimate,
             seroprev_lb=btest$conf.int[1],
             seroprev_ub=btest$conf.int[2])
}

dgi3 <- dgi3 %>% 
  left_join(spci_g,by=c("time","giardiaf")) %>%
  mutate(visit = ifelse(time=="A","Enrollment","Follow-up"),
         visit = factor(visit),
         antigenf = "Giardia VSP-3 or VSP-5") %>%
  ungroup() %>%
  select(antigenf,visit,giardiaf,nobs,npos,starts_with("seroprev"))
```

```
pcols <- cbPalette[c(6,2)]
pgiardia_sp <- ggplot(data=dgi3,aes(x=visit, y=seroprev,color=giardiaf,fill=giardiaf,shape=giardiaf)) +
  facet_grid(.~antigenf)+
  geom_errorbar(aes(ymin=seroprev_lb,ymax=seroprev_ub),
                width=0.1,
                position=position_nudge(x=rep(c(-0.1,0.1),2))
                )+
  geom_point(size=3,bg="white",
             position=position_nudge(x=rep(c(-0.1,0.1),2))
             )+
  scale_y_continuous(breaks=seq(0,1,by=0.2),labels = sprintf("%1.0f",seq(0,1,by=0.2)*100))+
  scale_shape_manual(values=c(21,19))+
  scale_color_manual(values=pcols)+
  coord_cartesian(ylim=c(0,1))+
  # guides()+
  labs(x="Measurement",y="Seroprevalence (%)")+
  theme_gray(base_size=14)+
  theme(
    legend.position="right",
    legend.title = element_blank()
  )

pgiardia_sp
```

```
dgi3 %>% 
  arrange(antigenf,giardiaf,visit) %>%
  select(antigenf,giardiaf,visit,nobs,npos,seroprev,seroprev_lb,seroprev_ub) %>%
  knitr::kable(digits=2,
             caption="Raw estimates: seroprevalence by visit and infection status",
             row.names = FALSE) %>%
  kable_styling(bootstrap_options = c("striped"),full_width = TRUE)
```

Raw estimates: seroprevalence by visit and infection status

| antigenf | giardiaf | visit | nobs | npos | seroprev | seroprev\_lb | seroprev\_ub |
| --- | --- | --- | --- | --- | --- | --- | --- |
| Giardia VSP-3 or VSP-5 | no stool-confirmed infection | Enrollment | 106 | 1 | 0.01 | 0.00 | 0.05 |
| Giardia VSP-3 or VSP-5 | no stool-confirmed infection | Follow-up | 92 | 20 | 0.22 | 0.14 | 0.32 |
| Giardia VSP-3 or VSP-5 | stool-confirmed infection | Enrollment | 25 | 7 | 0.28 | 0.12 | 0.49 |
| Giardia VSP-3 or VSP-5 | stool-confirmed infection | Follow-up | 25 | 23 | 0.92 | 0.74 | 0.99 |

All children had higher seroprevalence to *Giardia* VSP-3 or VSP-5 at follow-up. Although confidence intervals were wide due to small numbers of children with a confirmed infection in a diarrheal stool, seroprevalence increased to 92% (23/25) among that subgroup, far higher than the 22% (20/92) among children who did not have a confirmed infection.

## IgG trajectories among those with confirmed infection

**Figure caption:** IgG Luminex response among 25 children with antibodies measured who also had confirmed *Giardia intestinalis* infections during follow-up based on diarrheal stool testing. Diarrheal stools were tested for *Giardia* infection by immunoassay (Quik Check TM, TechLab, Inc. Blacksburg, VA) as described in Morris et al. (2018). Horizontal dashed lines mark seropositivity cutoffs for each antigen based on ROC curves of known positive and negative specimens (dashed) or based on Gaussian mixture model (dot dash) as described in the Methods. Children whose measurements were below the ROC seropositivity cutoff at follow-up are labeled with color.

```
dgi <- dl2 %>%
  group_by(childid,antigenf) %>%
  filter(pathogen=="Giardia" & (time=="A" | stool_giardia==1) ) %>%
  mutate(nobs=n()) %>%
  filter(nobs>1) %>%
  # identify seroconverters
  mutate(seroposA = max(ifelse(time=="A" & seropos==1, 1, 0)),
         seroposB = max(ifelse(time=="B" & seropos==1, 1, 0)),
         seroconv = factor(case_when(
           seroposA==0 & seroposB==1 ~ "seroconversion",
           seroposB==0 ~ "no seroconversion",
           seroposA==1 & seroposB==1 ~ "previously exposed",
         ),levels=c("previously exposed","seroconversion","no seroconversion"))
  ) %>%
  mutate(visit=ifelse(time=="A","Enrollment","Follow-up"),
         visit=factor(visit))

# make figure of results
log10labs <- c( 
  expression(10^0),
  expression(10^1),
  expression(10^2),
  expression(10^3),
  expression(10^4)
)

pcols <- cbPalette[c(2,3,4)]
pgiar_long <- ggplot(data=filter(dgi,seroconv!="no seroconversion"),aes(x=visit,y=logmfi,group=childid)) +
  facet_grid(.~antigenf)+
  geom_hline(aes(yintercept=roccut),linetype="dashed")+
  geom_hline(aes(yintercept=mixcut),linetype="dotdash")+ 
  # plot children who seroconverted or were previously exposed
  geom_point(size=2,alpha=0.4)+
  geom_line(alpha=0.4)+
  # add children who didn't seroconvert. Color them by ID
  geom_line(data=filter(dgi,seroconv=="no seroconversion"),
             aes(group=childid,color=childid), alpha=0.8)+
  geom_point(data=filter(dgi,seroconv=="no seroconversion"),
             aes(group=childid,fill=childid),pch=21,size=2,color="black",alpha=0.8)+
  scale_y_continuous(breaks=0:4,labels=log10labs)+
  scale_color_manual(values=pcols,guide=guide_legend(include=FALSE))+
  scale_fill_manual(values=pcols)+
  coord_cartesian(ylim=c(0,4.5)) +
  labs(x="Measurement",y="IgG Luminex response (MFI-bg)")+
  theme_gray(base_size=14)+
  theme(
    panel.grid.minor.x=element_blank()
  )
pgiar_long
```

```
# save PDF and TIFF versions
ggsave(here::here("figs","asembo-mfi-inf-giardia.pdf"),plot=pgiar_long,device=cairo_pdf,width=5,height=4)
ggsave(here::here("figs","asembo-mfi-inf-giardia.TIFF"),plot=pgiar_long,device="tiff",width=5,height=4)
```

Longitudinal IgG trajectories show that there were two children (IDs: 101, 37) who had confirmed *Giardia* infection in a diarrhea stool between enrollment and follow-up, but did not seroconvert to either the VSP-3 or VSP-5 antigens based on the ROC cutoff (though IgG levels did exceed the mixture model-based cutoff). Child ID=14 was one of two children infected at their first measurement, at enrollment. Seven children with confirmed infections between enrollment and follow-up were seropositive at enrollment to both antigens based on the ROC cutoff, suggesting a prior infection.

## IgG levels by time since confirmed infection

**Figure caption:** IgG levels by weeks between infection detected in stool and antibody measurement at follow-up among 25 children with confirmed *Giardia intestinalis* infections. Diarrheal stools were tested for *Giardia* infection by immunoassay (Quik CheckTM, TechLab, Inc. Blacksburg, VA) as described in Morris et al. (2018). Horizontal dashed lines mark seropositivity cutoffs for each antigen based on ROC curves of known positive and negative specimens (dashed) or based on Gaussian mixture model (dot dash) as described in the Methods. Children with measurements below the ROC seropositivity cutoff are labeled with color.

```
# ---------------------------
# filter to Giardia antigens
# and to confirmed infections
# ---------------------------
dgi <- dl2 %>%
  filter(pathogen=="Giardia" & stool_giardia==1)

# ---------------------------
# restrict to children with 
# follow-up antibody measurements
# ---------------------------
dgi2 <- dgi %>%
  filter(time=="B") %>%
  mutate(infwk = week-stool_week)

# ---------------------------
# confirm stool measures 
# preceded antibody measures
# ---------------------------
table(dgi2$infwk)
```

```
## 
##  0  3  6  9 10 11 12 13 14 16 17 18 19 20 21 23 28 
##  4  4  2  6  4  2  4  2  2  4  2  4  2  2  4  2  4
```

```
# ---------------------------
# make figure of results
# ---------------------------
log10labs <- c( 
  expression(10^0),
  expression(10^1),
  expression(10^2),
  expression(10^3),
  expression(10^4)
)

pcols <- cbPalette[c(2,3,4,7,8)]
pgiar_time <- ggplot(data=filter(dgi2,!childid %in% c("101","14","37")),aes(x=infwk,y=logmfi)) +
  facet_grid(.~antigenf)+
  geom_hline(aes(yintercept=roccut),linetype="dashed")+
  geom_hline(aes(yintercept=mixcut),linetype="dotdash")+
  geom_point(size=3,pch=21,color="black",fill="black",alpha=0.5)+
  # add children below seropositivity cutoff
  geom_point(data=filter(dgi2,childid %in% c("101","14","37")),
             aes(x=infwk,y=logmfi,fill=childid),
             size=3,pch=21,color="black")+
  scale_x_continuous(breaks=seq(0,28,by=2))+
  scale_y_continuous(breaks=0:4,labels=log10labs)+
  scale_fill_manual(values=pcols)+
  coord_cartesian(xlim=c(0,28),ylim=c(0,4.5)) +
  labs(x="weeks between infection in diarheal stool and antibody measurement",y="IgG Luminex response (MFI-bg)")+
  theme(
    panel.grid.minor.x=element_blank()
  )
pgiar_time
```

```
# save PDF and TIFF versions
ggsave(here::here("figs","asembo-mfi-v-inf-wks-giardia.pdf"),plot=pgiar_time,device=cairo_pdf,width=7,height=4)
ggsave(here::here("figs","asembo-mfi-v-inf-wks-giardia.TIFF"),plot=pgiar_time,device="tiff",width=7,height=4)
```

Most IgG responses remained high irrespective of time since infection over a range of 0 to 28 weeks. As with the longitudinal trajectories, this figure shows the two children (IDs: 101, 37) who had confirmed infections but did not have IgG levels above ROC seropositivity cutoffs for either VSP-3 or VSP-5 at their follow-up measurement 16-23 weeks later. Time since infection does not seem to explain why these three children had low IgG levels, as they are clear outliers from the distribution of the other children.

There appears to be a slight trend toward lower IgG levels with time since infection after approximately 10 weeks. Below, add a locally weighted regression smoother with default bandwidth and approximate pointwise confidence interval to visualize the trend.

```
# ---------------------------
# repeat the figure above, 
# but add a loess smooth and
# approximate pointwise CI
# ---------------------------
pgiar_time2 <- pgiar_time +
  geom_smooth(data=dgi2,method="loess",se=TRUE,color="black",lwd=0.8)

pgiar_time2
```

# Session Info

```
sessionInfo()
```

```
## R version 3.5.3 (2019-03-11)
## Platform: x86_64-apple-darwin15.6.0 (64-bit)
## Running under: macOS High Sierra 10.13.6
## 
## Matrix products: default
## BLAS: /Library/Frameworks/R.framework/Versions/3.5/Resources/lib/libRblas.0.dylib
## LAPACK: /Library/Frameworks/R.framework/Versions/3.5/Resources/lib/libRlapack.dylib
## 
## locale:
## [1] en_US.UTF-8/en_US.UTF-8/en_US.UTF-8/C/en_US.UTF-8/en_US.UTF-8
## 
## attached base packages:
## [1] parallel  stats     graphics  grDevices utils     datasets  methods  
## [8] base     
## 
## other attached packages:
##  [1] doParallel_1.0.11     iterators_1.0.9       foreach_1.4.4        
##  [4] kableExtra_0.8.0.0001 lubridate_1.7.4       forcats_0.3.0        
##  [7] stringr_1.4.0         dplyr_0.8.0.1         purrr_0.3.2          
## [10] readr_1.1.1           tidyr_0.8.3           tibble_2.1.1         
## [13] ggplot2_3.1.1         tidyverse_1.2.1       here_0.1             
## 
## loaded via a namespace (and not attached):
##  [1] tidyselect_0.2.5  xfun_0.6          reshape2_1.4.3   
##  [4] haven_2.1.0       lattice_0.20-38   colorspace_1.3-2 
##  [7] viridisLite_0.3.0 htmltools_0.3.6   yaml_2.2.0       
## [10] rlang_0.3.4       pillar_1.4.0      foreign_0.8-71   
## [13] glue_1.3.1        withr_2.1.2       modelr_0.1.2     
## [16] readxl_1.1.0      plyr_1.8.4        munsell_0.5.0    
## [19] gtable_0.3.0      cellranger_1.1.0  rvest_0.3.2      
## [22] codetools_0.2-16  psych_1.8.4       evaluate_0.13    
## [25] knitr_1.22        highr_0.8         broom_0.4.4      
## [28] Rcpp_1.0.1        backports_1.1.4   scales_1.0.0     
## [31] jsonlite_1.6      mnormt_1.5-5      hms_0.4.2        
## [34] digest_0.6.18     stringi_1.4.3     grid_3.5.3       
## [37] rprojroot_1.3-2   cli_1.1.0         tools_3.5.3      
## [40] magrittr_1.5      lazyeval_0.2.2    crayon_1.3.4     
## [43] pkgconfig_2.0.2   xml2_1.2.0        assertthat_0.2.1 
## [46] rmarkdown_1.12    httr_1.4.0        rstudioapi_0.9.0 
## [49] R6_2.4.0          nlme_3.1-137      compiler_3.5.3
```
