# Supplementary material for: Enteropathogen antibody dynamics and force of infection among children in low-resource settings
Source: eLife. 2019 Aug 19;8:e45594. doi: 10.7554/eLife.45594 (PMC6746552; doi:10.7554/eLife.45594)
Supplement: Supplementary file 5. [file elife-45594-supp5.zip › SIFile5.html]

Enteropathogen antibody dynamics and force of infection among children in low-resource settings


Code 

- Show All Code
- Hide All Code

# Enteropathogen antibody dynamics and force of infection among children in low-resource settings

### Supplementary Information File 5. Sensitivity analysis of fold-change in IgG used to identify incident boosting, incident waning, and presumed unexposed measurements.

# Notebook summary

This notebook includes two sensitivity analyses related to the choice of fold-changes in IgG.

The first sensitivity analysis assessed fold-changes in IgG used to identify boosting and waning. The analysis in the main text used a 4-fold change in IgG, corresponding to a common value used in challenge and immunogenicity studies. The sensitivity analyses repeat the analysis using fold changes that range from 1-10.

The second sensitivy analysis examined different fold-changes used to identify measurements among children who were presumably unexposed before the incident boost. The primary analysis conservatively used a 100-fold increase. Below, we considered increases that ranged from 2- to 316-fold increases and then compared agreement of each choice with classification based on ROC and mixture models where available for each antibody.

Each sensitivity analysis includes a brief written summary and figures to summarize results separately for the Haiti and Kenya cohorts.

# Script preamble

```
#-----------------------------
# preamble
#-----------------------------
library(here)
here::here()
```

```
## [1] "/Users/benarnold/enterics-seroepi"
```

```
library(tidyverse)
library(psych)
```

```
## 
## Attaching package: 'psych'
```

```
## The following objects are masked from 'package:scales':
## 
##     alpha, rescale
```

```
## The following objects are masked from 'package:ggplot2':
## 
##     %+%, alpha
```

```
library(gridExtra)

# set up for parallel computing
# configure for a laptop (use only 3 cores)
library(foreach)
library(doParallel)
registerDoParallel(cores = detectCores() - 1)

# bright color blind palette:  https://personal.sron.nl/~pault/ 
cblack <- "#000004FF"
cblue <- "#3366AA"
cteal <- "#11AA99"
cgreen <- "#66AA55"
cchartr <- "#CCCC55"
cmagent <- "#992288"
cred <- "#EE3333"
corange <- "#EEA722"
cyellow <- "#FFEE33"
cgrey <- "#777777"

# safe color blind palette
# http://jfly.iam.u-tokyo.ac.jp/color/
# http://www.cookbook-r.com/Graphs/Colors_(ggplot2)/
cbPalette <- c("#999999", "#E69F00", "#56B4E9", "#009E73", "#F0E442", "#0072B2", "#D55E00", "#CC79A7")
```

# Sensitivity analysis of fold-changes in IgG

## Summary

The primary analysis used rates of incident seroconversion to estimate force of infection. A secondary analysis used a 4-fold increase in IgG to identify incident boosting. A 4-fold increase is a magnitude of change that vaccine immunogenicity studies often use to define seroconversion and was the basis for our choice. The use of a 4-fold increase is somewhat arbitrary in this context, though there is some justification of that magnitude of IgG increase to define seroconversion based on enteric pathogen challenge studies (e.g., Moe et al. 2004 for norovirus https://www.ncbi.nlm.nih.gov/pubmed/15539501).

In this sensitivity analysis, we examined the influence of the assumed fold-change in IgG on seroconversion and seroreversion rates in the Haiti and Kenya cohorts where there were longitudinal measurements among enrolled children. In all cases, seroconversion was defined as a fold-increase in IgG to above a seropositivity cutoff, and seroreversion was defined as a fold-decrease in IgG from above a seropositivity cutoff. We examined from 1 to 10-fold changes. All estimates include bootstrapped 95% confidence intervals, as in the primary analyses.

The code below is a bit opaque because we have not had time to streamline it. It essentially repeats the workflow used in the main analysis scripts `Table2-haiti-incidence-analysis.Rmd` and `Fig6-asembo-incidence-analysis.Rmd` for each X-fold boosting or waning scenario. Since changing the fold-change definition requires re-creating an incidence dataset, combining information temporally across multiple antibody responses for some pathogens, each simulation replicate is a bit involved.

In Haiti, where children were measured from ages 0-11 years old over a median of 5-years, the results of the sensitivity analysis show that rates of boosting and waning go down as the fold-increase in IgG criterion increases. Across pathogens boosting rates stabilized around a 3-fold to 6-fold increase. Rates of incident waning were generally higher across fold-reductions in IgG compared with the seroreversion rate.

In the Kenya cohort, rates of incident boosting were insensitive to the assumption of fold-change in IgG, presumably because most increases in IgG were large, reflecting a primary infection for most children. Rates of incident waning declined and stabilized around a 4-fold decrease.

The sensitivity analyses support the use of a 4-fold change in IgG criterion to identify incident boosting. At a 4-fold increase, the only pathogen for which the incident boosting rate was significantly different from the seroconversion rate was *Cryptosporidium* in Haiti.

## Haiti

```
#-----------------------------
# load the formatted data
# created with 
# haiti-enteric-ab-data-format.Rmd ->
# Fig1-haiti-ab-distributions.Rmd
#-----------------------------
dl <- readRDS(here::here("data","haiti_analysis2.rds"))

#-----------------------------
# identify seropositive measures
# hierarchy of information for 
# cutoffs:
# 1. ROC
# 2. mixture model based
# 3. estimated among presumed unexposed
#
# store the cutoff value used
# for figures
#-----------------------------
dl <- dl %>%
  mutate(seropos=ifelse(!is.na(posroc),posroc,posmix),
         serocut=ifelse(!is.na(posroc),roccut,mixcut),
         serocut_desc=ifelse(!is.na(posroc),"ROC","Mixture Model")) %>%
  mutate(seropos=ifelse(!is.na(seropos),seropos,posunex),
         serocut_desc=ifelse(!is.na(serocut),serocut_desc,"Unexp dist"),
         serocut=ifelse(!is.na(serocut),serocut,unexpcut))
```

```
#-----------------------------
# loop over pathogens and
# fold-changes in IgG
#-----------------------------
dsens <- dl %>%
  group_by(antigenf,id) %>%
  arrange(antigenf,id,age) %>%
  mutate(nobs=n(),obsnum=row_number(),
         agediff=ifelse(obsnum==1,lead(age)-age,age-lag(age)),
         logmfi_lag  = lag(logmfi),
         logmfi_dlag  = logmfi - logmfi_lag
  ) %>%
  # Keep Norovirus separate as a pathogen
  mutate(pathogen2=as.character(pathogen),
         pathogen2=ifelse(antigenf=="Norovirus GI.4","Norovirus GI.4",pathogen2),
         pathogen2=ifelse(antigenf=="Norovirus GII.4.NO","Norovirus GII.4.NO",pathogen2)) %>%
  select(-pathogen) %>% rename(pathogen=pathogen2)


#-----------------------------
# loop over pathogens and
# fold-changes in IgG
# 
# Note: there are 7x41x1000=287,000
# bootstrap replicates in this
# so even though the estimator
# is simple it still takes a few mins
# because it has to re-create
# the incident dataset 637 times
#-----------------------------
set.seed(12)
dsens_res <- foreach(ab=unique(dsens$pathogen),.combine=rbind) %:%
  foreach(Xfold=seq(2,10,by=0.2),.combine=rbind) %dopar% {
    
    #-----------------------------
    # Identify incident boosting/waning 
    # of X-fold MFI or more. For
    # incident boosting, +X-fold increase
    # that ends above seropositivity cutoff
    # for incident waning, -X-fold decrease
    # that starts above seropositivity cutoff
    #-----------------------------
    di <- dsens %>%
      filter(pathogen==ab) %>%
      group_by(antigenf,id) %>%
      arrange(antigenf,id,obsnum) %>%
      mutate(seroi = ifelse(logmfi_dlag>log10(Xfold) & logmfi>serocut,1,0),
             seroi = ifelse(is.na(logmfi_dlag),NA,seroi),
             seror = ifelse(logmfi_dlag< -log10(Xfold) & logmfi_lag>serocut,1,0)
      ) %>%
      ungroup()
    
    #-----------------------------
    # create composite 
    # seropositivity, boosting,
    # and waning indicators
    # that use information from
    # multiple antigens if avail
    #-----------------------------
    di2 <- di %>%
      group_by(pathogen,id,obsnum) %>%
      # since incidence could fall at slightly different times across the antigens
      # for the same pathogen, use the earlier onset as the incidence measure
      arrange(pathogen,id,obsnum) %>%
      group_by(pathogen,id) %>%
      mutate(lagseroi=lag(seroi),
             seroi=ifelse(seroi==1 & lagseroi==1 & !is.na(lagseroi),0,seroi)) %>%
      # since incidence could fall at slightly different times across the antigens
      # for the same pathogen, use the later onset as the incident measure
      mutate(leadseror=lead(seror),
             seror=ifelse(seror==1 & leadseror==1 & !is.na(leadseror),0,seror)) %>%
      # now get the max for each pathogen at each timepoint
      group_by(pathogen,id,obsnum) %>%
      mutate(seropos=max(seropos),
             seroi=max(seroi),
             seror=max(seror)
      ) %>%
      slice(1) %>%
      select(pathogen,id,obsnum,agediff,seropos,seroi,seror) %>%
      # in unusual cases for multiple-antigen pathogens some children could in theory
      # boost in one antigen and wane in another. In the analysis of 4-fold changes
      # this occurred 7 times across >4,000 incident periods among
      # children with Cryptosporidium Cp17 and Cp23 antigens. In these cases,
      # recode the incident waning to 0 (assumption is that they had incident boosting)
      mutate(seror=ifelse(seroi==1 & seror==1,0,seror))
    
    #-----------------------------
    # estimate time at risk
    # for incident boosting and waning
    # assumed to be 1/2 of time
    # between measurements
    # if indivs change serostatus
    # 
    # create indicators of at risk
    # based on incidence boosting
    # and waning
    # at risk if seronegative OR experienced
    # a X-fold decrease in MFI in previous period
    #
    # the below code is opaque, but confirmed through 
    # extensive vetting and inspection that it does this:
    # children are at risk of boosting if they are seronegative or
    # if they experience a X-fold decrease in MFI between the previous
    # two measurements
    #-----------------------------
    di3 <- di2 %>%
      group_by(id) %>%
      mutate(seroi=ifelse(obsnum==1,0,seroi),
             seror=ifelse(obsnum==1,0,seror),
             maxobs=max(obsnum),
             # seronegative at obs 1: at risk
             # seropositive at obs 1: not at risk
             cntr = 0,
             cntr = ifelse(seroi==1 & lag(seroi)==0,-1,0), # counter -1 if first boost X-fold
             cntr = ifelse(obsnum==1 & seropos==0,1,cntr), # counter +1 if seroneg & obs1
             cntr = ifelse(seror==1,1,cntr), # counter +1 if seror
             cntr = ifelse(seror==1 & seropos==0 & lag(cntr)==1,cntr-1,cntr),
             
             # sum counter to help with recodes below
             cntrs = cumsum(cntr),
             
             cntr = ifelse(obsnum!=maxobs & lead(cntr)==-1 & lead(cntrs)==-1,1,cntr),
             cntr = ifelse(obsnum!=maxobs & lead(cntr)==-1 & lead(cntrs)==-2,1,cntr),
             cntr = ifelse(seror==1 & cntrs==2,0,cntr),
             
             # indicator of whether a chlid is at risk for incident boosting
             atrisk = cumsum(cntr),
             atrisk = lag(atrisk),
             
             # fix a very small number of idiosyncratic situations
             # that don't fit neatly into the above algorithm (n=5)
             atrisk = ifelse(obsnum==1,0,atrisk),
             atrisk = ifelse(obsnum > 2 & atrisk==2 | lag(atrisk==2),atrisk-1,atrisk),
             
             # now calculate person time at risk for boosting and waning
             atriskc = atrisk,
             atriskr = 1-atrisk,
             ptc = ifelse(atriskc==1,agediff,0),
             ptc = ifelse(atriskc==1 & seroi==1,agediff/2,ptc),
             ptr = ifelse(atriskr==1,agediff,0),
             ptr = ifelse(atriskr==1 & seror==1,agediff/2,ptr)
             
      )
    
    
    #-----------------------------
    # estimate incidence rates
    # for each antibody
    # units are episodes per child-year
    #-----------------------------
    dsensi_rates <- di3 %>% 
      ungroup() %>%
      summarize(ni=sum(seroi,na.rm=T),
                nit=sum(ptc,na.rm=T),
                nr=sum(seror,na.rm=T),
                nrt=sum(ptr,na.rm=T)) %>%
      mutate(seroi=ni/nit,seror=nr/nrt)
    
    #-----------------------------
    # get bootstrap CIs
    # resampling children with 
    # replacement due to repeated
    # measures
    #-----------------------------
    dboot <- di3 %>%
      ungroup() %>%
      select(id,seroi,seror,ptc,ptr)
    
    nreps <- 1000
    ids <- unique(dboot$id)
    bsamp <- matrix(sample(ids,size=length(ids)*nreps,replace=TRUE),
                    nrow=length(ids),ncol=nreps)
    dsens_bootests <- matrix(NA,nrow=nreps,ncol=2)
    i <- 1
    while(i <= nreps) {
      bi <- left_join(data.frame(id=bsamp[,i]),dboot,by=c("id")) %>%
        select(-id) %>% 
        summarize_all(function(x) sum(x,na.rm=TRUE))
      dsens_bootests[i,] <- c(bi$seroi/bi$ptc, bi$seror/bi$ptr)
      i <- i+1
    }
    
    dsensi_cis <- data.frame(bseroi=dsens_bootests[,1],bseror=dsens_bootests[,2]) %>% 
      # restrict to finite values (a few ETEC bootstrap replicates have a denominator of 0)
      summarize(seroi_lb = quantile(bseroi[is.finite(bseroi)],probs=c(0.025),na.rm=T),
                seroi_ub = quantile(bseroi[is.finite(bseroi)],probs=c(0.975),na.rm=T),
                seror_lb = quantile(bseror[is.finite(bseror)],probs=c(0.025),na.rm=T),
                seror_ub = quantile(bseror[is.finite(bseror)],probs=c(0.975),na.rm=T)
      )
    
    # post results
    dsensi_res <- bind_cols(dsensi_rates,dsensi_cis) %>%
      mutate(pathogen=ab,foldchange=Xfold) %>%
      select(pathogen,foldchange,ni,nit,starts_with("seroi"),nr,nrt,starts_with("seror")) 
    dsensi_res
    

  }    

# reshape the results for ggplot
dsens_res2 <- dsens_res %>%
  mutate(antigenf = case_when(
    pathogen == "Giardia" ~ "Giardia VSP-3 or VSP-5",
    pathogen == "Cryptosporidium" ~ "Cryptosporidium Cp17 or Cp23",
    pathogen == "E. histolytica" ~ "E. histolytica LecA",
    pathogen == "Salmonella" ~ "Salmonella LPS groups B or D",
    pathogen == "ETEC" ~ "ETEC LT B subunit",
    pathogen == "Norovirus GI.4" ~ "Norovirus GI.4",
    pathogen == "Norovirus GII.4.NO" ~ "Norovirus GII.4.NO"),
    antigenf=factor(antigenf,levels=c("Giardia VSP-3 or VSP-5",
                                  "Cryptosporidium Cp17 or Cp23",
                                  "E. histolytica LecA",
                                  "Salmonella LPS groups B or D",
                                  "ETEC LT B subunit",
                                  "Norovirus GI.4",
                                  "Norovirus GII.4.NO"))
  )
dsens_res_scr <- dsens_res2 %>%
  select(antigenf,foldchange,starts_with("seroi")) %>%
  mutate(outcome="incident boosting") %>%
  rename(rate=seroi,rate_lb=seroi_lb,rate_ub=seroi_ub)
dsens_res_srr <- dsens_res2 %>%
  select(antigenf,foldchange,starts_with("seror")) %>%
  mutate(outcome="incident waning") %>%
  rename(rate=seror,rate_lb=seror_lb,rate_ub=seror_ub)
dsens_plot <- bind_rows(dsens_res_scr,dsens_res_srr)
```

**Figure caption:** Estimates of prospective rates of incident boosting and waning by pathogen across a range of fold-changes in IgG used to define a change in serostatus. Estimates derived from 142 children ages 0-11 years in Leogane, Haiti measured prospectively over a median of 5 years, 1990-1999. Shaded regions indicate bootstrapped 95% confidence intervals. Horizontal dashed lines mark each pathogen’s seroconversion rate (boosting panels) or seroreversion rate (waning panels) estimated in the cohort using seropositivity cutoffs. Vertical dashed lines mark a 4-fold increase in IgG, commonly used to define seroconversion in challenge studies and vaccine immunogenicity studies.

```
# color palette
pcols <- cbPalette[c(2,6)]

# load Haiti seroconversion and seroreversion rate estimates 
# from the primary analysis for a comparison
# created by Table2-haiti-incidence-analysis.Rmd
rate_ests <- readRDS(file=here::here("output","haiti-enteric-ab-ests-incidencerates.rds"))
scr_plot <- rate_ests %>% mutate(outcome="incident boosting")
srr_plot <- rate_ests %>% mutate(outcome="incident waning")

dsensp <- ggplot(data=dsens_plot,aes(x=foldchange,color=outcome,fill=outcome))+
  # facet_wrap(~antigenf,ncol=2,nrow=4)+
  facet_grid(antigenf~outcome)+
  geom_vline(xintercept = 4,alpha=0.5,linetype="dashed")+
  geom_hline(data=scr_plot,aes(yintercept=seroi),linetype="dashed",alpha=0.5)+
  geom_hline(data=srr_plot,aes(yintercept=seror),linetype="dashed",alpha=0.5)+
  geom_ribbon(aes(ymin=rate_lb,ymax=rate_ub),alpha=0.2,color=NA)+
  geom_line(aes(y=rate))+
  
  scale_color_manual(values=pcols)+
  scale_fill_manual(values=pcols)+
  scale_x_continuous(breaks=2:10)+
  labs(x="fold change in IgG used to define change in serostatus",y="rate per child-year")+
  coord_cartesian(ylim=c(0,2),x=c(2,10))+
  theme_minimal() +
  theme(
    legend.position="none",
    legend.title=element_blank(),
    strip.text=element_text(hjust=0),
    strip.text.y=element_text(angle=0)
  )

dsensp
```

The results show that incident boosting rate (force of infection) estimates were relatively insensitive to choice of fold-change in IgG in this study, particularly over a range of 3-6 fold changes. In general, incident boosting rates declined with more stringent fold-change criteria. At a 4-fold increase, the only pathogen for which the incident boosting rate was significantly different from the seroconversion rate was *Cryptosporidium*.

## Kenya

```
#-----------------------------
# load the formatted data
# created with 
# asembo-enteric-ab-data-format.Rmd -->
# Fig1sup3-asembo-ab-distributions.Rmd
#-----------------------------
dl <- readRDS(here::here("data","asembo_analysis2.rds"))
```

```
#-----------------------------
# restrict to children measured 
# longitudinally
# exclude cholera CTB because
# it cannot be differentiated 
# from ETEC LTB (per main analysis)
# store MFI at each time point
# A or B
#-----------------------------
dsens <- dl %>%
  ungroup() %>% 
  filter(pathogen!="V. cholerae") %>%
  mutate(pathogen=droplevels(pathogen)) %>%
  group_by(antigenf,childid) %>%
  mutate(nobs=n()) %>%
  filter(nobs>1) %>% 
  mutate(mfiA=ifelse(time=="A",logmfi,NA), mfiA=max(mfiA,na.rm=TRUE),
         mfiB=ifelse(time=="B",logmfi,NA), mfiB=max(mfiB,na.rm=TRUE)
  ) %>%
  filter(time=="A")%>%
  select(pathogen,antigen,antigenf,childid,age,agediff,logmfidiff,serocut,mfiA,mfiB) %>%
  ungroup() %>%
  group_by(pathogen,childid) # group by pathogen and child for aggregating info over antigens in sens analysis


#-----------------------------
# loop over pathogens and
# fold-changes in IgG
#-----------------------------
set.seed(12)
dsens_res <- foreach(ab=levels(dsens$pathogen),.combine=rbind) %:%
  foreach(deltafold=seq(2,10,by=0.2),.combine=rbind) %dopar% {
    
    #-----------------------------
    # identify incident 
    # seroconversions and reversions
    # based on a fold change in MFI
    # to above the cutoff (seroconversion)
    # or starting above cutoff (seroreversion)
    # aggregate information over
    # multiple antigens if available
    #-----------------------------
    dsensi <- dsens %>%
      filter(pathogen==ab) %>%
      mutate(seroi=ifelse(logmfidiff >   log10(deltafold) & mfiB>serocut,1,0),
             seror=ifelse(logmfidiff < - log10(deltafold) & mfiA>serocut,1,0)
      ) %>%
      mutate(seroi=max(seroi),
             seror=max(seror)
      ) %>%
      slice(1)
    
    #-----------------------------
    # estimate time at risk
    # for seroconversion and reversion
    # assumed to be 1/2 of time
    # between measurements
    # if indivs are seropositive
    # at measurement 1 they are
    # not at risk for seroconversion
    # (and vice-versa for seroreversion)
    #-----------------------------
    dsensi <- dsensi %>%
      mutate(ptc = ifelse(mfiA<serocut,agediff,0),
             ptc = ifelse(seroi==1,agediff/2,ptc),
             ptr = ifelse(mfiA>serocut,agediff,0),
             ptr = ifelse(seror==1,agediff/2,ptr)
      )
    
    #-----------------------------
    # estimate seroconversion
    # and seroreversion rates
    # for each pathogen
    # units are episodes per child-year
    # (dividing person time in months by 12)
    #-----------------------------
    dsensi_ests <- dsensi %>%
      group_by(pathogen) %>%
      summarize(ni=sum(seroi,na.rm=T),
                nit=sum(ptc,na.rm=T)/12,
                nr=sum(seror,na.rm=T),
                nrt=sum(ptr,na.rm=T)/12) %>%
      mutate(scr=ni/nit,
             srr=nr/nrt)
    
    #-----------------------------
    # estimate bootstrap CIs
    #-----------------------------
    dsens_boot <- dsensi %>%
      group_by(pathogen) %>%
      select(pathogen,seroi,seror,ptc,ptr)
    nboot <- 1000
    dsens_bootests <- matrix(NA,nrow=nboot,ncol=2)
    i <- 1
    while(i <= nboot) {
      di <- sample_frac(dsens_boot,size=1,replace=TRUE) %>%
        summarize_all(function(x) sum(x,na.rm=TRUE))
      dsens_bootests[i,] <- c(di$seroi/(di$ptc/12), di$seror/(di$ptr/12))
      i <- i+1
    }
    dsens_bootests <- data.frame(pathogen=ab,scr=dsens_bootests[,1],srr=dsens_bootests[,2])
    dsensi_cis <- dsens_bootests %>% 
      group_by(pathogen) %>%
      summarize(scr_lb = quantile(scr,probs=c(0.025),na.rm=T),
                scr_ub = quantile(scr,probs=c(0.975),na.rm=T),
                srr_lb = quantile(srr,probs=c(0.025),na.rm=T),
                srr_ub = quantile(srr,probs=c(0.975),na.rm=T)
      )
    dsensi_res <- left_join(dsensi_ests,dsensi_cis,by="pathogen") %>%
      mutate(foldchange=deltafold) %>%
      select(pathogen,foldchange,ni,nit,starts_with("scr"),nr,nrt,starts_with("srr")) 
    dsensi_res
    }
    
# reshape the results for ggplot
dsens_res2 <- dsens_res %>%
  mutate(antigenf = case_when(
    pathogen == "Giardia" ~ "Giardia VSP-3 or VSP-5",
    pathogen == "Cryptosporidium" ~ "Cryptosporidium Cp17 or Cp23",
    pathogen == "E. histolytica" ~ "E. histolytica LecA",
    pathogen == "Salmonella" ~ "Salmonella LPS groups B or D",
    pathogen == "ETEC" ~ "ETEC LT B subunit",
    pathogen == "Campylobacter" ~ "Campylobacter p18 or p39"),
    antigenf = factor(antigenf,levels=c("Giardia VSP-3 or VSP-5",
                                  "Cryptosporidium Cp17 or Cp23",
                                   "E. histolytica LecA",
                                  "Salmonella LPS groups B or D",
                                  "ETEC LT B subunit",
                                  "Campylobacter p18 or p39")),
    antigenf = droplevels(antigenf)
  )
dsens_res_scr <- dsens_res2 %>%
  select(antigenf,foldchange,starts_with("scr")) %>%
  mutate(outcome="incident boosting") %>%
  rename(rate=scr,rate_lb=scr_lb,rate_ub=scr_ub)
dsens_res_srr <- dsens_res2 %>%
  select(antigenf,foldchange,starts_with("srr")) %>%
  mutate(outcome="incident waning") %>%
  rename(rate=srr,rate_lb=srr_lb,rate_ub=srr_ub)
dsens_plot <- bind_rows(dsens_res_scr,dsens_res_srr)
```

**Figure caption:** Estimates of prospective rates of incident boosting and waning by pathogen across a range of fold-changes in IgG used to define a change in serostatus. Estimates derived from 199 children ages 4-18 months in Asembo, Kenya measured prospectively over a 6-month period in 2013. Shaded regions indicate bootstrapped 95% confidence intervals. Horizontal dashed lines mark each pathogen’s seroconversion rate (boosting panels) or seroreversion rate (waning panels) estimated in the cohort using seropositivity cutoffs. Vertical dashed lines mark a 4-fold increase in IgG, commonly used to define seroconversion in challenge studies and vaccine immunogenicity studies.

```
# color palette
pcols <- cbPalette[c(2,6)]

# load Haiti seroconversion and seroreversion rate estimates 
# from the primary analysis for a comparison
# created by Fig6-asembo-incidence-analysis.Rmd
rate_ests <- readRDS(file=here::here("output","asembo-enteric-ab-ests-incidencerates.rds"))
scr_plot <- rate_ests %>% 
  filter(outcome=="seroconversion rate (force of infection)") %>% 
  mutate(outcome="incident boosting") %>% 
  filter(antigen!="cholera")
srr_plot <- rate_ests %>% 
  filter(outcome=="seroreversion rate") %>%
  mutate(outcome="incident waning") %>% 
  filter(antigen!="cholera")

dsensp <- ggplot(data=dsens_plot,aes(x=foldchange,color=outcome,fill=outcome))+
  facet_grid(antigenf~outcome)+
  geom_vline(xintercept = 4,alpha=0.5,linetype="dashed")+
  geom_hline(data=scr_plot,aes(yintercept=rate),linetype="dashed",alpha=0.5)+
  geom_hline(data=srr_plot,aes(yintercept=rate),linetype="dashed",alpha=0.5)+  
  geom_ribbon(aes(ymin=rate_lb,ymax=rate_ub),alpha=0.2,color=NA)+
  geom_line(aes(y=rate))+
  scale_color_manual(values=pcols)+
  scale_fill_manual(values=pcols)+
  scale_x_continuous(breaks=2:10)+
  labs(x="fold change in IgG used to define change in serostatus",y="rate per child-year")+
  coord_cartesian(ylim=c(0,4),x=c(2,10))+
  theme_minimal() +
  theme(
    legend.position = "none",
    legend.title=element_blank(),
    strip.text=element_text(hjust=0),
    strip.text.y=element_text(angle=0)
  )

dsensp
```

The results show that incident boosting rate (force of infection) estimates were insensitive to choice of fold-change in IgG in this study, and that incident waning rates stabilized for *Giardia* and *Salmonella* at approximately a 4-fold decrease. The incident waning rate for *E. histolytica* continued to decline until a 6.5 fold decrease in IgG, but events were rare for this pathogen with at most 6 waning events across the 199 children at the least stringent change assessed (1-fold decrease).

# Fold-change in IgG used to identify presumed unexposed

## Summary

### Identifying presumed seroconverters

One approach to identifying a seropositivity cutoff in a longitudinal study is to identify children who presumably seroconvert from negative to positive based on a change in their antibody levels, and then use the distribution of their antibody levels before seroconversion to identify a seropositivity cutoff. The rationale for this two-step process that estimates a cutoff (and does not simply classify children as seroconverers versus not) is that the cutoff can then be applied to identify children who were seropositive but did not actually seroconvert during the study. This was common for antibody response to enteric pathogens in the study cohorts, where exposure ocurred frequently and very early in life.

### Level of increase in antibody levels to identify seroconversion

The method relies on choosing the appropriate magnitude of change in antibody levels used identify presumed seroconverters, which is generally unknown. Most immunogenicity abnd pathogen challenge studies have used a 4-fold increase. In this study, we used an increase of +2 or more on the log\(\_{10}\) scale, equal to a 100-fold increase, to identify presumed seroconverters. Although this was likely a conservative assumption, it still led to high levels of agreement with other classification approachs (generally >95%) when applied across cohorts and antibodies.

### Sensitivity analysis description

Here, we conduct a sensitivity analysis that searches over a range of increases in antibody levels used to identify “presumed seroconverters”. For each increase, we then calculated the mean and SD of the distribution before the change, and used that as a seropositivity cutoff. We then compared the classification accuracy of each value against classifications based on cutoffs derived from ROC-curves or mixture models, as available.

In the present study, we used two additional methods to determine seropositivity cutoffs:

- For *Giardia* (VSP-3, VSP-5), *Cryptosporidium* (Cp17, Cp23), and *E. histolytica* (LecA) antigens, known negative and positive samples were used to identify an optimal cutoff based on a receiver-operator characteristic (ROC) curve.
- For all other pathogens, we fit a 2-component, finite Gaussian mixture model to the antibody distributions, and then used the lower component’s mean plus 3 standard deviations to define a cutoff value. This identified reasonable cutoff values for many, but not all, additional antibodies. In some cases the mixture model based approach did not identify reasonable cutoff values (i.e., near or above the upper limit assay’s dynamic range).

Given this other classification information, we compared a range of changes in antibody levels used to identify children who presumably seroconverted in terms of agreement with ROC-based and mixture model-based classification of seropositivity.

We searched over a range of changes from +0.3 to +2.5 on the log10 MFI-bg scale (this corresponds to a 10^0.3 = 2-fold to 10^2.5 = 316-fold increase in antibody levels). For each change in antibody levels (0.3 to 2.5), we identified the children who would be identified as seroconverters. From these children, we then estimated the mean plus 3 standard deviations of their antibody levels at their first measurement, when they were presumably unexposed. This is the seropositivity cutoff among the “presumed unexposed”. We then estimated agreement and Cohen’s Kappa statistics for each change and identify the magnitude of change that corresponded with the maximum level of agreement.

### Sensitivity analysis results

The optimal change in MFI levels used to identify presumed seroconverters varied by cohort and antigen, where “optimal” was defined as the minimum change in MFI with maximum agreement with respect to ROC-based or mixture model-based seropositivity classifications.

For antigens with both ROC-based cutoffs and mixture model-based cutoffs, results were similar between the two comparisons. This result reflects good agreement between ROC- and mixture model-based cutoffs (detailed results in this article’s Supplementary Information File 2).

Over the range of values considered, the method led to cutoff values with classification agreement that was very high compared with both ROC- and mixture model-based classifications. In Haiti, optimal agreement exceeded 0.92 for all comparisons. In Kenya, optimal agreement exceeded 0.98 for all comparisons.

In Haiti, the change in MFI values that led to highest agreement were generally \(\geq 2\) on the log\(\_{10}\) scale. In Kenya, change values were lower and generally in the range of 0.5 to 1.5. In general, across both countries, there was not a large decline in agreement at higher change values imposed to identify presumed seroconverters when developing a cutoff (an exception was *Cryptosporidium* Cp23 in Kenya).

Taken together, this sensitivity analysis supports the use of an increase of +2 log\(\_{10}\) MFI to identify presumed seroconverters in the definition of seropositivity cutoffs for antigens considered.

## Haiti

```
#-----------------------------
# load the formatted data
# created with 
# haiti-enteric-ab-data-format.Rmd ->
# haiti-enteric-ab-distributions.Rmd
#-----------------------------
dl <- readRDS(here::here("data","haiti_analysis2.rds"))

# list the enteric antigens and formatted labels for them
mbavars <- c("vsp3","vsp5","cp17","cp23","leca","salb","sald","etec","norogi","norogii")

mbalabs <- c("Giardia VSP-3","Giardia VSP-5","Cryptosporidium Cp17","Cryptosporidium Cp23","E. histolytica LecA","Salmonella LPS group B","Salmonella LPS group D","ETEC LT B subunit","Norovirus GI.4", "Norovirus GII.4.NO")
```

### Sensitivity analysis over fold-change values, ROC-based reference

*Giardia*, *Cryptosporidium*, and *E. histolytica* antigens have ROC-based cutoff values in this study. For these antigens, search over fold-change values to determine the cutoff with best agreement with ROC-based classification.

```
droc <- dl %>%
  filter(!is.na(roccut)) %>%
  droplevels()

gridroc <- foreach(ab=levels(droc$antigenf),.combine=rbind) %:%
  foreach(icut=seq(0.3,2.5,by=0.01),.combine=rbind) %dopar% {
    
    # downsample to measurements among children <=1 y 
    # who increased by the increment icut in the next period    
    # these are "presumed unexposed" under this cutoff in change icut
    # estimate mean and SD among the presumed unexposed to use as a 
    # seropositivity cutoff
    dmu <- droc %>% 
      ungroup() %>%
      select(antigenf,age,logmfi,logmfidiff) %>%
      filter(antigenf==ab & age <= 1 & logmfidiff>icut) %>%
      summarize(mu=mean(logmfi),sd=sd(logmfi),ucut=mu+3*sd)
    
    # classify all observations using the derived cutoff
    di <- droc %>%  
      filter(antigenf==ab) %>%
      mutate(posi=factor(ifelse(logmfi>dmu$ucut,1,0),levels=c(0,1)))
    
    # estimate agreement
    tabi <- as.vector(table(as.factor(di$posroc),as.factor(di$posi)))
    names(tabi) <- c("roc0cuti0","roc1cuti0","roc0cuti1","roc1cuti1")
    kappai <- psych::cohen.kappa(table(di$posroc,di$posi))$kappa
    tabi <- data.frame(t(tabi))
    tabi <- tabi %>%
      mutate(Nobs=roc0cuti0+roc1cuti0+roc0cuti1+roc1cuti1,
             agreement=(roc0cuti0+roc1cuti1)/Nobs,
             kappa=kappai,
             antigenf=ab,
             abchange=icut,
             abcutoff=dmu$ucut) 
    
  }

gridroc <- gridroc %>% mutate(antigenf=factor(antigenf,levels=levels(droc$antigenf)))

# identify the optimal change based on % agreement, take minimum change
optchgroc <- gridroc %>%
  group_by(antigenf) %>%
  mutate(maxagr=max(agreement,na.rm=TRUE),
         maxchg=ifelse( abs(agreement-maxagr)<0.001,1,0)) %>%
  filter(maxchg==1) %>%
  arrange(antigenf,abchange) %>%
  filter(row_number()==1) %>%
  select(antigenf,optchg=abchange,optagr=agreement,optkap=kappa,optcut=abcutoff)

# merge in the optimal change values
gridroc <- left_join(gridroc,optchgroc,by="antigenf")  %>%
  group_by(antigenf)
```

```
# this general figure scheme is specific to results stored in
# gridroc and gridmix objects.

plot_optchange <- function(results,classlab="XXX reference standard") {
  
  nantigens <- length(levels(results$antigenf))
  
  # Figure of cutoff by antibody change threshold
  plot_cutoff <- ggplot(data=results,aes(x=abchange)) +
  geom_line(aes(y=abcutoff),color=cmagent) +
  geom_vline(aes(xintercept=optchg),lty="dashed")+ 
  geom_hline(aes(yintercept=optcut),lty="dashed")+ 
  # labels for optimal change and corresponding cutoff based on % agreement
  geom_label(data=filter(results,row_number()==1),
            aes(x=optchg,y=0.5,label=sprintf("%1.2f",optchg)),
            position=position_nudge(x=0),
            fill="white",label.size=0) + 
  geom_label(data=filter(results,row_number()==1),
            aes(y=optcut,x=0,label=sprintf("%1.2f",optcut)),
            position=position_nudge(y=0,x=0.4),
            fill="white",label.size=0) + 
  facet_wrap(~antigenf,nrow=1,ncol=nantigens) +
  # aesthetics
  scale_y_continuous(breaks=seq(0.5,4,by=0.5))+
  scale_x_continuous(breaks=seq(0,3,by=0.5))+
  coord_cartesian(ylim=c(0.5,4),xlim=c(0,3)) +
  labs(x="log10 change in MFI used to identify presumed seroconverters",y="Estimated seropositivity cutoff\n(log10 MFI-bg)") +
  theme_minimal()
  
  # Figure of Kappa by antibody change threshold
  plot_kappa <- ggplot(data=results,aes(x=abchange)) +
  # geom_line(aes(y=agreement),color=cgreen) +
  geom_line(aes(y=kappa),color=cblue)+
  geom_vline(aes(xintercept=optchg),lty="dashed")+ 
  geom_hline(aes(yintercept=optkap),lty="dashed")+ 
  # labels for optimal change and corresponding kappa based on % agreement
  geom_label(data=filter(results,row_number()==1),
            aes(x=optchg,y=0,label=sprintf("%1.2f",optchg)),
            position=position_nudge(x=0),
            fill="white",label.size=0) + 
  geom_label(data=filter(results,row_number()==1),
            aes(y=optkap,x=0,label=sprintf("%1.2f",optkap)),
            position=position_nudge(y=0,x=0.4),
            fill="white",label.size=0) + 
  facet_wrap(~antigenf,nrow=1,ncol=nantigens) +
  # aesthetics
  scale_y_continuous(breaks=seq(0,1,by=0.1))+
  scale_x_continuous(breaks=seq(0,3,by=0.5))+
  coord_cartesian(ylim=c(0,1),xlim=c(0,3)) +  
  labs(x="",y=paste("Cohen's Kappa,\n",classlab)) +
  theme_minimal()
  
  # Figure of Agreement by antibody change threshold
  plot_agree <- ggplot(data=results,aes(x=abchange)) +
  geom_line(aes(y=agreement),color=cgreen) +
  # geom_line(aes(y=kappa),color=corange)+
  geom_vline(aes(xintercept=optchg),lty="dashed")+ 
  geom_hline(aes(yintercept=optagr),lty="dashed")+ 
  # labels for optimal change and agreement based on % agreement
  geom_label(data=filter(results,row_number()==1),
            aes(x=optchg,y=0,label=sprintf("%1.2f",optchg)),
            position=position_nudge(x=0),
            fill="white",label.size=0) + 
  geom_label(data=filter(results,row_number()==1),
            aes(y=optagr,x=0,label=sprintf("%1.2f",optagr)),
            position=position_nudge(y=0,x=0.4),
            fill="white",label.size=0) + 
  facet_wrap(~antigenf,nrow=1,ncol=nantigens) +
  # aesthetics
  scale_y_continuous(breaks=seq(0,1,by=0.1))+
  scale_x_continuous(breaks=seq(0,3,by=0.5))+
  coord_cartesian(ylim=c(0,1),xlim=c(0,3)) +
  labs(x="",y=paste("Agreement,\n",classlab)) +
  theme_minimal(base_size=9)
  
  # stack the figures into a single panel
  grid.arrange(plot_agree,plot_kappa,plot_cutoff)

}
```

The bottom panel plots the estimated seropositivity cutoff as a function of change in MFI levels used to identify presumed seroconverters. The mean plus 3 SDs of the distribution of antibody levels before presumed seroconversion was used to estimate the cutoff. For a given cutoff, the middle and top panels summarize classification agreement with the ROC-based seropositiity cutoff based on Cohen’s Kappa (middle) and proportion agreement (top).

Labeled dashed lines indicate the minimum magnitude of change in antibody levels that led to highest agreement, along with the corresponding seropositivity cutoff values, Cohen’s Kappa, and agreement.

```
plot_optchange(results=gridroc,classlab="ROC-based classification")
```

### Sensitivity analysis over fold-change values, mixture model-based reference

Search over fold-change values to determine the cutoff with best agreement with mixture model-based classification

```
dmix <- dl %>%
  filter(!is.na(mixcut)) %>%
  droplevels()

gridmix <- foreach(ab=levels(dmix$antigenf),.combine=rbind) %:%
  foreach(icut=seq(0.3,2.5,by=0.01),.combine=rbind) %dopar% {

    # downsample to measurements among children <=1 y 
    # who increased by the increment icut in the next period
    # these are "presumed unexposed" under this cutoff in change icut
    # estimate mean and SD among the presumed unexposed to use as a 
    # seropositivity cutoff
    dmu <- dmix %>% 
      ungroup() %>%
      select(antigenf,age,logmfi,logmfidiff) %>%
      filter(antigenf==ab & age <=1 & logmfidiff>icut)
    
    # if there are <2 obs, cannot estimate mean + SD, so skip
    if(nrow(dmu)>=2) {
      
      dmu <- dmu %>%
        summarize(mu=mean(logmfi),sd=sd(logmfi),ucut=mu+3*sd)
    
      # classify all observations using the derived cutoff
      di <- dmix %>%  
      filter(antigenf==ab) %>%
      mutate(posi=factor(ifelse(logmfi>dmu$ucut,1,0),levels=c(0,1)),
             posmix=factor(posmix,levels=c(0,1)))
    
      tabi <- as.vector(table(di$posmix,di$posi))
      names(tabi) <- c("mix0cuti0","mix1cuti0","mix0cuti1","mix1cuti1")
      kappai <- psych::cohen.kappa(table(di$posmix,di$posi))$kappa
      tabi <- data.frame(t(tabi))
      tabi <- tabi %>%
        mutate(Nobs=mix0cuti0+mix1cuti0+mix0cuti1+mix1cuti1,
             agreement=(mix0cuti0+mix1cuti1)/Nobs,
             kappa=kappai,
             antigenf=ab,
             abchange=icut,
             abcutoff=dmu$ucut) 
      
    } else{
      tabi <- data.frame(mix0cuti0=NA,mix1cuti0=NA,mix0cuti1=NA,mix1cuti1=NA,Nobs=NA,agreement=NA,kappa=NA,antigenf=ab,abchange=icut,abcutoff=NA)
      
      }

    }  

gridmix <- gridmix %>% mutate(antigenf=factor(antigenf,levels=levels(dmix$antigenf)))

# identify the optimal change based on % agreement, take minimum change
optchgmix <- gridmix %>%
  group_by(antigenf) %>%
  mutate(maxagr=max(agreement,na.rm=TRUE),
         maxchg=ifelse( abs(agreement-maxagr)<0.001,1,0)) %>%
  filter(maxchg==1) %>%
  # mutate(agr99=ifelse(agreement>0.99 & !is.na(agreement),1,0)) %>%
  # filter(agr99==1) %>%
  arrange(antigenf,abchange) %>%
  filter(row_number()==1) %>%
  select(antigenf,optchg=abchange,optagr=agreement,optkap=kappa,optcut=abcutoff)

gridmix <- left_join(gridmix,optchgmix,by="antigenf") %>%
  group_by(antigenf)
```

The bottom panel plots the estimated seropositivity cutoff as a function of change in MFI levels used to identify presumed seroconverters. The mean plus 3 SDs of the distribution of antibody levels before presumed seroconversion was used to estimate the cutoff. For a given cutoff, the middle and top panels summarize classification agreement with the mixture model-based seropositiity cutoff based on Cohen’s Kappa (middle) and proportion agreement (top).

Labeled dashed lines indicate the minimum magnitude of change in antibody levels that led to highest agreement, along with the corresponding seropositivity cutoff values, Cohen’s Kappa, and agreement.

```
plot_optchange(results=gridmix,classlab="Mixture model-based classification")
```

## Kenya

### Load the formatted data

```
#-----------------------------
# load the formatted data
# created with 
# asembo-enteric-ab-data-format.Rmd ->
# asembo-enteric-ab-distributions.Rmd
#-----------------------------
dl <- readRDS(here::here("data","asembo_analysis2.rds"))

# list the enteric antigens and make formatted labels for them
mbavars <- c("vsp3","vsp5","cp17","cp23","leca","salb","sald","etec","cholera","p18","p39")

mbalabs <- c("Giardia VSP-3","Giardia VSP-5","Cryptosporidium Cp17","Cryptosporidium Cp23","E. histolytica LecA","Salmonella LPS group B","Salmonella LPS group D","ETEC LT B subunit","Cholera toxin B subunit","Campylobacter p18","Campylobacter p39")

dl <- dl %>% mutate(antigenf=factor(antigenf,levels=mbalabs))
```

### Sensitivity analysis over fold-change values, ROC-based reference

*Giardia* and *Cryptosporidium* antigens have ROC-based cutoff values in this study. For these antigens, search over fold-change values to determine the cutoff with best agreement with ROC-based classification.

```
droc <- dl %>%
  filter(!is.na(roccut)) %>%
  mutate(antigenf=droplevels(antigenf))

gridroc <- foreach(ab=levels(droc$antigenf),.combine=rbind) %:%
  foreach(icut=seq(0.3,2.5,by=0.01),.combine=rbind) %dopar% {
    
    # downsample to first measurements who increased by the increment icut
    # these are "presumed unexposed" under this cutoff in change icut
    # estimate mean and SD among the presumed unexposed to use as a 
    # seropositivity cutoff
    dmu <- droc %>% 
      ungroup() %>%
      select(antigenf,time,logmfi,logmfidiff) %>%
      filter(antigenf==ab & time=="A" & logmfidiff>icut) %>%
      summarize(mu=mean(logmfi),sd=sd(logmfi),ucut=mu+3*sd)
    
    # classify all observations using the derived cutoff
    di <- droc %>%  
      filter(antigenf==ab) %>%
      mutate(posi=factor(ifelse(logmfi>dmu$ucut,1,0),levels=c(0,1)))
    
    # estimate agreement
    tabi <- as.vector(table(as.factor(di$posroc),as.factor(di$posi)))
    names(tabi) <- c("roc0cuti0","roc1cuti0","roc0cuti1","roc1cuti1")
    kappai <- psych::cohen.kappa(table(di$posroc,di$posi))$kappa
    tabi <- data.frame(t(tabi))
    tabi <- tabi %>%
      mutate(Nobs=roc0cuti0+roc1cuti0+roc0cuti1+roc1cuti1,
             agreement=(roc0cuti0+roc1cuti1)/Nobs,
             kappa=kappai,
             antigenf=ab,
             abchange=icut,
             abcutoff=dmu$ucut) 
    
  }

gridroc <- gridroc %>% mutate(antigenf=factor(antigenf,levels=levels(droc$antigenf)))

# identify the optimal change based on % agreement, take minimum change
optchgroc <- gridroc %>%
  group_by(antigenf) %>%
  mutate(maxagr=max(agreement,na.rm=TRUE),
         maxchg=ifelse( abs(agreement-maxagr)<0.001,1,0)) %>%
  filter(maxchg==1) %>%
  arrange(antigenf,abchange) %>%
  filter(row_number()==1) %>%
  select(antigenf,optchg=abchange,optagr=agreement,optkap=kappa,optcut=abcutoff)

# merge in the optimal change values
gridroc <- left_join(gridroc,optchgroc,by="antigenf")  %>%
  group_by(antigenf)
```

#### Figure to summarize results

The bottom panel plots the estimated seropositivity cutoff as a function of change in MFI levels used to identify presumed seroconverters. The mean plus 3 SDs of the distribution of antibody levels before presumed seroconversion was used to estimate the cutoff. For a given cutoff, the middle and top panels summarize classification agreement with the ROC-based seropositiity cutoff based on Cohen’s Kappa (middle) and proportion agreement (top).

Labeled dashed lines indicate the minimum magnitude of change in antibody levels that led to highest agreement, along with the corresponding seropositivity cutoff values, Cohen’s Kappa, and agreement.

```
plot_optchange(results=gridroc,classlab="ROC-based classification")
```

### Sensitivity analysis over fold-change values, mixture model-based reference

Search over fold-change values to determine the cutoff with best agreement with mixture model-based classification

```
dmix <- dl %>%
  filter(!is.na(mixcut)) %>%
  mutate(antigenf=droplevels(antigenf))

gridmix <- foreach(ab=levels(dmix$antigenf),.combine=rbind) %:%
  foreach(icut=seq(0.3,2.5,by=0.01),.combine=rbind) %dopar% {

    # downsample to first measurements who increased by the increment icut
    # these are "presumed unexposed" under this cutoff in change icut
    # estimate mean and SD among the presumed unexposed to use as a 
    # seropositivity cutoff
    dmu <- dmix %>% 
      ungroup() %>%
      select(antigenf,time,logmfi,logmfidiff) %>%
      filter(antigenf==ab & time=="A" & logmfidiff>icut)
    
    # if there are <2 obs, cannot estimate mean + SD, so skip
    if(nrow(dmu)>=2) {
      
      dmu <- dmu %>%
        summarize(mu=mean(logmfi),sd=sd(logmfi),ucut=mu+3*sd)
    
      # classify all observations using the derived cutoff
      di <- dmix %>%  
      filter(antigenf==ab) %>%
      mutate(posi=factor(ifelse(logmfi>dmu$ucut,1,0),levels=c(0,1)),
             posmix=factor(posmix,levels=c(0,1)))
    
      tabi <- as.vector(table(di$posmix,di$posi))
      names(tabi) <- c("mix0cuti0","mix1cuti0","mix0cuti1","mix1cuti1")
      kappai <- psych::cohen.kappa(table(di$posmix,di$posi))$kappa
      tabi <- data.frame(t(tabi))
      tabi <- tabi %>%
        mutate(Nobs=mix0cuti0+mix1cuti0+mix0cuti1+mix1cuti1,
             agreement=(mix0cuti0+mix1cuti1)/Nobs,
             kappa=kappai,
             antigenf=ab,
             abchange=icut,
             abcutoff=dmu$ucut) 
      
    } else{
      tabi <- data.frame(mix0cuti0=NA,mix1cuti0=NA,mix0cuti1=NA,mix1cuti1=NA,Nobs=NA,agreement=NA,kappa=NA,antigenf=ab,abchange=icut,abcutoff=NA)
      
      }

    }  

gridmix <- gridmix %>% mutate(antigenf=factor(antigenf,levels=levels(dmix$antigenf)))

# identify the optimal change based on % agreement, take minimum change
optchgmix <- gridmix %>%
  group_by(antigenf) %>%
  mutate(maxagr=max(agreement,na.rm=TRUE),
         maxchg=ifelse( abs(agreement-maxagr)<0.001,1,0)) %>%
  filter(maxchg==1) %>%
  # mutate(agr99=ifelse(agreement>0.99 & !is.na(agreement),1,0)) %>%
  # filter(agr99==1) %>%
  arrange(antigenf,abchange) %>%
  filter(row_number()==1) %>%
  select(antigenf,optchg=abchange,optagr=agreement,optkap=kappa,optcut=abcutoff)

gridmix <- left_join(gridmix,optchgmix,by="antigenf") %>%
  group_by(antigenf)
```

The bottom panel plots the estimated seropositivity cutoff as a function of change in MFI levels used to identify presumed seroconverters. The mean plus 3 SDs of the distribution of antibody levels before presumed seroconversion was used to estimate the cutoff. For a given cutoff, the middle and top panels summarize classification agreement with the mixture model-based seropositiity cutoff based on Cohen’s Kappa (middle) and proportion agreement (top).

Labeled dashed lines indicate the minimum magnitude of change in antibody levels that led to highest agreement, along with the corresponding seropositivity cutoff values, Cohen’s Kappa, and agreement.

```
plot_optchange(results=gridmix,classlab="Mixture model-based classification")
```

# Session Info

```
sessionInfo()
```

```
## R version 3.5.3 (2019-03-11)
## Platform: x86_64-apple-darwin15.6.0 (64-bit)
## Running under: macOS High Sierra 10.13.6
## 
## Matrix products: default
## BLAS: /System/Library/Frameworks/Accelerate.framework/Versions/A/Frameworks/vecLib.framework/Versions/A/libBLAS.dylib
## LAPACK: /Library/Frameworks/R.framework/Versions/3.5/Resources/lib/libRlapack.dylib
## 
## locale:
## [1] en_US.UTF-8/en_US.UTF-8/en_US.UTF-8/C/en_US.UTF-8/en_US.UTF-8
## 
## attached base packages:
## [1] grid      parallel  stats     graphics  grDevices utils     datasets 
## [8] methods   base     
## 
## other attached packages:
##  [1] psych_1.8.4              tmle_1.3.0-1            
##  [3] SuperLearner_2.0-25-9000 nnls_1.4                
##  [5] mgcv_1.8-27              ROCR_1.0-7              
##  [7] gplots_3.0.1             tmleAb_0.3.4            
##  [9] nlme_3.1-137             gridExtra_2.3           
## [11] RColorBrewer_1.1-2       ellipse_0.4.1           
## [13] scales_1.0.0             knitr_1.22              
## [15] mixtools_1.1.0           viridis_0.5.1           
## [17] viridisLite_0.3.0        foreign_0.8-71          
## [19] lubridate_1.7.4          doParallel_1.0.11       
## [21] iterators_1.0.9          foreach_1.4.4           
## [23] kableExtra_0.8.0.0001    forcats_0.3.0           
## [25] stringr_1.4.0            dplyr_0.8.0.1           
## [27] purrr_0.3.2              readr_1.1.1             
## [29] tidyr_0.8.3              tibble_2.1.1            
## [31] ggplot2_3.1.1            tidyverse_1.2.1         
## [33] here_0.1                
## 
## loaded via a namespace (and not attached):
##  [1] httr_1.4.0         jsonlite_1.6       splines_3.5.3     
##  [4] modelr_0.1.2       gtools_3.5.0       assertthat_0.2.1  
##  [7] highr_0.8          cellranger_1.1.0   yaml_2.2.0        
## [10] pillar_1.4.0       backports_1.1.4    lattice_0.20-38   
## [13] glue_1.3.1         digest_0.6.18      rvest_0.3.2       
## [16] colorspace_1.3-2   htmltools_0.3.6    Matrix_1.2-15     
## [19] plyr_1.8.4         pkgconfig_2.0.2    broom_0.4.4       
## [22] haven_2.1.0        gdata_2.18.0       withr_2.1.2       
## [25] lazyeval_0.2.2     cli_1.1.0          mnormt_1.5-5      
## [28] survival_2.43-3    magrittr_1.5       crayon_1.3.4      
## [31] readxl_1.1.0       evaluate_0.13      MASS_7.3-51.1     
## [34] segmented_0.5-3.0  xml2_1.2.0         tools_3.5.3       
## [37] hms_0.4.2          munsell_0.5.0      packrat_0.4.9-3   
## [40] compiler_3.5.3     caTools_1.17.1     rlang_0.3.4       
## [43] rstudioapi_0.9.0   bitops_1.0-6       labeling_0.3      
## [46] rmarkdown_1.12     gtable_0.3.0       codetools_0.2-16  
## [49] reshape2_1.4.3     R6_2.4.0           rprojroot_1.3-2   
## [52] KernSmooth_2.23-15 stringi_1.4.3      Rcpp_1.0.1        
## [55] tidyselect_0.2.5   xfun_0.6
```
