# Supplementary material for: Enteropathogen antibody dynamics and force of infection among children in low-resource settings
Source: eLife. 2019 Aug 19;8:e45594. doi: 10.7554/eLife.45594 (PMC6746552; doi:10.7554/eLife.45594)
Supplement: Supplementary file 6. [file elife-45594-supp6.zip › SIFile6.html]

Enteropathogen antibody dynamics and force of infection among children in low-resource settings


Code 

- Show All Code
- Hide All Code

# Enteropathogen antibody dynamics and force of infection among children in low-resource settings

### Supplementary Information File 6. Estimation of age-dependent means and seroprevalence using multiple approaches, Figure 3 including supplements 1, 2

# Notebook Summary

This notebook estimates age-dependent mean antibody and seroprevalence curves among children from Haiti, Kenya, and Tanzania. Estimators include cubic splines (in a GAM model from the `mgcv` package) and ensemble machine learning (using stacked super learning from the `tmleAb` package). Spline-based estimates are reported in the main text in Figure 3.

The notebook is fairly repetative by country.

# Script preamble

```
#-----------------------------
# preamble
#-----------------------------
library(here)
```

```
## here() starts at /Users/benarnold/enterics-seroepi
```

```
here::here()
```

```
## [1] "/Users/benarnold/enterics-seroepi"
```

```
library(tidyverse)
```

```
## ── Attaching packages ────────────────────────────── tidyverse 1.2.1 ──
```

```
## ✔ ggplot2 3.1.1       ✔ purrr   0.3.2  
## ✔ tibble  2.1.1       ✔ dplyr   0.8.0.1
## ✔ tidyr   0.8.3       ✔ stringr 1.4.0  
## ✔ readr   1.1.1       ✔ forcats 0.3.0
```

```
## ── Conflicts ───────────────────────────────── tidyverse_conflicts() ──
## ✖ dplyr::filter() masks stats::filter()
## ✖ dplyr::lag()    masks stats::lag()
```

```
library(viridis)
```

```
## Loading required package: viridisLite
```

```
library(gridExtra)
```

```
## 
## Attaching package: 'gridExtra'
```

```
## The following object is masked from 'package:dplyr':
## 
##     combine
```

```
library(kableExtra) # devtools::install_github("haozhu233/kableExtra")
```

```
## 
## Attaching package: 'kableExtra'
```

```
## The following object is masked from 'package:dplyr':
## 
##     group_rows
```

```
options(knitr.table.format = "html") 

# set up for parallel computing
# configure for a laptop (use only 3 cores)
library(foreach)
```

```
## 
## Attaching package: 'foreach'
```

```
## The following objects are masked from 'package:purrr':
## 
##     accumulate, when
```

```
library(doParallel)
```

```
## Loading required package: iterators
```

```
## Loading required package: parallel
```

```
registerDoParallel(cores=3)

# bright color blind palette:  https://personal.sron.nl/~pault/ 
cblack <- "#000004FF"
cblue <- "#3366AA"
cteal <- "#11AA99"
cgreen <- "#66AA55"
cchartr <- "#CCCC55"
cmagent <- "#992288"
cred <- "#EE3333"
corange <- "#EEA722"
cyellow <- "#FFEE33"
cgrey <- "#777777"

# viridis color palette
vircols <- viridis(n=7,alpha=1,begin=0.2,end=0.97)

# safe color blind palette
# http://jfly.iam.u-tokyo.ac.jp/color/
# http://www.cookbook-r.com/Graphs/Colors_(ggplot2)/
cbPalette <- c("#999999", "#E69F00", "#56B4E9", "#009E73", "#F0E442", "#0072B2", "#D55E00", "#CC79A7")
```

## Reused Functions

```
#----------------------------------
# simulataneous CIs for GAMs
# estimated by resampling the 
# Baysian posterior estimates of
# the variance-covariance matrix
# assuming that it is multivariate normal
# the function below also estimates 
# the unconditional variance-covariance
# matrix, Vb=vcov(x,unconditional=TRUE), 
# which allows for undertainty in the actual
# estimated mean as well 
# (Marra & Wood 2012 Scandinavian Journal of Statistics, 
#  Vol. 39: 53–74, 2012, doi: 10.1111/j.1467-9469.2011.00760.x )
# simultaneous CIs provide much better coverage than pointwise CIs
# see: http://www.fromthebottomoftheheap.net/2016/12/15/simultaneous-interval-revisited/
#----------------------------------

gamCI <- function(m,newdata,nreps=10000) {
  require(mgcv)
  require(dplyr)
  Vb <- vcov(m,unconditional = TRUE)
  pred <- predict(m, newdata, se.fit = TRUE)
  fit <- pred$fit
  se.fit <- pred$se.fit
  BUdiff <- MASS::mvrnorm(n=nreps, mu = rep(0, nrow(Vb)), Sigma = Vb)
  Cg <- predict(m, newdata, type = "lpmatrix")
  simDev <- Cg %*% t(BUdiff)
  absDev <- abs(sweep(simDev, 1, se.fit, FUN = "/"))
  masd <- apply(absDev, 2L, max)
  crit <- quantile(masd, prob = 0.95, type = 8)
  pred <- data.frame(newdata,fit=pred$fit,se.fit=pred$se.fit)
  pred <- mutate(pred,
                 uprP = fit + (2 * se.fit),
                 lwrP = fit - (2 * se.fit),
                 uprS = fit + (crit * se.fit),
                 lwrS = fit - (crit * se.fit)
  )
  return(pred)
}


#-----------------------------
# Function to estimate exact
# binomial confidence intervals
# for prevalence estimates
#-----------------------------
exactprev <- function(x) {
  # x : a binary indicator of the outcome (1/0)
  tabx <- table(x)
  if(length(tabx)<2) {
    if(names(tabx)=="1") {
      tabx <- c(0,tabx)
    } else{
      tabx <- c(tabx,0)
    }
  } 
  estx <- binom.test(x=tabx[2],n=sum(tabx))
  res <- c(estx$parameter,estx$statistic,estx$estimate,estx$conf.int)
  names(res) <- c("N","n","mean","min95","max95")
  return(res)
}
```

# Haiti

##Load the formatted data

```
#-----------------------------
# load the formatted data
# with seropositivity indicators
# created with:
# haiti-enteric-ab-data-format.Rmd -->
# Fig1-haiti-ab-distributions.Rmd
#-----------------------------
dl <- readRDS(here::here("data","haiti_analysis2.rds"))
```

## Estimate age-dependent mean antibody levels

### Estimate curves with splines in a generalized additive model (GAM)

```
# fit cubic spline over age, separately for each antigen
# estimate approximate simultaneous confidence intervals
# for the fits
library(mgcv)
```

```
## Loading required package: nlme
```

```
## 
## Attaching package: 'nlme'
```

```
## The following object is masked from 'package:dplyr':
## 
##     collapse
```

```
## This is mgcv 1.8-27. For overview type 'help("mgcv-package")'.
```

```
gamfits <- foreach(ab=levels(dl$antigenf),.combine=rbind) %dopar% {
    pd <- filter(dl, antigenf==ab)
    gfit <- gam(logmfi~s(age, bs="cr"),data=pd)
    gsci <- gamCI(m=gfit,newdata=pd,nreps=1000)
    gsci$antigen <- ab
    return(gsci)
  }
```

## Estimate curves with ensemble machine learning

```
#-----------------------------
# estimate curves with 
# ensemble machine learning
# including a library with
# GLM, GAM, Loess, and 
# extreme gradient boosting
#-----------------------------

# detach the mgcv package because
# the ensemble algorithm uses gam()
# from the gam package and there are
# conflicts between them
detach("package:mgcv", unload=TRUE)

# load the tmleAb package, at this time
# only available through github: 
# devtools::install_github("ben-arnold/tmleAb")
library(tmleAb)
set.seed(123)
slfits <-  foreach(ab=levels(dl$antigenf),.combine=rbind) %dopar% {
  pd <- filter(dl, antigenf==ab)
  slfit <- agecurveAb(Y=pd$logmfi,
                      Age=pd$age,
                      id=pd$id,
                      SL.library = c("SL.glm","SL.gam","SL.loess","SL.xgboost"))
  sld <- data.frame(age=slfit$Age,fit=slfit$pY)
  sld <- sld %>% group_by(age) %>% filter(row_number()==1) %>% ungroup()
  slres <- left_join(pd,sld,by=c("age"))
  return(slres)
}

#-----------------------------
# estimate means by age in 
# years (0-9)
# to get 95% CIs through the
# influence curve
# there are 19 measurements
# among children 10-12 that
# are excluded for these estimates
# due to small sample sizes
#-----------------------------

tmlemeans <- foreach(ab=levels(dl$antigenf),.combine=rbind) %:%
  foreach(agei=0:9,.combine=rbind) %dopar% {
    pd <- filter(dl,antigenf==ab & floor(age)==agei)
    mu <- tmleAb(Y=pd$logmfi,id=pd$id,SL.library="SL.mean")
    di <- data.frame(pathogen=pd$pathogen[1],antigenf=ab,age=agei,mean=mu$psi,se=mu$se,lb=mu$lb,ub=mu$ub)
    di
  }
```

### Age-dependent mean antibody curves, all estimates

Plot all of the estimates together. The spline fits are colored and the highly adaptive ensemble fits are grey. Empirical means with 95% CIs (averaged by year) are black. The approximate simultaneous confidence bands for the spline fit are shaded. The plots are trimmed at age 10 years to reduce edge effects because there are only 19 measurements older than 10 years.

```
log10labs <- c( 
  expression(10^0),
  expression(10^1),
  expression(10^2),
  expression(10^3),
  expression(10^4)
)

# custom color blind color palette is in the preamble chunck
# pcols <- c(cred,corange,cgreen,cteal,cblue,cmagent)
pcols <- cbPalette[c(2:4,6,7,8)]

pcurveall <- ggplot(data=gamfits,aes(x=age,group=pathogen,color=pathogen,fill=pathogen)) +
  # approximate simultaneous CI from spline fit
  geom_ribbon(aes(ymin=lwrS,ymax=uprS),alpha=0.2,color=NA,fill=cgrey) +
  # actual measurements
  # geom_jitter(aes(y=logmfi),width=0.2,height=0,color="black",alpha=0.4,size=0.3) +
  # ensemble fit
  geom_line(data=slfits,aes(x=age,y=fit),color=cgrey) +
  # spline fit
  geom_line(aes(y=fit)) +
  # empirical means 95% CI from the influence curve (averaged for each age)
  geom_pointrange(data=tmlemeans,aes(x=age+0.5,y=mean,ymin=lb,ymax=ub),size=0.2,color=cblack)+
  # plot aesthetics
  facet_wrap(~antigenf,nrow=6,ncol=2) +
  scale_x_continuous(limits=c(0,10),breaks=seq(0,10,by=2))+
  scale_y_continuous(breaks=0:4,labels=log10labs)+
  coord_cartesian(ylim=c(0,4.5))+
  labs(x="Age in years",y="Luminex Response (MFI-bg)") +
  scale_fill_manual(values=pcols) +
  scale_color_manual(values=pcols) +
  theme_minimal() +
  theme(legend.position = "none")

pcurveall
```

```
## Warning: Removed 114 rows containing missing values (geom_path).

## Warning: Removed 114 rows containing missing values (geom_path).
```

## Age-dependent seroprevalence

### Estimate seroprevalence curves with splines in a generalized additive model (GAM)

```
#-----------------------------
# subset the data to antigens
# with seropositivity estimates
# hierarchy of information for 
# cutoffs:
# 1. ROC
# 2. mixture model based
# 3. estimated among presumed unexposed
#
# store the cutoff value used
# for figures
#-----------------------------
dlp <- dl %>%
  mutate(seropos=ifelse(!is.na(posroc),posroc,posmix),
         serocut=ifelse(!is.na(posroc),log10(roccut),mixcut),
         serocut_desc=ifelse(!is.na(posroc),"ROC","Mixture Model")) %>%
  mutate(seropos=ifelse(!is.na(seropos),seropos,posunex),
         serocut_desc=ifelse(!is.na(serocut),serocut_desc,"Unexp dist"),
         serocut=ifelse(!is.na(serocut),serocut,unexpcut)) %>%
  filter(!is.na(seropos))

#-----------------------------
# create composite 
# seropositivity indicators
# that use information from
# multiple antigens
#-----------------------------

dgi <- dlp %>% 
  filter(antigen %in% c("vsp3","vsp5")) %>%
  select(id,sampleid,age,pathogen,antigen,seropos) %>%
  spread(antigen,seropos) %>%
  mutate(seropos=ifelse(vsp3==1|vsp5==1,1,0),
         antigen="vsp3vsp5",
         antigenf="Giardia VSP-3 or VSP-5") %>%
  select(-vsp3,-vsp5)

dcr <- dlp %>% 
  filter(antigen %in% c("cp17","cp23")) %>%
  select(id,sampleid,age,pathogen,antigen,seropos) %>%
  spread(antigen,seropos) %>%
  mutate(seropos=ifelse(cp17==1|cp23==1,1,0),
         antigen="cp17cp23",
         antigenf="Cryptosporidium Cp17 or Cp23") %>%
  select(-cp17,-cp23)

ds <- dlp %>% 
  filter(antigen %in% c("salb","sald")) %>%
  select(id,sampleid,age,pathogen,antigen,seropos) %>%
  spread(antigen,seropos) %>%
  mutate(seropos=ifelse(salb==1|sald==1,1,0),
         antigen="salbsald",
         antigenf="Salmonella LPS groups B or D") %>%
  select(-salb,-sald)


dlp <- bind_rows(dlp,dgi,dcr,ds) %>%
  mutate(antigenf=factor(antigenf,
                         levels=c("Giardia VSP-3 or VSP-5",
                                  "Cryptosporidium Cp17 or Cp23",
                                  "E. histolytica LecA",
                                  "Salmonella LPS groups B or D",
                                  "ETEC LT B subunit",
                                  "Norovirus GI.4", 
                                  "Norovirus GII.4.NO")) ) %>%
  filter(!is.na(antigenf)) %>%
  select(pathogen,antigen,antigenf,
         id,sampleid,age,
         seropos) %>%
  arrange(pathogen,antigenf,id,sampleid)
```

```
#-----------------------------
# estimate seroprevalence curves with 
# splines
#-----------------------------

# fit cubic spline over age, separately for each antigen
# estimate approximate simultaneous confidence intervals
# for the fits
library(mgcv)
```

```
## This is mgcv 1.8-27. For overview type 'help("mgcv-package")'.
```

```
pgamfits <- foreach(ab=levels(dlp$antigenf),.combine=rbind) %dopar% {
    pd <- filter(dlp, antigenf==ab)
    gfit <- mgcv::gam(seropos~s(age, bs="cr"),data=pd,family="binomial")
    gsci <- gamCI(m=gfit,newdata=pd,nreps=1000)
    gsci$antigenf <- ab
    return(gsci)
}

# use antilogit to get back to prevalence from the logit link
pgamfits <- pgamfits %>%
  mutate(fit  = 1/(1+exp(-fit)),
         uprP = 1/(1+exp(-uprP)),
         lwrP = 1/(1+exp(-lwrP)),
         uprS = 1/(1+exp(-uprS)),
         lwrS = 1/(1+exp(-lwrS))) %>%
  mutate(antigenf=factor(antigenf,levels=levels(dlp$antigenf)))
```

### Estimate seroprevalence curves with ensemble machine learning

```
#-----------------------------
# estimate curves with 
# ensemble machine learning
# including a library with
# GLM, GAM, Loess, and 
# extreme gradient boosting
#-----------------------------

# detach the mgcv package because
# the ensemble algorithm uses gam()
# from the gam package and there are
# conflicts between them
detach("package:mgcv", unload=TRUE)

set.seed(12345)
pslfits <-  foreach(ab=levels(dlp$antigenf),.combine=rbind) %dopar% {
  pd <- filter(dlp, antigenf==ab)
  slfit <- agecurveAb(Y=pd$seropos,
                      Age=pd$age,
                      id=pd$id,
                      family="binomial",
                      SL.library = c("SL.glm","SL.gam","SL.xgboost"))
  sld <- data.frame(age=slfit$Age,fit=slfit$pY)
  sld <- sld %>% group_by(age) %>% filter(row_number()==1) %>% ungroup()
  slres <- left_join(pd,sld,by=c("age"))
  return(slres)
}

#-----------------------------
# estimate prevalence by age in 
# years (0-9)
# since prevalence is very sparse
# in some strata, cannot use the 
# tmleAb() function above for
# influence-curve based SEs
# because it uses cross-validation
# and sometimes there are folds
# with no events. So, instead
# use an exact binomial approach
#-----------------------------
prevests <- foreach(ab=levels(dlp$antigenf),.combine=rbind) %:%
  foreach(agei=0:9,.combine=rbind) %dopar% {
      pd <- filter(dlp,antigenf==ab & floor(age)==agei)
      mus <- exactprev(pd$seropos)
      res <- data.frame(pathogen=pd$pathogen[1],antigenf=ab,age=agei,N=mus[1],n=mus[2],mean=mus[3],lb=mus[4],ub=mus[5])
      res
    }
```

### Age-dependent seroprevalence curves, all estimates

Plot all of the estimates together. The spline fits are colored and the highly adaptive ensemble fits are grey. Empirical means with 95% CIs (averaged by year) are black. The approximate simultaneous confidence bands for the spline fit are shaded. The plots are trimmed at age 10 years to reduce edge effects because there are only 19 measurements older than 10 years.

```
# set ETEC confidence band to NA after it flatlines at 100%
dplot <- pgamfits %>%
  mutate(lwrS=ifelse(antigen=="etec" & fit==1,NA,lwrS))


# bright color-blind palette defined in an earlier code chunk
# pcols <- c(cred,corange,cgreen,cteal,cblue,cmagent,cmagent)
# pcols <- vircols[c(1:5,7,7)]
pcols <- cbPalette[c(2:4,6,7,8,8)]


ppcurve <- ggplot(data=dplot,aes(x=age,color=antigenf,fill=antigenf)) +
  # approximate simultaneous CI from spline fit
  geom_ribbon(aes(ymin=lwrS,ymax=uprS),alpha=0.2,color=NA,fill=cgrey) +
  # actual measurements
  # geom_jitter(aes(y=posmix),width=0.2,height=0,color="black",alpha=0.4,size=0.3) +
   # ensemble fit
  geom_line(data=pslfits,aes(x=age,y=fit),color=cgrey) +
  # spline fit
  geom_line(aes(y=fit)) +
  # empirical means 95% CI from the influence curve (averaged for each age)
  geom_pointrange(data=prevests,aes(x=age+0.5,y=mean,ymin=lb,ymax=ub),size=0.2,color=cblack)+
  # plot aesthetics
  facet_wrap(~antigenf,nrow=6,ncol=2) +
  scale_x_continuous(limits=c(0,10),breaks=seq(0,10,by=2))+
  scale_y_continuous(breaks=seq(0,1,by=0.2),labels=sprintf("%1.0f",seq(0,1,by=0.2)*100))+
  coord_cartesian(ylim=c(0,1))+
  labs(x="Age in years",y="Seroprevalence (%)") +
  scale_fill_manual(values=pcols) +
  scale_color_manual(values=pcols) +
  theme_minimal() +
  theme(legend.position = "none")

ppcurve
```

```
## Warning: Removed 133 rows containing missing values (geom_path).

## Warning: Removed 133 rows containing missing values (geom_path).
```

## Figure 3, age dependent means

Create a more parsimonius plot, that only includes the spline fits but also includes the actual points.

Age-dependent mean MFI curves

```
log10labs <- c( 
  expression(10^0),
  expression(10^1),
  expression(10^2),
  expression(10^3),
  expression(10^4)
)

# create a variable for faceting panels by pathogen
# keep norovirus GI and GII split for consistency with other figures and
# because they exhibit distinct patterns across all analyses. 
gamfits <- gamfits %>%
  mutate(antigenn=ifelse(antigenf %in% c("Giardia VSP-5","Cryptosporidium Cp23","Salmonella LPS group D"),2,1),
         pathogeng=factor(pathogen,levels=c(levels(pathogen),"Norovirus GI","Norovirus GII"))
  )
gamfits$pathogeng[gamfits$antigenf=="Norovirus GI.4"] <- "Norovirus GI"
gamfits$pathogeng[gamfits$antigenf=="Norovirus GII.4.NO"] <- "Norovirus GII"

# labels for the panels in the plot 
plabs <- gamfits %>%
  group_by(pathogeng,antigenn) %>%
  select(pathogen,pathogeng,antigenn,antigenf) %>%
  slice(1)


# custom color blind color palette is in the preamble chunck
# pcols <- c(cred,corange,cgreen,cteal,cblue,cmagent,cmagent)
# pcols <- vircols[c(1:5,6,6)]
pcols <- cbPalette[c(2:4,6,7,8,8)]


pcurve <- ggplot(data=gamfits,aes(x=age,color=pathogeng,fill=pathogeng)) +
  # approximate simultaneous CI from spline fit
  geom_ribbon(aes(ymin=lwrS,ymax=uprS),alpha=0.3,color=NA,fill=cgrey) +
  # actual measurements
  geom_jitter(aes(y=logmfi),width=0.2,height=0,alpha=0.4,size=0.3) +
  # spline fit
  geom_line(aes(y=fit),color="black") +
  # labels
  geom_text(data=plabs,aes(x=0,y=4.55,label=antigenf,hjust=0,vjust=0),color="gray20",size=3.4)+
  # plot aesthetics
  facet_grid(pathogeng~antigenn) +
  scale_x_continuous(limits=c(0,10),breaks=seq(0,10,by=2))+
  scale_y_continuous(breaks=0:4,labels=log10labs)+
  coord_cartesian(ylim=c(0,4.65))+
  labs(x="Age in years",y="Luminex Response (MFI-bg)",tag="A") +
  scale_fill_manual(values=pcols) +
  scale_color_manual(values=pcols) +
  theme_minimal(base_size=16) +
  theme(legend.position = "none",
        strip.text=element_blank())
```

Seroprevalence curves

```
# labels for the panels in the plot 
pplabs <- pgamfits %>%
  group_by(antigenf) %>%
  select(pathogen,antigenf) %>%
  slice(1)

# bright color-blind palette defined in an earlier code chunk
# pcols <- c(cred,corange,cgreen,cteal,cblue,cmagent,cmagent)
# pcols <- vircols[c(1:5,6,6)]
pcols <- cbPalette[c(2:4,6,7,8,8)]


ppcurve2 <- ggplot(data=dplot,aes(x=age,color=antigenf,fill=antigenf)) +
  # approximate simultaneous CI from spline fit
  geom_ribbon(aes(ymin=lwrS,ymax=uprS),alpha=0.3,color=NA,fill=cgrey) +
  # actual measurements
  geom_point(aes(y=seropos),alpha=0.5,size=1.5,pch="|") +
  # spline fit
  geom_line(aes(y=fit),color="black") +
  # labels
  geom_text(data=pplabs,aes(x=0,y=1.03,label=antigenf,hjust=0,vjust=0),color="gray20",size=3.4)+
  # plot aesthetics
  facet_wrap(~antigenf,nrow=7,ncol=1) +
  scale_x_continuous(limits=c(0,10),breaks=seq(0,10,by=2))+
  scale_y_continuous(breaks=seq(0,1,by=0.2),labels=sprintf("%1.0f",seq(0,1,by=0.2)*100))+
  coord_cartesian(ylim=c(0,1.04))+
  labs(x="Age in years",y="Seroprevalence (%)",tag="B") +
  scale_fill_manual(values=pcols) +
  scale_color_manual(values=pcols) +
  theme_minimal(base_size=16) +
  theme(legend.position = "none",
        strip.text=element_blank())
```

Composite figure

\*\* This is Figure 3 in the manuscript \*\*

```
# labels for the panels in the plot 
pcurve_composite_haiti <- grid.arrange(pcurve,ppcurve2,ncol=2,nrow=1,widths=c(2,1.1))
```

```
## Warning: Removed 222 rows containing missing values (geom_point).
```

```
## Warning: Removed 133 rows containing missing values (geom_path).
```

```
## Warning: Removed 133 rows containing missing values (geom_point).
```

```
## Warning: Removed 133 rows containing missing values (geom_path).
```

```
# save PDF and TIFF versions
ggsave(here::here("figs","Fig3-haiti-ab-agecurves.pdf"),plot=pcurve_composite_haiti,device=cairo_pdf,width=9,height=14)
ggsave(here::here("figs","Fig3-haiti-ab-agecurves.TIFF"),plot=pcurve_composite_haiti,device="tiff",width=9,height=14)
```

### Numerical seroprevalence estimates to report in the manuscript.

```
# grab predicted means from cubic splines very close to each year of age
pests <- pgamfits %>%
  mutate(agey = floor(age)) %>%
  filter(age+0.02 > agey & age-0.02 < agey) %>%
  group_by(antigenf) %>%
  filter(!duplicated(agey)) %>%
  arrange(antigenf,agey) %>%
  select(antigenf,agey,prev=fit,prev_min95=lwrS,prev_max95=uprS)

pests
```

## Save fitted estimates for later use, Haiti

```
# spline fits of geometric means
saveRDS(gamfits,file=here::here("output","haiti-enteric-ab-gamfits-means.rds"))
# ensemble fits of geometric means
saveRDS(slfits,file=here::here("output","haiti-enteric-ab-slfits-means.rds"))
# spline fits of age-dependent seroprevalence
saveRDS(pgamfits,file=here::here("output","haiti-enteric-ab-gamfits-seroprev.rds"))
# spline fits of age-dependent seroprevalence
saveRDS(pslfits,file=here::here("output","haiti-enteric-ab-slfits-seroprev.rds"))
```

# Kenya

## Load the formatted Kenya data

```
#-----------------------------
# load the formatted data
# with seropositivity indicators
# created with:
# asembo-enteric-ab-data-format.Rmd -->
# Fig1sup3-asembo-enteric-ab-distributions.Rmd
#-----------------------------
dl <- readRDS(here::here("data","asembo_analysis2.rds"))
```

## Estimate age-dependent mean antibody levels

### Estimate curves with splines in a generalized additive model (GAM)

```
# fit cubic spline over age, separately for each antigen
# estimate approximate simultaneous confidence intervals
# for the fits
library(mgcv)
```

```
## This is mgcv 1.8-27. For overview type 'help("mgcv-package")'.
```

```
gamfits <- foreach(ab=levels(dl$antigenf),.combine=rbind) %dopar% {
    pd <- filter(dl, antigenf==ab)
    gfit <- gam(logmfi~s(age, bs="cr"),data=pd)
    gsci <- gamCI(m=gfit,newdata=pd,nreps=1000)
    gsci$antigen <- ab
    return(gsci)
  }
```

### Estimate curves with ensemble machine learning

```
#-----------------------------
# estimate curves with 
# ensemble machine learning
# including a library with
# GLM, GAM, Loess, and 
# extreme gradient boosting
#-----------------------------

# detach the mgcv package because
# the ensemble algorithm uses gam()
# from the gam package and there are
# conflicts between them
detach("package:mgcv", unload=TRUE)

# load the tmleAb package, at this time
# only available through github: 
# devtools::install_github("ben-arnold/tmleAb")
library(tmleAb)
set.seed(12345)
slfits <-  foreach(ab=levels(dl$antigenf),.combine=rbind) %dopar% {
  pd <- filter(dl, antigenf==ab)
  slfit <- agecurveAb(Y=pd$logmfi,
                      Age=pd$age,
                      id=pd$childid,
                      SL.library = c("SL.glm","SL.gam","SL.loess","SL.xgboost"))
  sld <- data.frame(age=slfit$Age,fit=slfit$pY)
  sld <- sld %>% group_by(age) %>% filter(row_number()==1) %>% ungroup()
  slres <- left_join(pd,sld,by=c("age"))
  return(slres)
}


#-----------------------------
# estimate means by age in 
# months (4-17)
# to get 95% CIs through the
# influence curve
# there is 1 measurement
# at age 18 months that is
# excluded from this calculation
#-----------------------------

tmlemeans <- foreach(ab=levels(dl$antigenf),.combine=rbind) %:%
  foreach(agei=4:17,.combine=rbind) %dopar% {
    pd <- filter(dl,antigenf==ab & floor(age)==agei)
    mu <- tmleAb(Y=pd$logmfi,id=pd$id,SL.library="SL.mean")
    di <- data.frame(pathogen=pd$pathogen[1],antigenf=ab,age=agei,mean=mu$psi,se=mu$se,lb=mu$lb,ub=mu$ub)
    di
  }
```

### Age-dependent mean antibody curves, all estimates

The spline fits are colored and the highly adaptive ensemble fits are grey. The approximate simultaneous confidence bands for the spline fit are shaded.

```
log10labs <- c( 
  expression(10^0),
  expression(10^1),
  expression(10^2),
  expression(10^3),
  expression(10^4)
)

# bright color-blind palette defined in an earlier code chunk
# pcols <- c(cred,corange,cgreen,cteal,cblue,cgrey,cmagent)
# pcols <- c(corange,cred,cmagent,cchartr,cblue,cteal,cgreen,cgrey)
# pcols <- vircols[c(1:7)]
pcols <- cbPalette[c(2:4,6,7,5,8,1)]


pcurve <- ggplot(data=gamfits,aes(x=age,group=pathogen,color=pathogen,fill=pathogen)) +
  # approximate simultaneous CI from spline fit
  geom_ribbon(aes(ymin=lwrS,ymax=uprS),alpha=0.2,color=NA,fill=cgrey) +
  # ensemble fit
  geom_line(data=slfits,aes(x=age,y=fit),color=cgrey) +
  # spline fit
  geom_line(aes(y=fit)) +
  # empirical means 95% CI from the influence curve (averaged for each age)
  geom_pointrange(data=tmlemeans,aes(x=age,y=mean,ymin=lb,ymax=ub),size=0.2,color=cblack)+
  # plot aesthetics
  facet_wrap(~antigenf,nrow=6,ncol=2) +
  scale_x_continuous(breaks=seq(4,18,by=2))+
  scale_y_continuous(breaks=0:4,labels=log10labs)+
  coord_cartesian(ylim=c(0,4.5))+
  labs(x="Age in months",y="Luminex Response (MFI-bg)") +
  scale_fill_manual(values=pcols) +
  scale_color_manual(values=pcols) +
  theme_minimal() +
  theme(legend.position = "none")

pcurve
```

## Estimate age-dependent seroprevalence

### Estimate seroprevalence curves with splines in a generalized additive model (GAM)

```
#-----------------------------
# create composite 
# seropositivity indicators
# that use information from
# multiple antigens
#-----------------------------

dgi <- dl %>% 
  filter(antigen %in% c("vsp3","vsp5")) %>%
  select(childid,time,tr,sex,age,agediff,pathogen,antigen,seropos) %>%
  spread(antigen,seropos) %>%
  mutate(seropos=ifelse(vsp3==1|vsp5==1,1,0),
         antigen="vsp3vsp5",
         antigenf="Giardia VSP-3 or VSP-5") %>%
  select(-vsp3,-vsp5)

dcr <- dl %>% 
  filter(antigen %in% c("cp17","cp23")) %>%
  select(childid,time,tr,sex,age,agediff,pathogen,antigen,seropos) %>%
  spread(antigen,seropos) %>%
  mutate(seropos=ifelse(cp17==1|cp23==1,1,0),
         antigen="cp17cp23",
         antigenf="Cryptosporidium Cp17 or Cp23") %>%
  select(-cp17,-cp23)

ds <- dl %>% 
  filter(antigen %in% c("salb","sald")) %>%
  select(childid,time,tr,sex,age,agediff,pathogen,antigen,seropos) %>%
  spread(antigen,seropos) %>%
  mutate(seropos=ifelse(salb==1|sald==1,1,0),
         antigen="salbsald",
         antigenf="Salmonella LPS groups B or D") %>%
  select(-salb,-sald)

dcp <- dl %>% 
  filter(antigen %in% c("p18","p39")) %>%
  select(childid,time,tr,sex,age,agediff,pathogen,antigen,seropos) %>%
  spread(antigen,seropos) %>%
  mutate(seropos=ifelse(p18==1|p39==1,1,0),
         antigen="p18p39",
         antigenf="Campylobacter p18 or p39") %>%
  select(-p18,-p39)


dlp <- bind_rows(dl,dgi,dcr,ds,dcp) %>%
  mutate(antigenf=factor(antigenf,
                         levels=c("Giardia VSP-3 or VSP-5",
                                  "Cryptosporidium Cp17 or Cp23",
                                  "E. histolytica LecA",
                                  "Salmonella LPS groups B or D",
                                  "ETEC LT B subunit",
                                  "Cholera toxin B subunit",
                                  "Campylobacter p18 or p39")) ) %>%
  filter(!is.na(antigenf)) %>%
  select(pathogen,antigen,antigenf,
         childid,time,tr,sex,age,agediff,
         mfi,logmfi,logmfidiff,
         roccut,mixcut,unexp,unexpcut,serocut,serocut_desc,
         posroc,posmix,posunex,seropos) %>%
  arrange(pathogen,antigenf,childid,time)
```

```
## Warning in bind_rows_(x, .id): binding factor and character vector,
## coercing into character vector
```

```
## Warning in bind_rows_(x, .id): binding character and factor vector,
## coercing into character vector
```

```
#-----------------------------
# estimate seroprevalence curves with 
# splines
#-----------------------------
# fit cubic spline over age, separately for each antigen
# estimate approximate simultaneous confidence intervals
# for the fits
library(mgcv)
```

```
## This is mgcv 1.8-27. For overview type 'help("mgcv-package")'.
```

```
pgamfits <- foreach(ab=levels(dlp$antigenf),.combine=rbind) %dopar% {
    pd <- filter(dlp, antigenf==ab)
    gfit <- mgcv::gam(seropos~s(age, bs="cr"),data=pd,family="binomial")
    gsci <- gamCI(m=gfit,newdata=pd,nreps=1000)
    gsci$antigenf <- ab
    return(gsci)
}

# use antilogit to get back to prevalence from the logit link
pgamfits <- pgamfits %>%
  mutate(fit  = 1/(1+exp(-fit)),
         uprP = 1/(1+exp(-uprP)),
         lwrP = 1/(1+exp(-lwrP)),
         uprS = 1/(1+exp(-uprS)),
         lwrS = 1/(1+exp(-lwrS))) %>%
  mutate(antigenf=factor(antigenf,levels=levels(dlp$antigenf)))
```

### Estimate seroprevalence curves with ensemble machine learning

```
#-----------------------------
# estimate curves with 
# ensemble machine learning
# including a library with
# GLM, GAM, Loess, and 
# extreme gradient boosting
#-----------------------------

# detach the mgcv package because
# the ensemble algorithm uses gam()
# from the gam package and there are
# conflicts between them
detach("package:mgcv", unload=TRUE)

set.seed(12345)
pslfits <-  foreach(ab=levels(dlp$antigenf),.combine=rbind) %dopar% {
  pd <- filter(dlp, antigenf==ab)
  slfit <- agecurveAb(Y=pd$seropos,
                      Age=pd$age,
                      id=pd$childid,
                      family="binomial",
                      SL.library = c("SL.glm","SL.gam","SL.xgboost"))
  sld <- data.frame(age=slfit$Age,fit=slfit$pY)
  sld <- sld %>% group_by(age) %>% filter(row_number()==1) %>% ungroup()
  slres <- left_join(pd,sld,by=c("age"))
  return(slres)
}

#-----------------------------
# estimate prevalence by age
# since prevalence is very sparse
# in some strata, cannot use the 
# tmleAb() function above for
# influence-curve based SEs
# because it uses cross-validation
# and sometimes there are folds
# with no events. So, instead
# use an exact binomial approach
#-----------------------------
prevests <- foreach(ab=levels(dlp$antigenf),.combine=rbind) %:%
  foreach(agei=4:17,.combine=rbind) %dopar% {
      pd <- filter(dlp,antigenf==ab & floor(age)==agei)
      mus <- exactprev(pd$seropos)
      res <- data.frame(pathogen=pd$pathogen[1],antigenf=ab,age=agei,N=mus[1],n=mus[2],mean=mus[3],lb=mus[4],ub=mus[5])
      res
    }
```

## Age-dependent seroprevalence curves, all estimates

The spline fits are colored and the highly adaptive ensemble fits are black. The approximate simultaneous confidence bands for the spline fit are shaded.

```
# bright color-blind palette defined in an earlier code chunk
# pcols <- c(cred,corange,cgreen,cteal,cblue,cgrey,cmagent)
# pcols <- c(corange,cred,cmagent,cblue,cteal,cgreen,cgrey)
# pcols <- vircols
pcols <- cbPalette[c(2:4,6,7,5,8,1)]


ppcurve <- ggplot(data=pgamfits,aes(x=age,color=antigenf,fill=antigenf)) +
  # approximate simultaneous CI from spline fit
  geom_ribbon(aes(ymin=lwrS,ymax=uprS),alpha=0.2,color=NA,fill=cgrey) +
  # actual measurements
  # geom_jitter(aes(y=seropos),width=0.2,height=0,color="black",alpha=0.4,size=0.3) +
  # spline fit
  geom_line(aes(y=fit)) +
  # ensemble fit
  geom_line(data=pslfits,aes(x=age,y=fit),col="black") +
  # empirical means 95% CI from the influence curve (averaged for each age)
  geom_pointrange(data=prevests,aes(x=age,y=mean,ymin=lb,ymax=ub),size=0.2,color=cblack)+
  # plot aesthetics
  facet_wrap(~antigenf,nrow=6,ncol=2) +
  scale_x_continuous(breaks=seq(4,18,by=2))+
  scale_y_continuous(breaks=seq(0,1,by=0.2),labels=sprintf("%1.0f",seq(0,1,by=0.2)*100))+
  coord_cartesian(ylim=c(0,1))+
  labs(x="Age in months",y="Seroprevalence (%)") +
  scale_fill_manual(values=pcols) +
  scale_color_manual(values=pcols) +
  theme_minimal() +
  theme(legend.position = "none")

ppcurve
```

## Figure 3-supplement 1, Age-dependent means

Create a more parsimonius plot, that only includes the spline fits but also includes the actual points. Only plot through age 17 months (since n=1 at 18 months).

Exclude Cholera due to cross-reactivity with ETEC and no suggestion that there was cholera transmission in the population at the time of the study.

```
log10labs <- c( 
  expression(10^0),
  expression(10^1),
  expression(10^2),
  expression(10^3),
  expression(10^4)
)

# drop cholera estimates, because of cross-reactivity with ETEC
# re-arrange antigen factor for parsimomy
dplot <- gamfits %>%
  filter(pathogen!="V. cholerae") %>%
  mutate(antigenf=factor(antigenf,levels=c("Giardia VSP-3","Giardia VSP-5",
                                           "Cryptosporidium Cp17","Cryptosporidium Cp23",
                                           "E. histolytica LecA","ETEC LT B subunit",
                                           "Salmonella LPS group B","Salmonella LPS group D",
                                           "Campylobacter p18","Campylobacter p39" )))

# create a variable for faceting panels by pathogen
dplot <- dplot %>%
  mutate(antigenn=ifelse(antigenf %in% c("Giardia VSP-5","Cryptosporidium Cp23","Salmonella LPS group D","Campylobacter p39"),2,1)
  )


# labels for the panels in the plot 
plabs <- dplot %>%
  group_by(pathogen,antigenn) %>%
  select(pathogen,antigenn,antigenf) %>%
  slice(1)


# bright color-blind palette defined in an earlier code chunk
# pcols <- c(cred,corange,cgreen,cteal,cblue,cmagent)
# pcols <- c(corange,cred,cmagent,cblue,cteal,cgreen,cgrey)
# pcols <- vircols[c(1:5,7)]
pcols <- cbPalette[c(2:4,6,7,8)]


pcurve <- ggplot(data=filter(dplot,age<=17),aes(x=age,group=pathogen,color=pathogen,fill=pathogen)) +
  # approximate simultaneous CI from spline fit
  geom_ribbon(aes(ymin=lwrS,ymax=uprS),alpha=0.3,color=NA,fill=cgrey) +
  # actual measurements
  geom_jitter(aes(y=logmfi),width=0.2,height=0,alpha=0.4,size=0.3) +
  # spline fit
  geom_line(aes(y=fit),color="black") +
  # labels
  geom_text(data=plabs,aes(x=4,y=4.55,label=antigenf,hjust=0,vjust=0),color="gray20",size=3.4)+
  # plot aesthetics
  facet_grid(pathogen~antigenn) +
  scale_x_continuous(breaks=seq(4,18,by=2))+
  scale_y_continuous(breaks=0:4,labels=log10labs)+
  scale_fill_manual(values=pcols) +
  scale_color_manual(values=pcols) +
  coord_cartesian(ylim=c(0,4.65))+
  labs(x="Age in months",y="Luminex Response (MFI-bg)",tag="A") +
  theme_minimal(base_size=16) +
  theme(legend.position = "none",
        strip.text=element_blank())
```

Seroprevalence curves

```
# bright color-blind palette defined in an earlier code chunk
# pcols <- c(cred,corange,cgreen,cteal,cblue,cmagent)
# pcols <- c(corange,cred,cmagent,cchartr,cblue,cteal,cgreen,cgrey)
# pcols <- vircols[c(1:5,7)]
pcols <- cbPalette[c(2:4,6,7,8)]

dplot <- pgamfits %>%
  filter(age<=17 & antigen!="cholera")

# labels for the panels in the plot 
pplabs <- dplot %>%
  group_by(antigenf) %>%
  select(pathogen,antigenf) %>%
  slice(1)


ppcurve2 <- ggplot(data=dplot,aes(x=age,color=antigenf,fill=antigenf)) +
  # approximate simultaneous CI from spline fit
  geom_ribbon(aes(ymin=lwrS,ymax=uprS),alpha=0.3,color=NA,fill=cgrey) +
  # actual measurements
  geom_jitter(aes(y=seropos),width=0.3,height=0,alpha=0.3,size=1.5,pch="|") + 
  # spline fit
  geom_line(aes(y=fit),color="black") +
  # labels
  geom_text(data=pplabs,aes(x=4,y=1.03,label=antigenf,hjust=0,vjust=0),color="gray20",size=3.4)+
  # plot aesthetics
  facet_grid(antigenf~.) +
  scale_fill_manual(values=pcols) +
  scale_color_manual(values=pcols) +
  scale_x_continuous(breaks=seq(4,18,by=2))+
  scale_y_continuous(breaks=seq(0,1,by=0.2),labels=sprintf("%1.0f",seq(0,1,by=0.2)*100))+
  coord_cartesian(ylim=c(0,1.04))+
  labs(x="Age in months",y="Seroprevalence (%)",tag="B") +
  theme_minimal(base_size=16) +
  theme(legend.position = "none",
        strip.text=element_blank())
```

Composite figure

\*\* This is Figure 3 - supplement 1 in the manuscript \*\*

```
# labels for the panels in the plot 
pcurve_composite_kenya <- grid.arrange(pcurve,ppcurve2,ncol=2,nrow=1,widths=c(2,1.1))
```

```
# save PDF and TIFF versions
ggsave(here::here("figs","Fig3sup1-asembo-ab-agecurves.pdf"),plot=pcurve_composite_kenya,device=cairo_pdf,width=9,height=14)
ggsave(here::here("figs","Fig3sup1-asembo-ab-agecurves.TIFF"),plot=pcurve_composite_kenya,device="tiff",width=9,height=14)
```

## Save fitted estimates for later use, Kenya

```
# spline fits of geometric means
saveRDS(gamfits,file=here::here("output","asembo-enteric-ab-gamfits-means.rds"))
# ensemble fits of geometric means
saveRDS(slfits,file=here::here("output","asembo-enteric-ab-slfits-means.rds"))
# spline fits of age-dependent seroprevalence
saveRDS(pgamfits,file=here::here("output","asembo-enteric-ab-gamfits-seroprev.rds"))
# spline fits of age-dependent seroprevalence
saveRDS(pslfits,file=here::here("output","asembo-enteric-ab-slfits-seroprev.rds"))
```

# Tanzania

## Load the formatted data from Tanzania

```
#-----------------------------
# load the formatted data
# with seropositivity indicators
# created with:
# kongwa-enteric-ab-data-format.Rmd -->
# Fig1sup1sup2-kongwa-ab-distributions.Rmd
#-----------------------------
dl <- readRDS(here::here("data","kongwa_analysis2.rds"))

# list the enteric antigens and formatted labels for them
mbavars <- c("vsp3","vsp5","cp17","cp23","leca","salb","sald","etec","cholera","p18","p39")

mbalabs <- c("Giardia VSP-3","Giardia VSP-5","Cryptosporidium Cp17","Cryptosporidium Cp23",
             "E. histolytica LecA","Salmonella LPS B","Salmonella LPS D","ETEC LT B subunit","Cholera toxin B subunit","Campylobacter p18","Campylobacter p39")
dl <- dl %>% filter(antigen %in% mbavars) %>% mutate(antigenf=droplevels(antigenf))
```

## Estimate age-dependent mean antibody levels

### Estimate curves with splines in a generalized additive model (GAM)

```
# fit cubic spline over age, separately for each antigen
# estimate approximate simultaneous confidence intervals
# for the fits
library(mgcv)
```

```
## This is mgcv 1.8-27. For overview type 'help("mgcv-package")'.
```

```
gamfits <- foreach(ab=levels(dl$antigenf),.combine=rbind) %dopar% {
    pd <- filter(dl, antigenf==ab)
    gfit <- gam(logmfi~s(age, bs="cr",k=9),data=pd)
    gsci <- gamCI(m=gfit,newdata=pd,nreps=1000)
    gsci$antigen <- ab
    return(gsci)
  }
```

## Estimate curves with ensemble machine learning

```
#-----------------------------
# estimate curves with 
# ensemble machine learning
# including a library with
# GLM, GAM, Loess, and 
# extreme gradient boosting
#-----------------------------

# detach the mgcv package because
# the ensemble algorithm uses gam()
# from the gam package and there are
# conflicts between them
detach("package:mgcv", unload=TRUE)

# load the tmleAb package, at this time
# only available through github: 
# devtools::install_github("ben-arnold/tmleAb")
library(tmleAb)
set.seed(123)
slfits <-  foreach(ab=levels(dl$antigenf),.combine=rbind) %dopar% {
  pd <- filter(dl, antigenf==ab)
  slfit <- agecurveAb(Y=pd$logmfi,
                      Age=pd$age,
                      id=pd$id,
                      SL.library = c("SL.glm","SL.gam","SL.loess","SL.xgboost"))
  sld <- data.frame(age=slfit$Age,fit=slfit$pY)
  sld <- sld %>% group_by(age) %>% filter(row_number()==1) %>% ungroup()
  slres <- left_join(pd,sld,by=c("age"))
  return(slres)
}

#-----------------------------
# estimate means by age in 
# years (1-9)
# to get 95% CIs through the
# influence curve
#-----------------------------

tmlemeans <- foreach(ab=levels(dl$antigenf),.combine=rbind) %:%
  foreach(agei=1:9,.combine=rbind) %dopar% {
    pd <- filter(dl,antigenf==ab & floor(age)==agei)
    mu <- tmleAb(Y=pd$logmfi,id=pd$clusterid,SL.library="SL.mean")
    di <- data.frame(pathogen=pd$pathogen[1],antigenf=ab,age=agei,mean=mu$psi,se=mu$se,lb=mu$lb,ub=mu$ub)
    di
  }
```

### Age-dependent mean antibody curves, all estimates

Plot all of the estimates together. The spline fits are colored and the highly adaptive ensemble fits are grey. Empirical means with 95% CIs (averaged by year) are black. The approximate simultaneous confidence bands for the spline fit are shaded. All of the plots shift the age in years completed by 0.5, assuming they are uniformly distributed between rounded values.

```
log10labs <- c( 
  expression(10^0),
  expression(10^1),
  expression(10^2),
  expression(10^3),
  expression(10^4)
)

# custom color blind color palette is in the preamble chunck
# pcols <- c(cred,corange,cgreen,cteal,cblue,cmagent,cgrey)
# pcols <- vircols
pcols <- cbPalette[c(2:4,6,7,5,8)]

pcurveall <- ggplot(data=gamfits,aes(x=age+0.5,group=pathogen,color=pathogen,fill=pathogen)) +
  # approximate simultaneous CI from spline fit
  geom_ribbon(aes(ymin=lwrS,ymax=uprS),alpha=0.2,color=NA,fill=cgrey) +
  # actual measurements
  # geom_jitter(aes(y=logmfi),width=0.2,height=0,color="black",alpha=0.4,size=0.3) +
  # ensemble fit
  geom_line(data=slfits,aes(x=age+0.5,y=fit),color=cgrey) +
  # spline fit
  geom_line(aes(y=fit)) +
  # empirical means 95% CI from the influence curve (averaged for each age)
  geom_pointrange(data=tmlemeans,aes(x=age+0.5,y=mean,ymin=lb,ymax=ub),size=0.2,color=cblack)+
  # plot aesthetics
  facet_wrap(~antigenf,nrow=6,ncol=2) +
  scale_x_continuous(limits=c(0,9),breaks=seq(0,8,by=2))+
  scale_y_continuous(breaks=0:4,labels=log10labs)+
  coord_cartesian(ylim=c(0,4.5))+
  labs(x="Age in years",y="Luminex Response (MFI-bg)") +
  scale_fill_manual(values=pcols) +
  scale_color_manual(values=pcols) +
  theme_minimal() +
  theme(legend.position = "none")

pcurveall
```

```
## Warning: Removed 3892 rows containing missing values (geom_path).

## Warning: Removed 3892 rows containing missing values (geom_path).
```

```
## Warning: Removed 11 rows containing missing values (geom_pointrange).
```

## Estimate age-dependent seroprevalence

### Estimate seroprevalence curves with splines in a generalized additive model (GAM)

```
#-----------------------------
# subset the data to antigens
# with seropositivity estimates
# In Tanzania, due to the older
# age of the cohort, the only
# antigens that have reliable
# cutoff values are those with
# ROC cutoffs
#-----------------------------
dlp <- dl %>%
  mutate(seropos=ifelse(!is.na(posroc),posroc,posmix),
         serocut=ifelse(!is.na(posroc),roccut,mixcut),
         serocut_desc=ifelse(!is.na(posroc),"ROC","Mixture Model")) %>%
  filter(!is.na(seropos)) %>%
  mutate(antigenf=droplevels(antigenf))


#-----------------------------
# create composite 
# seropositivity indicators
# that use information from
# multiple antigens
#-----------------------------

dgi <- dlp %>% 
  filter(antigen %in% c("vsp3","vsp5")) %>%
  select(svy,year,vilid,clusterid,id,aztr,age,pathogen,antigen,seropos) %>%
  spread(antigen,seropos) %>%
  mutate(seropos=ifelse(vsp3==1|vsp5==1,1,0),
         antigen="vsp3vsp5",
         antigenf="Giardia VSP-3 or VSP-5") %>%
  select(-vsp3,-vsp5)

dcr <- dlp %>% 
  filter(antigen %in% c("cp17","cp23")) %>%
  select(svy,year,vilid,clusterid,id,aztr,age,pathogen,antigen,seropos) %>%
  spread(antigen,seropos) %>%
  mutate(seropos=ifelse(cp17==1|cp23==1,1,0),
         antigen="cp17cp23",
         antigenf="Cryptosporidium Cp17 or Cp23") %>%
  select(-cp17,-cp23)


dlp <- bind_rows(dlp,dgi,dcr) %>%
  mutate(antigenf=factor(antigenf,
                         levels=c("Giardia VSP-3 or VSP-5",
                                  "Cryptosporidium Cp17 or Cp23",
                                  "E. histolytica LecA")) ) %>%
  filter(!is.na(antigenf)) %>%
  select(pathogen,antigen,antigenf,
         svy,year,vilid,clusterid,id,aztr,age,
         roccut,mixcut,serocut,serocut_desc,
         posroc,posmix,seropos) %>%
  arrange(pathogen,antigenf,svy,year,clusterid,id,age)
```

```
#-----------------------------
# estimate seroprevalence curves with 
# splines
#-----------------------------

# fit cubic spline over age, separately for each antigen
# estimate approximate simultaneous confidence intervals
# for the fits
library(mgcv)
```

```
## This is mgcv 1.8-27. For overview type 'help("mgcv-package")'.
```

```
pgamfits <- foreach(ab=c("Giardia VSP-3 or VSP-5","Cryptosporidium Cp17 or Cp23","E. histolytica LecA"),.combine=rbind) %dopar% {
    pd <- filter(dlp, antigenf==ab)
    gfit <- mgcv::gam(seropos~s(age, bs="cr",k=9),data=pd,family="binomial")
    gsci <- gamCI(m=gfit,newdata=pd,nreps=1000)
    gsci$antigen <- ab
    return(gsci)
}

# use antilogit to get back to prevalence from the logit link
pgamfits <- pgamfits %>%
  mutate(fit  = 1/(1+exp(-fit)),
         uprP = 1/(1+exp(-uprP)),
         lwrP = 1/(1+exp(-lwrP)),
         uprS = 1/(1+exp(-uprS)),
         lwrS = 1/(1+exp(-lwrS))) %>%
  mutate(antigenf=factor(antigenf,levels=levels(dlp$antigenf)))
```

### Estimate seroprevalence curves with ensemble machine learning

```
#-----------------------------
# estimate curves with 
# ensemble machine learning
# including a library with
# GLM, GAM, Loess, and 
# extreme gradient boosting
#-----------------------------

# detach the mgcv package because
# the ensemble algorithm uses gam()
# from the gam package and there are
# conflicts between them
detach("package:mgcv", unload=TRUE)

set.seed(12345)
pslfits <-  foreach(ab=c("Giardia VSP-3 or VSP-5","Cryptosporidium Cp17 or Cp23","E. histolytica LecA"),.combine=rbind) %dopar% {
  pd <- filter(dlp, antigenf==ab)
  slfit <- agecurveAb(Y=pd$seropos,
                      Age=pd$age,
                      id=pd$id,
                      family="binomial",
                      SL.library = c("SL.glm","SL.gam","SL.xgboost"))
  sld <- data.frame(age=slfit$Age,fit=slfit$pY)
  sld <- sld %>% group_by(age) %>% filter(row_number()==1) %>% ungroup()
  slres <- left_join(pd,sld,by=c("age"))
  return(slres)
}


#-----------------------------
# estimate means by age in 
# years (1-9)
# to get 95% CIs through the
# influence curve
#-----------------------------

tmlemeansp <- foreach(ab=c("Giardia VSP-3 or VSP-5","Cryptosporidium Cp17 or Cp23","E. histolytica LecA"),.combine=rbind) %:%
  foreach(agei=1:9,.combine=rbind) %dopar% {
    pd <- filter(dlp,antigenf==ab & floor(age)==agei)
    mu <- tmleAb(Y=pd$seropos,id=pd$clusterid,SL.library="SL.mean")
    di <- data.frame(pathogen=pd$pathogen[1],antigenf=ab,age=agei,mean=mu$psi,se=mu$se,lb=mu$lb,ub=mu$ub)
    di
  }
```

### Age-dependent seroprevalence curves, all estimates

Plot all of the estimates together. The spline fits are colored and the highly adaptive ensemble fits are grey. Empirical means with 95% CIs (averaged by year) are black. The approximate simultaneous confidence bands for the spline fit are shaded. Ages are shifted by 0.5 years because in the Kongwa study ages are years completed (e.g., an individual age 1 could be anywhere from 1.0 to 1.9 years old, and 1.5 is approximately unbiased).

```
# bright color-blind palette defined in an earlier code chunk
# pcols <- c(cred,corange,cgreen,cteal,cblue,cmagent)
# pcols <- vircols
pcols <- cbPalette[c(2:4,6,7,5,8)]

ppcurve <- ggplot(data=pgamfits,aes(x=age+0.5,color=pathogen,fill=pathogen)) +
  # approximate simultaneous CI from spline fit
  geom_ribbon(aes(ymin=lwrS,ymax=uprS),alpha=0.2,color=NA,fill=cgrey) +
  # actual measurements
  # geom_jitter(aes(y=posmix),width=0.2,height=0,color="black",alpha=0.4,size=0.3) +
   # ensemble fit
  geom_line(data=pslfits,aes(x=age+0.5,y=fit),color=cgrey) +
  # spline fit
  geom_line(aes(y=fit)) +
  # empirical means 95% CI from the influence curve (averaged for each age)
  geom_pointrange(data=tmlemeansp,aes(x=age+0.5,y=mean,ymin=lb,ymax=ub),size=0.2,color=cblack)+
  # plot aesthetics
  facet_wrap(~antigenf,nrow=4,ncol=1) +
  scale_x_continuous(limits=c(0,10),breaks=seq(0,10,by=2))+
  scale_y_continuous(breaks=seq(0,1,by=0.2),labels=sprintf("%1.0f",seq(0,1,by=0.2)*100))+
  coord_cartesian(ylim=c(0,1))+
  labs(x="Age in years",y="Seroprevalence (%)") +
  scale_fill_manual(values=pcols) +
  scale_color_manual(values=pcols) +
  theme_minimal(base_size=16) +
  theme(legend.position = "none")

ppcurve
```

## Figure 3 - supplement 2, age dependent means

Create a more parsimonius plot, that only includes the spline fits but also includes the actual points. Exclude *V. cholera* beta toxin response because of difficulty in interpretation because of cross-reactivity with ETEC heat labile toxin beta subunit.

```
log10labs <- c( 
  expression(10^0),
  expression(10^1),
  expression(10^2),
  expression(10^3),
  expression(10^4)
)

# drop cholera estimates, because of cross-reactivity with ETEC
# re-arrange antigen factor for parsimomy
dplot <- gamfits %>%
  filter(pathogen!="V. cholerae") %>%
  mutate(antigenf=factor(antigenf,levels=c("Giardia VSP-3","Giardia VSP-5",
                                           "Cryptosporidium Cp17","Cryptosporidium Cp23",
                                           "E. histolytica LecA","ETEC LT B subunit",
                                           "Salmonella LPS group B","Salmonella LPS group D",
                                           "Campylobacter p18","Campylobacter p39" )))

# create a variable for faceting panels by pathogen
dplot <- dplot %>%
  mutate(antigenn=ifelse(antigenf %in% c("Giardia VSP-5","Cryptosporidium Cp23","Salmonella LPS group D","Campylobacter p39"),2,1)
  )


# labels for the panels in the plot 
plabs <- dplot %>%
  group_by(pathogen,antigenn) %>%
  select(pathogen,antigenn,antigenf) %>%
  slice(1)


# custom color blind color palette is in the preamble chunck
# pcols <- c(cred,corange,cgreen,cteal,cblue,cmagent)
# pcols <- vircols[c(1:5,7)]
pcols <- cbPalette[c(2:4,6,7,8)]

pcurve <- ggplot(data=dplot,aes(x=age+0.5,color=pathogen,fill=pathogen)) +
  # approximate simultaneous CI from spline fit
  geom_ribbon(aes(ymin=lwrS,ymax=uprS),alpha=0.4,color=NA,fill=cgrey) +
  # actual measurements
  geom_jitter(aes(y=logmfi),width=0.2,height=0,alpha=0.4,size=0.3) +
  # spline fit
  geom_line(aes(y=fit),color="black") +
  # labels
  geom_text(data=plabs,aes(x=0,y=4.55,label=antigenf,hjust=0,vjust=0),color="gray20",size=3.4)+
  # plot aesthetics
  facet_grid(pathogen~antigenn) +
  scale_x_continuous(limits=c(0,10),breaks=seq(0,10,by=2))+
  scale_y_continuous(breaks=0:4,labels=log10labs)+
  coord_cartesian(ylim=c(0,4.65))+
  labs(x="Age in years",y="Luminex Response (MFI-bg)",tag="A") +
  scale_fill_manual(values=pcols) +
  scale_color_manual(values=pcols) +
  theme_minimal(base_size=16) +
  theme(legend.position = "none",
        strip.text=element_blank())
```

Seroprevalence curves

```
# include extra factor levels for plot faceting
dplot2 <- pgamfits %>%
  mutate(antigenf=factor(antigenf,
                         levels=c(levels(antigenf),
                                  "Salmonella LPS groups B or D",
                                  "ETEC LT B subunit", 
                                  "Campylobacter p18 or p39"))
  )

# labels for the panels in the plot 
pplabs <- data.frame(pathogen=c("Giardia","Cryptosporidium","E. histolytica","Salmonella","ETEC","Campylobacter"),
                     antigenf=levels(dplot2$antigenf))

# bright color-blind palette defined in an earlier code chunk
# pcols <- c(cred,corange,cgreen,cteal,cblue,cmagent)
# for reasons unclear, fill for Giardia is mapping to arg #5 in pcols
# tired of thrashing around with it, so just patching it here:
# pcols <- c(cred,corange,cgreen,cteal,cred,cmagent)
# pcols <- vircols[c(1:4,1,5:7)]
pcols <- cbPalette[c(2:4,6,7,8)]


ppcurve2 <- ggplot(data=dplot2,aes(x=age+0.5,color=antigenf,fill=antigenf)) +
  # approximate simultaneous CI from spline fit
  geom_ribbon(aes(ymin=lwrS,ymax=uprS),alpha=0.3,color=NA,fill=cgrey) +
  # actual measurements
  geom_jitter(aes(y=seropos),width=0.3,height=0,alpha=0.3,size=1.5,pch="|") + 
  # spline fit
  geom_line(aes(y=fit),color="black") +
  # labels
  geom_text(data=pplabs,aes(x=0,y=1.03,label=antigenf,hjust=0,vjust=0),color="gray20",size=3.4)+
  # plot aesthetics
  facet_wrap(antigenf~.,drop=FALSE,ncol=1,nrow=6) +
  scale_x_continuous(limits=c(0,10),breaks=seq(0,10,by=2))+
  scale_y_continuous(breaks=seq(0,1,by=0.2),labels=sprintf("%1.0f",seq(0,1,by=0.2)*100))+
  coord_cartesian(ylim=c(0,1.04))+
  labs(x="Age in years",y="Seroprevalence (%)",tag="B") +
  scale_fill_manual(values=pcols) +
  scale_color_manual(values=pcols) +
  theme_minimal(base_size=16) +
  theme(legend.position = "none",
        strip.text=element_blank())
```

Composite figure

\*\* This is Figure 3 - supplement 2 in the manuscript \*\*

```
# labels for the panels in the plot 
pcurve_composite_tanzania <- grid.arrange(pcurve,ppcurve2,ncol=2,nrow=1,widths=c(2,1.1))
```

```
## Warning: Removed 16348 rows containing missing values (geom_point).
```

```
# save PDF and TIFF versions
ggsave(here::here("figs","Fig3sup2-kongwa-ab-agecurves.pdf"),plot=pcurve_composite_tanzania,device=cairo_pdf,width=9,height=14)
ggsave(here::here("figs","Fig3sup2-kongwa-ab-agecurves.TIFF"),plot=pcurve_composite_tanzania,device="tiff",width=9,height=14)
```

### Numerical seroprevalence estimates to report in the manuscript.

```
# grab predicted means from cubic splines very close to each year of age
pests <- pgamfits %>%
  mutate(agey = floor(age)) %>%
  filter(age+0.02 > agey & age-0.02 < agey) %>%
  group_by(antigenf) %>%
  filter(!duplicated(agey)) %>%
  arrange(antigenf,agey) %>%
  select(antigenf,agey,prev=fit,prev_min95=lwrS,prev_max95=uprS)

pests
```

## Save fitted estimates for later use, Tanzania

```
# spline fits of geometric means
saveRDS(gamfits,file=here::here("output","kongwa-enteric-ab-gamfits-means.rds"))
# ensemble fits of geometric means
saveRDS(slfits,file=here::here("output","kongwa-enteric-ab-slfits-means.rds"))
# spline fits of age-dependent seroprevalence
saveRDS(pgamfits,file=here::here("output","kongwa-enteric-ab-gamfits-seroprev.rds"))
# spline fits of age-dependent seroprevalence
saveRDS(pslfits,file=here::here("output","kongwa-enteric-ab-slfits-seroprev.rds"))
```

# Session Info

```
sessionInfo()
```

```
## R version 3.5.3 (2019-03-11)
## Platform: x86_64-apple-darwin15.6.0 (64-bit)
## Running under: macOS High Sierra 10.13.6
## 
## Matrix products: default
## BLAS: /Library/Frameworks/R.framework/Versions/3.5/Resources/lib/libRblas.0.dylib
## LAPACK: /Library/Frameworks/R.framework/Versions/3.5/Resources/lib/libRlapack.dylib
## 
## locale:
## [1] en_US.UTF-8/en_US.UTF-8/en_US.UTF-8/C/en_US.UTF-8/en_US.UTF-8
## 
## attached base packages:
## [1] parallel  stats     graphics  grDevices utils     datasets  methods  
## [8] base     
## 
## other attached packages:
##  [1] tmleAb_0.3.4          nlme_3.1-137          doParallel_1.0.11    
##  [4] iterators_1.0.9       foreach_1.4.4         kableExtra_0.8.0.0001
##  [7] gridExtra_2.3         viridis_0.5.1         viridisLite_0.3.0    
## [10] forcats_0.3.0         stringr_1.4.0         dplyr_0.8.0.1        
## [13] purrr_0.3.2           readr_1.1.1           tidyr_0.8.3          
## [16] tibble_2.1.1          ggplot2_3.1.1         tidyverse_1.2.1      
## [19] here_0.1             
## 
## loaded via a namespace (and not attached):
##  [1] Rcpp_1.0.1       lubridate_1.7.4  lattice_0.20-38  assertthat_0.2.1
##  [5] rprojroot_1.3-2  digest_0.6.18    psych_1.8.4      R6_2.4.0        
##  [9] cellranger_1.1.0 plyr_1.8.4       backports_1.1.4  evaluate_0.13   
## [13] httr_1.4.0       pillar_1.4.0     rlang_0.3.4      lazyeval_0.2.2  
## [17] readxl_1.1.0     rstudioapi_0.9.0 Matrix_1.2-15    rmarkdown_1.12  
## [21] splines_3.5.3    foreign_0.8-71   munsell_0.5.0    broom_0.4.4     
## [25] compiler_3.5.3   modelr_0.1.2     xfun_0.6         pkgconfig_2.0.2 
## [29] mnormt_1.5-5     htmltools_0.3.6  tidyselect_0.2.5 codetools_0.2-16
## [33] crayon_1.3.4     withr_2.1.2      grid_3.5.3       jsonlite_1.6    
## [37] gtable_0.3.0     magrittr_1.5     scales_1.0.0     cli_1.1.0       
## [41] stringi_1.4.3    reshape2_1.4.3   xml2_1.2.0       tools_3.5.3     
## [45] glue_1.3.1       hms_0.4.2        yaml_2.2.0       colorspace_1.3-2
## [49] rvest_0.3.2      knitr_1.22       haven_2.1.0
```
