# Supplementary material for: Enteropathogen antibody dynamics and force of infection among children in low-resource settings
Source: eLife. 2019 Aug 19;8:e45594. doi: 10.7554/eLife.45594 (PMC6746552; doi:10.7554/eLife.45594)
Supplement: Supplementary file 7. [file elife-45594-supp7.zip › SIFile7.html]

Enteropathogen antibody dynamics and force of infection among children in low-resource settings


Code 

- Show All Code
- Hide All Code

# Enteropathogen antibody dynamics and force of infection among children in low-resource settings

### Supplementary Information File 7. Estimation of force of infection assuming cross-sectional (“current status”) data in Kenya. Figure 6.

# Summary

For enteric antibodies with defined cutoffs for seropositivity and approximately monotonically increasing age-dependent seroprevalence, estimate the hazard of seroconversion, or seroconversion rate, which is one measure of a pathogen’s force of infection.

This analysis focuses on the Asembo, Kenya cohort and estimates force of infection with three separate approaches: constant hazard, constant hazard with seroreversion, and age-varying hazard modeled with semiparametric cubic splines. At the end of the notebook, the estimates from cross-sectional estimators are compared to the nonparametric prospective analysis of the same parameter, which is Figure 6 in the manuscript.

# Script preamble

```
#-----------------------------
# preamble
#-----------------------------
library(here)
```

```
## here() starts at /Users/benarnold/enterics-seroepi
```

```
here::here()
```

```
## [1] "/Users/benarnold/enterics-seroepi"
```

```
# load packages
library(tidyverse)
```

```
## ── Attaching packages ────────────────────────────── tidyverse 1.2.1 ──
```

```
## ✔ ggplot2 3.1.1       ✔ purrr   0.3.2  
## ✔ tibble  2.1.1       ✔ dplyr   0.8.0.1
## ✔ tidyr   0.8.3       ✔ stringr 1.4.0  
## ✔ readr   1.1.1       ✔ forcats 0.3.0
```

```
## ── Conflicts ───────────────────────────────── tidyverse_conflicts() ──
## ✖ dplyr::filter() masks stats::filter()
## ✖ dplyr::lag()    masks stats::lag()
```

```
library(viridis)
```

```
## Loading required package: viridisLite
```

```
library(mgcv)
```

```
## Loading required package: nlme
```

```
## 
## Attaching package: 'nlme'
```

```
## The following object is masked from 'package:dplyr':
## 
##     collapse
```

```
## This is mgcv 1.8-27. For overview type 'help("mgcv-package")'.
```

```
library(grid)

# set up for parallel computing
# configure for a laptop (use only 3 cores)
library(foreach)
```

```
## 
## Attaching package: 'foreach'
```

```
## The following objects are masked from 'package:purrr':
## 
##     accumulate, when
```

```
library(doParallel)
```

```
## Loading required package: iterators
```

```
## Loading required package: parallel
```

```
registerDoParallel(cores=3)

# bright color blind palette:  https://personal.sron.nl/~pault/ 
cblack <- "#000004FF"
cblue <- "#3366AA"
cteal <- "#11AA99"
cgreen <- "#66AA55"
cchartr <- "#CCCC55"
cmagent <- "#992288"
cred <- "#EE3333"
corange <- "#EEA722"
cyellow <- "#FFEE33"
cgrey <- "#777777"

# safe color blind palette
cbPalette <- c("#999999", "#E69F00", "#56B4E9", "#009E73", "#F0E442", "#0072B2", "#D55E00", "#CC79A7")

# viridis color palette
vircols <- viridis(n=4,alpha=1,begin=0.2,end=0.97)
```

```
#-----------------------------
# load the formatted data
# with seropositivity indicators
# that are grouped into 
# composite antigens
# created with:
# asembo-enteric-ab-data-format.Rmd -->
# Fig1sup3-asembo-ab-distributions.Rmd -->
# asembo-enteric-ab-agecurves.Rmd
#-----------------------------
d <- readRDS(here::here("output","asembo-enteric-ab-gamfits-seroprev.rds"))

#--------------------------------
# exclude cholera beta toxin because
# of difficulty in its interpretation
# because of cross-reactivity with ETEC
#--------------------------------
dfoi <- d %>%
  filter(!antigen %in% c("cholera")) %>%
  mutate(antigenf=droplevels(antigenf))

#--------------------------------
# filter to children measured longitudinally
# to ensure the identical analysis population
#--------------------------------
dfoi <- dfoi %>%
  group_by(childid,antigen) %>%
  mutate(nobs=n()) %>%
  filter(nobs>1) %>%
  ungroup()
```

# Force of infection from cross-sectional seroprevalence curves

Suppose \(T\_1\) denotes the time of actual seroconversion. For enteric infections it is possible and in some cases likely for antibody response to wane over time and for individuals to sero-revert (move from being seropositive to seronegative). Let \(T\_2\) denote the time of seroreversion with \(T\_2 > T\_1\). We never observe \(T\_1\) or \(T\_2\) in a cross-sectional survey, but we do observe \(Y = I(T\_1 ≤ A < T\_2)\), an indicator that the person seroconverted before age \(A\) but has not sero-reverted. Let \(W\) be a set of additional characteristics. For a single individual we thus observe the following random variable: \(O = (Y= I(T\_1 ≤ A ≤ T\_2) , A, W)\).

Note that: \[\begin{eqnarray\*}
P(Y = 1 | A, W) & = & P(T\_1 \leq A < T\_2 | A, W) \\
& = & P(T\_1 \leq A, T\_2 > A | A, W) \\
& = & [ P(T\_1 \leq A, T\_2 > A | A, W) + \\
& & \qquad P(T\_1 < A, T\_2 \leq A | A, W) ] - P(T\_1 < A, T\_2 \leq A | A, W)\\
& = & P(T\_1 \leq A | A, W) - P(T\_2 \leq A | A, W)
\end{eqnarray\*}\]

Thus the age-dependent seroprevalence curve is the difference between the cumulative distribution functions of seroconversion times and seroreversion times. In the special case where seroreversion is unlikely during the age window, \(P(Y=1 | A, W) = P(T\_1 ≤ A | A, W)\). This is classic “current status” data – an extreme example of interval censored data in survival analysis. If an individual has seroconverted \((Y= 1)\) we know the event happened sometime between birth and their observed age \(A\) (left censored); if the individual has not seroconverted \((Y= 0)\), they are right-censored.

The age-specific force of infection is estimated as the hazard of seroconverting at age \(A=a\): \(\lambda(a) = \frac{F'(a)}{1-F(a)}\), where \(F(a) = P(Y|A=a)\) is the proportion of the population who are seropositive at age \(a\) and \(F'(a)\) is the derivative of \(F(a)\) with respect to \(a\). Key assumptions include stationarity/homogeneity (i.e., no cohort effects) and that there is no sero-reversion. We know the latter to be false in some cases of enteric pathogens, so estimates in that case would be lower-bounds of a pathogen’s force of infection.

### Confirm approximate monotonicity

Quick plot to confirm approximate monotonicity assumptions.

```
pcurve <- ggplot(data=dfoi,aes(x=age)) +
  geom_smooth(aes(y=seropos),se=FALSE,method="loess",color=vircols[1])+
  # plot aesthetics
  facet_wrap(~antigenf,nrow=6,ncol=2) +
  scale_x_continuous(breaks=seq(4,18,by=2))+
  scale_y_continuous(breaks=seq(0,1,by=0.2))+
  labs(x="Age in months",y="Seroprevalence") +
  theme_minimal() +
  theme(legend.position = "none")

pcurve
```

# Parametric estimators

## Exponential model

If we assume a constant force of infection (\(\lambda\)), equivalent to assuming an exponential model, then it can be shown that a generalized linear model with a complementary log-log link fit with current status, age-prevalence data is equivalent to an exponential proportional hazards model (*Jewell and van der Laan 1995*).

\(\log - \log(1-P(Y|A,W)) = \log \lambda + \log A + \beta W\)

Moroever, this model is also equivalent to a catalytic, SIR model with a single, constant rate parameter (*Hens et al. 2010*; *Hens et al. 2012*).

```
glmfoi <- foreach(ab=levels(dfoi$antigenf),.combine=rbind) %dopar% {
    pd <- filter(dfoi, antigenf==ab)
    gfit <- glm(seropos~1,offset=log(age),data=pd,family=binomial(link="cloglog"))
    gsum <- summary(gfit)
    lambda <- as.numeric(exp(gfit$coefficients))
    log_lambda_se  <- sqrt(gsum$cov.unscaled)
    lambda_lb <- as.numeric(exp(gfit$coefficients - 1.96*log_lambda_se))
    lambda_ub <- as.numeric(exp(gfit$coefficients + 1.96*log_lambda_se))
    di <- data.frame(antigenf=ab,lambda,lambda_lb,lambda_ub)
    return(di)
}

knitr::kable(glmfoi,digits=3,
             caption="Average force of infection estimated from a cross-sectional seroprevalence curve, exponential (constant rate) model",
             col.names = c("Pathogen","Incidence per month","min95","max95"),
             row.names = FALSE)
```

Average force of infection estimated from a cross-sectional seroprevalence curve, exponential (constant rate) model

| Pathogen | Incidence per month | min95 | max95 |
| --- | --- | --- | --- |
| Giardia VSP-3 or VSP-5 | 0.025 | 0.020 | 0.030 |
| Cryptosporidium Cp17 or Cp23 | 0.044 | 0.037 | 0.052 |
| E. histolytica LecA | 0.005 | 0.004 | 0.008 |
| Salmonella LPS groups B or D | 0.011 | 0.008 | 0.015 |
| ETEC LT B subunit | 0.243 | 0.212 | 0.279 |
| Campylobacter p18 or p39 | 0.301 | 0.259 | 0.349 |

## Estimates from a reversible catalytic model

For some infectious diseases, like malaria, it is common to fit a reversible catalytic model to the age-seroprevalence curve (*Corran et al 2007*). The model assumes a constant rate of seroconversion, \(\lambda\), (akin to an parametric exponential survival model), but also allows for a constant seroreversion rate, \(\rho\). \[\begin{equation}
P(Y=1|A) = \frac{\lambda}{\lambda + \rho} \left[1 - \exp( - (\lambda + \rho)\cdot A ) \right]
\end{equation}\]

Above, we showed that a cross-sectional seroprevalence measurement is equal to the difference in the cumulative distribution functions of seroconversion times and seroreversion times: \(P(Y=1 | A, W) = P(T\_1 ≤ A | A, W) - P(T\_2 ≤ A | A, W)\). This means there is no information about the joint distribution of \(T\_2\) and \(T\_1\), which is required to estimate seroreversion. Therefore, we will estimate the seroconversion rate under the model assuming a range of fixed seroreversion rates (to our knowledge, there are no good estimates of seroreversion rates for the enteric pathogens included in this study). We use the sero-reversion rates estimated prospectively in the cohort. Notice that since the seroreversion rates are 0 for both ETEC and *Campylobacter*, that the rate estimates from the SIS model are essentially identical to the estimates from the exponential proportional hazards model (above), which is a useful internal validity check since they should be the same.

```
#--------------------------------
# log likelihood function for reversible catalytic 
# model with a fixed seroreversion rate
#--------------------------------
Lrcm <- function(h,r,data) {
    # h    : \lambda seroconversion rate (to be estimtated)
    # r    : \rho seroreversion rate (fixed)
    # data  : data frame with 1 row per age group. cols = age / n / k
    h <- rep(h,nrow(data))
    r <- rep(r,nrow(data))
    t <- data[,1]
    n <- data[,2]
    k <- data[,3]
    p <- h/(r+h)*(1-exp(-(r+h)*t))
    # negative log likelihood function (to minimize with optim)
    sum( - (k)*log(p) - (n-k)*log(1-p) )
}

# reversible catalytic model probability function
# (not used)
rcp <- function(h,r,t) h/(r+h)*(1-exp(-(r+h)*t))

#--------------------------------
# Add up the person-time at risk
# for each child and create indicators of 
# seroreversion using the longitudinal
# data. These are used below in the
# reversible catalytic model to
# provide additional information
# about sero-reversion, which is
# technically not present in a 
# cross-sectional survey.
#--------------------------------
dsr <- readRDS(here::here("data","asembo_analysis2.rds"))
dsr <- dsr %>%
  group_by(antigen,antigenf,childid) %>%
  mutate(measA=ifelse(time=="A",1,0),
         measA=max(measA),
         measB=ifelse(time=="B",1,0),
         measB=max(measB),
         seroposA=ifelse(time=="A",seropos,NA),
         seroposA=max(seroposA,na.rm=TRUE),
         seroposB=ifelse(time=="B",seropos,NA),
         seroposB=max(seroposB,na.rm=TRUE),
         seror=ifelse(seroposB==0 & seroposA==1,1,0),
  ) %>%
  filter(measA==1 & measB==1) %>%
  filter(pathogen!="V. cholerae")

dsr2 <- dsr %>%
  ungroup() %>% 
  group_by(pathogen,childid) %>%
  mutate(seror=max(seror)) %>%
  group_by(pathogen,childid,time) %>%
  slice(1) %>%
  filter(time=="A") %>%
  mutate(ptr = ifelse(seropos==1,agediff,0),
         ptr = ifelse(seropos==1 & seror==1,agediff/2,ptr)) %>%
  ungroup() %>%
  select(pathogen,childid,seror,ptr)

#--------------------------------
# add-in the sero-reversion
# indicator and person time vars
#--------------------------------
drcm <- left_join(dfoi,dsr2,by=c("pathogen","childid")) %>%
  mutate(pathogen=droplevels(pathogen))

#--------------------------------
# estimate seroconversion rates
# under a reversible catalytic
# model with assumed 
# seroconversion rate the 
# empirical rate from the study
# (thus inference under-estimates
# the variability since there is
# uncertainty in the seroreversion
# rate)
#--------------------------------
rcmests <- foreach(ab=levels(drcm$pathogen),.combine=rbind) %dopar%  {
    # subset the data to children <=17 months for a particular pathogen
    pd <- filter(drcm, pathogen==ab & age<=17) 
    # estimate empirical sero-reversion rate to use in the model
    dri <- pd %>%
      ungroup() %>%
      select(seror,ptr) %>%
      summarise_all(function(x) sum(x,na.rm=T))
    ri <- dri$seror/dri$ptr
    # collapse data to counts by age in months
    pd <- pd %>%
      select(age,seropos) %>%
      mutate(n=1) %>%
      group_by(age) %>%
      summarize_all(sum) %>%
      select(age,n,seropos)
    
    # maximum likelihood solution
    # with fixed sero-reversion rate
    minll <- optim(c(0.1),fn=Lrcm,r=ri,data=pd,
    hessian=TRUE,method="Brent",lower=0,upper=1)
    lambda_hat <- minll$par
    # estimate SE and CI from the inverse information matrix
    I <- solve(minll$hessian)
    lambda_se <- sqrt(diag(I))
    lambda_lb <- lambda_hat-1.96*lambda_se
    lambda_ub <- lambda_hat+1.96*lambda_se
    di <- data.frame(pathogen=ab,rho=ri,lambda=lambda_hat,lambda_lb,lambda_ub)
    return(di)
}

knitr::kable(rcmests,digits=3,
             caption="Average force of infection estimated from a cross-sectional seroprevalence curve, reversible catalytic model",
             col.names = c("Pathogen","Seroreversion rate","Seroconversion rate per month","min95","max95"),
             row.names = FALSE)
```

Average force of infection estimated from a cross-sectional seroprevalence curve, reversible catalytic model

| Pathogen | Seroreversion rate | Seroconversion rate per month | min95 | max95 |
| --- | --- | --- | --- | --- |
| Giardia | 0.029 | 0.030 | 0.023 | 0.036 |
| Cryptosporidium | 0.112 | 0.083 | 0.068 | 0.098 |
| E. histolytica | 0.087 | 0.009 | 0.005 | 0.012 |
| Salmonella | 0.129 | 0.022 | 0.015 | 0.029 |
| ETEC | 0.000 | 0.249 | 0.214 | 0.283 |
| Campylobacter | 0.001 | 0.306 | 0.259 | 0.354 |

The above estimates do not account for uncertainty in estimates of the seroreversion rate. Below is a slightly modified approach to the estimate of seroconversion rate parameter variance that incorporates uncertainty in the seroreversion rate as well. It uses information in the longitudinal data and a nonparametric bootstrap that resamples children with replacement, and in each replicate estimates the seroreversion rate. With the seroreversion rate estimated in each replicate, it then fits the reversible catalytic model. 95% confidence intervals are then estimated using the 2.5% and 97.5% tiles of the bootstrap distribution.

```
#--------------------------------
# estimate bootstrap CIs
# that resample children with 
# replacement, and within each
# replicate estimate the 
# longitudinal seroreversion 
# rate and then the seroconversion
# rate within a reversible catalytic
# model that uses the seroconversion rate
# from the bootstrap replicate as a fixed
# parameter
#--------------------------------
dboot <- drcm %>%
  group_by(pathogen) %>%
  select(childid,pathogen,age,seropos,seror,ptr) %>%
  mutate(childid=as.character(childid))
set.seed(123)
nreps <- 1000
ids <- unique(dboot$childid)
bsamp <- matrix(sample(ids,size=length(ids)*nreps,replace=TRUE),
                nrow=length(ids),ncol=nreps)
bootests <- foreach(ab=levels(dboot$pathogen),.combine=rbind) %:%
  foreach(brep=1:nreps,.combine=rbind) %dopar% {
    di <- dboot %>% filter(pathogen==ab & age<=17)
    di <- left_join(data.frame(childid=bsamp[,brep]),di,by=c("childid")) 
    dri <- di %>%
      ungroup() %>%
      select(seror,ptr) %>%
      summarise_all(function(x) sum(x,na.rm=T))
    ri <- dri$seror/dri$ptr
    di <- di %>%
      select(age,seropos) %>% 
      mutate(n=1) %>%
      group_by(age) %>%
      summarize_all(function(x) sum(x,na.rm=TRUE)) %>%
      select(age,n,seropos)
    minll <- try(optim(c(0.1),fn=Lrcm,r=ri,data=di,method="Brent",lower=0,upper=1),silent=TRUE)
    if(class(minll)=="try-error") {
      resi <- data.frame(pathogen=ab,lambda=NA,rho=NA)
    } else{
      resi <- data.frame(pathogen=ab,lambda=minll$par,rho=ri)
    }
    
  }

boot_cis <-  bootests %>% 
  group_by(pathogen) %>%
  # restrict to replicates where the model converged
  summarize(lambda_boot_lb = quantile(lambda,probs=c(0.025),na.rm=T),
            lambda_boot_ub = quantile(lambda,probs=c(0.975),na.rm=T),
            rho_boot_lb = quantile(rho,probs=c(0.025),na.rm=T),
            rho_boot_ub = quantile(rho,probs=c(0.975),na.rm=T)
  )

# join the estimates to the bootstrap CIs
rcmests <- rcmests %>%
  left_join(boot_cis,by="pathogen") %>%
  select(pathogen,lambda,lambda_lb,lambda_ub,lambda_boot_lb,lambda_boot_ub,rho,rho_boot_lb,rho_boot_ub)

knitr::kable(rcmests,digits=3,
             caption="Average force of infection estimated from a cross-sectional seroprevalence curve, reversible catalytic model",
             col.names = c("Pathogen","Seroconversion rate per month","min95","max95","boot_min95","boot_max95","Seroreversion rate per month","min95","max95"),
             row.names = FALSE)
```

Average force of infection estimated from a cross-sectional seroprevalence curve, reversible catalytic model

| Pathogen | Seroconversion rate per month | min95 | max95 | boot\_min95 | boot\_max95 | Seroreversion rate per month | min95 | max95 |
| --- | --- | --- | --- | --- | --- | --- | --- | --- |
| Giardia | 0.030 | 0.023 | 0.036 | 0.022 | 0.039 | 0.029 | 0.000 | 0.069 |
| Cryptosporidium | 0.083 | 0.068 | 0.098 | 0.062 | 0.114 | 0.112 | 0.062 | 0.180 |
| E. histolytica | 0.009 | 0.005 | 0.012 | 0.004 | 0.015 | 0.087 | 0.000 | 0.226 |
| Salmonella | 0.022 | 0.015 | 0.029 | 0.013 | 0.033 | 0.129 | 0.054 | 0.230 |
| ETEC | 0.249 | 0.214 | 0.283 | 0.217 | 0.287 | 0.000 | 0.000 | 0.000 |
| Campylobacter | 0.306 | 0.259 | 0.354 | 0.259 | 0.363 | 0.001 | 0.000 | 0.003 |

```
# add-back the antigen labels to later merge with the other estimates
rcmests <- rcmests %>%
  mutate(antigenf = case_when(
    pathogen == "Giardia" ~ "Giardia VSP-3 or VSP-5",
    pathogen == "Cryptosporidium" ~ "Cryptosporidium Cp17 or Cp23",
    pathogen == "E. histolytica" ~ "E. histolytica LecA",
    pathogen == "Salmonella" ~ "Salmonella LPS groups B or D",
    pathogen == "ETEC" ~ "ETEC LT B subunit",
    pathogen == "Campylobacter" ~ "Campylobacter p18 or p39")
  ) %>%
  mutate(antigenf=factor(antigenf,levels=levels(dfoi$antigenf)))
```

Including sampling variability in the seroreversion rate widened the 95% confidence intervals on the seroconversion rate slightly, but not substantially. For ETEC and Campylobacter, which had no evidence for seroreversion during the study, asymptotic 95% CIs based on the information matrix and the nonparametric bootstrap were almost identical (figure below).

```
rcmp <- ggplot(data=rcmests,aes(x=antigenf,y=lambda*12))+
  geom_pointrange(aes(ymin=lambda_lb*12,ymax=lambda_ub*12),position=position_nudge(x=-0.1),color=cblue)+
  geom_pointrange(aes(ymin=lambda_boot_lb*12,ymax=lambda_boot_ub*12),position=position_nudge(x=0.1),color=corange)+
  annotate("text",x=6.3,y=3,label="boostrap CIs with variable seroreversion",color=corange)+
  annotate("text",x=5.7,y=3,label="asymptotic CIs with fixed seroreversion",color=cblue)+
  labs(y="seroconversion rate per year")+
  coord_flip()+
  theme_minimal()

rcmp
```

Below are the estimates converted to average rates per year.

```
# convert from rates per month to rates per year
rcmests_yr <- rcmests %>%
  mutate(lambda=lambda*12,lambda_lb=lambda_lb*12,lambda_ub=lambda_ub*12,lambda_boot_lb=lambda_boot_lb*12,lambda_boot_ub=lambda_boot_ub*12,rho=rho*12,rho_boot_lb=rho_boot_lb*12,rho_boot_ub=rho_boot_ub*12) %>%
  select(pathogen,lambda,lambda_lb,lambda_ub,lambda_boot_lb,lambda_boot_ub,rho,rho_boot_lb,rho_boot_ub)

knitr::kable(select(rcmests_yr,-lambda_lb,-lambda_ub),digits=1,
             caption="Average force of infection estimated from a cross-sectional seroprevalence curve, reversible catalytic model",
             col.names = c("Pathogen","Seroconversion rate per year","min95","max95","Seroreversion rate per year","min95","max95"),
             row.names = FALSE)
```

Average force of infection estimated from a cross-sectional seroprevalence curve, reversible catalytic model

| Pathogen | Seroconversion rate per year | min95 | max95 | Seroreversion rate per year | min95 | max95 |
| --- | --- | --- | --- | --- | --- | --- |
| Giardia | 0.4 | 0.3 | 0.5 | 0.4 | 0.0 | 0.8 |
| Cryptosporidium | 1.0 | 0.7 | 1.4 | 1.3 | 0.7 | 2.2 |
| E. histolytica | 0.1 | 0.1 | 0.2 | 1.0 | 0.0 | 2.7 |
| Salmonella | 0.3 | 0.2 | 0.4 | 1.5 | 0.6 | 2.8 |
| ETEC | 3.0 | 2.6 | 3.4 | 0.0 | 0.0 | 0.0 |
| Campylobacter | 3.7 | 3.1 | 4.4 | 0.0 | 0.0 | 0.0 |

# Semiparametric estimates of force of infection

## Spline estimation of FOI with approximate simultaneous CIs

Cubic splines are one, flexible approach to model age-dependent seroprevalence, which we have used in the main text. An advantage of using cubic splines in a GAM model to estimate the age-dependent seroprevalence curve is that under the model we can obtain approximate point-wise inference for the curve and functions of the curve (including its derivative). Pointwise intervals for fully nonparametric fits of the curve, such as those obtained with ensemble machine learning, is much more difficult. Below we derive approximate pointwise and simultaneous confidence intervals for the force of infection under a GAM with data-adaptive cubic splines fit to age. We use finite differences to estimate the derivative of the seroprevalence curve \(F'(a)\), and a parametric bootstrap of the posterior estimates of smoothing coefficients to obtain inference.

Below is a function to estimate the age-specific FOI under a GAM model with cubic splines for age. It also estimates approximate point-wise and simultaneous confidence intervals for the FOI. We have derived this estimator from methods inspired by Prof. Gavin Simpson’s approach to estimate simultaneous confidence intervals for the derivatives of smooths (https://www.fromthebottomoftheheap.net/2017/03/21/simultaneous-intervals-for-derivatives-of-smooths/).

The basic insight – originally articulated by *Shkedy 2003* and published with more elaboration in *Hens et al. 2012* – is that within generalized linear models, it is possible to express the force of infection (hazard of seroconversion) as the derivative of the linear predictor multiplied by the predicted response. The relationships for a variety of link functions are summarized in Table 5.1 of Hens et al. 2012 ( *Modeling Infectious Disease Parameters Based on Serological and Social Contact Data*. Springer), and can be derived using basic calculus. Here, we focus on the logit transformation.

Let \(\eta[ P(Y=1|A)] = \textrm{logit}~P(Y=1|A) = g(A)\) for seropositivity indicator \(Y\) and age \(A\), where \(g(A)\) is an arbitrary function (here, estimated semiparametrically with cubic splines). The age specific prevalence predicted from the model is: \(\hat E(Y|A=a) = \frac{\exp[\hat\eta(a)]}{1+\exp[\hat\eta(a)]}\) and the age-specific force of infection is: \(\lambda(a) = \eta'(a)\frac{\exp[\hat\eta(a)]}{1+\exp[\hat\eta(a)]}\), where \(\eta'(a)\) is the first derivative of the linear predictor from the logit model. Below, we estimate \(\eta'(a)\) using finite differences, which requires predicting prevalence over a fine grid of ages and then differncing the predictions to estimate the derivative. Although this study rounded age in months, the prediction grid needs to be at high resolution (much finer than months) to accurately estimate the derivative using finite differences. Below, we predict over a grid of ages in 0.01 months.

```
#----------------------------
# function to estimate the
# age-specific FOI under a
# GAM model smooth
#----------------------------
foiCI <- function(m,newdata,eps=1e-07,level=0.95,nreps=10000) {
  
  # variance-covariance matrix from the spline fit
  # unconditional on the fit of the mean
  # this is slightly more conservative and with better coverage
  # properties. 
  # See Marra G, Wood SN. 
  # Coverage Properties of Confidence Intervals 
  # for Generalized Additive Model Components.   
  # Scand Stat Theory Appl. 2012;39: 53–74.
  Vb <- vcov(m,unconditional = TRUE)
  
  # calculate the linear predictor, g(Ft), and its pointwise SE
  pred <- predict(m, newdata, se.fit = TRUE)
  Ft <- pred$fit
  Ft.se <- pred$se.fit
  
  # simulate from the posterior distrivbution
  # with mean zero and variance from the posterior
  # assuming multivariate normal coefficients
  BUdiff <- MASS::mvrnorm(n = nreps, mu = rep(0, nrow(Vb)), Sigma = Vb)
  
  # get a critical value for the
  # simultaneous confidence interval of g(Ft)
  X0 <- predict(m, newdata, type = "lpmatrix")
  Ft.simDev <- X0 %*% t(BUdiff)
  Ft.absDev <- abs(sweep(Ft.simDev, 1, Ft.se, FUN = "/"))
  Ft.masd <- apply(Ft.absDev, 2L, max)
  Ft.crit <- quantile(Ft.masd, prob = 0.95, type = 8)
  
  # calculate the derivative, g'(Ft), using
  # finite differences and its pointwise SE
  # ?mgcv::predict.gam includes an example of this
  newdata2 <- newdata+eps
  X1 <- predict(m,newdata2,type='lpmatrix')
  Xp <- (X1 - X0) / eps
  ft <- Xp %*% coef(m)
  ft.se <- rowSums(Xp %*% Vb * Xp)^0.5
  
  # get a critical value for the
  # simultaneous confidence interval of the derivative
  ft.simDev <- Xp %*% t(BUdiff)
  ft.absDev <- abs(sweep(ft.simDev, 1, ft.se, FUN = "/"))
  ft.masd <- apply(ft.absDev, 2L, max)
  ft.crit <- quantile(ft.masd, prob = level, type = 8)
  
  # parametric bootstrap simulation from the
  # model coefficient estimates, assuming the model is true
  # FOI is the derivative of the linear predictor
  # multiplied by the predicted probability, Ft
  sims<- MASS::mvrnorm(n = nreps, mu = coef(m), Sigma = Vb)
  Ft.bs <- X0 %*% t(sims)
  ft.bs <- Xp %*% t(sims)
  foi.bs <- ft.bs*(exp(Ft.bs)/(1+exp(Ft.bs)))
  
  # estimate age-specific force of infection
  # averaged over the bootstrap replicates
  # along with SE and confidence intervals for FOI
  # use whichever critical value is larger for Ft or ft
  # for the simultaneous CIs
  foi <- rowMeans(foi.bs)
  foi.crit <- max(Ft.crit,ft.crit)
  foi.se <- apply(foi.bs,1,function(x) sd(x))
  foi.ci <- t(apply(foi.bs,1,function(x) quantile(x,probs=c(0.025,0.975))) )
  foi.Sci <- cbind(foi - foi.crit*foi.se, foi + foi.crit*foi.se)
  
  # return objects
  list(foi=foi,foi.se=foi.se,foi.ci=foi.ci,foi.ciS=foi.Sci,Ft=Ft,Ft.se=Ft.se,Ft.crit=Ft.crit,ft=ft,ft.se=ft.se,ft.crit=ft.crit,model=m,data=newdata)
  
}
```

Ensure that the FOI estimation procedure is doing what it should be – namely that the simultaneous CIs have correct coverage. Use *Campylobacter* as an example.

```
#----------------------------
# subset the data to 
# campylobacter to test
#----------------------------
dd <- d %>%
  filter(antigen=="p18p39")

# fit a GAM model
m <- gam(seropos~s(age,bs="cr"),data=dd,family="binomial")

# Model predictions of FOI, with age grid newd
set.seed(1)
newd <- data.frame(age=seq(4,17,by=0.01))
foi_ests <- foiCI(m=m,newdata=newd)
foipd <- data.frame(age=foi_ests$data,foi=foi_ests$foi,foi_lb=foi_ests$foi.ciS[,1],foi_ub=foi_ests$foi.ciS[,2])

foiplot <- ggplot(data=foipd,aes(x=age,y=foi,ymin=foi_lb,ymax=foi_ub)) +
  geom_ribbon(fill=cgrey,color=NA,alpha=0.5) +
  geom_line() +
  coord_cartesian(ylim=c(0,1))+
  theme_minimal()
foiplot
```

Simulate 1000 draws from the posterior distribution to ensure there is correct coverage

```
#----------------------------
# simulate 1000 estimates of the prevalence
# and its derivatives from the posterior
# distribution 
#----------------------------
eps <- 1e-07
Vb <- vcov(m, unconditional = TRUE)
set.seed(1234)
sims <- MASS::mvrnorm(1000, mu = coef(m), Sigma = Vb)
X0 <- predict(m, newd, type = "lpmatrix")
newd <- newd + eps
X1 <- predict(m, newd, type = "lpmatrix")
Xp <- (X1 - X0) / eps
gprevs  <- X0 %*% t(sims)
derivs <- Xp %*% t(sims)

#----------------------------
# get the simulated 
# FOI estimates from the 
# linear predictor + its derivative
#----------------------------
fois <- derivs*(exp(gprevs)/(1+exp(gprevs)))
```

Plot a random sample of bootstrap replications from the posterior distribution, and overlay the mean plus simultaneous CI for visual inspection

```
set.seed(123)
matplot(foipd$age,fois[, sample(1000, 50)], type = "l", lty = "solid",col="grey80")
lines(foipd$age,foipd$foi,type="l",lwd=2)
lines(foipd$age,foipd$foi_lb,lty=2)
lines(foipd$age,foipd$foi_ub,lty=2)
```

Calculate the coverage probability: this is the proportion of smooths that fall *entirely* within the simultaneous confidence interval (should be close to 95% if have correct coverage).

```
inCI <- function(x, upr, lwr) {
    all(x >= lwr & x <= upr)
}
fitsInCI <- with(foipd, apply(fois, 2L, inCI, upr = foi_ub, lwr = foi_lb))
sum(fitsInCI)/length(fitsInCI)
```

```
## [1] 0.941
```

Compare the estimates using the finite differences on the GAM fit with a separate R function from Hens et al. 2012 to ensure the GAM approach derived above is consistent. Below is a function for the semiparametric estimation of the force of infection from any age-dependent seroprevalence curve, from Hens et al 2012, Chapter 8 (Hens N, Shkedy Z, Aerts M, Damme CFPV, Beutels P. *Modeling Infectious Disease Parameters Based on Serological and Social Contact Data*. Springer; 2012.).

```
#--------------------------------
# non parametric FOI estimation
# using age and predicted probabilities
#--------------------------------
# original function from Hens 2012 ch8 drops both the first and last 
# age category. But, see no reason to drop the first age category and
# so have modified this calculation to just drop the last obs
# this is the substituted line:
# foi   <- approx((grid[-1]+grid[-length(grid)])/2, dp, grid[c(-1,-length(grid))])$y / (1-pgrid[c(-1,-length(grid))])
#  and in the return object grid=grid[c(-1,-length(grid))]

foinp <-function(x,p) {
  grid  <- sort(unique(x))
  pgrid <- (p[order(x)])[duplicated(sort(x))==F]
  psus  <- 1-pgrid
  dp    <- diff(pgrid)/diff(grid)
  midpt <- (grid[-1]+grid[-length(grid)])/2
  foi <- approx(midpt,dp,midpt)$y/psus[-c(length(psus))]
  return(list(grid=grid[c(-length(grid))],foi=foi))

}
```

Now compare the GAM finite difference FOI with that estimated using the Hens et al. 2012 function (an internal consistency check). There is excellent agreement.

```
# GAM model fit and predictions over the FOI age grid (newd)
campd <- data.frame(newd,fit=predict(m,newdata=newd,type="response"))
foicamp <- foinp(x=campd$age,p=campd$fit)
foicamp <- data.frame(age=foicamp$grid,foi=foicamp$foi)

foicompp <- ggplot(data=foicamp,aes(x=age,y=foi)) +
  geom_line(color=cteal,lwd=1.5,alpha=0.8) +
  geom_line(data=foipd,aes(x=age,y=foi),color=corange,lwd=1.5,alpha=0.8) +
  coord_cartesian(ylim=c(0,0.5))+
  theme_minimal()
foicompp
```

## Estimate FOI for each pathogen

Estimate semiparamteric age-specific seroconversion rate (force of infection) using splines

```
# FOI from spline fits
newd <- data.frame(age=seq(4,17,by=0.01))
gamfoi <- foreach(ab=levels(dfoi$antigenf),.combine=rbind) %dopar% {
    pd <- filter(dfoi, antigenf==ab) 
    mi <- gam(seropos~s(age,bs="cr"),data=pd,family="binomial")
    foii <- foiCI(mi,newd)
    di <- data.frame(antigenf=ab,
                       age=newd,
                       foi=foii$foi,
                       foi_lwrS=foii$foi.ciS[,1],foi_uprS=foii$foi.ciS[,2],
                       foi_lwrP=foii$foi.ci[,1],foi_uprP=foii$foi.ci[,2])
    return(di)
}
```

## Figures of results

Faceted figure that includes 95% CIs

```
# bright color-blind palette defined in an earlier code chunk
# pcols <- c(cred,corange,cgreen,cteal,cblue,cmagent,cgrey)
# pcols <- c(corange,cred,cmagent,cblue,cteal,cgreen,cgrey)
vircols <- viridis(n=7,alpha=1,begin=0.2,end=0.97)
pcols <- cbPalette[c(1:4,6,8)]


pfoi <- ggplot(data=gamfoi,aes(x=age,fill=antigenf,color=antigenf)) +
  # foi estimate from splines with simultaneous 95% CI
  geom_ribbon(aes(ymin=foi_lwrS,ymax=foi_uprS),color=NA,alpha=0.2)+
  geom_line(aes(y=foi)) +
  # horizontal line at 0
  geom_hline(aes(yintercept=0)) +
  # plot aesthetics
  facet_wrap(~antigenf,ncol=2,nrow=6)+
  scale_x_continuous(breaks=seq(4,16,by=2))+
  # scale_y_continuous(breaks=seq(0,1,by=0.2),labels=sprintf("%1.0f",seq(0,1,by=0.2)*100))+
  coord_cartesian(ylim=c(0,1),xlim=c(4,17))+
  labs(x="Age in months",y="Age-specific force of infection (rate per child-month)") +
  scale_fill_manual(values=pcols) +
  scale_color_manual(values=pcols) +
  theme_minimal() +
  theme(legend.position = "none")

pfoi
```

Compact figure with all pathogens on the same panel

```
# bright color-blind palette defined in an earlier code chunk
# pcols <- c(cred,corange,cgreen,cteal,cblue,cmagent,cgrey)
# pcols <- c(corange,cred,cmagent,cblue,cteal,cgreen,cgrey)
pcols <- cbPalette[c(1:4,6,8)]


plabs <- gamfoi %>%
  filter(age==17) %>%
  mutate(foi=ifelse(antigenf=="E. histolytica LecA",foi-0.002,foi),
         foi=ifelse(antigenf=="Salmonella LPS groups B or D",foi+0.002,foi))

pfoi2 <- ggplot(data=gamfoi,aes(x=age,group=antigenf,color=antigenf,fill=antigenf)) +
  # foi estiamte from splines
  geom_line(aes(y=foi)) +
  # horizontal line at 0
  geom_hline(aes(yintercept=0)) +
  # plot aesthetics
  scale_x_continuous(breaks=seq(4,16,by=2))+
  # scale_y_continuous(breaks=seq(0,1,by=0.2),labels=sprintf("%1.0f",seq(0,1,by=0.2)*100))+
  coord_cartesian(ylim=c(0,0.5),xlim=c(4,17))+
  labs(x="Age in months",y="Force of infection\n(seroconversion rate per child-month)") +
  scale_fill_manual(values=pcols) +
  scale_color_manual(values=pcols,guide=guide_legend(title="Pathogen")) +
  theme_minimal(base_size = 14) +
  theme(legend.position = "right"
        )

pfoi2
```

## Semiparametric average FOI, weighted by P(A=a)

Semiparametric estimates above provide age-specific rates. To compare the estimator with constant rate models, we can average over the empirical distribution of age in the study population. This average is also most directly comparable to the average seroconversion rates estimated prospectively in the cohort. \(\hat{\lambda} = \int\_a \lambda(a) P(A=a)\)

```
#----------------------------
# function to estimate the
# average FOI over empirical
# age distribution
# the predictions are based
# on a spline fit and its
# derivative
# the gridA is the empirical
# age distribution
#----------------------------
pAfoi <- function(m,gridA,pA,eps=1e-07,level=0.95,nreps=10000) {
  
  # variance-covariance matrix from the spline fit
  # unconditional on the fit of the mean
  # this is slightly more conservative and with better coverage
  # properties. 
  # See Marra G, Wood SN. 
  # Coverage Properties of Confidence Intervals 
  # for Generalized Additive Model Components.   
  # Scand Stat Theory Appl. 2012;39: 53–74.
  Vb <- vcov(m,unconditional = TRUE)
  
  # calculate the linear predictor, g(Ft), and its pointwise SE
  pred <- predict(m, gridA, se.fit = TRUE)
  Ft <- pred$fit
  Ft.se <- pred$se.fit

  # calculate the derivative, g'(Ft), using
  # finite differences and its pointwise SE
  # ?mgcv::predict.gam includes an example of this
  X0 <- predict(m, gridA, type = "lpmatrix")
  gridA2 <- gridA+eps
  X1 <- predict(m,gridA2,type='lpmatrix')
  Xp <- (X1 - X0) / eps
  ft <- Xp %*% coef(m)
  ft.se <- rowSums(Xp %*% Vb * Xp)^0.5
  
  # parametric bootstrap simulation from the
  # model coefficient estimates, assuming the model is true
  # FOI is the derivative of the linear predictor
  # multiplied by the predicted probability, Ft
  sims<- MASS::mvrnorm(n = nreps, mu = coef(m), Sigma = Vb)
  Ft.bs <- X0 %*% t(sims)
  ft.bs <- Xp %*% t(sims)
  foi.bs <- ft.bs*(exp(Ft.bs)/(1+exp(Ft.bs)))
  
  # merge the parametric bootstrap draws to the empirical 
  # distribution
  foi.bs <- data.frame(age=gridA$age,foi.bs)
  pA_foi.bs <- left_join(data.frame(age=pA),foi.bs,by="age") %>%
    select(-age)
  
  # Average over age for each bootstrap replication
  foi_bs <- colMeans(pA_foi.bs,na.rm=T)
  
  # estimate mean and 
  # percentile based 95% CIs
  foi <- mean(foi_bs)
  foi.se <- sd(foi_bs)
  foi.ci <- quantile(foi_bs,probs=c(0.025,0.975))  

  # return objects
  list(foi=foi,foi.se=foi.se,foi.ci=foi.ci,model=m,gridA=gridA,pA=pA)
  
}


# FOI from spline fits
agegrid <- data.frame(age=seq(4,17,by=0.01))
popavgfoi <- foreach(ab=levels(dfoi$antigenf),.combine=rbind) %dopar% {
    pd <- filter(dfoi, antigenf==ab) 
    mi <- gam(seropos~s(age,bs="cr"),data=pd,family="binomial")
    foii <- pAfoi(mi,gridA=agegrid,pA=pd$age)
    di <- data.frame(antigenf=ab,
                     foi=foii$foi,
                     foi_se=foii$foi.se,
                     foi_lb=foii$foi.ci[1],
                     foi_ub=foii$foi.ci[2])
    return(di)
}

knitr::kable(select(popavgfoi,-foi_se),digits=3,
             caption="Population-average force of infection estimated from a cross-sectional seroprevalence curve",
             col.names = c("Pathogen","Incidence per month","min95","max95"),
             row.names = FALSE)
```

Population-average force of infection estimated from a cross-sectional seroprevalence curve

| Pathogen | Incidence per month | min95 | max95 |
| --- | --- | --- | --- |
| Giardia VSP-3 or VSP-5 | 0.055 | 0.030 | 0.086 |
| Cryptosporidium Cp17 or Cp23 | 0.080 | 0.048 | 0.117 |
| E. histolytica LecA | 0.007 | 0.000 | 0.015 |
| Salmonella LPS groups B or D | 0.011 | 0.002 | 0.023 |
| ETEC LT B subunit | 0.223 | 0.083 | 0.370 |
| Campylobacter p18 or p39 | 0.311 | 0.119 | 0.506 |

## Semiparametric average FOI, integrating over age

Another way to estimate the average force of infection from the seroprevalence curve is to use the basic relationship between the hazard (force of infection) and the cumulative hazard. This ignores the distribution of age in the study population – in this particular study, the age distriution is fairly uniform so this estimator should be very similar to the previous estimator that averages over the empirical age distribution.

The hazard or force of infection at age \(a\) is: \(\lambda(a) = F'(a)/(1-F(a))\). From the basic definition of the hazard rate, we have:

\(1- F(a) = \exp [ - \int\_0^a \lambda(a) da ]\) and \(\int\_0^a \lambda(a) da = - \log [1-F(a)]\).

This is the cumulative hazard. The average hazard per month or year (units of \(a\)) also divided \(-\log[1-F(a)]\) by \(a\). So, all of the information is embedded in the cumulative distribution function, \(F(a)\), which here is the age-dependent seroprevalence curve.

The average hazard over the age period \(a\_1=4\) to \(a\_2=17\) months is:

\[\begin{equation}
\int\_{a\_1}^{a\_2} \lambda(a) da = \frac{\log[1-F(a\_1)]-\log[1-F(a\_2)]}{a\_2 - a\_1}
\end{equation}\]

We estimate its variance from a parametric bootstrap simulation of the posterior distributions of the spline parameters (above).

```
avgFOI <- function(m,newdata,a1,a2,nreps=10000) {
  require(mgcv)
  require(dplyr)
  
  # limit the prediction data to the two time points
  newdata <- newdata %>%
    filter(age %in% c(a1,a2)) %>%
    arrange(age)
  
  # get predicted seroprevalence, Ft, from GAM model
  gFt <- predict(m, newdata)
  Ft <- exp(gFt)/(1+exp(gFt))
  
  # average FOI over a1 to a2 is: 
  # foi = [log(1-F(a1))-log(1-F(a2))] / (a2-a1)
  mufoi <- (log(1-Ft[1]) - log(1-Ft[2])) / (a2-a1) 

  # parametric bootstrap simulation from the
  # model coefficient estimates, assuming the model is true
  X0 <- predict(m, newdata, type = "lpmatrix")
  Vb <- vcov(m,unconditional = TRUE)
  sims<- MASS::mvrnorm(n = nreps, mu = coef(m), Sigma = Vb)
  gFt.bs <- X0 %*% t(sims)
  Ft.bs <- exp(gFt.bs)/(1+exp(gFt.bs))
  mufoi.bs <- (log(1-Ft.bs[1,]) - log(1-Ft.bs[2,])) / (a2-a1)
  
  # estimate approximate bs SE and percentile-based 95% credible interval
  mufoi.se <- sd(mufoi.bs)
  mufoi.ci <- quantile(mufoi.bs,probs=c(0.025,0.975))
  res <- data.frame(mufoi,mufoi_se=mufoi.se,mufoi_lb=mufoi.ci[1],mufoi_ub=mufoi.ci[2])
  return(res)
}


# average FOI from a GAM model
newd <- data.frame(age=c(4,17))
avgfoi <- foreach(ab=levels(dfoi$antigenf),.combine=rbind) %dopar% {
    pd <- filter(dfoi, antigenf==ab) 
    mi <- gam(seropos~s(age,bs="cr"),data=pd,family="binomial")
    foii <- avgFOI(mi,newd,a1=4,a2=17)
    di <- data.frame(antigenf=ab,foii)
    return(di)
}

knitr::kable(select(avgfoi,-mufoi_se),digits=3,
             caption="Average force of infection estimated from a cross-sectional seroprevalence curve",
             col.names = c("Pathogen","Incidence per month","min95","max95"),
             row.names = FALSE)
```

Average force of infection estimated from a cross-sectional seroprevalence curve

| Pathogen | Incidence per month | min95 | max95 |
| --- | --- | --- | --- |
| Giardia VSP-3 or VSP-5 | 0.052 | 0.032 | 0.078 |
| Cryptosporidium Cp17 or Cp23 | 0.076 | 0.049 | 0.107 |
| E. histolytica LecA | 0.006 | 0.000 | 0.015 |
| Salmonella LPS groups B or D | 0.011 | 0.002 | 0.022 |
| ETEC LT B subunit | 0.236 | 0.111 | 0.371 |
| Campylobacter p18 or p39 | 0.315 | 0.129 | 0.494 |

Indeed the estimates are very similar with those above, averaged over the empirical distribution of age. This is because measurements were almost uniformly distributed over the age range in this cohort. Given this similarity, the population-averaged estimates will be used below in the comparison across methods.

# Comparison of estimates

Retrieve the results from the longitudinal analysis of the data and compare estimates with those estimated above from the seroprevalence curves, which assume the data are cross-sectional.

```
#--------------------------------
# load the prospective estimates
# these are incidence per year
# created with script:
# Fig6-asembo-incidence-analysis.Rmd
#
# Exclude cholera due to cross-reactivity
# with ETEC and thus difficulty of 
# interpretation
#--------------------------------
est_long <- readRDS("~/enterics-seroepi/output/asembo-enteric-ab-ests-incidencerates.rds") %>%
  filter(antigen!="cholera") %>%
  mutate(method="Longitudinal") %>% 
  mutate(outcome = case_when(
    outcome=="seroconversion rate (force of infection)" ~ "seroconversion (force of infection)",
    outcome=="seroreversion rate" ~ "seroreversion")) %>%
  select(method,outcome,antigenf,starts_with("rate"))

# rename columns and stack the data for plotting
# re-scale monthly rates to per year
est_xc <- popavgfoi %>%
  mutate(method="Cross sectional, spline",foi=foi*12,foi_lb=foi_lb*12,foi_ub=foi_ub*12) %>%
  select(method,antigenf,rate=foi,rate_lb=foi_lb,rate_ub=foi_ub) %>%
  mutate(outcome="seroconversion (force of infection)")

# estimates from constant rate (exponential) model
est_cr <- glmfoi %>%
  mutate(method="Cross sectional, exponential",
         lambda=lambda*12,lambda_lb=lambda_lb*12,lambda_ub=lambda_ub*12) %>%
  select(method,antigenf,rate=lambda,rate_lb=lambda_lb,rate_ub=lambda_ub) %>%
  mutate(outcome="seroconversion (force of infection)")

# estimates from RC model, use bootstrap estimates of 95% CIs
est_rcm <- rcmests %>%
  mutate(method="Cross sectional, RCM",
         lambda=lambda*12,lambda_lb=lambda_boot_lb*12,lambda_ub=lambda_boot_ub*12) %>%
  select(method,antigenf,rate=lambda,rate_lb=lambda_lb,rate_ub=lambda_ub) %>%
  mutate(outcome="seroconversion (force of infection)")


#--------------------------------
# append the longitudinal and
# cross sectional estimates
#--------------------------------
rate_comp <- bind_rows(est_long,est_xc,est_cr,est_rcm) %>%
  mutate(antigenf=factor(antigenf,levels=levels(est_long$antigenf)),
         method=factor(method,levels=c("Longitudinal","Cross sectional, spline","Cross sectional, RCM","Cross sectional, exponential"))) %>%
  arrange(antigenf,outcome,method)
```

# Figure 6, FOI Comparison

**Figure caption**: Enteropathogen seroconversion and seroreversion rates among 205 children ages 4 to 17 months measured longitudinally in Asembo, Kenya, 2013. The seroconversion rate is a measure of a pathogen’s force of infection. Longitudinal estimates are non-parametric rates of incident seroconversions and seroreversions among children at risk, assumed to oc- cur at the midpoint of the measurement interval. Cross-sectional estimators were derived from age-specific seroprevalence curves using semiparametric cubic splines (spline), a parametric constant rate survival model (exponential), and a reversible catalytic model (RCM) that as- sumed constant seroconversion and seroreversion rates, with the seroreversion rate estimated from prospective data. Lines mark 95% confidence intervals. IgG response measured in mul- tiplex using median fluorescence units minus background (MFI-bg) on the Luminex platform (N=410 measurements from 205 children).

```
# sort pathogens by rates
ratelabs <- c("E. histolytica LecA",
              "Salmonella LPS groups B or D",
              "Giardia VSP-3 or VSP-5",
              "Cryptosporidium Cp17 or Cp23",
              "ETEC LT B subunit",
              "Campylobacter p18 or p39")
rate_comp2 <- rate_comp %>%
  mutate(antigenf=factor(antigenf,levels=ratelabs),
         method=factor(method,levels=rev(levels(method)))) %>%
  arrange(antigenf,outcome,method)


pcols <- c(cbPalette[c(2,3,7)],"black")
ratecomp_plot <- ggplot(data=rate_comp2,
                        aes(x=antigenf,y=rate,color=method,fill=method)) +
  geom_errorbar(aes(ymin=rate_lb,ymax=rate_ub),
                  width=0.15,
                  position=position_nudge(x=rep(c(-0.3,-0.1,0.1,0.3,0),6)))+
  geom_point(size=2.5,pch=21,color="black",
             position=position_nudge(x=rep(c(-0.3,-0.1,0.1,0.3,0),6)))+
  facet_grid(~outcome)+
  labs(x="",y="rate per child-year at risk") +
  scale_y_continuous(breaks=0:7)+
  scale_fill_manual(values=pcols) +
  scale_color_manual(values=pcols,guide=FALSE) +
  # scale_shape_manual(values=c(21,22,23,24),guide_legend(title="Estimation method"))+
  guides(fill=guide_legend(title="method", 
                            reverse=TRUE,
                            keywidth=0.15,
                            keyheight=0.15,
                            default.unit="inch",
                            ncol=1))+
  coord_flip(ylim=c(0,7.5))+
  theme_minimal(base_size = 16) +
  theme(
        legend.position = c(0.35,0.17),
        legend.background=element_rect(color="white"),
        legend.title=element_text(size=12,vjust=1),
        legend.margin=margin(0,0,0,0),
        legend.box.margin=margin(b=-10,t=10,l=-10,r=-10),
        legend.box.just="left",
        # legend.title=element_text(size=12),
        legend.text=element_text(size=8),
        axis.title.x = element_text(size=12)
        )

ratecomp_plot
```

```
# save PDF and TIFF versions
ggsave(here::here("figs","Fig6-asembo-ab-foi-comparison.pdf"),plot=ratecomp_plot,device=cairo_pdf,width=10,height=4.5)
ggsave(here::here("figs","Fig6-asembo-ab-foi-comparison.TIFF"),plot=ratecomp_plot,device="tiff",width=10,height=4.5)
```

We note that *Cryptosporidium* seroconversion rate estimates of 1.1 per child-year are very close to an estimate of 0.8 per child-year from a Peruvian cohort (Priest et al. 2005 J Clin Micro; https://www.ncbi.nlm.nih.gov/pubmed/16208002).

Below, plot the estimates on separate scales for lower and higher transmission pathogens to enable clearer comparisons.

```
pcols <- c(cbPalette[c(2,3,7)],"black")
rate_comp3 <- rate_comp2 %>%
  filter(outcome=="seroconversion (force of infection)") %>%
  mutate(pathgroup=ifelse(antigenf %in% c("ETEC LT B subunit","Campylobacter p18 or p39"),"Higher transmission","Lower transmission"),
         pathgroup=factor(pathgroup,levels=c("Lower transmission","Higher transmission")))


ratecomp_plot2 <- ggplot(data=rate_comp3,aes(color=method,fill=method)) +
  facet_wrap(~pathgroup,scales="free")+
  geom_pointrange(aes(x=antigenf,y=rate,ymin=rate_lb,ymax=rate_ub),
                  fill="white",size=0.5,
                  position=position_nudge(x=rep(c(-0.3,-0.1,0.1,0.3),3)) )+
  labs(x="",y="force of infection\n(seroconversion rate per child-year)") +
  scale_y_continuous(breaks=0:7)+
  scale_x_discrete(drop=TRUE,aesthetics=c("color"))+
  # scale_fill_manual(values=pcols) +
  scale_color_manual(values=pcols) +
    guides(color=guide_legend(title="force of infection method", 
                            reverse=TRUE,
                            keywidth=0.2,
                            keyheight=0.15,
                            default.unit="inch",
                            ncol=1))+
  # coord_cartesian(ylim=c(0,7.5))+
  coord_flip()+
  theme_minimal() +
  theme(legend.position = "top",
        strip.text = element_text(size=12))
```

```
## Scale for 'colour' is already present. Adding another scale for
## 'colour', which will replace the existing scale.
```

```
ratecomp_plot2
```

For most pathogens, the semiparametric estimate of force of infection using splines was close to the parameter estimated prospectively. In all cases, uncertainty in spline-based estimates of the force of infection derived from seroprevalence was much higher than the prospective estimates, as reflected by wider 95% credible intervals. As Jewell and Emerson (2013) demonstrate under a parametric exponential survival model, the asymptotic relative efficiency of a cross-sectional, current-status analysis compared to a longitudinal analysis is 96.1% if the cumulative proportion of failures is 0.5, but only 65.5% if the proportion of failures is 0.9. These results are broadly consistent with that analysis, with the loss in efficiency much higher among ETEC and *Campylobacter* with seroprevalence that approached 100% by ages 10-12 months in this cohort.

For most pathogens, force of infection estimates from cross-sectional analysis were biased downward compared to the longitudinal analysis – a result that would be expected in the case of seroreversion, which was common in this cohort for all but ETEC and *Campylobacter*. All estimation methods were rank-preserving across pathogens, and many estimates were reasonably close with one-another. Overall, these results show that longitudinal designs should be preferred to estimate enteropathogen force of infection from serological antibody levels, but that modeling approaches based on age-structured seroprevalence reasonably capture relative differences between pathogens in this study.

Jewell, N. P. & Emerson, R. Current Status Data: An Illustration with Data on Avalanche Victims, in *Handbook of Survival Analysis* (eds. Klein, J. P., van Houwelingen, H. C., Ibrahim, J. G. & Scheike, T. H.) 391–412 (Chapman and Hall, 2013).

# Session Info

```
sessionInfo()
```

```
## R version 3.5.3 (2019-03-11)
## Platform: x86_64-apple-darwin15.6.0 (64-bit)
## Running under: macOS High Sierra 10.13.6
## 
## Matrix products: default
## BLAS: /Library/Frameworks/R.framework/Versions/3.5/Resources/lib/libRblas.0.dylib
## LAPACK: /Library/Frameworks/R.framework/Versions/3.5/Resources/lib/libRlapack.dylib
## 
## locale:
## [1] en_US.UTF-8/en_US.UTF-8/en_US.UTF-8/C/en_US.UTF-8/en_US.UTF-8
## 
## attached base packages:
## [1] parallel  grid      stats     graphics  grDevices utils     datasets 
## [8] methods   base     
## 
## other attached packages:
##  [1] doParallel_1.0.11 iterators_1.0.9   foreach_1.4.4    
##  [4] mgcv_1.8-27       nlme_3.1-137      viridis_0.5.1    
##  [7] viridisLite_0.3.0 forcats_0.3.0     stringr_1.4.0    
## [10] dplyr_0.8.0.1     purrr_0.3.2       readr_1.1.1      
## [13] tidyr_0.8.3       tibble_2.1.1      ggplot2_3.1.1    
## [16] tidyverse_1.2.1   here_0.1         
## 
## loaded via a namespace (and not attached):
##  [1] Rcpp_1.0.1       lubridate_1.7.4  lattice_0.20-38  assertthat_0.2.1
##  [5] rprojroot_1.3-2  digest_0.6.18    psych_1.8.4      R6_2.4.0        
##  [9] cellranger_1.1.0 plyr_1.8.4       backports_1.1.4  evaluate_0.13   
## [13] httr_1.4.0       highr_0.8        pillar_1.4.0     rlang_0.3.4     
## [17] lazyeval_0.2.2   readxl_1.1.0     rstudioapi_0.9.0 Matrix_1.2-15   
## [21] rmarkdown_1.12   labeling_0.3     splines_3.5.3    foreign_0.8-71  
## [25] munsell_0.5.0    broom_0.4.4      compiler_3.5.3   modelr_0.1.2    
## [29] xfun_0.6         pkgconfig_2.0.2  mnormt_1.5-5     htmltools_0.3.6 
## [33] tidyselect_0.2.5 gridExtra_2.3    codetools_0.2-16 crayon_1.3.4    
## [37] withr_2.1.2      MASS_7.3-51.1    jsonlite_1.6     gtable_0.3.0    
## [41] magrittr_1.5     scales_1.0.0     cli_1.1.0        stringi_1.4.3   
## [45] reshape2_1.4.3   xml2_1.2.0       tools_3.5.3      glue_1.3.1      
## [49] hms_0.4.2        yaml_2.2.0       colorspace_1.3-2 rvest_0.3.2     
## [53] knitr_1.22       haven_2.1.0
```
