# Supplementary material for: Enteropathogen antibody dynamics and force of infection among children in low-resource settings
Source: eLife. 2019 Aug 19;8:e45594. doi: 10.7554/eLife.45594 (PMC6746552; doi:10.7554/eLife.45594)
Supplement: Supplementary file 8. [file elife-45594-supp8.zip › SIFile8.html]

Enteropathogen antibody dynamics and force of infection among children in low-resource settings


Code 

- Show All Code
- Hide All Code

# Enteropathogen antibody dynamics and force of infection among children in low-resource settings

### Supplementary Information File 8: Sampling interval simulation and sensitivity analyses in Haiti and Kenya

# Notebook Summary

IgG measurements in the Haitian cohort were separated by approximately 1 year, and in the Kenyan cohort by 6 months. We hypothesized that since many enteric pathogens are thought to elicit transiently high IgG responses with rapid waning, tthis could lead us to miss seroconversion events in the interim periods. The empirical estimates in the primary analysis would thus under-estimate the true serological force of infection.

This notebook uses simulation to estimate an approximate upper bound of seroconversion rates that would be possible in the Haiti and Kenya cohorts under conditions of near-continuous boosting and waning. We developed an imputation algorithm using boosting and waning parameters estimated empirically to simulate daily IgG levels for each child in the cohort. That we describe in detail below. We simulated 100 datasets with stochastically generated boosting and waning profiles for each child, and then downsampled the daily resolution at sampling intervals of 30, 60, 180, and 360 days; in Kenya, the cohort was only followed for 6 months, so we did not simulate a 360 day interval.

We then compared the simulation-based estimates of seroconversion rates with similar sampling intervals to the empirical measurements in each cohort to ensure internal consistency, and then estimated rate differences and ratios for each pathogen with more frequent sampling intervals. The results suggest that for most pathogens, increases seroconversion rates with monthly sampling would be < 1 episode per-child year. However, for highest-transmission pathogens ETEC and *Campylobacter*, rates could be 5-8 episodes per year higher if monthly sampling intervals were used.

The entire simulation routine is repeated for the Haiti and Kenya cohorts. Within each cohort, we did not attempt to simulate dependent antibody dynamics, so we included only one antibody per pathogen (e.g., we only included *Cryptosporidium* Cp17, not Cp23). The analysis proceeds in four parts for each cohort: (1) load data, summarize descriptive statistics and antibody-specific estimates of IgG decay and half life; (2) simulate daily IgG trajectories; (3) estimate empirical seroconversion rates for comparison; (4) create figures that summarize results.

# Script preamble

```
#-----------------------------
# preamble
#-----------------------------
library(here)
here::here()
```

```
## [1] "/Users/benarnold/enterics-seroepi"
```

```
# load packages
library(tidyverse)
library(kableExtra)

# set up for parallel computing
library(foreach)
library(doParallel)
registerDoParallel(cores = detectCores() - 1)

# safe color blind palette
# http://jfly.iam.u-tokyo.ac.jp/color/
# http://www.cookbook-r.com/Graphs/Colors_(ggplot2)/
cbPalette <- c("#999999", "#E69F00", "#56B4E9", "#009E73", "#F0E442", "#0072B2", "#D55E00", "#CC79A7")
```

# Haiti

## Load the Haiti data

```
#-----------------------------
# load the formatted data
# created with
# haiti-enteric-ab-data-format.Rmd ->
# Fig1-haiti-ab-distributions.Rmd
#-----------------------------
dl <- readRDS(here::here("data", "haiti_analysis2.rds"))
```

Identify seropositive measurements

```
#-----------------------------
# identify seropositive measures
# hierarchy of information for
# cutoffs:
# 1. ROC
# 2. mixture model based
# 3. estimated among presumed unexposed
#
# store the cutoff value used
# for figures
#
# # restrict to a single antigen 
# per pathogen because the simulations
# do not attempt to model the 
# joint distribution of antibody response
#-----------------------------
ab_in_sim <- c("Giardia VSP-5","Cryptosporidium Cp17","E. histolytica LecA","Salmonella LPS group B","ETEC LT B subunit","Norovirus GI.4","Norovirus GII.4.NO")
dl <- dl %>%
  ungroup() %>%
  filter(antigenf %in% ab_in_sim) %>%
  mutate(antigenf=droplevels(antigenf),
         antigenf=factor(antigenf,levels=ab_in_sim)
  ) %>%
  mutate(
    seropos = ifelse(!is.na(posroc), posroc, posmix),
    serocut = ifelse(!is.na(posroc), roccut, mixcut),
    serocut_desc = ifelse(!is.na(posroc), "ROC", "Mixture Model")
  ) %>%
  mutate(
    seropos = ifelse(!is.na(seropos), seropos, posunex),
    serocut_desc = ifelse(!is.na(serocut), serocut_desc, "Unexp dist"),
    serocut = ifelse(!is.na(serocut), serocut, unexpcut)
  )
```

## Estimates of IgG decay and half life

Identify adjacent measurements where children experienced larger drops in IgG and where the two measurements were separated by less than 1 year.

The rationale for this restriction is to try to identify periods of IgG waning that were unlikely to be influenced by intermediate boosting between measurements. From these data, estimate decay rates for use in the sensitivity analysis, below.

The code below selects, for each antibody, adjacent measurements within a child where IgG MFI declined between measurements, and from those take the largest 20% reductions (bottom quintile). Then, restrict to measurements separated by less than 1 year, and estimate IgG decay rates among this subset of measurements.

Under an exponential decay model where IgG level at time \(t\) is \(N\_t\) and the decay rate parameter is \(\lambda\), \(N\_t = N\_0e^{(-\lambda t)}\), \(\lambda = \frac{-\log(N\_t/N\_0)}{t}\), and the half life is \(t\_{1/2}=\log(2)/\lambda\).

```
# calculate difference in age and restrict data to needed variables
dl2 <- dl %>%
  ungroup() %>%
  group_by(antigenf, id) %>%
  arrange(antigenf, id, age) %>%
  mutate(agediff = lead(age) - age) %>%
  select(pathogen, antigen, antigenf, id, sampleid, age, agediff, seropos, mfi, logmfi, logmfidiff)

# filter to drops in MFI, identify quintiles and select the lowest quintile
# the estimate the decay rate in MFI per day
# filter to observations less than 1 year of one-another to make the assumption
# of no boosting between measurements more plausible.
#
# Rough estimates of half life among this group of measurements
# with large drops assuming an exponential decay
# lambda = -log(N(t)/N(o))*(1/t); t1/2 = log2/lambda
# or
# t_1/2 = t / log_{1/2}(N(t)/N(o))
#
# Note: there are 4 Salmonella LPS group D values that are 0 on the log10
# scale. Drop these observations to avoid problems with log transformation.
d_bigdrop <- dl2 %>%
  ungroup() %>%
  group_by(antigenf) %>%
  filter(logmfidiff < 0) %>%
  filter(logmfi + logmfidiff != 0) %>%
  mutate(qmfidiff = cut(logmfidiff,
    breaks = quantile(logmfidiff, probs = seq(0, 1, 0.2)),
    labels = F
  )) %>%
  filter(qmfidiff == 1 & agediff < 1) %>%
  mutate(mfidiff = 10^logmfidiff * mfi - mfi) %>%
  mutate(
    decayrate = logmfidiff / agediff * (1 / 365.25),
    lambda = -log((mfi + mfidiff) / mfi) / agediff * (1 / 365.25),
    halflife = log(2) / lambda,
    halflife2 = (agediff * 365.25) / log((mfi + mfidiff) / mfi, base = 0.5),
    n=1
  )
```

```
# summarize ages (in years) by antibody
d_bigdrop %>% summarize(n=sum(n), mean = mean(age), med = median(age), min = min(age), max = max(age))
```

```
# summarize MFI drops by antibody
d_bigdrop %>% summarize(n=sum(n), mean = mean(logmfidiff), sd = sd(logmfidiff), min = min(logmfidiff), max = max(logmfidiff))
```

```
# summarize MFI decay rates by antibody
d_bigdrop %>% summarize(n=sum(n), mean = mean(decayrate), sd = sd(decayrate), min = min(decayrate), max = max(decayrate))
```

```
# summarize exponential rate parameter by antibody
d_bigdrop %>% summarize(n=sum(n), mean = mean(lambda), sd = sd(lambda), min = min(lambda), max = max(lambda))
```

```
# summarize MFI half life estimates by antibody
d_bigdrop %>% summarize(n=sum(n), mean = mean(halflife, na.rm = T), sd = sd(halflife, na.rm = T), median = median(halflife, na.rm = T), min = min(halflife, na.rm = T), max = max(halflife, na.rm = T))
```

Box plot of MFI decay

```
pcols <- cbPalette[c(2:4, 6:8)]
pdiff <- ggplot(data = d_bigdrop, aes(y = logmfidiff, x = antigenf, color = pathogen)) +
  geom_jitter() +
  geom_boxplot(color = "black", fill = NA, outlier.shape = NA) +
  coord_flip() +
  scale_color_manual(values = pcols) +
  labs(x = "", y = "difference in log10 MFI") +
  theme(
    legend.position = "none"
  )
pdiff
```

Box plot of individual-level MFI negative exponential rate parameter estimates

```
plambda <- ggplot(data = d_bigdrop, aes(y = lambda, x = antigenf, color = pathogen)) +
  geom_jitter() +
  geom_boxplot(color = "black", fill = NA, outlier.shape = NA) +
  scale_color_manual(values = pcols) +
  scale_y_continuous(breaks = seq(0, 0.04, by = 0.01)) +
  coord_flip(ylim = c(0, 0.04)) +
  labs(x = "", y = expression(paste("Exponential decay in IgG, rate parameter estimates (",lambda,")",sep="")) ) +
  theme(
    legend.position = "none"
  )
plambda
```

Box plot of individual-level MFI half life estimates

```
phalflife <- ggplot(data = d_bigdrop, aes(y = halflife, x = antigenf, color = pathogen)) +
  geom_jitter() +
  geom_boxplot(color = "black", fill = NA, outlier.shape = NA) +
  scale_color_manual(values = pcols) +
  scale_y_continuous(breaks = seq(0, 200, by = 20)) +
  coord_flip(ylim = c(0, 200)) +
  labs(x = "", y = "IgG half life in days, assuming exponential decay") +
  theme(
    legend.position = "none"
  )
phalflife
```

## Model-based estimates of IgG decay and half life

Here, estimate the decay rate parameters assuming an exponential model for each antibody. Save the parameter estimates and their SE to use in the simulations, below.

Note that the units for the rates are in days (not years) to align with the simulations below.

Reassuringly, the model-based estimates align very closely with the non-parametric medians displayed above in the figures, both for decay rates (\(\lambda\)) and estimates of IgG half life.

```
# Fit separate models by antibody
est_lambda <- foreach(ab = levels(d_bigdrop$antigenf), .combine = rbind) %do% {
  # reshape the data back to long format for estimation
  di <- d_bigdrop %>%
    filter(antigenf == ab) %>%
    mutate(y0 = logmfi, y1 = logmfi + logmfidiff, t1 = agediff) %>%
    select(antigenf, id, t1, y0, y1) %>%
    gather(desc, logmfi, -antigenf, -id, -t1) %>%
    arrange(antigenf, id, desc) %>%
    mutate(t = ifelse(desc == "y0", 0, t1 * 365.25)) %>%
    mutate(mfi = 10^logmfi) %>%
    select(antigenf, id, t, mfi)
  # fit an exponential model with OLS by log-transforming the outcome
  # e.g.,  https://stackoverflow.com/questions/19453861/how-to-fit-and-plot-exponential-decay-function-using-ggplot2-and-linear-approxim
  # note: when developing this approach, plotted individual trajectories against
  # fitted curves for all antibodies and this modeling assumption of exponential decay
  # looks quite reasonable.  Nearly all individual trajectories were basically parallel
  # with the fitted curve, suggesting that they all shared a common decay rate
  fiti <- summary(lm(log(mfi) ~ t, data = di))
  res <- data.frame(antigenf = ab, lambda = fiti$coefficients[2, 1], lambda_se = fiti$coefficients[2, 2])
  # estmimate IgG half life = log(2)/lambda and SE of half life using the delta method
  # where g(u) = log(2)/u and g'(u) = -log(2)/u^2 and var[g(u)] = g'(u)^2 x SE(u)^2
  res <- res %>%
    mutate(
      lambda_lb = lambda - 2 * lambda_se,
      lambda_ub = lambda + 2 * lambda_se,
      halflife = log(2) / -lambda,
      halflife_se = sqrt((-log(2) / -lambda^2)^2 * lambda_se^2),
      halflife_lb = halflife - 2 * halflife_se,
      halflife_ub = halflife + 2 * halflife_se
    )
  res
}

est_lambda_print <- est_lambda %>%
  select(antigenf, lambda, lambda_lb, lambda_ub, halflife, halflife_lb, halflife_ub)

knitr::kable(est_lambda_print,
  digits = c(0, 3, 3, 3, 0, 0, 0),
  caption = "Estimates of IgG exponential decay rates (per day) and half life in days among children in Haiti, 1990-1999. ",
  col.names = c("Antibody", "Lambda", "min95", "max95", "Half Life", "min95", "max95")
) %>%
  kable_styling(bootstrap_options = c("striped"), full_width = TRUE) %>%
  add_header_above(c(" " = 1, "IgG exponential decay rate" = 3, "IgG Half life (days)" = 3))
```

Estimates of IgG exponential decay rates (per day) and half life in days among children in Haiti, 1990-1999.

|  | IgG exponential decay rate | | | IgG Half life (days) | | |
| --- | --- | --- | --- | --- | --- | --- |
| Antibody | Lambda | min95 | max95 | Half Life | min95 | max95 |
| Giardia VSP-5 | -0.010 | -0.014 | -0.006 | 69 | 40 | 98 |
| Cryptosporidium Cp17 | -0.014 | -0.016 | -0.011 | 51 | 41 | 61 |
| E. histolytica LecA | -0.009 | -0.013 | -0.005 | 76 | 43 | 109 |
| Salmonella LPS group B | -0.010 | -0.013 | -0.007 | 70 | 48 | 93 |
| ETEC LT B subunit | -0.004 | -0.006 | -0.003 | 169 | 107 | 231 |
| Norovirus GI.4 | -0.012 | -0.016 | -0.007 | 58 | 37 | 80 |
| Norovirus GII.4.NO | -0.006 | -0.009 | -0.004 | 108 | 66 | 151 |

## Simulate daily IgG trajectories

In this section of the notebook, we simulate a child’s full longitudinal IgG trajectory for each antibody. The simulation is informed by each child’s empirical IgG measurements (timing and level), the IgG decay rates estimated in the previous section, and the empirical distribution of IgG boosting in the cohort. We use the simulated IgG trajectories to study the influence of less frequent measurement on estimates of incidence rates / force of infection.

Most measurements in the original study were separated by approximately 1 year. We hypothesized that this could lead us to miss IgG boosting events in the interim periods, and would thus provide an approximate lower bound on the serological measures of force of infection.

The simulation below is designed to estimate the highest seroconversion rates possible – those that would result from continuous boosting and waning, while ensuring that the individual IgG kinetics are consistent with the empirical data. The simulations are not intended to simulate all of the nuance of a child’s longitudinal immune development. For example, the simulation makes no assumptions for different incidence rates by age and so could over-estimate boosting rates in cases where a pathogen’s incidence declines at older ages. The simulation estimates should provide an approximate upper bound on force of infection measured with serology.

A possible limitation of using empirical estimates of IgG decay from the cohort is that there is no way to prove that intervals were free from exposure. ETEC and norovirus GII were the pathogens with the highest force of infection in the cohort, but lowest estimated IgG decay rates. It is possible that the decay rates were under-estimated in these cases from an insufficient number of longitudinal measurement periods without exposure. If decay rates are too low, then the simulation might under-estimate the upper bound of seroconversion rates possible in the cohort. Nearly all of the estimates above are close to \(\lambda = -0.01\), with the exception of ETEC LT B subunit. We suspect that few children in the study experienced two consecutive measurements in which they were not exposed to ETEC, but that would require stool-based confirmation.

### Boosting imputation algorithm

Below is an algorithm that imputes IgG levels with daily resolution for each child. These are the steps in the algorithm:

1. Start with the actual measurements in the study
2. Allow IgG to decay exponentially with rate parameter \(\hat\lambda\), conservatively estimated above using adjacent measurements with the largest decline in IgG and measurements separated by < 1 year (trying to ensure no influence of boosting in decay rate estimation).
3. For every day of the time series, draw a candidate IgG boost from the empirical distribution of \(\geq 4\)-fold IgG increases in the data.
4. Apply the candiate boost to the decaying IgG levels from previous measurement.
5. Estimate the time required for the candidate boost to decay exponentially to the next actual measurment. Denote actual IgG measures at measurement times \(t\) and \(t+1\) by \(N\_t\) and \(N\_{t+1}\). Let \(N\_{b}\) be the IgG level with the candidate boost at intermediate time \(t < b < t+1\). The time required to exponentially decay to \(N\_{t+1}\) is \(-\log (\frac{N\_{t+1}}{N\_b}) (\frac{1}{\hat\lambda})\)
6. If time required to decay is less than the time to the next measurement, and the IgG level is either below the seropositivity cutoff or has waned at least 4-fold from the previous boost (if any), then boost IgG at the earliest candidate boost.
7. Treat the new boosting events as actual measurements in the next iteration.
8. Iterate steps 2-7 until a child’s time series cannot accommodate any additional boosts (we conservatively used 10 iterations).

The function also allows for a subsample of the time series (default is no subsample) Below we illustrate how a 30-day sampling interval captures all of the IgG dynamics in the model given the empirical boosting levels and decay rates. This simulation could be sped up considerably, possibly by modeling each child’s antibody kinetics in 30-day time steps, but we have not had time to implement that refinement.

```
ab_impute <- function(d, ab, lambda, nfill = 10, samp_interval = 1) {
  # function arguments:
  # d      : dataset with quite a few specific variables (see dsim below in next chunk)
  # ab     : antibody to select for imputation
  # lambda : empirical rate of antibody decay
  # nfill  : number of passes for the imputation algorithm (default=10)
  # samp_interval : interval in days to sample from the full time series
  #                 1 = no subsample, 7 = weekly subsample, 30 = monthly subsample

  # create the full data with daily resolution
  # for each child from enrollment to final measurements
  dshell <- d %>%
    filter(antigenf == ab) %>%
    select(id,agedays) %>%
    group_by(id) %>%
    summarize(start = min(agedays), end = max(agedays)) %>%
    group_by(id) %>%
    do(data.frame(id = .$id, agedays = seq(from = .$start, to = .$end, by = 1)))

  # filter to specific antibody
  di <- d %>% 
    filter(antigenf == ab) %>%
    group_by(id) %>%
    arrange(id,agedays) %>%
    mutate(empmeas=row_number())

  # empirical distribution of IgG boosting in the sample
  # below, draw from this empirical distribution of changes
  # with at least a 4-fold increase in IgG for each boosting event
  logboosti <- di$logmfidiff[di$logmfidiff > log10(4) & !is.na(di$logmfidiff)]

  # merge the Ab measurements onto the full data
  # model log MFI decay over time since last measurement
  dis <- left_join(dshell, di, by = c("id", "agedays")) %>%
    group_by(id) %>%
    arrange(id, agedays)

  # for each measurement, store the difference in MFI
  # and the MFI value of the next measurement
  # fill in the seropositivity cutoff
  dis <- dis %>%
    group_by(id, idx = cumsum(!is.na(mfi))) %>%
    mutate(
      mfi_0 = max(mfi, na.rm = TRUE),
      mfi_diff = max(mfidiff, na.rm = TRUE),
      mfi_t1 = mfi_0 + mfi_diff
    ) %>%
    ungroup() %>%
    select(-idx, -mfi_0) %>%
    mutate(sero_cut = max(serocut, na.rm = T))

  # iterative boosting imputation algorithm
  i <- 1
  while (i <= nfill) {
    dis <- dis %>%
      ungroup() %>%
      mutate(boost_t = sample(logboosti, size = nrow(dis), replace = TRUE)) %>%
      group_by(id, idx = cumsum(!is.na(mfi))) %>%
      mutate(
        # time steps (days) between measurements
        min_t = min(agedays),
        t = agedays - min_t,
        max_t = max(t),
        # IgG decay from last measurement
        mfi_0 = max(mfi, na.rm = TRUE),
        mfi_t = as.integer(mfi_0 * exp(lambda * t)),
        # draw a candidate IgG boost from the empirical distribution (above)
        # with a ceiling at the maximum of the MFI dynamic range ~32,000
        mfi_b = as.integer(mfi_t * 10^boost_t),
        mfi_b = ifelse(mfi_b > 32000, 32000, mfi_b),
        # estimated time required to decay exponentially
        # to the next measurement
        t_b = as.integer(ifelse(mfi_b > mfi_t1 & is.finite(mfi_t1), -log(mfi_t1 / mfi_b) / -lambda, 0)),
        # indicator of whether the candidate IgG boost is plausible
        # condition 1: not the first measurement in the study
        # condition 2: IgG level is below seropos+ cutoff, or >=4-fold lower than last boost
        # condition 3: IgG levels can decay sufficiently to be at or below the next measurement
        boost = ifelse(
          (is.na(empmeas) | empmeas != 1 ) & 
          ( (mfi_t < 10^sero_cut) | (mfi_t < mfi_0 / 4) ) & 
          (max_t - t >= t_b), 
          1, 0
        ),
        # counter of the cumulative number of acceptible boosts
        # between existing measurements
        # (only the first will be used in each iterative update)
        cumboost = cumsum(boost)
      ) %>%
      ungroup() %>%
      group_by(id) %>%
      mutate(mfi = ifelse(boost - lag(boost) == 1 & cumboost == 1 & is.na(mfi), mfi_b, mfi))

    i <- i + 1
  }

  # downsample the data to the specified resolution
  # and return the data.frame
  dsub <- dis %>%
    group_by(id) %>%
    mutate(
      dnum = row_number() - 1,
      samp = ifelse(dnum %% samp_interval == 0, 1, 0)
    ) %>%
    filter(samp == 1) %>%
    select(antigenf, sero_cut, id, age, agedays, idx, mfi_t)

  return(dsub)
}
```

```
#-------------------------------
# restrict the data to essential
# variables to speed things up a bit
#-------------------------------
dsim <- dl %>%
  ungroup() %>%
  select(antigenf, id, year, week, age, mfi, logmfi, logmfidiff, serocut, seropos) %>%
  mutate(
    mfidiff = 10^logmfidiff * mfi - mfi,
    agedays = floor(age * 365.25)
  )
```

### Illustrative simulation figure

Create a didactic figure of a single child’s antibody trajectory to illustrate what the algorithm does and the influence of less frequent sampling intervals on the approximation of a child’s longitudinal IgG trajectory. Use *Cryptosporidium* Cp17 as an example.

The figures show how multiple, intermediate boosting episodes are possible while still maintaining consistency with the empirical measurements. The figures also illustrate how monthly sampling frequency appears to capture all of the kinetics in IgG based on the empirical boosting and exponential waning parameters (true in this special case, but also more generally), but that progressively lower resolution sampling intervals increasily fail to capture the true IgG boosting and waning kinetics.

```
#-------------------------------
# Pick an example pathogen
# and arbitrary child id
#-------------------------------
ab <- "Cryptosporidium Cp17"
cid <- 4951

#-------------------------------
# Resolution: daily
#-------------------------------
set.seed(12345)
d1 <- ab_impute(d = dsim,
                ab = ab,
                lambda = est_lambda$lambda[est_lambda$antigenf == ab],
                samp_interval = 1) %>%
  filter(id == cid) %>%
  mutate(t = agedays - min(agedays)) %>%
  mutate(sinterval = "daily")

#-------------------------------
# Resolution: monthly
#-------------------------------
d2 <- d1 %>%
  filter(ifelse(t %% 30 == 0, TRUE, FALSE)) %>%
  mutate(sinterval = "30 days")

#-------------------------------
# Resolution: every 3 months
#-------------------------------
d3 <- d1 %>%
  filter(ifelse(t %% 90 == 0, TRUE, FALSE)) %>%
  mutate(sinterval = "90 days")

#-------------------------------
# Resolution: every 6 months
#-------------------------------
d4 <- d1 %>%
  filter(ifelse(t %% 180 == 0, TRUE, FALSE)) %>%
  mutate(sinterval = "180 days")

#-------------------------------
# Resolution: every 12 months
#-------------------------------
d5 <- d1 %>%
  filter(ifelse(t %% 360 == 0, TRUE, FALSE)) %>%
  mutate(sinterval = "360 days")

#-------------------------------
# Bind all of the data together
# for plotting
#-------------------------------
d_all <- bind_rows(d1, d2, d3, d4, d5) %>%
  mutate(sinterval = factor(sinterval, levels = c("daily", "30 days", "90 days", "180 days", "360 days")))

d_emp <- dsim %>% filter(antigenf == ab & id == cid)

psim <- ggplot(data = d1, aes(x = agedays/365.25, y = mfi_t)) +
  geom_ribbon(aes(ymin = 0, ymax = mfi_t), fill = "black", color = NA, alpha = 0.2) +
  geom_line(color = "gray20") +
  geom_point(data = d_emp, aes(x = agedays/365.25, y = mfi), size = 2, col = cbPalette[4]) +
  geom_line(data = d_emp, aes(x = agedays/365.25, y = mfi), color = cbPalette[4], linetype = "dashed") +
  annotate("text", x = 0.75, y = 25000, label = "Empirical IgG measurements", color = cbPalette[4], hjust = 0) +
  annotate("text", x = 3.25, y = 30000, label = "Simulated boosting and waning kinetics\n(one possible trajectory)", hjust = 1) +
  scale_y_continuous(breaks = seq(0, 32000, by = 5000)) +
  scale_x_continuous(breaks = 0:5) +
  coord_cartesian(ylim=c(0,32000),xlim=c(0.5,5.5))+
  labs(x = "age in years (child id=4951)", y = "IgG Luminex response (MFI-bg)") +
  theme_minimal()
psim
```

```
pcols <- c(cbPalette[c(2, 3, 4, 8)])
psim_ds <- ggplot(data = filter(d_all, sinterval != "daily"), aes(x = agedays/365.25, y = mfi_t, color = sinterval)) +
  geom_ribbon(data = d1, aes(x = agedays/365.25, ymin = 0, ymax = mfi_t), fill = "black", color = NA, alpha = 0.3) +
  geom_line(lwd = 0.8) +
  scale_y_continuous(breaks = seq(0, 32000, by = 5000)) +
  scale_x_continuous(breaks = 0:5) +
  scale_color_manual(values = pcols, guide = guide_legend(title = "sampling interval")) +
  scale_size_manual(values = c(rep(0.9, 4), 0.2), guide = FALSE) +
  labs(x = "age in years (child id=4951)", y = "IgG Luminex response (MFI-bg)") +
  coord_cartesian(ylim=c(0,32000),xlim=c(0.5,5.5))+
  theme_minimal() +
  theme(
    legend.position = c(0.8,0.8)
  )
psim_ds
```

```
# save the figures
# ggsave(here::here("figs", "haiti-interval-sim-example1.pdf"), plot = psim, device = cairo_pdf, width = 7, height = 5)
# ggsave(here::here("figs", "haiti-interval-sim-example2.pdf"), plot = psim_ds, device = cairo_pdf, width = 7, height = 5)
```

### Simulate 100 datasets

Use the boosting imputation algorithm described above to simulate 100 datasets with daily resolution for each child’s IgG levels for each antibody. Downsample the time series to IgG measures every 30 days.

```
#-------------------------------
# simulate 100 datasets,
# stored in a big data.frame
#-------------------------------
start_time <- Sys.time()
set.seed(123)
nsim <- 100
simdata <- foreach(ab = levels(dsim$antigenf), .combine = rbind) %:%
  foreach(simi = 1:nsim, .combine = rbind) %dopar% {
    di <- dsim %>% filter(antigenf == ab)
    dimp <- ab_impute(
      d = di,
      ab = ab,
      lambda = est_lambda$lambda[est_lambda$antigenf == ab],
      nfill = 10,
      samp_interval = 30
    ) %>%
      mutate(antigenf = ab, simnum = simi)
    dimp
  }

end_time <- Sys.time()
end_time - start_time
```

```
## Time difference of 51.1155 mins
```

```
#-------------------------------
# save simulation data since
# it takes some time to generate
#-------------------------------
saveRDS(simdata, file = here::here("output", "haiti-imputation-simdata.rds"))
```

## Seroconversion rate estimates

Estimates of the seroconversion rates. The rates assume the incident seroconversion or seroreversion events occurred at the midpoint between measurements.

```
#-----------------------------
# load the simulated datasets
# add a pathogen field to
# combine information across
# antibodies for seroconversion
#-----------------------------
simdata <- readRDS(file = here::here("output", "haiti-imputation-simdata.rds"))
```

```
#-----------------------------
# from each simulated dataset:
# for each antibody measurement
# summarize seroconversion and
# seroreversion rates with
# sampling intervals of
# 30, 90, 180, and 360 days
#
# this results in 100 estimates
# for each antibody and scenario
# the variation in estimates
# is not from child sampling variability
# but instead from the stochastic
# IgG boosting and waning process
#-----------------------------
start_time <- Sys.time()
sim_res <- foreach(sintervali=c(30,90,180,360),.combine=rbind) %dopar% {
  # downsample the data, depending on the sampling interval
    di1 <- simdata %>%
      group_by(antigenf,simnum,id) %>%
      mutate(t = agedays - min(agedays)) %>%
      filter(ifelse(t %% sintervali == 0, TRUE, FALSE))
    
    di2 <- di1 %>%
      mutate(
        # calculate difference in person-years between measurements
        agediff = (agedays - lag(agedays)) / 365.25,
        # incident seroconversions and reversions
        # based on crossing seropositivity cutoff
        seropos = ifelse(log10(mfi_t)>sero_cut,1,0),
        seropos_lag = lag(seropos),
        seroi = ifelse(seropos == 1 & seropos_lag == 0, 1, 0),
        seroi = ifelse(is.na(agediff), NA, seroi),
        seror = ifelse(seropos == 0 & seropos_lag == 1, 1, 0),
        seror = ifelse(is.na(agediff), NA, seror),
        # estimate person-time at risk, 
        # assuming events occur the mid-point of measures
        ptc = ifelse(seropos==0,agediff,0),
        ptc = ifelse(seropos==1 & seroi==1,agediff/2,ptc),
        ptr = ifelse(seropos==1 & seroi==0,agediff,0),
        ptr = ifelse(seropos==0 & seror==1,agediff/2,ptr)
        )
    
    #-----------------------------
    # estimate incidence rates
    # units are episodes per child-year
    #-----------------------------
    rate_ests <- di2 %>%
      group_by(antigenf,simnum) %>%
      summarize(
        ni = sum(seroi, na.rm = T),
        nit = sum(ptc, na.rm = T),
        nr = sum(seror, na.rm = T),
        nrt = sum(ptr, na.rm = T)
      ) %>%
      mutate(seroi = ni / nit, 
             seror = nr / nrt
      ) %>%
      mutate(sinterval = sintervali)
    
    rate_ests
}
end_time <- Sys.time()
end_time - start_time
```

```
## Time difference of 58.75143 secs
```

```
#-----------------------------
# format the simulation results
#-----------------------------
sim_res2 <- sim_res %>%
  ungroup() %>%
  mutate(antigenf=factor(antigenf,levels=levels(dsim$antigenf))) %>%
  select(antigenf,sinterval,simnum,everything()) %>%
  # estimate the rate difference compared with a 30 day sampling interval
  group_by(antigenf,simnum) %>%
  mutate(
    seroi30 = max(ifelse(sinterval==30,seroi,-99)),
    seror30 = max(ifelse(sinterval==30,seror,-99)),
    diff30i = seroi-seroi30,
    diff30r = seror-seror30
  ) %>%
  select(-seroi30,-seror30)

#-----------------------------
# save the simulation results
#-----------------------------
saveRDS(sim_res2,file=here::here("output","haiti-imputation-simulation-results.rds"))
```

## Estimate empirical seroconversion rates

In the primary analysis, we combined information across multiple antigens for each pathogen when we estimated seroconversion and seroreversion rates. In this simulation, we did not attempt to model the joint dynamics of multiple antigens. To benchmark the simulation, we need to estimate seroconversion rates using information from only a single antibody response for each pathogen. The differences between estimates based on joint- vs. single antigen seroconversion rates are slight, but this helps ensure a consistent benchmark for the simulation results.

```
#-----------------------------
# estimate seroincidence rates
# for conversion and reversion
# estimate SEs with a bootstrap
#-----------------------------

#-----------------------------
# identify incident 
# seroconversions and reversions
#-----------------------------

# group the data by child and
# use lags to identify
# time in years between measurements,
# sero-conversions + sero-reversions 
# between measurements
# set the first measurement to 
# missing for the incidence indicators
# (using the is.na(age_diff) to identify them)
dl3 <- dl2 %>%
  group_by(antigenf,id) %>% 
  arrange(antigenf,id,age) %>%
  mutate(age_min  = min(age),
         agediff = age - lag(age),
         # incident seroconversions and reversions
         # based on crossing seropositivity cutoff
         seropos_lag  = lag(seropos),
         seroi = ifelse(seropos==1 & seropos_lag==0,1,0),
         seroi = ifelse(is.na(agediff),NA,seroi),
         seror = ifelse(seropos==0 & seropos_lag==1,1,0),
         seror = ifelse(is.na(agediff),NA,seror)
         ) %>%
  select(
         antigenf,id,
         age, agediff,
         seropos,seroi,seror
  )

#-----------------------------
# estimate time at risk
# for seroconversion and reversion
# assumed to be 1/2 of time
# between measurements
# if indivs are seropositive
# at measurement 1 they are
# not at risk for seroconversion
# (and vice-versa for seroreversion)
#-----------------------------
dl4<- dl3 %>%
  mutate(ptc = ifelse(seropos==0,agediff,0),
        ptc = ifelse(seropos==1 & seroi==1,agediff/2,ptc),
        ptr = ifelse(seropos==1 & seroi==0,agediff,0),
        ptr = ifelse(seropos==0 & seror==1,agediff/2,ptr)
  )

#-----------------------------
# estimate incidence rates
# for each antibody
# units are per child-year
#-----------------------------
rate_ests <- dl4 %>%
  group_by(antigenf) %>%
  summarize(ni=sum(seroi,na.rm=T),
            nit=sum(ptc,na.rm=T),
            nr=sum(seror,na.rm=T),
            nrt=sum(ptr,na.rm=T)) %>%
  mutate(seroi=ni/nit,
         seror=nr/nrt)

#-----------------------------
# estimate bootstrap CIs
# resampling children with 
# replacement due to repeated
# measures
#-----------------------------
dboot <- dl4 %>%
  group_by(antigenf) %>%
  select(id,antigenf,seroi,seror,ptc,ptr) %>%
  mutate(id=as.character(id))

set.seed(123)
nreps <- 1000
ids <- unique(dboot$id)
bsamp <- matrix(sample(ids,size=length(ids)*nreps,replace=TRUE),
                nrow=length(ids),ncol=nreps)
bootests <- foreach(ab=levels(dboot$antigenf),.combine=rbind) %:%
  foreach(brep=1:nreps,.combine=rbind) %dopar% {
    di <- dboot %>% filter(antigenf==ab)
    di <- left_join(data.frame(id=bsamp[,brep]),di,by=c("id")) %>%
      select(-id) %>% group_by(antigenf) %>%
      summarize_all(function(x) sum(x,na.rm=TRUE))
    }

rate_cis <- bootests %>% 
  group_by(antigenf) %>%
  mutate(sero_c=seroi/ptc,sero_r=seror/ptr) %>%
  summarize(seroi_lb = quantile(sero_c,probs=c(0.025),na.rm=T),
            seroi_ub = quantile(sero_c,probs=c(0.975),na.rm=T),
            seror_lb = quantile(sero_r,probs=c(0.025),na.rm=T),
            seror_ub = quantile(sero_r,probs=c(0.975),na.rm=T)
            )

rate_ests <- left_join(rate_ests,rate_cis,by="antigenf") %>%
  select(antigenf,ni,nit,starts_with("seroi"),nr,nrt,starts_with("seror"))

knitr::kable(rate_ests,
  digits = c(0, 0,2,2,2,2, 0,2,2,2,2),
  caption = "Estimates of seroconversion and seroreversion rates among children in Haiti, 1990-1999.",
  col.names = c("Antibody", "events", "child-years\nat risk", "rate", "min95", "max95", "events", "child-years\nat risk", "rate", "min95", "max95")
) %>%
  kable_styling(bootstrap_options = c("striped"), full_width = TRUE) %>%
  add_header_above(c(" " = 1, "Seroconverion" = 5, "Seroreversion" = 5))
```

Estimates of seroconversion and seroreversion rates among children in Haiti, 1990-1999.

|  | Seroconverion | | | | | Seroreversion | | | | |
| --- | --- | --- | --- | --- | --- | --- | --- | --- | --- | --- |
| Antibody | events | child-years at risk | rate | min95 | max95 | events | child-years at risk | rate | min95 | max95 |
| Giardia VSP-5 | 109 | 270.93 | 0.40 | 0.34 | 0.48 | 91 | 439.62 | 0.21 | 0.17 | 0.25 |
| Cryptosporidium Cp17 | 81 | 155.73 | 0.52 | 0.44 | 0.62 | 44 | 542.37 | 0.08 | 0.06 | 0.11 |
| E. histolytica LecA | 97 | 283.71 | 0.34 | 0.28 | 0.42 | 43 | 395.22 | 0.11 | 0.08 | 0.15 |
| Salmonella LPS group B | 83 | 153.07 | 0.54 | 0.46 | 0.65 | 30 | 520.85 | 0.06 | 0.04 | 0.08 |
| ETEC LT B subunit | 11 | 9.72 | 1.13 | 0.75 | 1.82 | 2 | 702.11 | 0.00 | 0.00 | 0.01 |
| Norovirus GI.4 | 80 | 213.01 | 0.38 | 0.30 | 0.47 | 28 | 464.89 | 0.06 | 0.03 | 0.09 |
| Norovirus GII.4.NO | 67 | 105.82 | 0.63 | 0.51 | 0.80 | 19 | 574.35 | 0.03 | 0.02 | 0.05 |

## Figure: seroconversion rates

**Figure caption:** Seroconversion rates (force of infection) estimated through simulation of daily IgG boosting and waning kinetics with different measurement frequencies. Simulations were based on antibody boosting and decay parameters estimated from Haitian children ages 0-11 years old. Points represent each of the 100 estimates from the simulated datasets and box plots summarize their distribution. Horizontal dashed lines and shaded regions mark the empirical seroconversion rates and 95% confidence intervals estimated in Haitian cohort, which had an approximately annual measurement frequency.

```
sim_res_plot <- sim_res2 %>%
  ungroup() %>%
  mutate(antigenf=factor(antigenf,levels=ab_in_sim[c(1:4,6,7,5)]),
         sinterval=factor(sinterval)
  )

pcols <- cbPalette[c(2,3,4,6,8,8,7)]
simres_ratep <- ggplot(data=sim_res_plot,aes(y=seroi,color=antigenf)) +
  facet_wrap(~antigenf,ncol=2,scales="free_y")+
  geom_boxplot(aes(x=sinterval),fill=NA,outlier.shape=NA,color=NA)+
  # add shading for rates estimated in the study
  geom_rect(data=rate_ests,
            aes(ymin=seroi_lb,ymax=seroi_ub,xmin=0,xmax=5),
            color=NA,alpha=0.2)+
  geom_hline(data=rate_ests,aes(yintercept=seroi),color="gray40",linetype="dashed")+
  # add simulation points and box plots
  geom_jitter(aes(x=sinterval),width=0.25,alpha=0.3,pch=19,size=0.3)+
  geom_boxplot(aes(x=sinterval),fill=NA,outlier.shape=NA,color="black",lwd=0.2)+
  
  # scale_y_continuous(breaks=seq(0,2,by=0.5))+
  scale_color_manual(values=pcols)+
  coord_cartesian(xlim=c(1,4))+ # ylim=c(0,2),
  labs(y="seroconversion rate per child-year (force of infection)",x="sampling interval, days") +
  theme_minimal()+
  theme(
    legend.position="none",
    strip.text=element_text(size=8)
  )

simres_ratep
```

```
# save a figure
# ggsave(filename = here::here("figs","haiti-interval-sim-rate-ests.pdf"), plot = simres_ratep,device = cairo_pdf, width = 3.5, height = 6)

# print medians
sim_res_plot %>%
  group_by(antigenf,sinterval) %>%
  select(antigenf,sinterval,seroi,seror) %>%
  summarize_all(median) %>%
  knitr::kable(
  digits = c(0,0,2,2),
  caption = "Median seroconversion and seroreversion rates per year at risk across 100 simulated datasets downsampled at different frequencies.",
  col.names = c("Antibody", "Sampling\ninterval (days)","Seroconversion\nrate","Seroreversion\nrate")
) %>%
  kable_styling(bootstrap_options = c("striped"), full_width = TRUE)
```

Median seroconversion and seroreversion rates per year at risk across 100 simulated datasets downsampled at different frequencies.

| Antibody | Sampling interval (days) | Seroconversion rate | Seroreversion rate |
| --- | --- | --- | --- |
| Giardia VSP-5 | 30 | 1.20 | 0.41 |
| Giardia VSP-5 | 90 | 0.89 | 0.32 |
| Giardia VSP-5 | 180 | 0.64 | 0.22 |
| Giardia VSP-5 | 360 | 0.45 | 0.16 |
| Cryptosporidium Cp17 | 30 | 1.28 | 0.18 |
| Cryptosporidium Cp17 | 90 | 1.00 | 0.14 |
| Cryptosporidium Cp17 | 180 | 0.78 | 0.11 |
| Cryptosporidium Cp17 | 360 | 0.58 | 0.08 |
| E. histolytica LecA | 30 | 0.93 | 0.32 |
| E. histolytica LecA | 90 | 0.76 | 0.27 |
| E. histolytica LecA | 180 | 0.59 | 0.19 |
| E. histolytica LecA | 360 | 0.44 | 0.12 |
| Salmonella LPS group B | 30 | 1.42 | 0.17 |
| Salmonella LPS group B | 90 | 1.09 | 0.13 |
| Salmonella LPS group B | 180 | 0.83 | 0.08 |
| Salmonella LPS group B | 360 | 0.59 | 0.05 |
| Norovirus GI.4 | 30 | 0.81 | 0.19 |
| Norovirus GI.4 | 90 | 0.68 | 0.17 |
| Norovirus GI.4 | 180 | 0.54 | 0.12 |
| Norovirus GI.4 | 360 | 0.39 | 0.07 |
| Norovirus GII.4.NO | 30 | 1.60 | 0.11 |
| Norovirus GII.4.NO | 90 | 1.27 | 0.09 |
| Norovirus GII.4.NO | 180 | 0.97 | 0.05 |
| Norovirus GII.4.NO | 360 | 0.72 | 0.03 |
| ETEC LT B subunit | 30 | 6.23 | 0.00 |
| ETEC LT B subunit | 90 | 3.81 | 0.00 |
| ETEC LT B subunit | 180 | 2.63 | 0.00 |
| ETEC LT B subunit | 360 | 1.69 | 0.00 |

The results of the simulations show that seroconversion rates estimated with a 360 day sampling interval were internally consistent with the empirical estimates from the cohort. The simulations show that in cases of nearly continuous IgG boosting and waning, less frequenty measured cohorts will underestimate force of infection, but the magnitude of difference was < 1 episode per year for all pathogens except for ETEC (below).

## Figure: difference in rates

**Figure caption:** Simulation-based estimates of the difference in seroconversion rates (force of infection) in the Haitian cohort with longer sampling intervals. Estimates were generated through simulation of longitudinal IgG boosting and waning kinetics with daily resolution using boosting and decay parameters estimated from Haitian children ages 0-11 years old, which were then down-sampled at different frequencies. Points represent each of the 100 estimates from the simulated datasets and horizontal lines indicate median differences from the reference sampling interval of 30 days. Across pathogens, median differences in rates were < 1 episode per year with the exception of ETEC LT B subunit, which was higher.

```
pcols <- cbPalette[c(2:4,6,8,8,7)]
sim_res_diff <- sim_res_plot 

simres_diffp <- ggplot(data=sim_res_diff,aes(x=factor(sinterval),y=diff30i,color=antigenf)) +
  facet_wrap(~antigenf,ncol=2,scales="free_y")+
  geom_jitter(width=0.15,alpha=0.3,pch=19,size=0.5)+
  stat_summary(fun.y = median, fun.ymin = median, fun.ymax = median,
                 geom = "crossbar", width = 0.5,color="black",size=0.2)+
  annotate("text",x=1.5,y=0,label="ref.",size=2,color="gray20")+
  scale_color_manual(values=pcols)+
  labs(y="difference in seroconversion rate per child-year, 100 simulations",x="sampling interval, days") +
  theme_minimal()+
  theme(
    legend.position="none",
    strip.text=element_text(size=8)
  )

simres_diffp
```

```
# save a figure
# ggsave(filename = here::here("figs","haiti-interval-sim-rate-diff.pdf"), plot = simres_diffp,device = cairo_pdf, width = 3.5, height = 6)

# print median differences
sim_res_diff %>%
  group_by(antigenf,sinterval) %>%
  select(antigenf,sinterval,diff30i,diff30r) %>%
  summarize_all(median) %>%
  knitr::kable(
  digits = c(0,0,2,2),
  caption = "Median difference in seroconversion and seroreversion rates per year at risk across 100 simulated datasets downsampled at different frequencies, with a 30-day sampling interval as the reference.",
  col.names = c("Antibody", "Sampling\ninterval (days)","Seroconversion\nrate","Seroreversion\nrate")
) %>%
  kable_styling(bootstrap_options = c("striped"), full_width = TRUE)
```

Median difference in seroconversion and seroreversion rates per year at risk across 100 simulated datasets downsampled at different frequencies, with a 30-day sampling interval as the reference.

| Antibody | Sampling interval (days) | Seroconversion rate | Seroreversion rate |
| --- | --- | --- | --- |
| Giardia VSP-5 | 30 | 0.00 | 0.00 |
| Giardia VSP-5 | 90 | -0.32 | -0.08 |
| Giardia VSP-5 | 180 | -0.57 | -0.19 |
| Giardia VSP-5 | 360 | -0.76 | -0.25 |
| Cryptosporidium Cp17 | 30 | 0.00 | 0.00 |
| Cryptosporidium Cp17 | 90 | -0.29 | -0.03 |
| Cryptosporidium Cp17 | 180 | -0.51 | -0.07 |
| Cryptosporidium Cp17 | 360 | -0.70 | -0.10 |
| E. histolytica LecA | 30 | 0.00 | 0.00 |
| E. histolytica LecA | 90 | -0.18 | -0.05 |
| E. histolytica LecA | 180 | -0.35 | -0.13 |
| E. histolytica LecA | 360 | -0.49 | -0.20 |
| Salmonella LPS group B | 30 | 0.00 | 0.00 |
| Salmonella LPS group B | 90 | -0.33 | -0.03 |
| Salmonella LPS group B | 180 | -0.60 | -0.09 |
| Salmonella LPS group B | 360 | -0.83 | -0.12 |
| Norovirus GI.4 | 30 | 0.00 | 0.00 |
| Norovirus GI.4 | 90 | -0.13 | -0.03 |
| Norovirus GI.4 | 180 | -0.27 | -0.08 |
| Norovirus GI.4 | 360 | -0.42 | -0.12 |
| Norovirus GII.4.NO | 30 | 0.00 | 0.00 |
| Norovirus GII.4.NO | 90 | -0.34 | -0.02 |
| Norovirus GII.4.NO | 180 | -0.63 | -0.06 |
| Norovirus GII.4.NO | 360 | -0.89 | -0.08 |
| ETEC LT B subunit | 30 | 0.00 | 0.00 |
| ETEC LT B subunit | 90 | -2.28 | 0.00 |
| ETEC LT B subunit | 180 | -3.60 | 0.00 |
| ETEC LT B subunit | 360 | -4.54 | 0.00 |

# Kenya

## Load the Kenya data

```
#-----------------------------
# load the formatted data
# created with
# asembo-enteric-ab-data-format.Rmd ->
# Fig1sup3-asembo-ab-distributions.Rmd
#-----------------------------
dl <- readRDS(here::here("data", "asembo_analysis2.rds"))
```

## Estimates of IgG decay and half life

Identify longitudinal measurements where children experienced larger drops in IgG.

The goal is to try to identify periods of IgG waning that were unlikely to be influenced by intermediate boosting between measurements.

The code below selects, for each antibody, adjacent measurements within a child where IgG MFI declined between measurements, and from those take the largest 20% reductions (bottom quintile). Then, restrict to measurements separated by less than 1 year, and estimate IgG decay rates among this subset of measurements.

Under an exponential decay model where IgG level at time \(t\) is \(N\_t\) and the decay rate parameter is \(\lambda\), \(N\_t = N\_0e^{(-\lambda t)}\), \(\lambda = \frac{-\log(N\_t/N\_0)}{t}\), and the half life is \(t\_{1/2}=\log(2)/\lambda\).

```
# restrict to a single antigen per pathogen because the simulations
# do not attempt to model the joint distribution of antibody responses
# filter to children with repeated measures
# calculate difference in age and restrict data to needed variables
ab_in_sim <- c("Giardia VSP-5","Cryptosporidium Cp17","E. histolytica LecA","Salmonella LPS group B","ETEC LT B subunit","Campylobacter p18")
dl2 <- dl %>%
  ungroup() %>%
  filter(antigenf %in% ab_in_sim) %>%
  mutate(antigenf=droplevels(antigenf),
         antigenf=factor(antigenf,levels=ab_in_sim)
  ) %>%
  group_by(antigenf, childid) %>%
  mutate(nobs=n()) %>% filter(nobs==2) %>% select(-nobs) %>%
  arrange(antigenf, childid, age) %>%
  select(pathogen, antigen, antigenf, childid, time,age, agediff, serocut,seropos, mfi, logmfi, logmfidiff)
```

```
# filter to drops in IgG, identify quintiles and select the lowest quintile
# the estimate the decay rate in MFI per day
# filter to observations less than 1 year of one-another to make the assumption
# of no boosting between measurements more plausible.
#
# Rough estimates of half life among this group of measurements
# with large drops assuming an exponential decay
# lambda = -log(N(t)/N(o))*(1/t); t1/2 = log2/lambda
# or
# t_1/2 = t / log_{1/2}(N(t)/N(o))
#
# Note: there are a few values that are 0 on the log10
# scale. Drop these observations to avoid problems with log transformation.
d_bigdrop <- dl2 %>%
  ungroup() %>%
  group_by(antigenf) %>%
  filter(logmfidiff < 0 & time=="A") %>%
  filter(logmfi + logmfidiff != 0) %>%
  mutate(qmfidiff = cut(logmfidiff,
    breaks = quantile(logmfidiff, probs = seq(0, 1, 0.2)),
    labels = F
  )) %>%
  filter(qmfidiff == 1) %>%
  mutate(mfidiff = 10^logmfidiff * mfi - mfi) %>%
  mutate(
    decayrate = logmfidiff / (agediff * 30.4167),
    lambda = -log((mfi + mfidiff) / mfi) / (agediff * 30.4167),
    halflife = log(2) / lambda,
    halflife2 = (agediff * 30.4167) / log((mfi + mfidiff) / mfi, base = 0.5),
    n=1
  )
```

```
# summarize ages (in months) by antibody
d_bigdrop %>% summarize(n=sum(n), mean = mean(age), med = median(age), min = min(age), max = max(age))
```

```
# summarize MFI drops by antibody
d_bigdrop %>% summarize(n=sum(n), mean = mean(logmfidiff), sd = sd(logmfidiff), min = min(logmfidiff), max = max(logmfidiff))
```

```
# summarize exponential rate parameter by antibody
d_bigdrop %>% summarize(n=sum(n), mean = mean(lambda), sd = sd(lambda), min = min(lambda), max = max(lambda))
```

```
# summarize MFI half life estimates (days) by antibody
d_bigdrop %>% summarize(n=sum(n), mean = mean(halflife, na.rm = T), sd = sd(halflife, na.rm = T), median = median(halflife, na.rm = T), min = min(halflife, na.rm = T), max = max(halflife, na.rm = T))
```

Box plot of MFI decay

```
pcols <- cbPalette[c(2:4, 6:8)]
pdiff <- ggplot(data = d_bigdrop, aes(y = logmfidiff, x = antigenf, color = pathogen)) +
  geom_jitter() +
  geom_boxplot(color = "black", fill = NA, outlier.shape = NA) +
  coord_flip() +
  scale_color_manual(values = pcols) +
  labs(x = "", y = "difference in log10 MFI") +
  theme(
    legend.position = "none"
  )
pdiff
```

Box plot of individual-level MFI negative exponential rate parameter estimates

```
plambda <- ggplot(data = d_bigdrop, aes(y = lambda, x = antigenf, color = pathogen)) +
  geom_jitter() +
  geom_boxplot(color = "black", fill = NA, outlier.shape = NA) +
  scale_color_manual(values = pcols) +
  scale_y_continuous(breaks = seq(0, 0.02, by = 0.005)) +
  coord_flip(ylim = c(0, 0.02)) +
  labs(x = "", y = expression(paste("Exponential decay in IgG, rate parameter estimates (",lambda,")",sep=""))) +
  theme(
    legend.position = "none"
  )
plambda
```

Box plot of individual-level MFI half life estimates

```
phalflife <- ggplot(data = d_bigdrop, aes(y = halflife, x = antigenf, color = pathogen)) +
  geom_jitter() +
  geom_boxplot(color = "black", fill = NA, outlier.shape = NA) +
  scale_color_manual(values = pcols) +
  scale_y_continuous(breaks = seq(0, 300, by = 20)) +
  coord_flip(ylim = c(0, 300)) +
  labs(x = "", y = "IgG half life in days, assuming exponential decay") +
  theme(
    legend.position = "none"
  )
phalflife
```

In the Asembo cohort, there were very few children who experienced reductions in IgG between measurements (approx 10 - 20 per antibody), and so we were uncomfortable estimating model-based IgG decay rate parameters for each parameter (\(\lambda\)). Since the distributions of half life estimates are reasonably consistent across pathogens (ETEC is a clear exception), and are consistent with distributions in the Haiti cohort (earlier in the notebook), the simulations below assume a common exponential decay parameter of \(\lambda = 0.01\), which corresponds to a half life of \(\log(2)/\lambda = 69\) days. This is broadly consistent with the distributions above, with the exception of ETEC, where we had scant information about IgG decay in this cohort.

## Simulate daily IgG trajectories

As in the Haiti cohort, simulate daily IgG trajectories for each child in the Asembo, Kenya cohort.

### Illustrative simulation figure

```
#-------------------------------
# restrict the data to essential
# variables to speed things up a bit
#
# only include the diff in IgG
# for the first obs to be consistent
# with the Haiti data structure
# (necessary for the simulation 
#  function ab_impute() to work properly)
#-------------------------------
dsim <- dl2 %>%
  ungroup() %>%
  select(antigenf, childid, time, age, mfi, logmfi, logmfidiff, serocut, seropos) %>%
  mutate(
    logmfidiff = ifelse(time == "A", logmfidiff, NA),
    mfidiff = 10^logmfidiff * mfi - mfi,
    agedays = floor(age * 30.4167),
    id=as.character(childid)
  )
```

```
#-------------------------------
# Pick an example pathogen
# and arbitrary child id
# use the same seed for each
# dataset created to ensure they
# have the identical draws from
# the empirical distribution of
# IgG boosts
#-------------------------------
ab <- "Cryptosporidium Cp17"
cid <- "10"

#-------------------------------
# Resolution: daily
#-------------------------------
set.seed(1)
d1 <- ab_impute(d = dsim, ab = ab, lambda = -0.01, samp_interval = 1) %>%
  filter(id == cid) %>%
  mutate(t = agedays - min(agedays)) %>%
  mutate(sinterval = "daily")

#-------------------------------
# Resolution: monthly
#-------------------------------
d2 <- d1 %>%
  filter(ifelse(t %% 30 == 0, TRUE, FALSE)) %>%
  mutate(sinterval = "30 days")

#-------------------------------
# Resolution: every 3 months
#-------------------------------
d3 <- d1 %>%
  filter(ifelse(t %% 90 == 0, TRUE, FALSE)) %>%
  mutate(sinterval = "90 days")

#-------------------------------
# Resolution: every 6 months
#-------------------------------
d4 <- d1 %>%
  filter(ifelse(t %% 180 == 0, TRUE, FALSE)) %>%
  mutate(sinterval = "180 days")

#-------------------------------
# Bind all of the data together
# for plotting
#-------------------------------
d_all <- bind_rows(d1, d2, d3, d4) %>%
  mutate(sinterval = factor(sinterval, levels = c("daily", "30 days", "90 days", "180 days", "360 days")))

d_emp <- dsim %>% filter(antigenf == ab & id == cid)

psim <- ggplot(data = d1, aes(x = agedays, y = mfi_t)) +
  geom_ribbon(aes(ymin = 0, ymax = mfi_t), fill = "black", color = NA, alpha = 0.2) +
  geom_line(color = "gray20") +
  geom_point(data = d_emp, aes(x = agedays, y = mfi), size = 2, col = cbPalette[4]) +
  geom_line(data = d_emp, aes(x = agedays, y = mfi), color = cbPalette[4], linetype = "dashed") +
  annotate("text", x = 300, y = 175, label = "Empirical measurements", color = cbPalette[4], hjust = 0) +
  annotate("text", x = 350, y = 500, label = "Simulated boosting and waning kinetics", hjust = 0) +
  scale_y_continuous(breaks = seq(0, 800, by = 200)) +
  scale_x_continuous(breaks = seq(250, 450, by = 50)) +
  coord_cartesian(ylim=c(0,800),xlim=c(250,450))+
  labs(x = "age in days (child id=10)", y = "IgG Luminex response (MFI-bg)") +
  theme_minimal()
psim
```

```
pcols <- c(cbPalette[c(2, 3, 4, 8)])
psim_ds <- ggplot(data = filter(d_all, sinterval != "daily"), aes(x = agedays, y = mfi_t, color = sinterval)) +
  geom_ribbon(data = d1, aes(x = agedays, ymin = 0, ymax = mfi_t), fill = "black", color = NA, alpha = 0.3) +
  geom_line(lwd = 0.8) +
  scale_y_continuous(breaks = seq(0, 800, by = 200)) +
  scale_x_continuous(breaks = seq(250, 450, by = 50)) +
  coord_cartesian(ylim=c(0,800),xlim=c(250,450))+
  scale_color_manual(values = pcols, guide = guide_legend(title = "sampling interval")) +
  scale_size_manual(values = c(rep(0.9, 4), 0.2), guide = FALSE) +
  labs(x = "age in days (child id=10)", y = "IgG Luminex response (MFI-bg)") +
  theme_minimal() +
  theme(
    legend.position = "top"
  )
psim_ds
```

### Simulate 100 datasets

Use the boosting imputation algorithm described above to simulate 100 datasets with daily resolution for each child’s IgG levels for each antibody. Downsample the time series to IgG measures every 10 days.

```
#-------------------------------
# simulate 100 datasets,
# stored in a big data.frame
#-------------------------------
start_time <- Sys.time()
set.seed(123)
nsim <- 100
simdata <- foreach(ab = levels(dsim$antigenf), .combine = rbind) %:%
  foreach(simi = 1:nsim, .combine = rbind) %dopar% {
    di <- dsim %>% filter(antigenf == ab)
    dimp <- ab_impute(
      d = di,
      ab = ab,
      lambda = -0.01,
      nfill = 10,
      samp_interval = 10
    ) %>%
      mutate(antigenf = ab, simnum = simi)
    dimp
  }

end_time <- Sys.time()
end_time - start_time
```

```
## Time difference of 2.654705 hours
```

```
#-------------------------------
# save simulation data since
# it takes some time to generate
#-------------------------------
saveRDS(simdata, file = here::here("output", "asembo-imputation-simdata.rds"))
```

## Seroconversion rate estimates

Estimates of the seroconversion rates. The rates assume the incident seroconversion or seroreversion events occurred at the midpoint between measurements.

In the Asembo simulations, we simulated measurement frequencies of 30, 90, and 180 days. We did not simulate a 360 day sampling interval because the cohort was only followed up for 6 months (approximately 180 days).

```
#-----------------------------
# load the simulated datasets
# add a pathogen field to
# combine information across
# antibodies for seroconversion
#-----------------------------
simdata <- readRDS(file = here::here("output", "asembo-imputation-simdata.rds"))
```

```
#-----------------------------
# from each simulated dataset:
# for each antibody measurement
# summarize seroconversion and
# seroreversion rates with
# sampling intervals of
# 30, 90, and 180 days
#
# this results in 100 estimates
# for each antibody and scenario
#-----------------------------
start_time <- Sys.time()
sim_res <- foreach(sintervali=c(30,90,180),.combine=rbind) %dopar% {
  # downsample the data, depending on the sampling interval
  # include each child's final measurement in the Kenya cohort 
  # to ensure consistency because in many cases measurements 
  # exceeded 180 days (just slightly) 
    di1 <- simdata %>%
      group_by(antigenf,simnum,id) %>%
      mutate(t = agedays - min(agedays)) %>%
      filter(ifelse(t %% sintervali == 0 | !is.na(age), TRUE, FALSE))
    
    di2 <- di1 %>%
      mutate(
        # calculate difference in person-years between measurements
        agediff = (agedays - lag(agedays)) / 365.25,
        # incident seroconversions and reversions
        # based on crossing seropositivity cutoff
        seropos = ifelse(log10(mfi_t)>sero_cut,1,0),
        seropos_lag = lag(seropos),
        seroi = ifelse(seropos == 1 & seropos_lag == 0, 1, 0),
        seroi = ifelse(is.na(agediff), NA, seroi),
        seror = ifelse(seropos == 0 & seropos_lag == 1, 1, 0),
        seror = ifelse(is.na(agediff), NA, seror),
        # estimate person-time at risk, 
        # assuming events occur the mid-point of measures
        ptc = ifelse(seropos == 0, agediff, 0),
        ptr = ifelse(seropos == 1, agediff, 0),
        ptc = ifelse(seroi == 1 | seror == 1 , agediff / 2, ptc),
        ptr = ifelse(seroi == 1 | seror == 1, agediff / 2, ptr)
        )
    
    #-----------------------------
    # estimate incidence rates
    # units are episodes per child-year
    #-----------------------------
    rate_ests <- di2 %>%
      group_by(antigenf,simnum) %>%
      summarize(
        ni = sum(seroi, na.rm = T),
        nit = sum(ptc, na.rm = T),
        nr = sum(seror, na.rm = T),
        nrt = sum(ptr, na.rm = T)
      ) %>%
      mutate(seroi = ni / nit, 
             seror = nr / nrt
      ) %>%
      mutate(sinterval = sintervali)
    
    rate_ests
}
end_time <- Sys.time()
end_time - start_time
```

```
## Time difference of 31.82011 secs
```

```
#-----------------------------
# format the simulation results
#-----------------------------
sim_res2 <- sim_res %>%
  ungroup() %>%
  mutate(antigenf=factor(antigenf,levels=levels(dsim$antigenf))) %>%
  select(antigenf,sinterval,simnum,everything()) %>%
  # estimate the rate difference compared with a 30 day sampling interval
  group_by(antigenf,simnum) %>%
  mutate(
    seroi30 = max(ifelse(sinterval==30,seroi,-99)),
    seror30 = max(ifelse(sinterval==30,seror,-99)),
    diff30i = seroi-seroi30,
    diff30r = seror-seror30
  ) %>%
  select(-seroi30,-seror30)

#-----------------------------
# save the simulation results
#-----------------------------
saveRDS(sim_res2,file=here::here("output","asembo-imputation-simulation-results.rds"))
```

## Estimate empirical seroconversion rates

In the primary analysis, we combined information across multiple antigens for each pathogen when we estimated seroconversion and seroreversion rates. In this simulation, we did not attempt to model the joint dynamics of multiple antigens. To benchmark the simulation, we need to estimate seroconversion rates using information from only a single antibody response for each pathogen. The differences between estimates based on joint- vs. single antigen seroconversion rates are slight, but this helps ensure a consistent benchmark for the simulation results.

```
#-----------------------------
# estimate seroincidence rates
# for conversion and reversion
# estimate SEs with a bootstrap
#-----------------------------
#-----------------------------
# filter to just 1 obs per child
# (enrolment information)
# convert ages from months to years
#-----------------------------
dl3 <- dl2 %>% 
  group_by(antigenf,childid) %>%
  mutate(measA=ifelse(time=="A",1,0), measA=max(measA),
         measB=ifelse(time=="B",1,0), measB=max(measB),
         seroposA=ifelse(time=="A",seropos,NA),
         seroposA=max(seroposA,na.rm=TRUE),
         seroposB=ifelse(time=="B",seropos,NA),
         seroposB=max(seroposB,na.rm=TRUE),
         seroi=ifelse(seroposB==1 & seroposA==0,1,0),
         seror=ifelse(seroposB==0 & seroposA==1,1,0),
         agediff=agediff/12
  ) %>%
  filter(time=="A")

#-----------------------------
# estimate time at risk
# for seroconversion and reversion
# assumed to be 1/2 of time
# between measurements
# if indivs are seropositive
# at measurement 1 they are
# not at risk for seroconversion
# (and vice-versa for seroreversion)
#-----------------------------
dl4<- dl3 %>%
  mutate(ptc = ifelse(seropos==0,agediff,0),
         ptr = ifelse(seropos==1,agediff,0),
         ptc = ifelse(seroi == 1 | seror == 1 , agediff / 2, ptc),
         ptr = ifelse(seroi == 1 | seror == 1, agediff / 2, ptr)
  )

#-----------------------------
# estimate incidence rates
# for each antibody
# units are episodes per child-year
#-----------------------------
rate_ests <- dl4 %>%
  group_by(antigenf) %>%
  summarize(ni=sum(seroi,na.rm=T),
            nit=sum(ptc,na.rm=T),
            nr=sum(seror,na.rm=T),
            nrt=sum(ptr,na.rm=T)) %>%
  mutate(seroi=ni/nit,
         seror=nr/nrt)

#-----------------------------
# get bootstrap CIs
#-----------------------------
dboot <- dl4 %>%
  group_by(pathogen,antigen,antigenf) %>%
  select(pathogen,antigen,antigenf,seroi,seror,ptc,ptr)

set.seed(123)
bootests <- foreach(brep=1:1000,.combine=rbind) %dopar% {
    di <- sample_frac(dboot,size=1,replace=TRUE) %>%
      summarize_all(function(x) sum(x,na.rm=TRUE))
    }

rate_cis <- bootests %>% 
  group_by(pathogen,antigen,antigenf) %>%
  mutate(sero_c=seroi/ptc,sero_r=seror/ptr) %>%
  summarize(seroi_lb = quantile(sero_c,probs=c(0.025)),
            seroi_ub = quantile(sero_c,probs=c(0.975)),
            seror_lb = quantile(sero_r,probs=c(0.025),na.rm=T),
            seror_ub = quantile(sero_r,probs=c(0.975),na.rm=T)
            )

rate_ests <- left_join(rate_ests,rate_cis,by="antigenf") %>%
  select(pathogen,antigen,antigenf,ni,nit,starts_with("seroi"),nr,nrt,starts_with("seror"))

knitr::kable(rate_ests %>% select(-pathogen,-antigen),
  digits = c(0, 0,2,2,2,2, 0,2,2,2,2),
  caption = "Estimates of seroconversion and seroreversion rates among children in Kenya, 2013.",
  col.names = c("Antibody", "events", "child-years\nat risk", "rate", "min95", "max95", "events", "child-years\nat risk", "rate", "min95", "max95")
) %>%
  kable_styling(bootstrap_options = c("striped"), full_width = TRUE) %>%
  add_header_above(c(" " = 1, "Seroconverion" = 5, "Seroreversion" = 5))
```

Estimates of seroconversion and seroreversion rates among children in Kenya, 2013.

|  | Seroconverion | | | | | Seroreversion | | | | |
| --- | --- | --- | --- | --- | --- | --- | --- | --- | --- | --- |
| Antibody | events | child-years at risk | rate | min95 | max95 | events | child-years at risk | rate | min95 | max95 |
| Giardia VSP-5 | 61 | 86.75 | 0.70 | 0.54 | 0.90 | 2 | 25.25 | 0.08 | 0.00 | 0.21 |
| Cryptosporidium Cp17 | 79 | 75.88 | 1.04 | 0.86 | 1.27 | 9 | 36.12 | 0.25 | 0.10 | 0.40 |
| E. histolytica LecA | 12 | 105.83 | 0.11 | 0.06 | 0.17 | 3 | 6.17 | 0.49 | 0.00 | 1.10 |
| Salmonella LPS group B | 26 | 100.00 | 0.26 | 0.17 | 0.37 | 8 | 12.00 | 0.67 | 0.28 | 1.11 |
| ETEC LT B subunit | 41 | 12.83 | 3.19 | 2.67 | 3.71 | 0 | 99.17 | 0.00 | 0.00 | 0.00 |
| Campylobacter p18 | 28 | 8.67 | 3.23 | 2.68 | 3.73 | 0 | 103.33 | 0.00 | 0.00 | 0.00 |

## Figure: seroconversion rates

**Figure caption:** Seroconversion rates (force of infection) estimated through simulation of daily IgG boosting and waning kinetics with different measurement frequencies. Simulations were based on antibody boosting and decay parameters estimated from Kenyan children ages 4-17 months old. Points represent each of the 100 estimates from the simulated datasets and box plots summarize their distribution. Horizontal dashed lines and shaded regions mark the empirical seroconversion rates and 95% confidence intervals estimated in Kenyan cohort, which was measured twice over 6 months (approximate 180 day sampling interval).

```
sim_res_plot <- sim_res2 %>%
  ungroup() %>%
  mutate(sinterval=factor(sinterval))

pcols <- cbPalette[c(2,3,4,6,7,8)]
simres_ratep <- ggplot(data=sim_res_plot,aes(y=seroi,color=antigenf)) +
  facet_wrap(~antigenf,ncol=2,scales="free_y")+
  geom_boxplot(aes(x=sinterval),fill=NA,outlier.shape=NA,color=NA)+
    
  
  # add shading for rates estimated in the study
  geom_rect(data=rate_ests,
            aes(ymin=seroi_lb,ymax=seroi_ub,xmin=0,xmax=5),
            color=NA,alpha=0.2)+
  geom_hline(data=rate_ests,aes(yintercept=seroi),color="gray40",linetype="dashed")+

  geom_jitter(aes(x=sinterval),width=0.25,alpha=0.3,pch=19,size=0.3)+
  geom_boxplot(aes(x=sinterval),fill=NA,outlier.shape=NA,color="black",lwd=0.2)+
  
  # scale_y_continuous(breaks=seq(0,4,by=0.5))+
  scale_color_manual(values=pcols)+
  coord_cartesian(xlim=c(1,3))+
  labs(y="seroconversion rate per child-year (force of infection)",x="sampling interval, days") +
  theme_minimal()+
  theme(
    legend.position="none",
    strip.text=element_text(size=8)
  )

simres_ratep
```

```
# save a figure
# ggsave(filename = here::here("figs","asembo-interval-sim-rate-ests.pdf"), plot = simres_ratep,device = cairo_pdf, width = 3.4, height = 4.5)

# print medians
sim_res_plot %>%
  group_by(antigenf,sinterval) %>%
  select(antigenf,sinterval,seroi,seror) %>%
  summarize_all(median) %>%
  knitr::kable(
  digits = c(0,0,2,2),
  caption = "Median seroconversion and seroreversion rates per year at risk across 100 simulated datasets downsampled at different frequencies.",
  col.names = c("Antibody", "Sampling\ninterval (days)","Seroconversion\nrate","Seroreversion\nrate")
) %>%
  kable_styling(bootstrap_options = c("striped"), full_width = TRUE)
```

Median seroconversion and seroreversion rates per year at risk across 100 simulated datasets downsampled at different frequencies.

| Antibody | Sampling interval (days) | Seroconversion rate | Seroreversion rate |
| --- | --- | --- | --- |
| Giardia VSP-5 | 30 | 0.66 | 0.39 |
| Giardia VSP-5 | 90 | 0.65 | 0.24 |
| Giardia VSP-5 | 180 | 0.53 | 0.11 |
| Cryptosporidium Cp17 | 30 | 1.30 | 0.42 |
| Cryptosporidium Cp17 | 90 | 1.14 | 0.25 |
| Cryptosporidium Cp17 | 180 | 0.88 | 0.10 |
| E. histolytica LecA | 30 | 0.40 | 2.91 |
| E. histolytica LecA | 90 | 0.21 | 1.84 |
| E. histolytica LecA | 180 | 0.09 | 1.07 |
| Salmonella LPS group B | 30 | 1.12 | 3.12 |
| Salmonella LPS group B | 90 | 0.50 | 1.59 |
| Salmonella LPS group B | 180 | 0.22 | 0.61 |
| ETEC LT B subunit | 30 | 10.91 | 0.06 |
| ETEC LT B subunit | 90 | 5.94 | 0.03 |
| ETEC LT B subunit | 180 | 3.06 | 0.00 |
| Campylobacter p18 | 30 | 9.47 | 0.02 |
| Campylobacter p18 | 90 | 5.41 | 0.01 |
| Campylobacter p18 | 180 | 3.10 | 0.00 |

The results of the simulations show thatseroconversion rates estimated with a 180 day sampling interval were internally consistent with the empirical estimates from the cohort. The simulations show that in cases of nearly continuous IgG boosting and waning, less frequenty measured cohorts will underestimate force of infection, but the magnitude of difference was < 1 episode per year for all pathogens except for ETEC and *Campylobacter* (below).

## Figure: difference in rates

**Figure caption:** Simulation-based estimates of the difference in seroconversion rates (force of infection) in the Asembo cohort with longer sampling intervals. Estimates were generated through simulation of longitudinal IgG boosting and waning kinetics with daily resolution using boosting and decay parameters estimated from Kenyan children ages 4-17 months old, which were then down-sampled at different frequencies. Points represent each of the 100 estimates from the simulated datasets and horizontal lines indicate median differences from the reference sampling interval of 30 days. Across pathogens, median differences in rates were < 1 episode per year with the exception of ETEC LT B subunit and *Campylobacter* p18, which were much higher.

```
pcols <- cbPalette[c(2:4,6:8)]
sim_res_diff <- sim_res_plot 

simres_diffp <- ggplot(data=sim_res_diff,aes(x=factor(sinterval),y=diff30i,color=antigenf)) +
  facet_wrap(~antigenf,ncol=2,scales="free_y")+
  geom_jitter(width=0.15,alpha=0.3,pch=19,size=0.5)+
  stat_summary(fun.y = median, fun.ymin = median, fun.ymax = median,
                 geom = "crossbar", width = 0.5,color="black",size=0.2)+
  annotate("text",x=1.5,y=0,label="ref.",size=2,color="gray20")+
  scale_color_manual(values=pcols)+
  labs(y="difference in seroconversion rate per child-year, 100 simulations",x="sampling interval, days") +
  theme_minimal()+
  theme(
    legend.position="none",
    strip.text=element_text(size=8)
  )

simres_diffp
```

```
# save a figure
# ggsave(filename = here::here("figs","asembo-interval-sim-rate-diff.pdf"), plot = simres_diffp,device = cairo_pdf, width = 3.5, height = 4.5)

# print median differences
sim_res_diff %>%
  group_by(antigenf,sinterval) %>%
  select(antigenf,sinterval,diff30i,diff30r) %>%
  summarize_all(median) %>%
  knitr::kable(
  digits = c(0,0,2,2),
  caption = "Median difference in seroconversion and seroreversion rates per year at risk across 100 simulated datasets downsampled at different frequencies, with a 30-day sampling interval as the reference.",
  col.names = c("Antibody", "Sampling\ninterval (days)","Seroconversion\nrate","Seroreversion\nrate")
) %>%
  kable_styling(bootstrap_options = c("striped"), full_width = TRUE)
```

Median difference in seroconversion and seroreversion rates per year at risk across 100 simulated datasets downsampled at different frequencies, with a 30-day sampling interval as the reference.

| Antibody | Sampling interval (days) | Seroconversion rate | Seroreversion rate |
| --- | --- | --- | --- |
| Giardia VSP-5 | 30 | 0.00 | 0.00 |
| Giardia VSP-5 | 90 | -0.02 | -0.14 |
| Giardia VSP-5 | 180 | -0.14 | -0.29 |
| Cryptosporidium Cp17 | 30 | 0.00 | 0.00 |
| Cryptosporidium Cp17 | 90 | -0.16 | -0.16 |
| Cryptosporidium Cp17 | 180 | -0.42 | -0.32 |
| E. histolytica LecA | 30 | 0.00 | 0.00 |
| E. histolytica LecA | 90 | -0.19 | -1.07 |
| E. histolytica LecA | 180 | -0.30 | -1.89 |
| Salmonella LPS group B | 30 | 0.00 | 0.00 |
| Salmonella LPS group B | 90 | -0.61 | -1.52 |
| Salmonella LPS group B | 180 | -0.90 | -2.49 |
| ETEC LT B subunit | 30 | 0.00 | 0.00 |
| ETEC LT B subunit | 90 | -5.05 | -0.01 |
| ETEC LT B subunit | 180 | -7.86 | -0.06 |
| Campylobacter p18 | 30 | 0.00 | 0.00 |
| Campylobacter p18 | 90 | -4.10 | -0.01 |
| Campylobacter p18 | 180 | -6.37 | -0.02 |

# Session Info

```
sessionInfo()
```

```
## R version 3.5.3 (2019-03-11)
## Platform: x86_64-apple-darwin15.6.0 (64-bit)
## Running under: macOS High Sierra 10.13.6
## 
## Matrix products: default
## BLAS: /System/Library/Frameworks/Accelerate.framework/Versions/A/Frameworks/vecLib.framework/Versions/A/libBLAS.dylib
## LAPACK: /Library/Frameworks/R.framework/Versions/3.5/Resources/lib/libRlapack.dylib
## 
## locale:
## [1] en_US.UTF-8/en_US.UTF-8/en_US.UTF-8/C/en_US.UTF-8/en_US.UTF-8
## 
## attached base packages:
## [1] grid      parallel  stats     graphics  grDevices utils     datasets 
## [8] methods   base     
## 
## other attached packages:
##  [1] psych_1.8.4              tmle_1.3.0-1            
##  [3] SuperLearner_2.0-25-9000 nnls_1.4                
##  [5] mgcv_1.8-27              ROCR_1.0-7              
##  [7] gplots_3.0.1             tmleAb_0.3.4            
##  [9] nlme_3.1-137             gridExtra_2.3           
## [11] RColorBrewer_1.1-2       ellipse_0.4.1           
## [13] scales_1.0.0             knitr_1.22              
## [15] mixtools_1.1.0           viridis_0.5.1           
## [17] viridisLite_0.3.0        foreign_0.8-71          
## [19] lubridate_1.7.4          doParallel_1.0.11       
## [21] iterators_1.0.9          foreach_1.4.4           
## [23] kableExtra_0.8.0.0001    forcats_0.3.0           
## [25] stringr_1.4.0            dplyr_0.8.0.1           
## [27] purrr_0.3.2              readr_1.1.1             
## [29] tidyr_0.8.3              tibble_2.1.1            
## [31] ggplot2_3.1.1            tidyverse_1.2.1         
## [33] here_0.1                
## 
## loaded via a namespace (and not attached):
##  [1] httr_1.4.0         jsonlite_1.6       splines_3.5.3     
##  [4] modelr_0.1.2       gtools_3.5.0       assertthat_0.2.1  
##  [7] highr_0.8          cellranger_1.1.0   yaml_2.2.0        
## [10] pillar_1.4.0       backports_1.1.4    lattice_0.20-38   
## [13] glue_1.3.1         digest_0.6.18      rvest_0.3.2       
## [16] colorspace_1.3-2   htmltools_0.3.6    Matrix_1.2-15     
## [19] plyr_1.8.4         pkgconfig_2.0.2    broom_0.4.4       
## [22] haven_2.1.0        gdata_2.18.0       withr_2.1.2       
## [25] lazyeval_0.2.2     cli_1.1.0          mnormt_1.5-5      
## [28] survival_2.43-3    magrittr_1.5       crayon_1.3.4      
## [31] readxl_1.1.0       evaluate_0.13      MASS_7.3-51.1     
## [34] segmented_0.5-3.0  xml2_1.2.0         tools_3.5.3       
## [37] hms_0.4.2          munsell_0.5.0      packrat_0.4.9-3   
## [40] compiler_3.5.3     caTools_1.17.1     rlang_0.3.4       
## [43] rstudioapi_0.9.0   bitops_1.0-6       labeling_0.3      
## [46] rmarkdown_1.12     gtable_0.3.0       codetools_0.2-16  
## [49] reshape2_1.4.3     R6_2.4.0           rprojroot_1.3-2   
## [52] KernSmooth_2.23-15 stringi_1.4.3      Rcpp_1.0.1        
## [55] tidyselect_0.2.5   xfun_0.6
```
