# Supplementary material for: Enteropathogen antibody dynamics and force of infection among children in low-resource settings
Source: eLife. 2019 Aug 19;8:e45594. doi: 10.7554/eLife.45594 (PMC6746552; doi:10.7554/eLife.45594)
Supplement: Transparent reporting form [file elife-45594-transrepform.docx]

***eLife’s* transparent reporting form**

We encourage authors to provide detailed information *within their submission* to facilitate the interpretation and replication of experiments. Authors can upload supporting documentation to indicate the use of appropriate reporting guidelines for health-related research (see [EQUATOR Network](http://www.equator-network.org/%20)), life science research (see the [BioSharing Information Resource](https://biosharing.org/)), or the [ARRIVE guidelines](http://www.plosbiology.org/article/info:doi/10.1371/journal.pbio.1000412) for reporting work involving animal research. Where applicable, authors should refer to any relevant reporting standards documents in this form.

If you have any questions, please consult our Journal Policies and/or contact us: [editorial@elifesciences.org](mailto:editorial@elifesciences.org).

**Sample-size estimation**

- You should state whether an appropriate sample size was computed when the study was being designed
- You should state the statistical method of sample size computation and any required assumptions
- If no explicit power analysis was used, you should describe how you decided what sample (replicate) size (number) to use

Please outline where this information can be found within the submission (e.g., sections or figure legends), or explain why this information doesn’t apply to your submission:

This study used antibody data collected in previous studies. We did not conduct a power analysis because, to our knowledge, there are no closed form sample size equations for the parameters of interest (force of infection estimated through longitudinal and cross-sectional analyses).

We used all samples available from three cohorts (details in Table 1). Narrow confidence intervals on all estimates illustrate empirically that the study had sufficient power for all analytic objectives.

**Replicates**

- You should report how often each experiment was performed
- You should include a definition of biological versus technical replication
- The data obtained should be provided and sufficient information should be provided to indicate the number of independent biological and/or technical replicates
- If you encountered any outliers, you should describe how these were handled
- Criteria for exclusion/inclusion of data should be clearly stated
- High-throughput sequence data should be uploaded before submission, with a private link for reviewers provided (these are available from both GEO and ArrayExpress)

Please outline where this information can be found within the submission (e.g., sections or figure legends), or explain why this information doesn’t apply to your submission:

In Results / Study Populations, we describe the details of sample selection and testing. Table 1 includes number of children and samples from each cohort. We included all samples available.

In Materials and Methods / Multiplex Bead Assays, we report information about technical replicates, including negative and positive controls and coefficient of variation means and ranges.

All individual-participant data used in the analyses are provided through the Open Science Framework (see “Additional Data Files”, below)

**Statistical reporting**

- Statistical analysis methods should be described and justified
- Raw data should be presented in figures whenever informative to do so (typically when N per group is less than 10)
- For each experiment, you should identify the statistical tests used, exact values of N, definitions of center, methods of multiple test correction, and dispersion and precision measures (e.g., mean, median, SD, SEM, confidence intervals; and, for the major substantive results, a measure of effect size (e.g., Pearson's r, Cohen's d)
- Report exact p-values wherever possible alongside the summary statistics and 95% confidence intervals. These should be reported for all key questions and not only when the p-value is less than 0.05.

Please outline where this information can be found within the submission (e.g., sections or figure legends), or explain why this information doesn’t apply to your submission:

All statistical methods are reported and justified in the subsections in Materials and Methods.

For all parameters of interest, we estimated 95% confidence intervals using bootstraps that correctly accounted for any outcome dependence in the data (e.g., repeated measures among children). We have not reported p-values because we did not test dichotomous hypotheses.

All results tables and figures include uncertainty intervals around the estimates, along with the exact values of N used in each analysis.

(For large datasets, or papers with a very large number of statistical tests, you may upload a single table file with tests, Ns, etc., with reference to sections in the manuscript.)

**Group allocation**

- Indicate how samples were allocated into experimental groups (in the case of clinical studies, please specify allocation to treatment method); if randomization was used, please also state if restricted randomization was applied
- Indicate if masking was used during group allocation, data collection and/or data analysis

Please outline where this information can be found within the submission (e.g., sections or figure legends), or explain why this information doesn’t apply to your submission:

Children were not allocated into experimental groups for the purpose of this analysis. Two of the cohorts (Kenya, Tanzania) were originally enrolled as part of randomized intervention trials of water filters (Kenya) and annual azithromycin (Tanzania). Supplementary Information File 1 includes an analysis that demonstrates no effect of intervention on enteropathogen antibody response, justifying pooling the measurements across arms for this analysis.

**Additional data files (“source data”)**

- We encourage you to upload relevant additional data files, such as numerical data that are represented as a graph in a figure, or as a summary table
- Where provided, these should be in the most useful format, and they can be uploaded as “Source data” files linked to a main figure or table
- Include model definition files including the full list of parameters used
- Include code used for data analysis (e.g., R, MatLab)
- Avoid stating that data files are “available upon request”

Please indicate the figures or tables for which source data files have been provided:

All data and computational notebooks used to complete the analyses are available through GitHub and the Open Science Framework ([osf.io/r4av7](https://osf.io/r4av7)). Source data are in .csv and .rds formats. All datasets include companion metadata files with references and dictionaries. Analyses were conducted in R version 3.5.3.

Each figure legend includes hyperlinks to the underlying dataset(s) and computational notebook(s) used to create it, which are archived on GitHub and the Open Science Framework.
